# Recurrent miscalling of missense variation from short-read genome sequence data

**DOI:** 10.1186/s12864-019-5863-2

**Published:** 2019-07-16

**Authors:** Matthew A. Field, Gaetan Burgio, Aaron Chuah, Jalila Al Shekaili, Batool Hassan, Nashat Al Sukaiti, Simon J. Foote, Matthew C. Cook, T. Daniel Andrews

**Affiliations:** 1 Department of Immunology and Infectious Disease, The John Curtin School of Medical Research, The Australian National University, Canberra, Australian Capital Territory Australia; 2 Australian Institute of Tropical Health and Medicine, Centre for Tropical Bioinformatics and Molecular Biology, James Cook University, Cairns, Queensland Australia; 3 Department of Microbiology and Immunology, Sultan Qaboos University Hospital, Seeb, Oman; 4 Department of Medicine, Sultan Qaboos University Hospital, Muscat, Oman; 5 Department of Paediatrics, Allergy, and Clinical Immunology Unit, Royal Hospital, Muscat, Oman; 6 Department of Immunology, Canberra Hospital, Canberra, Australian Capital Territory Australia

**Keywords:** Single nucleotide variant, Miscall, Resampling, Exome, Alignment

## Abstract

**Background:**

Short-read resequencing of genomes produces abundant information of the genetic variation of individuals. Due to their numerous nature, these variants are rarely exhaustively validated. Furthermore, low levels of undetected variant miscalling will have a systematic and disproportionate impact on the interpretation of individual genome sequence information, especially should these also be carried through into in reference databases of genomic variation.

**Results:**

We find that sequence variation from short-read sequence data is subject to recurrent-yet-intermittent miscalling that occurs in a sequence intrinsic manner and is very sensitive to sequence read length. The miscalls arise from difficulties aligning short reads to redundant genomic regions, where the rate of sequencing error approaches the sequence diversity between redundant regions. We find the resultant miscalled variants to be sensitive to small sequence variations between genomes, and thereby are often intrinsic to an individual, pedigree, strain or human ethnic group. In human exome sequences, we identify 2–300 recurrent false positive variants per individual, almost all of which are present in public databases of human genomic variation. From the exomes of non-reference strains of inbred mice, we identify 3–5000 recurrent false positive variants per mouse – the number of which increasing with greater distance between an individual mouse strain and the reference C57BL6 mouse genome. We show that recurrently miscalled variants may be reproduced for a given genome from repeated simulation rounds of read resampling, realignment and recalling. As such, it is possible to identify more than two-thirds of false positive variation from only ten rounds of simulation.

**Conclusion:**

Identification and removal of recurrent false positive variants from specific individual variant sets will improve overall data quality. Variant miscalls arising are highly sequence intrinsic and are often specific to an individual, pedigree or ethnicity. Further, read length is a strong determinant of whether given false variants will be called for any given genome – which has profound significance for cohort studies that pool datasets collected and sequenced at different points in time.

**Electronic supplementary material:**

The online version of this article (10.1186/s12864-019-5863-2) contains supplementary material, which is available to authorized users.

## Background

Significant effort has been made to validate the abundant genetic variation identified from personal genome sequencing [1, 2], yet it remains impractical to exhaustively validate rare or novel variants identified in most individuals. Genomic sequence can be easily obtained in clinical care, but subsequently encounters the difficulty of pathogenic variant identification [3]. Generally, pathogenic variants are not found at high population frequencies and, subsequently, rare variants are prioritised in searching for causal mutations [3–5]. In this context, trace levels of spurious SNV miscalls from short-read sequencing have a disproportionately large impact [6] and may lead to an incorrect diagnosis of pathogenicity.

Until very recently, the history of mammalian genome sequencing has been a progression towards shorter sequencing reads and now relies heavily on aligning these to a reference genome [7]. Highly similar genomic regions are difficult to resolve with short-read information and read misalignment is a prevailing source of variant miscalls [8]. Algorithmically, for example, the Burrows-Wheeler Transformation method implemented by the BWA tool [9] must resort to random read assignment between highly similar regions should their mapping quality score fail to differentiate them.

When mapping short-read data to a reference genome, read misalignment has been identified as the predominant source of incorrect variant calls [8]. Misalignment of reads in redundant genomic regions is often highly specific to the given genome sequence from which it is derived. It has remained difficult to appraise the quality of single nucleotide variant sets identified for any given individual genome sequence obtained from short-read sequencing data. For any given location within the genome it is possible to calculate how unique that sequence may be – or how easy it is to uniquely align (or map) a short sequence read to that region. Such mappability scores may be calculated genome-wide for any genome reference sequence [10]. For paralogous coding regions resulting from recent gene duplication, the mappability of any given base in these genes will be low. As the mappability of a region decreases to the point where sequence variation between regions approaches the inherent rate of sequencing error, the chances of read misalignment increase substantially. In such cases, the true variation between paralogous regions may become miscalled as variants in similar regions due to the mis-assignment of reads between these regions. For a given reference sequence, variants that may potentially arise is this way may be catalogued to create a black-list for subsequent filtering of miscalled variation [11].

We could replicate and catalogue these recurrent miscalls for any given exome by only ten rounds of read resampling, realignment and recalling. We identify that recurrent false positives are almost wholly present in databases of human sequence variation and demonstrate how each individual sample generates a unique set of recurrent false positive variants. We show that these variants correlate with redundant coding regions of the genome, including the non-coding sequence surrounding these recently-duplicated regions. Miscalled variants caused by this mechanism are frequently found within genes that might legitimately being clinically-actionable and thus presents a risk to patients with disease phenotypes that correlate with the miscalled gene variant. The strong dependence of read length and miscalled variants also is a pitfall to comparison of cohorts sequenced at different times and with different technologies. Identifying and removing recurrent false positive variants, while computationally more expensive, is simple to achieve and allows their removal from the set of true variant calls for a given genome.

## Results

### Recurrent false positive variants identified from inbred mice

**Fig. 1:**
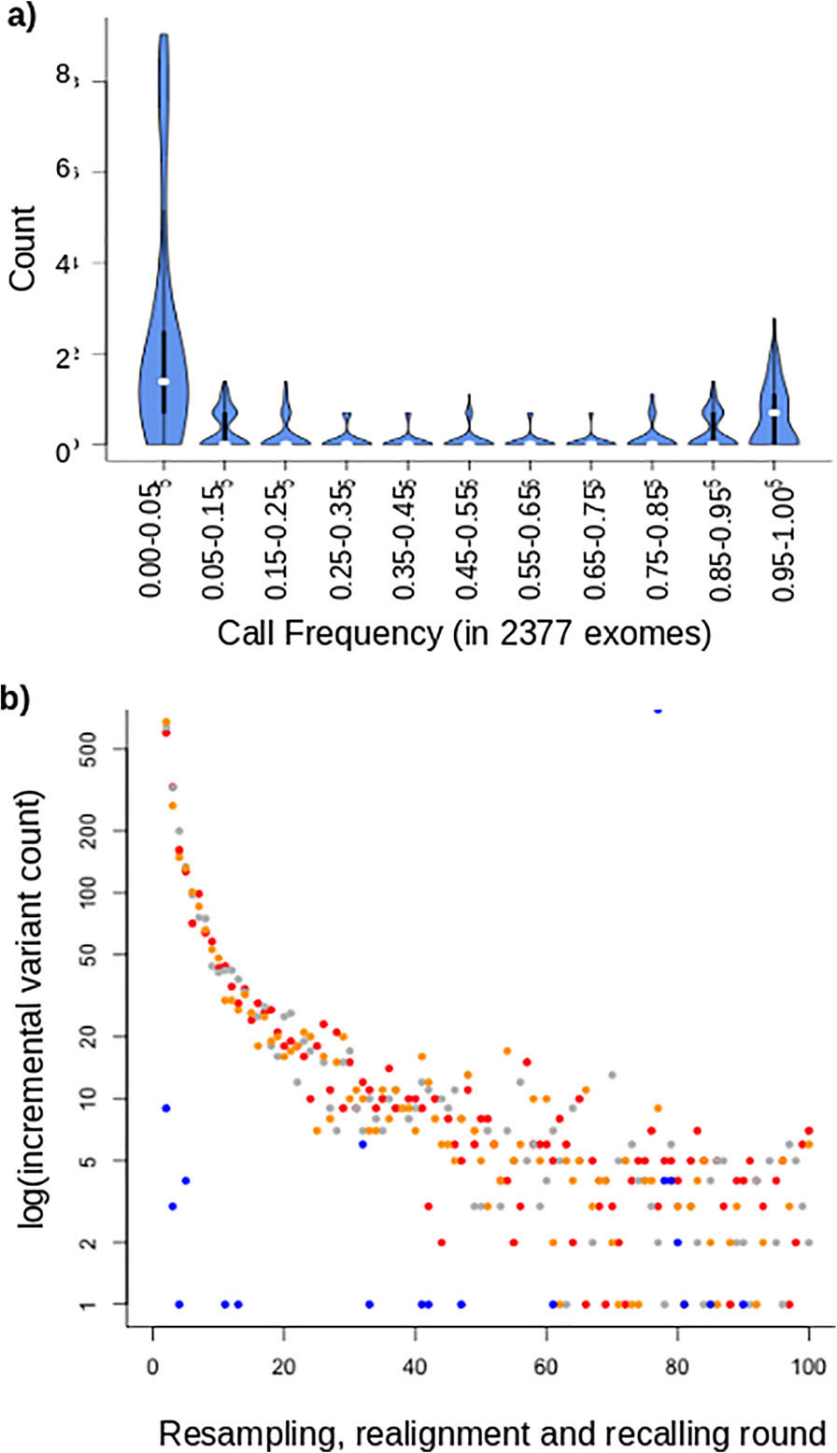
Recurrent false positive variant calls result from difficulties encountered with alignment of short reads to complex mammalian genomes. **a** The frequencies of observed variant calls from 2314 exomes of inbred mice show an intermediate category of recurrent-yet-intermittent SNVs, between the frequency extremes of fixed strain-specific variation and the rare, pedigree-specific induced mutation. **b** Recurrent false positive variants can be replicated for a given individual sequence through randomly sampling short reads, realigning these to a reference genome and recalling sequence variants. Variants that are only intermittently called accumulate after multiple cycles of this process and increase in number following an approximately Poisson distribution. Blue dots show the smaller number of recurrent false positive variants obtained from sampling a C57BL6 mouse and realigning it to itself. Greater numbers are obtained through simulation with three non-reference mouse strains FVB (orange), CBA (red), and C3H (grey)

We have previously generated a large dataset of mouse exomes from inbred C57BL6 mice harbouring random, N-ethyl-N-nitrosourea (ENU) induced mutations [12]. In addition to the 30–60 induced mutations present per pedigree, we observed a category of variant calls that recurred at seemingly random sites in an intermittent mode. These did not validate with genotyping and were not heritable [12]. We refer to these as Recurrent False Positive (RFP) variants. From 2114 sequenced mouse exomes, we identified at total of 104,303 unique SNV sites, the bulk of which are strain-specific variation, but also include ENU-induced mutations (https://databases.apf.edu.au/mutations/) and RFP variants. Figure 1a shows the frequency distribution of all SNVs identified in this population of exome sequences. Strain-specific variation occurs at a frequency approaching 100% and, conversely, ENU induced mutations were pedigree-specific at very low frequencies (< 1%). RFP variants are comparatively fewer and occur at intermediate frequencies between these extremes, conservatively between 5 and 95% in our mouse exome population.

Using these population frequency thresholds, we identified 649,984 variants at 708 unique sites at which RFP variants occur in our data. A distinguishing feature of these variants (compared to randomly chosen single nucleotide positions) is they occur in genomic regions with significantly lower alignability ([10], t-test *p* < 2.2e-16). Previous work has demonstrated that redundant genomic sequences correlate with low alignability scores and represent one cause of read misalignment [8]. Another cause of low alignability is the quality of the reference genome. With variant calling from mouse exomes, we routinely needed to filter 42.5% fewer RFP SNVs when aligning to the improved mm10 reference genome compared to the mm9 reference. Collectively, these factors provide a cogent explanation for the miscalling of RFP variants.

### Recapitulation of mouse common variants through simulation

We were able to reproduce RFP variant calls from any given single mouse exome by simulation. Through multiple rounds of resampling of short reads, realigning these with the mouse reference genome and recalling sequence variants we could reproduce more than half of the RFP variants identified from our population of C57BL6 mouse exomes. In this process, reads were redundantly and randomly sampled from a full exome sequence to extract 80 million 120 bp reads. Each sampled read included randomly introduced sequence errors at a base frequency of 1% to mimic the observed error rate encountered during resequencing with current short-read sequencing technology. These simulated reads were subsequently realigned to the reference genome and variants recalled (called an additional time from the new sequence alignment). Each simulated round from the same exome produced a number of intermittent variant calls that were not reproduced in every round, similar to the RFP variants. Variants called with > 95% frequency were predominantly true strain variation and were discarded. Further rounds of this procedure with the same exome incrementally increased the unique RFP variants identified (Fig. 1b). The RFP variants obtained in this manner incrementally replicates a subset of the false positive variants observed from actual resequencing. 380 iterations of this resampling, realignment and recalling of the colony C57BL6 genome produced 656 RFP variants, matching 367 (51.8%) of the set observed from actual exome data. A greater number of RFP variants are called when aligning a non-reference mouse strain with the reference. We repeated the resampling, realignment and recalling procedure with three more distant strains FVB [13], CBA and C3H ([14] (Fig. 1b). The incremental increase of RFP variants with repeated iteration approximated a Poisson distribution with λ = ~ 1. Just 10 rounds of resampling, realignment and recalling produced 70% of the final total of intermittent variants observed with 100 rounds.

The FVB, CBA and C3H genetic backgrounds produced thousands of RFP variants per genome and these variants variably overlap by between one to two thirds in each mouse strain (Fig. 1c). The greater overlap of RFP variants between the more closely related C3H and CBA strains demonstrates how sequence divergence of non-reference strains gives rise to strain-specific variation. The degree of this sequence difference between reference and individual genome substantially contributes to the quantity and distribution of RFP variants in any individual. This has large implications for detecting causal variants in human disease, as the genetic background of any individual will generate a sample-specific set of RFP variants not relevant to their disease.

### Recurrent false positive variants from human sequence data

RFP variants in human sequences could also be identified with the same sampling, realignment and calling method applied to mouse sequences. We generated a catalogue of RFP variants for the HapMap individual NA12878 (Platinum Genome [15]; whole genome; Additional file 1: Table S1) and compared these with the Genome Aggregation Database (GnomAD; gnomad.broadinstitute.org). Almost, but not all, RFP variants for this individual (96.7%) were present in this database, noting that the NA12878 individual is already present in GnomAD and that RFP variants arise stochastically due to the random sampling of short reads and sequencing error. As with the mouse, RFP variants simulated from human short-read data were similarly found more frequently in redundant genomic regions. When all RFP variants from NA12878 were compared with the same number of randomly chosen coding nucleotide sites they showed lower alignability ([10]; t-test *p* < 3.4e-12).

In this study RFP variants were simulated within exonic regions, and visually, the false variants are evenly distributed. We annotated the RFP variants with Variant Effect Predictor [16]. In this individual and from this sequence, RFP variants were identified in coding exons (missense 59.0%; synonymous 22.2%), untranslated exon regions (3′ UTR 4.2%; 5′ UTR 7.7%), critical splice-donor sites (2.1%) and caused gain (3.4%) and loss (1.2%) of Stop codons. Further to this, we appraised whether the RFP variants were subject to being flagged when filtered due to low map quality or other variant quality filters (such as those applied the the GnomAD variant set). Interestingly, 80.2% of the RFP variants that matched a GnomAD variant for this NA12878 individual were flagged as PASS and only 19.8% non-PASS (combined VQSRTranches filters 12.4%; InbreedingCoeff filter 4.4%; AC_Adj0 filter 3.1%).

Further RFP variant cataloguing analysis was also performed with genomes of two further individuals of ethnicities not dominant in the GnomAD set (an Omani and an Indigenous Australian from the Tiwi community; Fig. 1d). The number of RFP variants in each of these individuals was broadly similar, with an average of 334.5 per individual. The majority are unique to each individual and the proportion of RFP variants that are unique to a given individual varies substantially, though not predictably with, say, sequence divergence from the human reference sequence. The RFP variants per individual were also almost entirely present in the GnomAD database, with population frequencies ranging from common to rare – and importantly, each individual possessed a small number that were unique (Omani, 1.8%; Indigenous Australian, 3.1%; NA12878, 3.3%).

### Sources of recurrent false positive variants

We investigated, by hand, a randomly-selected subset of the RFP variants recapitulated in the above human sequences. Twelve examples of these are shown in Additional file 2: Table S2. For each RFP variant, the surrounding exon coding sequence was used as a query sequence for homology search against the human genome reference sequence (hg37d5 assembly) with BLASTN [17]. Each exon containing an RFP variant had matches to paralogous sequences that included one or several with sequence identity generally greater than 98%. The paralogous regions are mostly between coding regions and their associated untranslated regions, yet frequently include non-coding paralogous regions present as psuedogenes.

While read mismapping is related to the difficulty an aligner encounters with short-read data, variant callers may differ in their propensity to make RFP variant calls from the same alignment. Our mouse variant calls made with real exomes derived from a mutagenised population of thousands of laboratory mice were performed over time using SAMtools, and we have not replicated these. However, the variant calls for the resampled human data here were made with both SAMtools and GATK (see Methods). Direct comparison was made by making variant calls on the NA12878 individual using the same alignments generated by 30 rounds of read resampling and realignment. Hence, variant callers were working from the same set of read misalignments in each replicate. Interestingly, both variant callers produce RFP variant calls of similar propensity (SAMtools: 334; GATK: 398) but these only overlap by 64 variants, less than a fifth in both cases. Clearly, more four-fifths of RFP variants could be identified and removed by excluding variants not identified by both callers.

### Gene distribution of recurrent false positive variants

RFP variants are skewed towards redundant regions of the genome and the genes in which these occur reflects this. In total, 1458 human genes were identified containing one or more RFP variants. Six of the top ten RFP variant-containing genes are HLA genes (HLA-DRB1, 59; HLA-B, 56; HLA-DRB5, 39; HLA-DQB1, 36; HLA-A, 24; HLA-DQA1, 19). The other members of the top ten include a mucin gene (MUC4, 32 RFP variants), the autoimmunity-associated LILRB3 [18] RFP variants) and two others (MAGEC1, 24 and DSPP, 33). The full list of genes that contain more than one RFP variant is included in Additional file 3: Table S3. Of these top ten RFP variant-containing genes, five of the six HLA genes contain pathogenic variants in the ClinVar database [19]. Of the hundred-most RFP variant-containing genes, another five genes are similarly clinically relevant. A total of 138 (138/1458 = 9.5%) clinically-relevant genes associated with disease phenotypes in the ClinVar database were identified to contain RFP variants in the cohorts we analysed. Conversely, redundant gene families were well represented among the RFP variant-containing genes, and include the mucins (8 genes), zinc-finger (38 genes), collagen (17 genes) and solute carrier protein genes (27 genes).

### Sequence-specificity of recurrent false positive variants

**Fig. 2:**
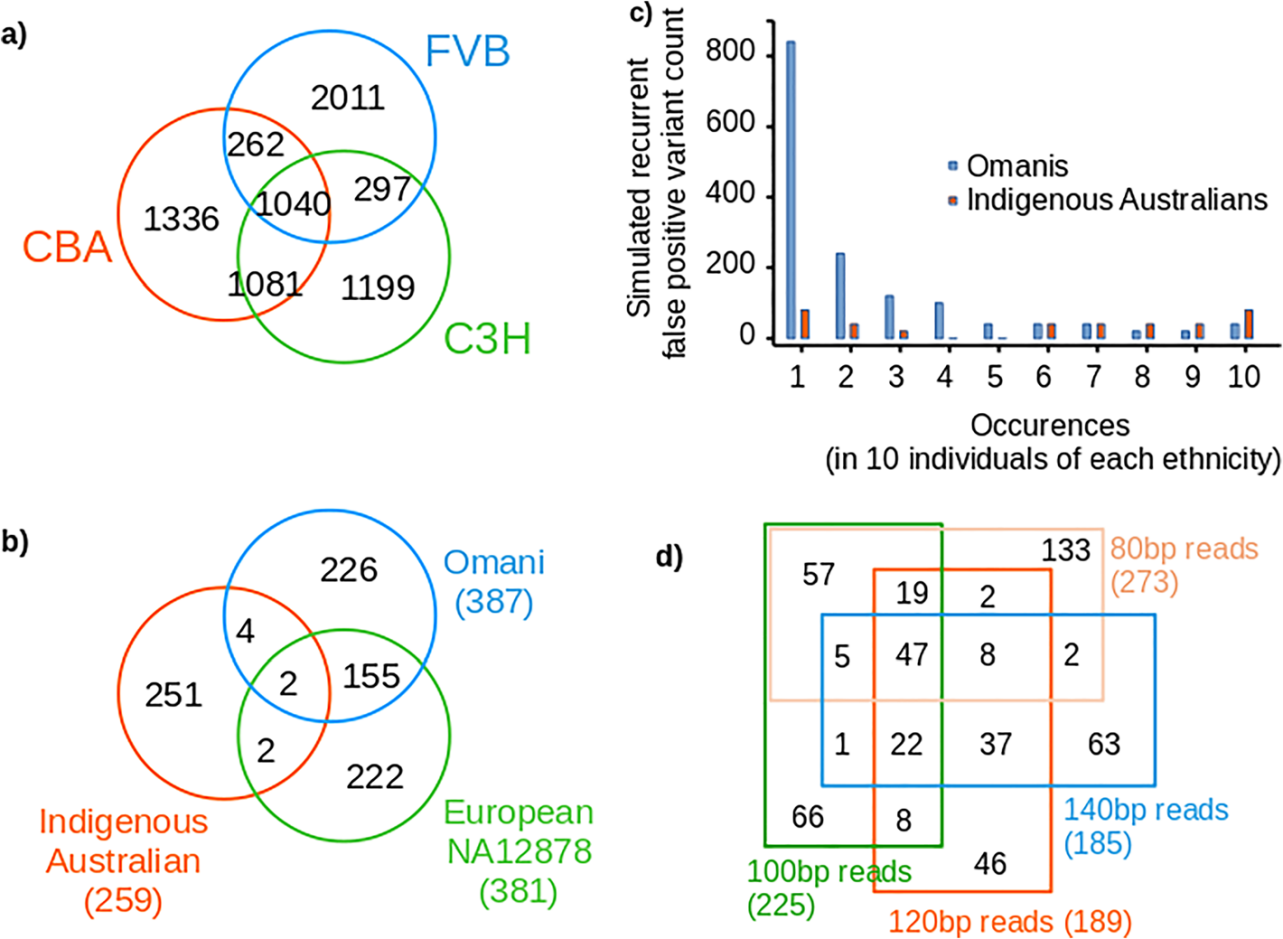
**a** Recurrent false positive variants arise in a sequence-specific manner in three mouse strains. Similarities between these variant sets closely mirror the sequence similarity and relatedness between individual genomes. **b** Likewise, with human sequences, while a small set of recurrent false positive variants are common between all four individuals, the majority are highly ethnically-dependent to individual-specific. **c** Further to this, simulation of recurrent false positive variants from a closely related group of Omani individuals indicates that most are individual-specific, with a smaller number being population-specific. **d** Effect of read length on identification of Recurrent False Positives. Simulation of recurrent false positive variants with different length short-reads from the same genome (NA12878) demonstrates that most of these false positives are specific to read length

The sequence-specificity and covariation with ethnicity of RFP variants was further investigated within single ethnic groups. We repeated the analysis with exomes from ten Omani individuals from pedigrees with an inherited predisposition to autoimmune disease and ten Indigenous Australian individuals with a predisposition to kidney disease. The RFP variants from these individuals of the same ethnicity show more similarity than with other groups (Fig. 2c). This is especially apparent for the Indigenous Australian individuals, for whom the RFP variants they hold do not intersect overly with, say, the Omani or NA12878 individuals. The Omani individuals show a strong tendency towards unique RFPs in every individual and less overlap between individuals. Hence there are clear differences between the representative ethnic groups shown here - and this reflects the sequence similarity between the individuals included in each group. In this instance, the catalogue of RFP variants for the Omani individuals is of substantial practical clinical value. Autoimmunity in these individuals could plausibly have been ascribed to predicted-damaging RFP variants in genes with strong associations to lupus (IRF5, LILRB3) or autoimmune hepatitis (C4A). The IRF5 variant in particular was a strong candidate, yet was proven to be miscalled on subsequent genotyping.

### Effect of read length versus ethnicity

The effect of read length on the propensity to cause RFP variants from the same genome was investigated with the NA12878 genome (Fig. 2d). At four incremental read lengths (80, 100, 120 and 140 base pairs) RFP variants were simulated through with 30 rounds of resampling, realignment and recalling from the genome of this individual. As shown in Fig. 2d, the RFP variants simulated in this manner are predominantly unique (0.29–0.48), or overlap modestly with read sets of similar length. The overall number of RFP variants decreases by about a third (32.5%) as read lengths increase from 80 bp to 140 bp.

As the read lengths obtained by sequencing technology has increased, it has become commonplace to pool individuals to produce population cohorts with produced with heterogenous read lengths between samples. We investigated this effect on RFP variant calls on the combined data used in this work, plus that available from the 1000 Genomes Project [18]. From 1000 Genomes data we selected a single genome representative for each broad ethnic group (Additional file 4: Table S4). These data represented 15 different ethnic designations, obtained using a 75 bp read length, but including two samples with 90 bp and 100 bp read lengths, and were combined with the Omani and Indigenous Australian cohorts that were whole genomes sequenced with 150 bp reads (Additional file 4: Table S4). For each genome, RFPs were simulated from 30 rounds of resampling, realignment and recalling of single nucleotide variants. Across the three cohorts that include all individual sequences, plus the platinum genome NA12878 sequence, we created a catalogue of the RFP variants called (Additional file 5: Table S5).

**Fig. 3:**
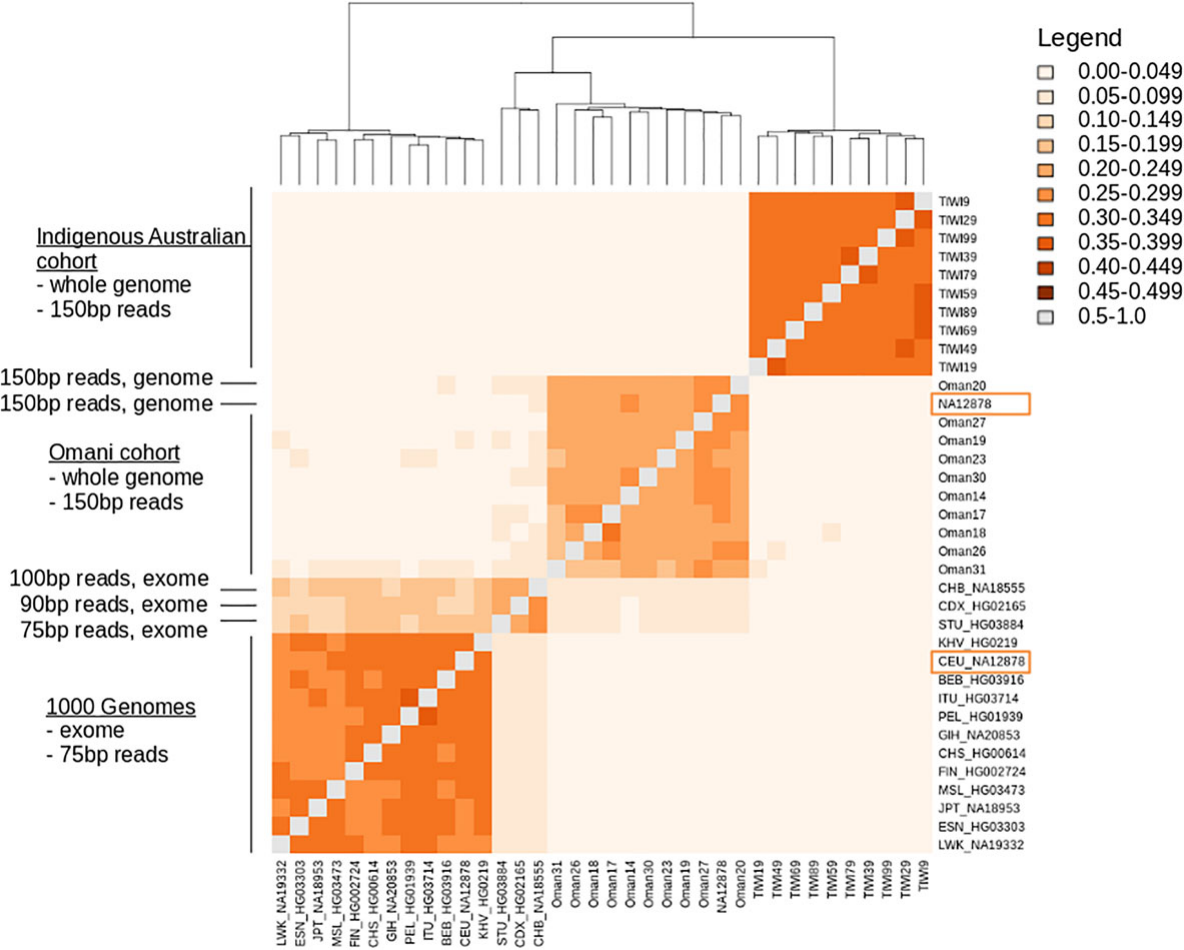
A heatmap of similarity between pairs of RFP variant sets between individuals. Labels show population cohorts and sequence metadata, including replicates of the NA12878 genome obtained with slight methodological differences (labelled CEU_NA12878 (from 1000 Genomes) and NA12878). Points in grey are comparisons of the same individual with themselves – and have a similarity score of 1.0, coloured in grey. Increasingly dark shades of orange indicate, as per the legend, increasing pairwise similarities between RFP variant sets obtained for each individual. No individuals had similarity with another individual in this set that had better than 0.5 similarity

Similarities between these RFP variant sets are summarised in Fig. 3 and shows that the Indigenous Australian cohort, the Omani cohort and the 1000 Genomes cohort present distinct subsets of the total RFP variant set identified (Additional file 6: Table S6). One key feature to note in this comparison is that the NA12878 genome occurs twice in this figure – once as en exome of 75 bp reads from the 1000 Genomes Project and again as an platinum genome [15] obtained as 150 bp reads. Given that this genome was obtained twice, it is significant that the RFP variants identified each time do not group together, but instead group with the 1000 Genomes cohort and the Omani cohort. Even stronger than sequence specificity, the RFP variants correlate with another factor that causes genomes sequenced with similar methodologies to be similar.

## Discussion

Our results identify a class of false variant calls that are an inherent factor in reliably realigning short read sequence information to a complex mammalian genome. This class of variants was shown to arise from analysis of both mouse and human sequences. Significantly, we find that human recurrent false positive SNVs are almost completely represented in human population sequence databases, such as GnomAD. Further, these recurrent false positive SNVs may be identified for any given genome sequence through repeated sampling and realignment against a reference sequence. Hence, from this work we show that it is possible to computationally remove the bulk of these spurious SNV calls. Also, we show that sequence read length has a strong influence on the RFP variants called for a given genome – and these read length similarities are stronger than those caused by sequence similarity. This effect of read length and false positive variants calls is highly relevant to cohort studies where genome sequences of affected and unaffected groups were obtained at different times and with different read lengths.

At the heart of these variant miscalls is misalignment of reads between redundant regions of the genome. These redundant regions differ very slightly, so as that the low level of sequencing error inherent in short read data will similar to the true variation that exists between near duplicate sequences in the genome reference. Hence, reads may be stochastically misassigned between these redundant regions and when calling sequence variation, the true differences present in the misaligned reads become called as sequence variation between the re-sequenced and reference genomes. Hence, these miscalls will be recurrent to specific nucleotide sites in any given re-sequenced genome, yet will recur in an intermittent manner due to the stochastic way in which sequencing error and misalignment occurs. Importantly, this will produce some miscalled variants with what will appear as rare sequence variation when these variants are aggregated in human population variation databases. This mechanism of RFP genesis suggests that their removal can be achieved by both exhaustive cataloguing of RFP variants and/or identifying for a given genome the near-identical regions and the minor sequence differences that exist between them. In practice, the exhaustive cataloguing through simulation is simple to achieve. Even more simply, a quick work-around that will remove more than 80% of RFP variants is to remove variant calls that are not replicated by both of SAMtools and GATK. This work-around is a methodology that has increasingly been gaining acceptance for many other purposes also [20] and is further reason for clinical variants to be made from the union of calls made with multiple callers [21, 22].

Simulation and cataloguing of RFP variants is technically simple, but does substantially increase the computation required to perform this analysis for any given genome. Each simulation round requires computation to simulate a population of reads from a given genome, subsequent realignment of these reads and the calling of sequence variation between this alignment and the reference genome. This is effectively (at least) a ten-times increase in computation for a single genome. However, the cost of computing a single exome or genome (in the order of tens of dollars) is relatively small compared to the cost of generating this sequence data (two orders of magnitude more, at current costs). Hence, increasing this compute cost by a factor of ten is inconsequential should this improve the quality of the derived information substantially. Furthermore, the costs of a misidentified variant that leads to misdiagnosis in a clinical context is difficult to quantify. Yet practically this will easily dwarf both the cost of computation and sequence data generation.

Given that the genesis of the false positive variant calls lies with the misplacement of difficult-to-map reads from near identical regions of the genome, these reads are difficult to map purely because the sequence diversity between regions is similar or less than the inherent rate of sequencing error from short-read sequencing. However, should an aligner have a catalogue of these regions and be aware of the sequence diversity that differentiates these, the real sequence variation might be the anchor that decides to which paralogous region the read should be mapped. The map of near-identical paralogous sequences would need to be more detailed that just expressing sequence map quality, and when a read is being mapped between very similar regions, the map could be used to differentiate inter-paralog variation from read-specific sequence variation and/or sequencing error. Using this map to best solve this problem, the aligner needs to have information of local sequence diversity between near-identical paralogous regions and make read assignment choices based on this information, differentiated from noise due to sequencing error.

Having identified this source of error in variant identification, there may be better ways found to produce reference datasets of RFP variation that can routinely be filtered from variant call sets. As we have shown, these will be specific to the ethnic background of any given genome and argues for the ascertainment of reference population datasets of genomes from diverse ethnic groups. While any reference dataset of RFP variants for a given ethnic group, unless exhaustively ascertained, will almost certainly be incomplete. A further complexity will be the sensitivity of the RFP variant set to the sequence read length for which the genome was obtained. However, it is evident from our analysis that the sequences of other members of a population, even if only a handful, will likely catalogue the majority of the most prevalent RFP variants specific to a given population. This by itself, without multiple rounds of resampling, realignment and recalling on a given genome, may be sufficient to ameliorate the risk of RFP variant-related misdiagnosis to acceptable levels. Generation of such RFP variant reference sets might be most efficiently performed by databases of genomic variation on a routine basis, and comparison of a given personal genome to this data corpus will annotate variation likely to be due to read misalignment.

## Conclusions

Resequencing of individual genomes with short-reads – given the redundancy of complex mammalian genomes – leads to a category of single nucleotide variant miscalls termed Recurrent False Positive variants. Miscalls can be simulated by a resampling, realignment and recalling methodology to facilitate their computational identification for removal. A practical application of this methodology may be the undertaking to curate a community (and potentially clinical) resource of RFP variants, simulated from a broad, ethnically-diverse set of input genomes, sequenced with diverse read lengths. Such a resource may then act as a filter set to remove the majority of RFP variants from an individual variant set with very little additional computational effort.

## Methods

### Mouse recurrent false positive variants

The exomes from 2114 distinct C57BL6 mice were obtained and single nucleotide variation identified as previously described [12]. The frequency of each variant identified from this genome set was counted by determining variant occurrence in the full population of 2114 mice. Recurrent false positive variants were defined as those that were present at frequencies greater than 5% but less that 95%.

### Mouse variation and exome database

Mouse exome data was obtained under ethics approval 2014/61 (Production and phenotyping of exome sequences ENU gene variant mouse pedigrees) to Dr. E Bertram of the Australian National University. The mouse variation data is available from the Missense Mutation Library of the Australian Phenomics Facility (https://databases.apf.edu.au/mutations/).

Exome sequences of the FVB, C3H and CBA mouse strains were derived from the whole genome sequences available for these strains [13, 14].

### Human whole genome data

The genome data analysed in the work comprised both whole genome and exome sequences, obtained with predominantly 150 bp and 75 bp reads, respectively (see Additional file 4: Table S4).

Genome data from Omani individuals were obtained with hospital-based consent to J Al Shekaili (Royal Hospital, Muscat) for the genetic analysis of these individuals. Indigenous Australian genome data from the Tiwi community was obtained with appropriate consent granted to SJ Foote (The Australian National University 2014/663). Data from public genome sequences was obtained from the 1000 Genomes Project (http://1000genomes.org).

The genome sequence of the human NA12878 individual was obtained from the Illumina Platinum Genomes resource [15].

### Simulation of recurrently miscalled variants

To resample, realign and recall variants for any given genome, random reads were sampled from an input genome sequence in Fasta format with the tool WGSim (version 0.3.1; WGSim GitHub repository: http://github.com/lh3/wgsim). The parameters passed to the WGSim tool used were –e 0.01, −r 0, −R 0, −X 0 and –A 1. Synthetic exome sequences for both the mouse genome (mm10) and the human genome (hg37d5) were derived from EnsEMBL BioMart (www.ensembl.org) and consisted of the sequences of all exons, including non-coding exons, and 325 bp of padding upstream and downstream of each exon. From these derived sequences and the WGSim parameters used, mock exome sequence datasets contained 80 million, 120 bp paired-end reads with random sequencing errors at a frequency of 1%. Sequence datasets were then aligned to the chosen reference genome with BWA mem (v0.7.12; [9]) using default parameters. From these alignments, single nucleotide variants were called with SAMtools (v1.3.1; http://www.htslib.org; [23]), using a workflow consisting of rmdup, sort, mpileup and bcftools call. Also, to test tool-independence of human calls, GATK (v3.6.0; https://software.broadinstitute.org/gatk/; [24]) was used with best-practice methodology and parameter sets. Individual exome and whole genome sequences were derived as alternative reference sequences using the FastaAlternateReferenceMaker tool from the GATK suite. A more detailed description of the workflow employed, including example commands, along with scripted execution is available at https://sourceforge.net/projects/recurrentmiscalls/.

## Additional files

Additional file 1: **Table S1.** Recurrent false positive variants for NA12878 individual. (DOCX 31 kb)

Additional file 2: **Table S2.** Near-paralogs of example human recurrent false positive variants. (DOCX 23 kb)

Additional file 3: **Table S3.** Genes that contain one or more RFP variant. (DOCX 23 kb)

Additional file 4: **Table S4.** Genome sequence metadata. (DOCX 22 kb)

Additional file 5: **Table S5.** A catalogue of all recurrent false positive variants identified among 1000 Genome, Omani and Indigenous Australian cohorts. (DOCX 263 kb)

Additional file 6: **Table S6.** Recurrent false positive variants by population cohort. (DOCX 304 kb)

## Data Availability

An aggregate dataset of variants identified in ENU mutagenized is available from the Australian Phenomics Facility (https://databases.apf.edu.au/download/) and includes the possibility of reanimating cryopreserved strains of interest. Exome sequences for particular mouse strains are available on request to TDA. Human data analysed in the study is not publically available and controlled under the relevant ethics approvals covering this data, in rare circumstances the data may available from the corresponding author and approval holders, given sufficient justification.

## Abbreviations

ENU: N-ethyl-N-nitrosourea
RFP: Recurrent false positive
SNV: Single nucleotide variant
