# Supplementary material for: Recurrent miscalling of missense variation from short-read genome sequence data
Source: BMC Genomics. 2019 Jul 16;20(Suppl 8):546. doi: 10.1186/s12864-019-5863-2 (PMC6631443; doi:10.1186/s12864-019-5863-2)
Supplement: Supplementary file 1 — Table S1. Recurrent false positive variants for NA12878 individual. (DOCX 31 kb) [file 12864_2019_5863_MOESM1_ESM.docx]

**Additional file 1: Table S1** Recurrent false positive variants for NA12878 individual (GRCh37 coordinates, comma-separated values)

**RFP,recurrence count (from 30 simulations),median depth (from 30 simultations),Genotype,Chr,Coord ,Rsid,Ref,Var,PASS or non-PASS,InbreedingCoefficient,MapQuality,AlleleFreq(all)**

10:118368606:C,28,23,1/1,10,118368606,rs1049125,T,C,PASS,0.0256,58.08,0.175

10:120919246:C,5,11,1/1,10,120919246,rs2181118,T,C,PASS,0.0532,59.34,0.995

10:124329710:A,25,28,1/1,,,,,,,,,

10:124330427:T,25,20,1/1,10,124330427,rs3013236,C,T,PASS,0.0273,59.47,0.716

10:46321555:A,28,149,1/1,,,,,,,,,

10:47919971:C,1,19,1/1,,,,,,,,,

10:49382926:T,18,22,1/1,10,49382926,rs144837285,G,T,PASS,0.718,29,0.58

10:49388901:T,5,59,1/1,10,49388901,.,C,T,AC_Adj0_Filter,0.2443,26.4,0.061

10:51225872:T,25,175,0/1,10,51225872,.,G,T,AC_Adj0_Filter,0.0989,22,0.045

10:51568378:G,12,23,1/1,10,51568378,rs10761581,T,G,PASS,0.0959,58.72,0.489

10:88992622:T,24,70,0/1,10,88992622,.,C,T,PASS,0.2354,28.23,0.131

10:91487649:T,25,25,1/1,10,91487649,rs12572012,A,T,PASS,0.0224,60,0.218

10:95389041:A,27,28,1/1,10,95389041,rs714550,G,A,PASS,0.0185,59.53,0.35

10:97744383:C,26,31,1/1,10,97744383,rs4488132,T,C,PASS,0.5521,60,0.46

10:97990583:G,24,21,1/1,,,,,,,,,

11:111795085:G,23,25,1/1,11,111795085,rs7124407,C,G,PASS,0.0417,59.58,0.355

11:112123095:G,28,26,1/1,11,112123095,rs2564872,A,G,PASS,0.1062,59.61,0.685

11:117781435:G,23,21,1/1,11,117781435,rs2155194,A,G,PASS,0.0465,60,0.942

11:117789327:C,25,125,0/1,,,,,,,,,

11:118430519:T,26,31,1/1,11,118430519,rs11552421,C,T,PASS,0.0827,59.65,0.187

11:120107411:A,15,20,1/1,11,120107411,rs882856,G,A,PASS,0.2035,59.62,0.428

11:124750453:C,28,37,0/1,,,,,,,,,

11:22881002:T,28,24,1/1,11,22881002,rs3213706,C,T,PASS,0.1095,59.61,0.439

11:60971069:G,28,30,1/1,,,,,,,,,

11:60971694:G,2,9,1/1,11,60971694,rs117494851,A,G,VQSRTrancheSNP99.80to99.90,0.218,29,na

11:71249152:T,7,96,0/1,,,,,,,,,

11:71544269:T,27,36,1/1,,,,,,,,,

11:7656809:A,20,23,1/1,11,7656809,rs11041490,G,A,PASS,0.0393,59.64,0.097

11:76701606:A,14,17,1/1,,,,,,,,,

11:772490:A,16,19,1/1,11,772490,rs12224894,G,A,PASS,0.0697,57.84,0.424

11:82973004:T,28,32,1/1,11,82973004,rs8789,C,T,PASS,0.031,59.65,0.415

11:89531619:G,28,288,0/1,,,,,,,,,

11:95825383:T,19,96,0/1,,,,,,,,,

11:95825407:T,25,172,0/1,,,,,,,,,

12:10041364:A,27,19,1/1,12,10041364,rs1797517,G,A,PASS,0.1307,59.64,0.748

12:10763236:C,27,30,1/1,,,,,,,,,

12:118511677:A,28,26,1/1,,,,,,,,,

12:133049516:G,14,16,1/1,,,,,,,,,

12:40852535:G,25,21,1/1,12,40852535,rs17128233,A,G,PASS,0.0134,60,0.049

12:40876891:A,9,109,0/1,,,,,,,,,

12:52402998:T,26,27,1/1,12,52402998,rs73104710,C,T,PASS,0.0087,59.69,na

12:52696930:C,24,131,1/1,12,52696930,.,A,C,PASS,na,na,na

12:6450945:C,22,23,1/1,12,6450945,rs767455,T,C,PASS,0.0369,59.32,0.374

12:7080212:C,13,80,1/1,12,7080212,rs17857448,T,C,PASS,na,na,1

12:9315209:G,25,25,1/1,12,9315209,rs10771381,A,G,PASS,0.0102,59.63,0.412

12:97098549:C,25,32,1/1,12,97098549,rs11108643,T,C,PASS,0.0638,60,0.746

13:24468329:G,3,12,1/1,13,24468329,rs4067961,A,G,PASS,0.4587,32.95,0.135

13:26148966:T,27,30,1/1,13,26148966,rs6491066,C,T,PASS,0.1122,59.63,0.628

13:28674628:C,22,18,1/1,13,28674628,rs12872889,T,C,AC_Adj0_Filter,0.1214,60,0.026

13:32676114:G,28,22,1/1,13,32676114,rs364995,A,G,PASS,0.0212,59.59,0.605

13:46108853:C,3,16,1/1,13,46108853,rs3014902,T,C,PASS,0.002,60,0.998

13:46108854:A,3,16,1/1,13,46108854,rs56244086,G,A,PASS,0.2156,60,0.831

14:106471448:G,26,148,1/1,,,,,,,,,

14:106471449:A,25,148,0/1,,,,,,,,,

14:22476144:T,26,154,1/1,,,,,,,,,

14:23000062:C,28,24,1/1,14,23000062,rs227003,T,C,PASS,0.0601,59.81,0.706

14:32419331:T,28,29,1/1,14,32419331,rs1278942,C,T,PASS,0.1885,59.74,0.712

14:55864130:G,28,25,1/1,14,55864130,rs8003279,A,G,PASS,0.0379,59.75,0.275

14:60063472:G,26,30,1/1,,,,,,,,,

14:70039805:A,4,38,0/1,,,,,,,,,

14:70039809:C,22,34,0/1,,,,,,,,,

14:74992800:G,15,15,1/1,,,,,,,,,

15:100794363:T,21,15,1/1,15,100794363,rs4369638,C,T,PASS,0.0228,59.62,0.755

15:20743796:T,1,9,1/1,15,20743796,.,C,T,PASS,0.7532,26.17,0.622

15:21071485:G,28,193,1/1,15,21071485,.,A,G,VQSRTrancheSNP99.60to99.80,na,na,0.056

15:32891501:T,28,135,1/1,,,,,,,,,

15:34674006:T,28,155,1/1,15,34674006,.,C,T,VQSRTrancheSNP99.60to99.80,0.0336,22,0.106

15:34820404:C,22,211,0/1,15,34820404,rs151232638,T,C,AC_Adj0_Filter,0.3381,22.45,0.223

15:60690089:G,18,14,1/1,15,60690089,rs12904657,A,G,PASS,0.2167,60,0.691

15:65134221:T,4,14,1/1,15,65134221,.,G,T,PASS,na,na,na

15:82555242:G,23,26,1/1,15,82555242,rs11631813,A,G,PASS,0.0516,59.53,0.187

15:82932833:A,6,145,1/1,,,,,,,,,

15:90320161:A,19,183,0/1,,,,,,,,,

15:93616975:G,17,16,1/1,15,93616975,rs4598860,A,G,PASS,0.1302,59.64,0.55

15:99544429:A,3,13,1/1,15,99544429,rs2593051,G,A,PASS,0.153,58.71,0.639

16:1291318:A,1,385,0/1,,,,,,,,,

16:1291318:C,23,385,0/1,,,,,,,,,

16:25239805:C,27,23,1/1,,,,,,,,,

16:27974487:C,28,28,1/1,16,27974487,rs2051743,T,C,PASS,0.0383,59.59,0.588

16:4445327:T,28,27,1/1,16,4445327,rs3747579,C,T,PASS,0.1102,59.57,0.649

16:72042682:C,4,11,1/1,16,72042682,rs3213422,A,C,PASS,0.2748,60,0.409

16:72822033:T,6,21,1/1,,,,,,,,,

16:82203742:C,27,33,1/1,,,,,,,,,

16:82203758:T,23,29,1/1,16,82203758,rs2303262,C,T,PASS,0.0482,54.98,0.794

16:82203768:T,26,27,1/1,,,,,,,,,

16:90095573:T,23,191,0/1,,,,,,,,,

17:1733399:G,18,23,1/1,17,1733399,rs5030749,A,G,PASS,0.3747,60,0.527

17:18291544:G,1,16,1/1,17,18291544,rs586322,A,G,PASS,0.1038,49.35,0.523

17:19713740:T,24,27,1/1,17,19713740,rs150122,C,T,PASS,0.0734,59.28,0.932

17:35696804:A,28,20,1/1,17,35696804,rs58654829,G,A,PASS,0.1253,46.58,0.717

17:35771468:T,5,16,1/1,17,35771468,rs7211875,C,T,PASS,0.0168,59.44,0.86

17:36339597:T,22,114,1/1,17,36339597,.,G,T,InbreedingCoeff_Filter,na,na,0.421

17:38975158:G,24,97,0/1,,,,,,,,,

17:38975272:T,5,106,0/1,,,,,,,,,

17:60351457:A,1,13,1/1,17,60351457,rs4968502,G,A,PASS,0.0992,37.95,0.325

17:6406883:T,26,21,1/1,17,6406883,rs3809835,C,T,PASS,0.0215,59.64,0.284

17:65212042:T,19,23,1/1,17,65212042,rs8080100,C,T,PASS,0.0089,60,0.163

17:72889676:C,28,77,0/1,,,,,,,,,

17:74288410:T,27,120,0/1,,,,,,,,,

17:74288421:G,28,111,1/1,,,,,,,,,

17:74288421:G,T,2,111,1/1,,,,,,,,,

17:77769127:G,22,17,1/1,,,,,,,,,

17:77769130:T,13,16,1/1,,,,,,,,,

17:79395747:A,11,22,1/1,17,79395747,rs72853236,G,A,PASS,na,na,na

18:14524966:C,25,26,1/1,,,,,,,,,

18:20716021:T,23,24,1/1,,,,,,,,,

18:20953720:A,27,21,1/1,18,20953720,rs8099409,G,A,PASS,0.0392,59.65,0.437

18:28898294:G,25,31,1/1,18,28898294,rs1426310,A,G,PASS,0.0699,59.61,0.434

18:30352078:C,9,140,0/1,,,,,,,,,

18:5410574:G,27,32,1/1,18,5410574,rs3817466,A,G,PASS,0.1865,59.48,0.853

18:59810563:G,20,18,1/1,18,59810563,rs34227891,A,G,PASS,0.0857,60,0.151

18:71928150:T,15,15,1/1,18,71928150,rs7238987,C,T,PASS,0.022,59.61,0.153

19:14769339:G,15,17,1/1,19,14769339,rs4606855,C,G,PASS,0.0384,59.49,0.774

19:14877845:G,11,23,1/1,,,,,,,,,

19:14877848:A,9,21,1/1,,,,,,,,,

19:14877857:A,26,35,1/1,,,,,,,,,

19:1535196:A,1,17,0/1,,,,,,,,,

19:2820105:A,26,30,1/1,19,2820105,rs2537856,G,A,PASS,0.3136,60,0.153

19:35660508:A,28,22,1/1,19,35660508,rs12110,G,A,PASS,0.0341,59.59,0.811

19:38202516:C,1,8,1/1,19,38202516,rs2909097,A,C,PASS,0.0338,59.65,0.997

19:40368733:C,8,229,0/1,,,,,,,,,

19:40389657:G,16,21,1/1,19,40389657,rs148187888,T,G,VQSRTrancheSNP99.90to99.95,na,na,0.093

19:43709654:A,27,24,1/1,,,,,,,,,

19:43709656:G,27,23,1/1,19,43709656,rs11883278,C,G,PASS,na,na,0.881

19:48305538:G,10,106,0/1,,,,,,,,,

19:49558210:G,9,20,1/1,,,,,,,,,

19:49558211:G,11,21,1/1,,,,,,,,,

19:49558216:T,26,28,1/1,19,49558216,rs35728583,C,T,VQSRTrancheSNP99.90to99.95,na,na,0.27

19:49894152:T,25,20,1/1,19,49894152,rs7256629,C,T,PASS,0.017,59.53,0.5

19:54724430:A,22,46,1/1,,,,,,,,,

19:54724431:A,24,45,1/1,,,,,,,,,

19:54871664:C,25,15,1/1,19,54871664,rs74463408,G,C,PASS,0.06,32.77,0.133

19:58038964:A,11,22,1/1,19,58038964,rs12461014,T,A,PASS,0.0181,59.72,0.981

1:115236057:A,6,16,1/1,1,115236057,rs17602729,G,A,PASS,0.0332,59.62,0.087

1:120611964:C,26,24,1/1,,,,,,,,,

1:1423281:A,28,26,1/1,,,,,,,,,

1:144340089:G,21,96,1/1,1,144340089,.,A,G,AC_Adj0_Filter,0.1637,22,na

1:144811810:G,23,41,0/1,1,144811810,.,A,G,VQSRTrancheSNP99.80to99.90,na,na,0.01

1:144811823:C,23,34,1/1,1,144811823,rs9725770,A,C,VQSRTrancheSNP99.80to99.90,na,na,0.025

1:144990002:T,18,32,1/1,,,,,,,,,

1:145299792:G,25,133,0/1,1,145299792,rs61814628,A,G,VQSRTrancheSNP99.80to99.90,na,na,na

1:145299805:C,26,146,0/1,,,,,,,,,

1:145299809:A,22,151,0/1,1,145299809,rs61814630,G,A,VQSRTrancheSNP99.80to99.90,na,na,na

1:145301793:G,24,18,0/1,1,145301793,rs75332562,A,G,InbreedingCoeff_Filter,na,na,0.48

1:145301802:G,11,16,0/1,1,145301802,rs6690575,C,G,InbreedingCoeff_Filter,na,na,0.494

1:145302704:G,11,13,1/1,1,145302704,rs141935360,A,G,VQSRTrancheSNP99.90to99.95,na,na,0.579

1:145311110:C,26,26,1/1,1,145311110,rs141899955,G,C,InbreedingCoeff_Filter,na,na,0.479

1:146409969:C,26,47,1/1,1,146409969,.,A,C,PASS,0.1585,27.73,0.07

1:146414187:A,16,20,1/1,,,,,,,,,

1:152189055:C,17,52,1/1,,,,,,,,,

1:153320372:G,26,13,1/1,,,,,,,,,

1:153320401:A,28,22,1/1,1,153320401,rs12096209,G,A,PASS,0.0365,59.59,0.06

1:162367071:T,23,23,1/1,1,162367071,rs17852003,C,T,PASS,0.0203,59.52,0.301

1:167893759:A,14,14,1/1,1,167893759,rs203861,G,A,PASS,0.0666,60,0.355

1:16899669:T,2,13,1/1,1,16899669,.,C,T,PASS,na,na,0.054

1:16902884:C,28,33,0/1,1,16902884,rs74630591,T,C,InbreedingCoeff_Filter,na,na,0.24

1:16902894:G,3,20,1/1,,,,,,,,,

1:16909208:G,28,90,1/1,1,16909208,.,C,G,VQSRTrancheSNP99.95to100.00,na,na,0.02

1:175129946:T,25,150,0/1,,,,,,,,,

1:207795320:G,7,13,1/1,1,207795320,rs2296160,A,G,PASS,0.0575,60,0.808

1:21807427:T,4,10,1/1,1,21807427,rs116579083,G,T,VQSRTrancheSNP99.80to99.90,0.0321,33.36,na

1:21809014:C,12,38,1/1,1,21809014,.,G,C,PASS,0.3433,30.74,0.37

1:21945530:T,26,27,1/1,1,21945530,rs77539762,C,T,PASS,0.016,60,0.031

1:220603324:G,27,30,1/1,,,,,,,,,

1:236557771:A,28,26,1/1,,,,,,,,,

1:248637115:C,16,188,1/1,,,,,,,,,

1:248652058:A,15,184,0/1,,,,,,,,,

1:248652061:T,10,176,0/1,1,248652061,.,C,T,VQSRTrancheSNP99.80to99.90,0.0387,22.41,0.01

1:248722722:C,21,44,1/1,,,,,,,,,

1:248802469:T,25,28,1/1,1,248802469,.,C,T,PASS,0.6082,29,0.805

1:2488153:G,28,27,1/1,1,2488153,rs4870,A,G,PASS,0.0405,60,0.51

1:25747230:C,7,17,1/1,,,,,,,,,

1:26608843:A,28,64,0/1,,,,,,,,,

1:53681699:G,28,31,1/1,1,53681699,rs1134688,T,G,PASS,0.0333,59.65,0.161

1:53712727:T,28,27,1/1,1,53712727,rs5174,C,T,PASS,0.1006,59.61,0.294

1:86375654:G,28,23,1/1,,,,,,,,,

1:86512536:T,23,18,1/1,,,,,,,,,

1:86557967:A,27,29,1/1,,,,,,,,,

1:92457843:T,20,17,1/1,1,92457843,rs10747493,C,T,PASS,0.0556,58.83,0.811

20:29623215:A,25,31,1/1,,,,,,,,,

20:29623223:C,26,32,1/1,20,29623223,rs10439604,T,C,InbreedingCoeff_Filter,na,na,0.496

20:2996497:T,20,24,1/1,20,2996497,rs1178016,C,T,PASS,0.0358,59.58,0.547

20:30452782:T,26,18,1/1,20,30452782,rs4911536,C,T,PASS,0.0253,59.5,0.312

20:31981849:C,12,21,1/1,20,31981849,rs291700,T,C,PASS,0.0271,59.2,0.647

21:34614250:C,28,18,1/1,21,34614250,rs2229207,T,C,PASS,0.0183,59.61,0.105

21:34614255:G,27,19,1/1,,,,,,,,,

21:40584598:T,22,20,1/1,21,40584598,rs6517529,C,T,PASS,0.0867,59.65,0.897

21:42879909:A,27,32,1/1,21,42879909,rs75603675,C,A,PASS,0.1118,60,0.224

21:43169357:G,27,36,1/1,,,,,,,,,

21:43985955:A,26,21,1/1,21,43985955,rs228104,G,A,PASS,0.1068,59.62,0.852

21:46908355:C,9,17,1/1,,,,,,,,,

21:46924434:C,28,52,0/1,,,,,,,,,

22:18727115:T,24,21,1/1,22,18727115,.,G,T,VQSRTrancheSNP99.60to99.80,0.0153,29,na

22:20456851:T,28,168,1/1,22,20456851,.,C,T,VQSRTrancheSNP99.80to99.90,na,na,na

22:20780031:C,25,161,0/1,,,,,,,,,

22:21570790:G,23,28,1/1,22,21570790,rs4051635,A,G,VQSRTrancheSNP99.60to99.80,na,na,na

22:21570832:G,9,19,1/1,,,,,,,,,

22:23263602:T,24,14,1/1,22,23263602,rs373405,C,T,PASS,0.0138,59.53,0.923

22:24300634:T,21,82,1/1,,,,,,,,,

22:26862212:C,15,14,1/1,22,26862212,rs713998,T,C,PASS,0.0642,59.65,0.849

22:29885594:T,28,144,0/1,,,,,,,,,

22:32205632:C,23,16,1/1,22,32205632,rs5998135,A,C,PASS,0.0208,59.25,0.101

22:37964413:A,C,19,60,1/2,,,,,,,,,

22:37964413:C,A,11,60,1/2,,,,,,,,,

22:37964419:C,25,65,0/1,,,,,,,,,

22:38506509:G,27,21,1/1,22,38506509,rs4820313,A,G,PASS,0.0005,60,1

22:42911257:G,24,57,1/1,22,42911257,rs1137212,A,G,PASS,0.5211,29,0.133

22:44379838:G,28,35,1/1,22,44379838,rs8418,A,G,PASS,0.044,59.6,0.65

2:100343557:T,24,26,1/1,2,100343557,rs4851223,C,T,PASS,0.0001,59.63,1

2:111598958:T,12,18,1/1,,,,,,,,,

2:112536264:T,5,16,1/1,2,112536264,rs76115652,G,T,VQSRTrancheSNP99.90to99.95,0.0876,22,0.999

2:118771566:A,24,28,1/1,2,118771566,rs11545372,C,A,PASS,0.0603,60,0.276

2:120199140:G,26,25,1/1,2,120199140,rs1530100,A,G,PASS,0.0188,59.69,0.863

2:121981950:G,27,25,1/1,2,121981950,rs11890430,A,G,PASS,0.0232,59.7,0.334

2:130832256:G,26,148,0/1,,,,,,,,,

2:131220699:T,15,265,0/1,2,131220699,.,C,T,PASS,na,na,na

2:131377707:G,23,35,1/1,2,131377707,.,A,G,VQSRTrancheSNP99.90to99.95,0.0864,23,0.833

2:132021781:T,21,324,0/1,,,,,,,,,

2:175202208:A,5,13,1/1,,,,,,,,,

2:187559047:A,28,173,0/1,,,,,,,,,

2:187559050:G,26,171,0/1,,,,,,,,,

2:188250301:A,28,27,1/1,,,,,,,,,

2:191184475:G,21,27,1/1,2,191184475,rs291466,A,G,PASS,0.0855,59.87,0.519

2:207603234:G,12,17,1/1,2,207603234,rs2287631,T,G,PASS,0.1433,59.67,0.266

2:228111435:C,25,30,1/1,2,228111435,rs10178458,T,C,PASS,0.0428,59.63,0.83

2:228194480:T,9,22,1/1,,,,,,,,,

2:228194481:T,11,21,1/1,,,,,,,,,

2:233712227:G,17,26,1/1,,,,,,,,,

2:240946766:C,26,22,1/1,2,240946766,rs13848,T,C,PASS,0.0274,60,0.392

2:24390517:A,19,23,1/1,2,24390517,rs2288072,G,A,PASS,0.0695,59.57,0.598

2:26804247:C,10,21,1/1,2,26804247,rs935172,T,C,PASS,0.0307,59.52,0.547

2:70129800:T,28,59,0/1,,,,,,,,,

2:70677994:A,1,15,1/1,2,70677994,rs2166975,G,A,PASS,0.0242,60,0.238

2:71170807:T,28,33,1/1,2,71170807,rs2266918,C,T,PASS,0.0679,59.53,0.24

2:85549868:G,28,26,1/1,2,85549868,rs4240199,A,G,PASS,0.0635,59.59,0.676

2:87088964:G,21,21,1/1,2,87088964,rs62146888,A,G,PASS,0.3243,49.66,0.205

2:90260243:G,12,38,1/1,,,,,,,,,

2:96780986:T,25,178,0/1,,,,,,,,,

3:113222036:A,26,30,1/1,3,113222036,rs11537650,G,A,PASS,0.0306,59.45,0.22

3:11596302:C,8,22,1/1,3,11596302,rs2305295,T,C,PASS,0.0255,59.65,0.279

3:122103120:A,13,16,1/1,3,122103120,rs115407410,G,A,PASS,na,na,0.021

3:14105893:C,26,65,0/1,,,,,,,,,

3:14105894:A,26,64,0/1,,,,,,,,,

3:14105897:A,G,20,60,1/2,,,,,,,,,

3:14105897:G,A,10,60,1/2,,,,,,,,,

3:14106033:C,24,24,1/1,,,,,,,,,

3:14106037:C,27,23,1/1,,,,,,,,,

3:14106354:T,16,159,0/1,3,14106354,.,C,T,PASS,na,na,na

3:46487937:T,28,30,1/1,,,,,,,,,

3:4767262:C,16,27,1/1,3,4767262,rs7613447,T,C,PASS,0.1278,59.58,0.687

3:57261947:C,27,26,1/1,3,57261947,rs79282761,G,C,PASS,na,na,na

3:58625875:A,24,19,1/1,,,,,,,,,

3:59997112:A,8,11,1/1,3,59997112,rs17255497,G,A,PASS,0.0308,59.45,0.128

3:9920138:C,26,14,1/1,3,9920138,rs17851444,G,C,PASS,0.0219,59.61,0.113

4:100045616:T,7,24,1/1,4,100045616,rs1126673,C,T,PASS,0.0649,60,0.749

4:108641300:C,27,27,1/1,4,108641300,rs973126,T,C,PASS,0.0179,58.66,0.898

4:109841743:T,11,21,1/1,4,109841743,rs17596705,C,T,PASS,0.0429,59.65,0.169

4:110678925:C,13,14,1/1,4,110678925,rs11098044,T,C,PASS,0.1451,49.08,0.989

4:144920596:A,21,27,1/1,4,144920596,rs7683365,G,A,PASS,0.0468,56.04,0.302

4:145041686:G,16,14,1/1,4,145041686,rs4867,A,G,PASS,0.6359,29,0.941

4:145041720:G,12,24,1/1,4,145041720,rs7682260,A,G,PASS,0.576,29,0.162

4:155295076:G,17,16,1/1,4,155295076,rs11721758,A,G,PASS,0.0323,57.85,0.406

4:156787340:A,27,27,1/1,4,156787340,rs6848883,G,A,PASS,0.1093,59.64,0.916

4:157557726:G,18,16,1/1,4,157557726,rs11943855,A,G,PASS,0.1303,59.66,0.51

4:185655192:C,23,27,1/1,,,,,,,,,

4:26673787:G,18,18,1/1,,,,,,,,,

4:41673604:C,20,10,1/1,4,41673604,rs11734372,T,C,PASS,0.0586,59.11,0.504

4:48988450:A,27,23,1/1,4,48988450,rs3747690,C,A,PASS,0.2095,60,0.28

4:68995529:T,2,12,1/1,4,68995529,rs10030708,C,T,PASS,0.0225,59.62,0.298

4:7765495:T,8,13,1/1,,,,,,,,,

4:78987157:G,10,19,1/1,,,,,,,,,

4:80905990:G,12,15,1/1,,,,,,,,,

4:80905991:T,18,15,1/1,,,,,,,,,

4:81529518:C,27,23,1/1,4,81529518,rs2867782,T,C,PASS,0.1264,59.77,0.951

4:88535830:C,23,131,0/1,4,88535830,.,T,C,VQSRTrancheSNP99.80to99.90,na,na,na

4:88535831:A,23,130,0/1,4,88535831,.,G,A,VQSRTrancheSNP99.80to99.90,na,na,na

4:88535832:G,23,129,0/1,,,,,,,,,

4:88536886:T,15,117,0/1,,,,,,,,,

4:9250440:G,12,96,1/1,4,9250440,.,T,G,AC_Adj0_Filter,na,na,na

5:112312676:C,27,30,1/1,5,112312676,rs33555,T,C,PASS,0,59.62,1

5:135178124:C,23,17,1/1,5,135178124,rs2304075,T,C,PASS,0.048,59.55,0.771

5:13944512:C,28,20,1/1,5,13944512,rs339445,A,C,PASS,0.0263,59.66,0.936

5:140558314:C,25,35,1/1,,,,,,,,,

5:140558315:C,24,34,1/1,,,,,,,,,

5:140558317:G,27,38,1/1,,,,,,,,,

5:146619206:C,28,32,1/1,,,,,,,,,

5:156479568:T,28,47,0/1,,,,,,,,,

5:167913510:A,25,32,1/1,,,,,,,,,

5:177161891:G,25,27,1/1,,,,,,,,,

5:179070463:G,3,54,0/1,,,,,,,,,

5:179070464:G,13,54,0/1,,,,,,,,,

5:179070469:A,14,54,0/1,,,,,,,,,

5:53606295:C,25,28,1/1,5,53606295,rs35941,T,C,PASS,0.047,60,0.815

5:57854018:T,7,21,1/1,5,57854018,rs13181639,A,T,PASS,0.0169,50.02,0.041

5:668499:G,10,17,1/1,,,,,,,,,

5:668500:C,11,17,1/1,,,,,,,,,

5:72894207:A,7,17,1/1,5,72894207,rs3108889,G,A,PASS,0.542,60,0.79

5:73090261:C,28,35,1/1,5,73090261,rs7716253,T,C,PASS,0.0457,58.7,0.546

5:73148481:A,27,21,1/1,5,73148481,rs2973566,G,A,PASS,0.0318,59.63,0.241

5:741736:T,13,16,1/1,5,741736,rs61128505,G,T,PASS,0.1355,22,0.82

5:833915:T,26,27,1/1,5,833915,rs605088,G,T,VQSRTrancheSNP99.80to99.90,na,na,na

5:837556:G,25,32,1/1,5,837556,rs28449575,T,G,InbreedingCoeff_Filter,na,na,0.363

6:132029857:T,19,84,1/1,6,132029857,.,C,T,VQSRTrancheSNP99.80to99.90,0.0166,22,0.029

6:132029865:A,18,88,1/1,,,,,,,,,

6:150343148:C,28,45,1/1,,,,,,,,,

6:166739646:C,12,25,1/1,6,166739646,rs11551053,T,C,PASS,0.0823,59.61,0.077

6:27115129:G,26,154,0/1,,,,,,,,,

6:31084945:G,3,11,0/1,,,,,,,,,

6:32299822:A,4,14,1/1,6,32299822,rs115199069,G,A,PASS,0.0252,59.62,0.202

6:32497917:A,1,13,1/1,6,32497917,rs74766936,C,A,VQSRTrancheSNP99.60to99.80,na,na,0.077

6:32552075:G,28,25,1/1,6,32552075,rs1064664,A,G,PASS,0.1427,39.45,0.287

6:32552078:T,27,24,1/1,,,,,,,,,

6:32552080:C,27,24,1/1,,,,,,,,,

6:32552085:T,26,24,1/1,,,,,,,,,

6:32552091:C,26,24,1/1,,,,,,,,,

6:32552092:T,26,24,1/1,6,32552092,rs16822516,A,T,PASS,na,na,0.252

6:32609192:G,14,18,1/1,6,32609192,rs1142323,A,G,PASS,0.2334,46.31,0.264

6:32609312:C,17,22,1/1,,,,,,,,,

6:32628022:G,26,22,1/1,,,,,,,,,

6:32634300:C,22,22,1/1,,,,,,,,,

6:32634302:G,22,22,1/1,,,,,,,,,

6:32634303:A,22,22,1/1,,,,,,,,,

6:32634306:T,23,23,1/1,6,32634306,rs1049060,A,T,PASS,0.2431,41.23,0.532

6:32634313:G,25,26,1/1,6,32634313,rs1049059,C,G,PASS,0.1941,47.51,0.194

6:32634318:A,25,26,1/1,6,32634318,rs3891176,C,A,PASS,0.19,38.61,0.109

6:32725062:A,5,17,1/1,6,32725062,rs3213484,T,A,PASS,0.1045,59.29,0.611

6:350829:A,27,34,1/1,6,350829,rs1129085,G,A,InbreedingCoeff_Filter,na,na,0.389

6:36733132:A,14,16,1/1,6,36733132,rs763048,G,A,PASS,0.0317,59.42,0.611

6:39048491:C,21,14,1/1,6,39048491,rs1126476,A,C,PASS,0.0086,59.54,0.499

6:42123317:C,28,28,1/1,6,42123317,rs2207730,A,C,PASS,0.1578,59.73,0.654

6:54054686:A,22,23,1/1,6,54054686,rs6934690,T,A,PASS,0.0796,59.59,0.879

6:57467100:C,2,16,1/1,,,,,,,,,

6:74466377:T,17,18,1/1,6,74466377,rs6453696,C,T,PASS,0.056,59.56,0.588

6:7563983:G,28,22,1/1,,,,,,,,,

7:100641745:A,20,187,0/1,7,100641745,.,G,A,VQSRTrancheSNP99.60to99.80,na,na,0.012

7:100642986:A,28,150,0/1,7,100642986,rs149850933,G,A,VQSRTrancheSNP99.80to99.90,na,na,na

7:100643575:T,19,128,0/1,,,,,,,,,

7:100644027:A,27,150,1/1,7,100644027,rs140825627,C,A,VQSRTrancheSNP99.80to99.90,na,na,0.017

7:101194424:T,23,21,1/1,7,101194424,rs17135626,C,T,PASS,0.14,60,0.234

7:101837149:A,24,22,1/1,7,101837149,rs11540899,G,A,PASS,0.0352,59.42,0.352

7:102294074:C,28,39,1/1,,,,,,,,,

7:104110492:T,1,10,1/1,7,104110492,rs6949536,C,T,PASS,0.1417,59.61,0.251

7:123672479:T,19,172,0/1,,,,,,,,,

7:128587381:C,28,24,1/1,,,,,,,,,

7:134225827:G,16,19,1/1,7,134225827,rs4728329,A,G,PASS,0.0305,59.54,0.938

7:134264286:T,16,19,1/1,7,134264286,rs6467538,C,T,PASS,0.0303,59.62,0.33

7:137128830:C,4,18,1/1,7,137128830,rs1918837,T,C,PASS,0.0619,60,0.407

7:148936878:T,6,19,1/1,7,148936878,rs71532769,G,T,PASS,0.028,58.78,0.085

7:154429560:T,25,16,1/1,7,154429560,rs11243339,C,T,PASS,0.1237,60,0.683

7:32529936:C,26,29,1/1,7,32529936,rs1584614,G,C,PASS,0.0365,59.61,0.786

7:43810764:A,1,11,1/1,7,43810764,rs699512,G,A,PASS,0.0308,59.58,0.801

7:6013049:G,20,71,1/1,,,,,,,,,

7:6798735:A,1,36,0/1,7,6798735,.,G,A,PASS,na,na,na

8:10467605:T,12,133,0/1,,,,,,,,,

8:10467652:C,23,121,0/1,,,,,,,,,

8:10530218:T,24,21,1/1,8,10530218,rs11250058,C,T,PASS,0.0494,60,0.996

8:119964052:C,10,22,1/1,,,,,,,,,

8:139642974:C,25,30,1/1,8,139642974,rs4131277,T,C,PASS,0.1102,59.63,0.168

8:17270787:G,5,19,1/1,8,17270787,rs67972978,A,G,PASS,0.0318,59.52,0.992

8:22526559:A,2,6,1/1,8,22526559,rs17088526,G,A,PASS,0.062,60,0.176

8:22584718:C,25,21,1/1,8,22584718,rs1129474,T,C,PASS,0.038,59.63,0.468

8:27396208:A,21,18,1/1,8,27396208,rs4149253,G,A,PASS,0.0258,59.63,0.078

8:6500544:T,26,21,1/1,8,6500544,rs1057091,C,T,PASS,0.0193,59.63,0.292

8:69143589:G,20,14,1/1,8,69143589,rs2280637,A,G,PASS,0.0436,59.11,0.319

8:7673126:A,26,32,1/1,8,7673126,.,C,A,InbreedingCoeff_Filter,na,na,0.306

8:87666251:G,28,31,1/1,,,,,,,,,

9:117033022:C,28,25,1/1,9,117033022,rs4143245,T,C,PASS,0.0313,59.63,0.416

9:130698029:C,28,30,1/1,9,130698029,rs7997,G,C,PASS,0.0824,59.54,0.787

9:132630668:C,5,157,1/1,,,,,,,,,

9:134401335:A,23,30,1/1,,,,,,,,,

9:135105964:G,8,16,1/1,9,135105964,rs4962173,A,G,PASS,0.04,59.27,0.993

9:138836946:A,3,107,0/1,,,,,,,,,

9:140262426:T,28,30,1/1,9,140262426,rs11533158,C,T,PASS,0.05,59.57,0.694

9:19622268:C,17,19,1/1,9,19622268,rs4977308,T,C,PASS,0.0238,59.47,0.76

9:42410368:C,5,148,1/1,9,42410368,.,T,C,AC_Adj0_Filter,0.592,29.61,0.308

9:43876040:A,22,35,1/1,,,,,,,,,

9:69238268:A,28,34,1/1,9,69238268,.,G,A,InbreedingCoeff_Filter,na,na,0.505

9:69247550:A,6,16,1/1,,,,,,,,,

9:69256816:A,28,56,1/1,9,69256816,rs62557779,G,A,PASS,0.1433,30.57,0.517

9:69653122:T,26,21,1/1,9,69653122,.,C,T,VQSRTrancheSNP99.80to99.90,0.0282,22,0.016

9:8497250:C,7,14,1/1,9,8497250,rs72694737,T,C,PASS,0.0075,60,0.022

X:2632482:T,14,18,1/1,,,,,,,,,

X:2724760:C,23,26,1/1,X,2724760,rs111382948,T,C,PASS,0.4496,58.52,0.974

X:27765405:A,6,75,0/1,,,,,,,,,

X:8433801:G,27,231,0/1,X,8433801,.,A,G,VQSRTrancheSNP99.60to99.80,na,na,na
