# Supplementary material for: Recurrent miscalling of missense variation from short-read genome sequence data
Source: BMC Genomics. 2019 Jul 16;20(Suppl 8):546. doi: 10.1186/s12864-019-5863-2 (PMC6631443; doi:10.1186/s12864-019-5863-2)
Supplement: Supplementary file 2 — Table S2. Near-paralogs of example human recurrent false positive variants. (DOCX 23 kb) [file 12864_2019_5863_MOESM2_ESM.docx]

**Additional file 2: Table S2 –** Near-paralogs of example human recurrent false positive variants.

| Recurrent False Positive Coordinate | Region Coordinates | Gene | Match Gene | Match Coords | Match length (bp) | Match identity (%) | Search type |
| --- | --- | --- | --- | --- | --- | --- | --- |
|  |  |  |  |  |  |  |  |
| 14:74010331:G | 14:74009832-74010414 | ACOT1 | ACOT2 | 14:74041690-74042272 | 583 | 99.83 | single exon |
| 19:40385103:G | 19:40384562-40385135 | FCGBP | FCGBP | 19:40368303-40368876 | 574 | 98.08 | single exon |
| 1:144811827:A | 1:144811744-144811957 | NBPF9 | NBPF10 | HG1287_PATCH:149762070-149762283 | 408 | 99.07 | single exon |
| 1:148754858:C | 1:148579606-148579708 | NBFP15 | NBFP16 | 1:144162001-144162173 | 173 | 99.4 | single exon |
| X:2724742 | X:2724742-2724786 | XG | XGPY | Y:14556556-14556598 | 43 | 95.35 | single exon |
| 9:67968720:C | 9:67987826-67987998 | RP11-195B21.3 | CR848007.2 | 9:44057213-44057385 | 173 | 98.84 | single exon |
| 7:72436652:G | 7:72436290-72436687 | TRIM74 | TRIM73 | 7:75028219-75028616 | 398 | 99.5 | single exon |
| 2:231258150:T | 2:231191899-231268447 | SP140L | SP140 | 2:231134614-231176310 | 1116 | 95.79 | Whole cDNA |
| 2:100343557:T | 2:100162323-100721178 | AFF3 | AFF3 | 2:100623088-100623924 | 707 | 99.86 | Whole cDNA |
| 2:187559053:G | 22:18721427-18745407 | AC008132.1 | FAM230A | 22:20692509-20706405 | 474 | 97.87 | Whole cDNA |
| 2:228194481:T | 22:22890123-22901747 | PRAME | RP11-219C24.6 | 1:13411818-13412260 | 47 | 97.87 | Whole cDNA |
| 3:195501149:T | 3:195473636-195539148 | MUC4 | MUC4 | 3:195508560-195515533 | 7133 | 87.9 | Whole cDNA |
