# Supplementary material for: Recurrent miscalling of missense variation from short-read genome sequence data
Source: BMC Genomics. 2019 Jul 16;20(Suppl 8):546. doi: 10.1186/s12864-019-5863-2 (PMC6631443; doi:10.1186/s12864-019-5863-2)
Supplement: Supplementary file 3 — Table S3. Genes that contain one or more RFP variant. (DOCX 23 kb) [file 12864_2019_5863_MOESM3_ESM.docx]

**Additional file 3: Table S3** - Genes that contain one or more RFP variant (comma-separated values).

**Gene,RFP Count**

HLA-DRB1,59

HLA-B,56

HLA-DRB5,39

HLA-DQB1,36

DSPP,33

MUC4,32

MAGEC1,24

HLA-A,24

LILRB3,19

HLA-DQA1,19

VCX3B,17

PRAMEF1,17

ZXDB,16

VCX3A,16

FCGBP,16

MUC12,15

PRR21,14

OR6B3,14

ZXDA,13

PLIN4,13

OPN1LW,13

MAPT,13

LILRA6,13

RPGR,11

MUC5B,11

ESX1,11

TPSAB1,10

SPPL2C,10

PDE4DIP,10

CT45A5,10

UBXN11,9

PASD1,9

OR8U1,9

MAGEA6,9

GOLGA6L6,9

SH3BGRL3,8

RFPL4A,8

OPN1MW,8

NBPF16,8

MAGEA3,8

HRNR,8

FAM9A,8

CEP85,8

ZNF93,7

VARS2,7

TAS2R31,7

PRAMEF22,7

PRAMEF10,7

PCDHA4,7

PCDHA2,7

KCNJ12,7

FAM47C,7

C4A,7

start_lost,6

PSORS1C1,6

OR12D2,6

MUC21,6

MUC20,6

FLG2,6

FAM47A,6

FAM186A,6

CDSN,6

CDHR2,6

C6orf15,6

TAF7L,5

RP1L1,5

PRAMEF7,5

PRAMEF11,5

POTEF,5

POM121,5

OR51A2,5

OR2T8,5

OR2T29,5

OR10G2,5

NUDT10,5

NOTCH2,5

MUC22,5

KRTAP5-5,5

KRTAP5-10,5

KRTAP4-7,5

KANSL1,5

HLA-C,5

GOLGA8R,5

GOLGA8K,5

GOLGA8A,5

FAM86B2,5

DMBT1,5

CRIPAK,5

ZNF233,4

ZDHHC11,4

VCX2,4

TCP10,4

TCEANC,4

TBC1D3,4

TAS2R46,4

SPANXC,4

SELPLG,4

RSPH10B,4

PRR25,4

PRAMEF13,4

PPP2R3B,4

PPIAL4G,4

POTEI,4

POTEE,4

PER3,4

PCDHB8,4

PCDHB16,4

OR4M2,4

OR2T35,4

OR2T34,4

NBPF3,4

MUC17,4

MTRNR2L2,4

MSH3,4

KRTAP5-8,4

KRTAP4-5,4

KRTAP1-1,4

KRTAP10-10,4

KLF14,4

KIR2DL4,4

IGFN1,4

GYPA,4

FAM86B1,4

DRD4,4

DHFR,4

DCAF8L2,4

CXorf40B,4

CXorf40A,4

COL24A1,4

CLIC6,4

CCHCR1,4

CCDC177,4

C6orf10,4

AMOT,4

ZFPM1,3

VRK3,3

USP17L18,3

USP17L17,3

TRPM4,3

TRDN,3

TPSG1,3

TPSD1,3

TPRX1,3

TNXB,3

TMPRSS13,3

TAS2R19,3

SYCP2L,3

SSX4B,3

SPANXD,3

SOX3,3

SLC25A26,3

SKIV2L,3

RGPD4,3

RFPL4AL1,3

QRICH2,3

PTPN18,3

PSG8,3

PSG4,3

PPIAL4A,3

PPFIA3,3

POTEG,3

POTEC,3

POTEB2,3

POLR2J3,3

PLEKHM1,3

PCDHB7,3

PAGE2,3

OR9G1,3

OR5P3,3

OR2T4,3

OR2T3,3

ODF1,3

NUTM2A,3

NELFE,3

N6AMT1,3

MPHOSPH6,3

MFF,3

MAML3,3

MADCAM1,3

LRCH2,3

LILRB2,3

LCE1F,3

KRTAP9-2,3

KRTAP10-2,3

KIR3DL3,3

KIR3DL1,3

KIR2DL3,3

HTT,3

HRC,3

GTF2IRD2,3

GSTT2B,3

GSE1,3

GPRIN1,3

GOLGA6A,3

GGT1,3

GAGE12J,3

FRMPD2,3

FOXP3,3

FMN2,3

FAM174B,3

FAM171B,3

FAM153A,3

EMR2,3

DEAF1,3

CYP21A2,3

CR1,3

COL25A1,3

COL22A1,3

COL13A1,3

CGB7,3

CFB,3

CENPU,3

CD99,3

CBWD6,3

CACNA1F,3

BRD2,3

APOB,3

ANP32E,3

ANKRD36,3

ANKRD20A1,3

AGAP6,3

AGAP4,3

ADAM29,3

ABCC6,3

ZNF812,2

ZNF512,2

ZNF408,2

ZNF284,2

ZNF219,2

ZNF205,2

ZIC3,2

ZFHX3,2

YIF1B,2

XKR3,2

WDR93,2

WDR87,2

WDR17,2

WBP5,2

VSIG10,2

VARS,2

USP17L19,2

ULK2,2

UCK1,2

TRPM1,2

TRIM49,2

TRIM31,2

TPTE,2

TMEM99,2

TMEM52,2

TMEM247,2

TMC4,2

TIMM23,2

TIMM17B,2

THSD7A,2

TGOLN2,2

TCP10L2,2

TCF19,2

TCERG1,2

TCEAL4,2

TBP,2

TAP2,2

TAF1,2

SYT15,2

STS,2

STH,2

SPRR3,2

SPIN2A,2

SPATA7,2

SPATA4,2

SPATA31A3,2

SLC5A3,2

SLC45A3,2

SLC44A4,2

SLC37A1,2

SLC35A2,2

SLC25A48,2

SLC25A11,2

SIRPA,2

SH2D1B,2

SFT2D1,2

SERPINA1,2

SALL3,2

RSPH10B2,2

RPUSD1,2

RPS6KA6,2

ROBO4,2

ROBO3,2

RIMBP3B,2

RIMBP3,2

RGPD8,2

REXO1,2

RASGRP1,2

RAP1GAP,2

RAB40AL,2

PWP2,2

PTGES3L,2

PRUNE2,2

PRB4,2

PRAMEF5,2

PRAM1,2

PQBP1,2

POTEJ,2

POTEB,2

POMT1,2

POM121C,2

PNPLA3,2

PLA2G5,2

PHF2,2

PHF20L1,2

PGPEP1L,2

PGLYRP4,2

PGA3,2

PCDHB10,2

PAPSS1,2

PAGE2B,2

OTOP1,2

OR5M10,2

OR5H6,2

OR4N4,2

OR4D11,2

OR2T5,2

OR2T27,2

OR13C2,2

OR12D3,2

OPN4,2

OPN1MW2,2

OC90,2

NTM,2

NPVF,2

NPIPB6,2

NOTCH2NL,2

NCF1,2

MYLK,2

MUC6,2

MIPEP,2

MESP2,2

MED16,2

MDH1B,2

MBD3L3,2

MBD3L2,2

MAP7D3,2

MAP1A,2

MAMLD1,2

MAML2,2

MAGED2,2

MAGEB6,2

LYPD2,2

LTBP3,2

LSM2,2

LRRC37A3,2

LRRC37A2,2

LRRC37A,2

LGR4,2

LGALS2,2

LBP,2

LAMA4,2

LAIR2,2

LAIR1,2

KSR1,2

KRTAP9-8,2

KRT18,2

KRT10,2

ITIH6,2

ITGA8,2

IRS1,2

IRF2BPL,2

IL27,2

IFNAR2,2

IFITM3,2

HTN3,2

HPS4,2

HERC2,2

HDLBP,2

HDGFRP2,2

HCLS1,2

GTF2H4,2

GSTM4,2

GREB1L,2

GPKOW,2

GP1BA,2

GOLGA8B,2

GIGYF2,2

GALNT6,2

GAGE13,2

FXYD5,2

FOXO4,2

FNBP4,2

FBXW10,2

FANCB,2

FAM98B,2

FAM25A,2

FAM21A,2

FAM200B,2

FAM133A,2

FADS6,2

ELMO3,2

EHMT2,2

E2F4,2

DTHD1,2

DENND4B,2

DCAF8,2

DAPL1,2

CXorf65,2

CXorf27,2

CUX1,2

CTAGE9,2

CTAGE15,2

CT45A2,2

CPAMD8,2

CORO7-PAM16,2

COL6A6,2

COL4A3,2

COL27A1,2

COL18A1,2

COG3,2

CNTNAP3B,2

CLN3,2

CGB,2

CFD,2

CEP170B,2

CELA1,2

CDRT1,2

CDC42EP1,2

CD276,2

CD109,2

CCZ1,2

CCDC34,2

CCDC155,2

CBX8,2

CBWD1,2

CASZ1,2

CALML6,2

C5orf60,2

C4B,2

C2orf16,2

C1QTNF9B,2

C19orf33,2

BTNL9,2

BPIFB1,2

B3GNTL1,2

ATAD5,2

ATAD3B,2

ASIC5,2

ARSD,2

ARHGEF40,2

ARHGEF28,2

ARHGAP1,2

AQP12B,2

APOBR,2

ANTXR2,2

ANO7,2

ANAPC1,2

AGAP8,2

AGAP7,2

ACACA,2

AARSD1,2

AADACL3,2
