# Supplementary material for: Recurrent miscalling of missense variation from short-read genome sequence data
Source: BMC Genomics. 2019 Jul 16;20(Suppl 8):546. doi: 10.1186/s12864-019-5863-2 (PMC6631443; doi:10.1186/s12864-019-5863-2)
Supplement: Supplementary file 4 — Table S4. Genome sequence metadata. (DOCX 22 kb) [file 12864_2019_5863_MOESM4_ESM.docx]

**Additional file 4: Table S4**– Genome sequence metadata.

| **Cohort** | **Individual** | **Ethnicity** | **Genome_Exome** | **ReadLength (bp)** | **Facility** |
| --- | --- | --- | --- | --- | --- |
| **1000 Genomes** | NA19332 | LWK | exome | 75 | 1000 Genomes |
|  | HG03303 | ESN | exome | 75 | 1000 Genomes |
|  | NA18954 | JPT | exome | 75 | 1000 Genomes |
|  | HG03473 | MSL | exome | 75 | 1000 Genomes |
|  | HG00272 | FIN | exome | 75 | 1000 Genomes |
|  | HG00614 | CHS | exome | 75 | 1000 Genomes |
|  | NA20853 | GIH | exome | 75 | 1000 Genomes |
|  | HG01939 | PEL | exome | 75 | 1000 Genomes |
|  | HG03714 | ITU | exome | 75 | 1000 Genomes |
|  | HG03916 | BEB | exome | 75 | 1000 Genomes |
|  | NA12878 | CEU | exome | 75 | 1000 Genomes |
|  | HG02019 | KHV | exome | 75 | 1000 Genomes |
|  | HG03884 | STU | exome | 75 | 1000 Genomes |
|  | NA18555 | CHB | exome | 90 | 1000 Genomes |
|  | HG02165 | CDX | exome | 100 | 1000 Genomes |
|  |  |  |  |  |  |
| **Indigenous Australian** | Tiwi9 | Indigenous Australian | whole genome | 150 | Kinghorn Centre Clinical Genomics |
|  | Tiwi19 | Indigenous Australian | whole genome | 150 | Kinghorn Centre Clinical Genomics |
|  | Tiwi29 | Indigenous Australian | whole genome | 150 | Kinghorn Centre Clinical Genomics |
|  | Tiwi39 | Indigenous Australian | whole genome | 150 | Kinghorn Centre Clinical Genomics |
|  | Tiwi49 | Indigenous Australian | whole genome | 150 | Kinghorn Centre Clinical Genomics |
|  | Tiwi59 | Indigenous Australian | whole genome | 150 | Kinghorn Centre Clinical Genomics |
|  | Tiwi69 | Indigenous Australian | whole genome | 150 | Kinghorn Centre Clinical Genomics |
|  | Tiwi79 | Indigenous Australian | whole genome | 150 | Kinghorn Centre Clinical Genomics |
|  | Tiwi89 | Indigenous Australian | whole genome | 150 | Kinghorn Centre Clinical Genomics |
|  | Tiwi99 | Indigenous Australian | whole genome | 150 | Kinghorn Centre Clinical Genomics |
|  |  |  |  |  |  |
| **Omani** | Oman1 | Omani | whole genome | 150 | Macrogen |
|  | Oman2 | Omani | whole genome | 150 | Macrogen |
|  | Oman3 | Omani | whole genome | 150 | Macrogen |
|  | Oman4 | Omani | whole genome | 150 | Macrogen |
|  | Oman5 | Omani | whole genome | 150 | Macrogen |
|  | Oman6 | Omani | whole genome | 150 | Macrogen |
|  | Oman7 | Omani | whole genome | 150 | Macrogen |
|  | Oman8 | Omani | whole genome | 150 | Macrogen |
|  | Oman9 | Omani | whole genome | 150 | Macrogen |
|  | Oman10 | Omani | whole genome | 150 | Macrogen |
|  |  |  |  |  |  |
| **GenomeInABottle** | NA12878 | CEU | whole genome | 150 | Illumina Platinum genome |
