# Supplementary material for: Recurrent miscalling of missense variation from short-read genome sequence data
Source: BMC Genomics. 2019 Jul 16;20(Suppl 8):546. doi: 10.1186/s12864-019-5863-2 (PMC6631443; doi:10.1186/s12864-019-5863-2)
Supplement: Supplementary file 5 — Table S5. A catalogue of all recurrent false positive variants identified among 1000 Genome, Omani and Indigenous Australian cohorts. (DOCX 263 kb) [file 12864_2019_5863_MOESM5_ESM.docx]

**Additional file 5: Table S5** – A catalogue of all recurrent false positive variants identified among 1000 Genome, Omani and Indigenous Australian cohorts.

| **RFP (GRCh37)** | **Multi-cohort RFP count** | **DP** | **MQ** |
| --- | --- | --- | --- |
| 10:104622661:C | 1 | 24 | 49 |
| 10:116008497:A | 3 | 10 | 50 |
| 10:118368606:C | 1 | 24 | 49 |
| 10:119774598:A | 1 | 25 | 50 |
| 10:120919246:C | 11 | 15 | 49 |
| 10:122216862:T | 1 | 25 | 49 |
| 10:124329710:A | 8 | 19 | 49 |
| 10:124330427:T | 9 | 27 | 49 |
| 10:124331867:T | 6 | 16 | 49 |
| 10:124340406:C | 1 | 11 | 46 |
| 10:124356566:G | 3 | 12 | 20 |
| 10:124768583:A | 4 | 37 | 50 |
| 10:13166076:G | 9 | 6 | 50 |
| 10:13699153:A | 4 | 192 | 45 |
| 10:15719604:G | 1 | 20 | 49 |
| 10:15719621:G | 1 | 18 | 49 |
| 10:17869577:T | 5 | 137.5 |  |
| 10:18138656:T | 1 | 4.5 |  |
| 10:27436463:T | 4 | 5 |  |
| 10:3214942:C | 1 | 24 | 49 |
| 10:37488689:C | 2 | 35 | 49 |
| 10:43650737:G | 1 | 23 | 50 |
| 10:44878713:A | 1 | 17 | 50 |
| 10:46248649:C | 3 | 6.5 |  |
| 10:46321555:A | 1 | 137 | 7 |
| 10:46321555:C | 1 | 137 | 7 |
| 10:46322030:G | 1 | 291 | 24 |
| 10:46965727:A | 3 | 82 | 49 |
| 10:46967672:T | 6 | 103 | 50 |
| 10:46999601:A | 6 | 146 | 47 |
| 10:49382926:T | 7 | 19 | 26 |
| 10:49386184:T | 1 | 18 | 12 |
| 10:49388901:T | 1 | 76 | 1 |
| 10:51225872:T | 1 | 205 | 9 |
| 10:51226118:A | 1 | 77 | 2 |
| 10:51371565:G | 2 | 112 | 0 |
| 10:51371581:T | 2 | 89 | 0 |
| 10:51464617:T | 3 | 96 | 4 |
| 10:51464656:C | 7 | 118 |  |
| 10:51568378:G | 6 | 16 | 50 |
| 10:51620361:C | 1 | 10 | 11 |
| 10:51623174:A | 2 | 99 | 2 |
| 10:51748683:A | 1 | 200.5 |  |
| 10:51748684:A | 1 | 205.5 |  |
| 10:51768676:G | 3 | 11 | 35 |
| 10:51827896:T | 1 | 12 | 10 |
| 10:51859758:G | 1 | 20 | 10 |
| 10:5260682:G | 1 | 25 | 49 |
| 10:55581885:C | 1 | 12.5 |  |
| 10:58120988:A | 1 | 18 | 49 |
| 10:63976918:T | 5 | 4 |  |
| 10:70741323:C | 1 | 26 | 49 |
| 10:71647223:C | 2 | 20 | 49 |
| 10:71657272:G | 2 | 28 | 50 |
| 10:71678058:G | 4 | 19 | 49 |
| 10:75000739:A | 3 | 8 |  |
| 10:7749183:T | 1 | 16 | 50 |
| 10:81270319:A | 7 | 24 | 43 |
| 10:81465798:T | 3 | 123 |  |
| 10:81606700:T | 1 | 80 | 3 |
| 10:81608302:C | 1 | 11.5 |  |
| 10:81608501:A | 1 | 160 | 6 |
| 10:81608550:A | 1 | 123 |  |
| 10:81608553:G | 1 | 121.5 |  |
| 10:82122737:T | 1 | 9.5 |  |
| 10:88416963:A | 1 | 23 | 50 |
| 10:88416964:G | 1 | 22 | 49 |
| 10:88782100:C | 1 | 19 | 47 |
| 10:88782111:T | 1 | 30 | 46 |
| 10:88992622:T | 1 | 61 | 5 |
| 10:88992705:T | 2 | 140 |  |
| 10:88994452:A | 7 | 17 | 5 |
| 10:89120396:C | 2 | 105 |  |
| 10:89124819:C | 1 | 22 | 8 |
| 10:91487649:T | 4 | 21 | 49 |
| 10:93841227:G | 9 | 19 | 50 |
| 10:95389041:A | 5 | 27 | 49 |
| 10:97106165:C | 2 | 30 | 49 |
| 10:97744383:C | 7 | 24 | 49 |
| 10:97990583:G | 6 | 24 | 50 |
| 10:98115019:A | 1 | 17 | 49 |
| 11:1017981:A | 3 | 139 | 42 |
| 11:1018374:A | 7 | 171 | 49 |
| 11:103152919:A | 2 | 33 | 49 |
| 11:10602082:T | 1 | 9 |  |
| 11:108183167:G | 10 | 14 | 49 |
| 11:1092800:A | 2 | 3.5 |  |
| 11:1092800:C | 11 | 8 |  |
| 11:1092800:C,G | 1 | 6 |  |
| 11:1092800:G | 9 | 3.5 |  |
| 11:1092800:G,C | 1 | 4 |  |
| 11:1092802:C | 11 | 131.5 |  |
| 11:1092802:G | 8 | 74.5 |  |
| 11:1092802:G,C | 1 | 74 |  |
| 11:1093342:A,T | 1 | 35 | 18 |
| 11:1093342:T | 1 | 45 | 19 |
| 11:1093710:G | 6 | 108 | 50 |
| 11:111795085:G | 3 | 19 | 50 |
| 11:112123095:G | 2 | 27 | 49 |
| 11:117078702:C | 1 | 32 | 5 |
| 11:117772955:C | 2 | 18 | 49 |
| 11:117781435:G | 11 | 19 | 50 |
| 11:117789327:C | 10 | 122 | 47 |
| 11:118178007:T | 4 | 10 | 50 |
| 11:118430519:T | 3 | 23 | 49 |
| 11:120107411:A | 8 | 12 | 50 |
| 11:121403229:C | 10 | 22 | 49 |
| 11:122646967:A | 3 | 31 | 49 |
| 11:122774946:A | 1 | 222 | 50 |
| 11:123306172:C | 1 | 14 | 49 |
| 11:123754844:C | 9 | 9 | 50 |
| 11:124750453:C | 5 | 38 | 46 |
| 11:124750455:A | 10 | 165 | 48 |
| 11:1264183:A | 1 | 53 | 16 |
| 11:1264188:A | 1 | 54 | 14 |
| 11:1264569:A | 1 | 23 | 25 |
| 11:1264742:C | 5 | 65 |  |
| 11:1265627:T | 2 | 85 |  |
| 11:1265854:G | 1 | 93 | 17 |
| 11:1266479:T | 1 | 123 | 18 |
| 11:1266892:G | 1 | 58 | 15 |
| 11:1268931:C | 1 | 18 | 13 |
| 11:1270767:C | 1 | 172 | 21 |
| 11:1271223:G | 2 | 135 |  |
| 11:129991670:T | 4 | 172 | 49 |
| 11:132200049:A | 1 | 19 | 49 |
| 11:132200056:T | 2 | 11 | 50 |
| 11:134856252:G | 2 | 44.5 |  |
| 11:14991539:C | 10 | 25 | 50 |
| 11:1642884:C | 4 | 289 | 49 |
| 11:1642989:A | 1 | 314 | 46 |
| 11:1643246:G | 1 | 105 | 44 |
| 11:1643267:A | 1 | 88 | 42 |
| 11:1651157:G | 1 | 178 | 36 |
| 11:1651161:C | 5 | 167 | 46 |
| 11:1651199:G | 1 | 100 | 45 |
| 11:1651211:A | 2 | 133 | 43 |
| 11:1651643:T | 4 | 105 | 46 |
| 11:193722:G | 1 | 89 | 49 |
| 11:1959707:T | 2 | 27 | 49 |
| 11:22881002:T | 6 | 37 | 49 |
| 11:27384466:T | 1 | 10 |  |
| 11:27384469:C | 2 | 117 | 42 |
| 11:320606:T | 1 | 288 | 48 |
| 11:320649:A | 1 | 115.5 |  |
| 11:33763565:A | 1 | 6 | 49 |
| 11:35473473:T | 1 | 27 | 50 |
| 11:36440836:G | 1 | 30 | 49 |
| 11:46724722:A | 5 | 6.5 |  |
| 11:46724728:C | 5 | 3.5 |  |
| 11:47788669:C | 2 | 8.5 |  |
| 11:47788670:C | 6 | 62 |  |
| 11:49059278:T | 1 | 12 | 49 |
| 11:4976013:G | 2 | 10 | 17 |
| 11:4976077:G | 2 | 28 | 33 |
| 11:4976444:T | 1 | 12 | 31 |
| 11:4976654:T | 1 | 4.5 |  |
| 11:4976659:T | 1 | 5 |  |
| 11:502144:A | 2 | 6 |  |
| 11:5221442:T | 3 | 11.5 |  |
| 11:55029787:A | 1 | 19 | 48 |
| 11:5510426:A | 8 | 27 | 49 |
| 11:55606812:G | 1 | 7 |  |
| 11:56143537:T | 1 | 32 | 14 |
| 11:56143539:C | 1 | 32 | 14 |
| 11:56143543:C | 1 | 32 | 14 |
| 11:56143544:C | 1 | 32 | 14 |
| 11:56143556:A | 2 | 23 | 9 |
| 11:56143559:T | 3 | 23 | 9 |
| 11:56143561:G | 3 | 23 | 9 |
| 11:56143562:G | 3 | 23 | 9 |
| 11:56143592:T | 1 | 57 | 48 |
| 11:5629607:A | 7 | 10 | 50 |
| 11:56344846:A | 2 | 6 |  |
| 11:56344847:G | 2 | 2 |  |
| 11:56468554:C | 9 | 46 | 42 |
| 11:56468560:C | 8 | 48 | 43 |
| 11:56468561:G | 8 | 49 | 43 |
| 11:5688940:T | 1 | 16 | 50 |
| 11:59271101:A | 1 | 9.5 |  |
| 11:59271103:T | 1 | 9 |  |
| 11:59945745:C | 2 | 40 | 49 |
| 11:60165353:G | 12 | 2.5 |  |
| 11:60291413:T | 2 | 38 | 49 |
| 11:60971069:G | 3 | 20 | 11 |
| 11:60971694:G | 6 | 11 | 35 |
| 11:62847453:C | 2 | 32 | 49 |
| 11:637373:C | 1 | 34 |  |
| 11:640061:C | 1 | 23 | 35 |
| 11:640064:A | 1 | 20 | 35 |
| 11:640065:C | 1 | 20 | 35 |
| 11:64591972:C | 6 | 38 | 49 |
| 11:65325348:G | 2 | 165 |  |
| 11:65325349:C | 2 | 76 |  |
| 11:65383896:C | 10 | 30 | 49 |
| 11:65601560:C | 2 | 16 | 39 |
| 11:68777323:C | 1 | 32 | 50 |
| 11:71249125:G | 2 | 34.5 |  |
| 11:71249152:T | 12 | 89 | 39 |
| 11:71249158:C | 3 | 43.5 |  |
| 11:71249605:T | 1 | 123 | 47 |
| 11:71276655:A | 1 | 83 | 35 |
| 11:71276681:C | 1 | 80 | 36 |
| 11:71276702:T | 8 | 135.5 |  |
| 11:71276909:A | 1 | 255 | 46 |
| 11:71276948:C | 1 | 255 | 45 |
| 11:71544269:T | 6 | 28 | 26 |
| 11:7656809:A | 1 | 24 | 49 |
| 11:76701606:A | 10 | 21 | 50 |
| 11:76751587:A | 1 | 28 | 49 |
| 11:76928356:A | 1 | 22 | 49 |
| 11:772490:A | 3 | 25 | 49 |
| 11:77921062:G | 1 | 9 |  |
| 11:7847208:C | 1 | 121 | 49 |
| 11:7847466:G | 10 | 70 | 49 |
| 11:7847472:T | 7 | 72 | 49 |
| 11:82973004:T | 1 | 32 | 49 |
| 11:89531619:G | 2 | 324 | 23 |
| 11:89536930:A | 1 | 129 | 4 |
| 11:89774363:A | 1 | 163 |  |
| 11:95825383:T | 18 | 97 | 48 |
| 11:95825407:T | 19 | 142 | 49 |
| 11:985547:T | 6 | 314 | 49 |
| 12:10041364:A | 11 | 13 | 50 |
| 12:101368625:A | 1 | 30 | 49 |
| 12:106641502:T | 17 | 178 | 49 |
| 12:10763236:C | 7 | 26 | 50 |
| 12:109017465:G | 1 | 5 |  |
| 12:109017465:T | 2 | 6 |  |
| 12:109017673:T | 1 | 33 | 45 |
| 12:109017674:G | 1 | 33 | 45 |
| 12:11150240:C | 4 | 107 | 50 |
| 12:11174276:A | 10 | 71 | 50 |
| 12:11174753:A | 1 | 116 | 49 |
| 12:11175087:A | 2 | 90 | 49 |
| 12:11183217:T | 2 | 109 | 49 |
| 12:11183255:A | 1 | 114 | 49 |
| 12:11183451:T | 2 | 112 | 49 |
| 12:11183512:G | 1 | 106 | 49 |
| 12:11183832:A | 2 | 98 | 49 |
| 12:11187101:A | 1 | 79 | 38 |
| 12:11187133:T | 2 | 85 | 40 |
| 12:111993712:T | 1 | 23 | 50 |
| 12:11214145:T | 2 | 128 | 49 |
| 12:11214212:T | 2 | 107 | 49 |
| 12:11214632:C | 1 | 6 |  |
| 12:11214634:C | 1 | 162 |  |
| 12:112375990:C | 5 | 24 | 50 |
| 12:11286790:C | 5 | 81 | 48 |
| 12:113717990:A | 1 | 20 | 49 |
| 12:11420589:C | 1 | 194 |  |
| 12:11461583:G | 2 | 92 | 28 |
| 12:11461584:G | 2 | 91 | 27 |
| 12:118506348:T | 8 | 139 |  |
| 12:118511677:A | 1 | 27 | 38 |
| 12:122064785:G | 19 | 183 | 45 |
| 12:123011395:G | 1 | 3 |  |
| 12:123186880:T | 1 | 128 | 31 |
| 12:131323639:G | 1 | 20 | 50 |
| 12:132547093:G | 2 | 204 |  |
| 12:133049481:C | 1 | 16 | 22 |
| 12:133049516:G | 4 | 28 | 26 |
| 12:133049751:A | 1 | 29 | 34 |
| 12:21487544:G | 1 | 31 | 50 |
| 12:22063115:G | 10 | 15 | 50 |
| 12:25368462:T | 10 | 16 | 49 |
| 12:25656706:C | 2 | 27 | 50 |
| 12:27867727:A | 1 | 21 | 50 |
| 12:29521193:G | 6 | 25 | 49 |
| 12:40852535:G | 2 | 26 | 50 |
| 12:40875390:A | 4 | 24 | 41 |
| 12:40875413:A | 8 | 3 |  |
| 12:40875415:T | 8 | 5 |  |
| 12:40876352:C | 3 | 16 |  |
| 12:40876719:A | 3 | 98 | 46 |
| 12:40876740:T | 1 | 96 | 45 |
| 12:40876741:G | 1 | 94 | 45 |
| 12:40876752:A | 1 | 100 | 46 |
| 12:40876891:A | 1 | 88 | 44 |
| 12:47168898:A | 6 | 14 | 50 |
| 12:48439141:T | 2 | 32 | 50 |
| 12:49171288:A | 1 | 56 |  |
| 12:49724085:G | 1 | 58.5 |  |
| 12:50745783:A | 1 | 152 | 29 |
| 12:50745791:G | 1 | 137 | 28 |
| 12:50745821:A | 1 | 99 | 40 |
| 12:50745822:G | 1 | 98 | 40 |
| 12:50745863:G | 1 | 42 | 35 |
| 12:50745863:G,A | 1 | 42 | 35 |
| 12:50749739:G | 1 | 4.5 |  |
| 12:51740409:G | 5 | 3 |  |
| 12:51740410:G | 5 | 3 |  |
| 12:52402998:T | 1 | 25 | 50 |
| 12:52435691:A | 1 | 18 | 50 |
| 12:52631313:G | 1 | 25 | 50 |
| 12:52696930:C | 1 | 141 | 4 |
| 12:52844400:T | 1 | 143.5 |  |
| 12:53045615:G | 5 | 84.5 |  |
| 12:53073837:T | 1 | 97 |  |
| 12:53167395:T | 2 | 21 | 49 |
| 12:53343132:A | 1 | 77 | 28 |
| 12:53343133:T | 1 | 79 | 28 |
| 12:56184353:C | 1 | 14 | 49 |
| 12:6450945:C | 7 | 23 | 49 |
| 12:7045897:A | 1 | 77 | 49 |
| 12:7080212:C | 30 | 78 | 50 |
| 12:72070509:C | 1 | 141 |  |
| 12:80765800:G | 3 | 33 | 49 |
| 12:8325226:C | 1 | 13 | 49 |
| 12:9085337:A | 1 | 127.5 |  |
| 12:9315209:G | 7 | 28 | 50 |
| 12:95774672:G | 2 | 20 | 49 |
| 12:97098549:C | 8 | 23 | 49 |
| 12:97098555:T | 1 | 29 | 49 |
| 12:9847364:C | 1 | 28 | 50 |
| 12:9994450:A | 16 | 123 | 50 |
| 13:100888122:G | 2 | 23 | 50 |
| 13:107145463:C | 2 | 27 | 49 |
| 13:108518717:G | 1 | 51 |  |
| 13:110839550:G | 10 | 22 | 50 |
| 13:112722441:A | 1 | 15.5 |  |
| 13:114312354:G | 10 | 26 | 49 |
| 13:114325815:G | 1 | 3 |  |
| 13:114503880:A | 3 | 88 |  |
| 13:20006620:T | 1 | 19 | 28 |
| 13:24322265:T | 1 | 26 | 49 |
| 13:24436475:T | 1 | 30 | 49 |
| 13:24468329:G | 4 | 13 | 26 |
| 13:24471039:G | 1 | 74.5 |  |
| 13:25440318:A | 1 | 31 | 50 |
| 13:25671163:A | 4 | 87 | 21 |
| 13:26148966:T | 10 | 21 | 50 |
| 13:28674628:C | 4 | 22 | 50 |
| 13:31309784:T | 1 | 30 | 49 |
| 13:32676114:G | 5 | 27 | 49 |
| 13:38138689:T | 5 | 17 | 49 |
| 13:39263714:C | 1 | 17 | 50 |
| 13:43639845:C | 5 | 29 | 49 |
| 13:45148705:C | 1 | 163 | 50 |
| 13:46108853:C | 10 | 13 | 49 |
| 13:46108854:A | 9 | 13 | 49 |
| 13:61102581:C | 3 | 65.5 |  |
| 13:77736068:C | 1 | 22 | 50 |
| 13:78146301:T | 1 | 26 | 50 |
| 14:105352889:A | 5 | 2 |  |
| 14:105352890:C | 5 | 8 |  |
| 14:106053289:G | 4 | 5 |  |
| 14:106053293:A | 1 | 1 |  |
| 14:106053321:C | 5 | 4 |  |
| 14:106053322:A | 5 | 4 |  |
| 14:106053454:C | 1 | 55 | 43 |
| 14:106053461:A | 1 | 55 | 42 |
| 14:106111107:G | 2 | 56.5 |  |
| 14:106354418:A | 3 | 17 | 46 |
| 14:106363818:A | 1 | 14 | 37 |
| 14:106363835:C | 1 | 12 | 39 |
| 14:106363843:G | 1 | 13 | 41 |
| 14:106382687:T | 8 | 24 | 50 |
| 14:106471448:G | 1 | 162 | 29 |
| 14:106471449:A | 1 | 162 | 29 |
| 14:106478183:T | 10 | 16 | 30 |
| 14:106478194:T | 10 | 11 | 30 |
| 14:106478242:A | 1 | 7 |  |
| 14:106478243:T | 1 | 6 |  |
| 14:106478244:G | 1 | 5 |  |
| 14:106478252:T | 1 | 6 |  |
| 14:106478278:T | 2 | 1.5 |  |
| 14:106478280:G | 2 | 1 |  |
| 14:106478287:G | 5 | 3 |  |
| 14:106478292:C | 5 | 3.5 |  |
| 14:106478296:A | 5 | 3.5 |  |
| 14:106478302:C | 4 | 4 |  |
| 14:106478303:C | 3 | 4 |  |
| 14:106791006:T | 8 | 128 | 46 |
| 14:106791119:G | 5 | 140 | 44 |
| 14:106791131:G | 6 | 154 | 46 |
| 14:106791200:A | 6 | 220 | 38 |
| 14:106791201:G | 6 | 221 | 37 |
| 14:106805242:G | 1 | 20 | 4 |
| 14:106815723:T | 1 | 47 | 2 |
| 14:106815970:C | 1 | 39.5 |  |
| 14:106926223:C | 1 | 88 | 49 |
| 14:106926287:A | 7 | 118 | 49 |
| 14:106926311:G | 5 | 149 | 49 |
| 14:107095153:T | 1 | 197 | 28 |
| 14:107095156:G | 2 | 196 | 28 |
| 14:107178938:C | 9 | 85 | 36 |
| 14:107178965:C | 2 | 78 | 37 |
| 14:19377907:C | 1 | 174 | 1 |
| 14:19553642:G | 1 | 39 | 2 |
| 14:19562053:T | 1 | 6 |  |
| 14:19574244:G | 1 | 124 | 5 |
| 14:20014613:T | 2 | 210 | 4 |
| 14:20528451:C | 14 | 4.5 |  |
| 14:21557021:A | 4 | 26 | 49 |
| 14:21560770:G | 4 | 137 | 47 |
| 14:22102293:T | 1 | 7 | 29 |
| 14:22102333:C | 6 | 12 | 18 |
| 14:22102374:C | 9 | 26 | 34 |
| 14:22102592:C | 10 | 18 | 32 |
| 14:22102612:C | 10 | 16 | 32 |
| 14:22133416:G | 1 | 24 | 49 |
| 14:22236756:C | 10 | 21 | 49 |
| 14:22476139:A | 3 | 6 |  |
| 14:22476139:T | 2 | 8 |  |
| 14:22476143:T | 3 | 153.5 |  |
| 14:22476144:T | 5 | 178 | 49 |
| 14:22476146:T | 4 | 148 | 50 |
| 14:22476147:C | 3 | 150 |  |
| 14:22476147:G | 1 | 85.5 |  |
| 14:22997524:G | 1 | 30 | 50 |
| 14:23000062:C | 2 | 42 | 49 |
| 14:23549896:T | 2 | 148 |  |
| 14:32419331:T | 4 | 18 | 49 |
| 14:53019985:C | 1 | 8 | 50 |
| 14:55864130:G | 1 | 33 | 49 |
| 14:56105908:T | 1 | 26 | 50 |
| 14:58598408:A | 1 | 145 |  |
| 14:60063472:G | 11 | 27 | 49 |
| 14:60932752:A | 2 | 28 | 48 |
| 14:63735862:T | 1 | 31 | 50 |
| 14:70039805:A | 14 | 41 |  |
| 14:70039809:A | 1 | 40 | 46 |
| 14:70039809:A,C | 1 | 40 | 46 |
| 14:70039809:C | 21 | 51 | 46 |
| 14:70039824:C | 14 | 75.5 |  |
| 14:74010331:G | 6 | 160 | 16 |
| 14:74992800:G | 9 | 22 | 49 |
| 14:76452150:A | 1 | 27 | 49 |
| 14:77493794:C | 2 | 155.5 |  |
| 14:77493809:T | 1 | 141 | 49 |
| 14:77697967:G | 10 | 16 | 50 |
| 14:78234796:T | 1 | 10 | 48 |
| 14:88852166:A | 6 | 13 | 50 |
| 14:88862529:A | 4 | 17 | 49 |
| 14:92537364:T | 1 | 74 | 45 |
| 14:94847285:G | 6 | 122 | 49 |
| 14:94847415:G | 5 | 92 | 50 |
| 15:100794363:T | 7 | 19 | 49 |
| 15:20739883:T | 1 | 35 | 25 |
| 15:20739886:A | 1 | 37 | 27 |
| 15:20739887:C | 1 | 39 | 27 |
| 15:20740292:C | 8 | 1 |  |
| 15:20740293:C | 7 | 3 |  |
| 15:20740294:T | 8 | 4 |  |
| 15:20740295:T | 6 | 5 |  |
| 15:20740347:C | 1 | 66 |  |
| 15:20743796:T | 2 | 14 | 9 |
| 15:21004910:A | 1 | 218 | 0 |
| 15:21063501:G | 1 | 24 | 3 |
| 15:21071460:T | 2 | 237 | 3 |
| 15:21071485:G | 1 | 210 | 4 |
| 15:22074657:G | 2 | 38 | 3 |
| 15:22077592:C | 1 | 147 | 5 |
| 15:22368862:A | 1 | 302 | 49 |
| 15:22368905:A | 2 | 262 | 49 |
| 15:22369290:G | 2 | 231 | 46 |
| 15:22369426:A | 2 | 114 | 49 |
| 15:22383189:A | 2 | 353 | 49 |
| 15:22383249:A | 6 | 370 | 49 |
| 15:22473042:T | 2 | 186 | 49 |
| 15:22473054:T | 2 | 327 |  |
| 15:22473061:A | 3 | 343 |  |
| 15:22473063:G | 3 | 340 |  |
| 15:22473106:A | 3 | 365 |  |
| 15:23264144:G | 1 | 73 | 14 |
| 15:23406990:T | 1 | 66 | 24 |
| 15:23406993:A | 1 | 68 | 25 |
| 15:23406993:T | 1 | 105 | 30 |
| 15:23406994:C | 1 | 68 | 25 |
| 15:23407160:C | 1 | 19 | 25 |
| 15:23407162:T | 1 | 18 | 25 |
| 15:23407163:A | 1 | 18 | 25 |
| 15:23407173:G | 1 | 17 | 24 |
| 15:23603641:A | 6 | 15 | 17 |
| 15:23605893:A | 1 | 17 | 40 |
| 15:23606545:G | 2 | 18 | 17 |
| 15:23606546:G | 2 | 20 | 16 |
| 15:23685904:G | 3 | 197 | 42 |
| 15:23685913:C | 5 | 194 | 43 |
| 15:23686207:G | 1 | 156 | 45 |
| 15:23686217:C | 1 | 160 | 45 |
| 15:23686218:T | 1 | 161 | 45 |
| 15:28235773:T | 7 | 14 | 50 |
| 15:28502279:G | 7 | 65 | 49 |
| 15:28566562:C | 1 | 15 | 34 |
| 15:28625673:G | 1 | 63 | 2 |
| 15:28632820:C | 2 | 125 |  |
| 15:28949049:C | 1 | 54 | 15 |
| 15:30381003:A | 1 | 72 | 3 |
| 15:30437693:G | 1 | 111 | 18 |
| 15:30696687:C | 1 | 33 | 9 |
| 15:30699639:C | 1 | 60 | 10 |
| 15:30699723:C | 1 | 91 | 10 |
| 15:30700159:G | 6 | 66 | 37 |
| 15:30700618:A | 1 | 72 | 26 |
| 15:31362352:T | 4 | 67 | 49 |
| 15:31453147:A | 1 | 29 | 50 |
| 15:32685060:G | 3 | 18 | 19 |
| 15:32688214:T | 4 | 50.5 |  |
| 15:32690804:G | 2 | 142 | 3 |
| 15:32690806:C | 1 | 123 |  |
| 15:32690806:G | 1 | 145 | 3 |
| 15:33091105:C | 1 | 6 |  |
| 15:34673722:T | 3 | 197 |  |
| 15:34673973:T | 9 | 175 | 3 |
| 15:34674006:T | 3 | 159 | 4 |
| 15:34674008:A | 1 | 162 | 3 |
| 15:34677517:A | 8 | 108 |  |
| 15:34820227:T | 1 | 164 |  |
| 15:34820404:C | 2 | 207 | 15 |
| 15:35271874:G | 1 | 12 |  |
| 15:38776827:A | 2 | 116 | 46 |
| 15:38776830:A | 1 | 133 | 47 |
| 15:40453430:T | 2 | 21 | 49 |
| 15:42386630:T | 1 | 19 | 49 |
| 15:43814989:G | 1 | 20 |  |
| 15:43817807:A | 1 | 13.5 |  |
| 15:47425885:G | 1 | 14 | 10 |
| 15:48443699:C | 10 | 29 | 50 |
| 15:48807637:T | 10 | 11 | 50 |
| 15:50875343:T | 2 | 4 |  |
| 15:54841874:G | 2 | 29 | 49 |
| 15:55700981:A | 4 | 15 | 50 |
| 15:57967219:C | 10 | 12 | 50 |
| 15:60690089:G | 10 | 19 | 49 |
| 15:65042560:A | 1 | 21 | 49 |
| 15:65134221:T | 1 | 8 | 50 |
| 15:65236875:C | 1 | 14 | 49 |
| 15:69652451:A | 1 | 33 | 49 |
| 15:70975111:T | 1 | 19 | 50 |
| 15:72955443:T | 1 | 81 | 2 |
| 15:74001993:C | 3 | 21 | 50 |
| 15:74002006:A | 1 | 24 | 49 |
| 15:74365090:C | 1 | 166 |  |
| 15:74366897:A | 1 | 148 | 5 |
| 15:74368293:G | 1 | 16 | 25 |
| 15:74536404:G | 9 | 92 | 50 |
| 15:75581966:T | 1 | 174 |  |
| 15:77176158:C | 1 | 10 | 49 |
| 15:78441769:C | 6 | 12 | 50 |
| 15:82387862:C | 2 | 36 | 49 |
| 15:82555242:G | 2 | 26 | 49 |
| 15:82637611:T | 1 | 30 | 6 |
| 15:82932833:A | 1 | 144 | 5 |
| 15:84651452:C | 2 | 6 |  |
| 15:90248819:T | 2 | 19 | 50 |
| 15:90260145:A | 6 | 25 | 49 |
| 15:90320161:A | 5 | 182 | 49 |
| 15:90320173:G | 4 | 70.5 |  |
| 15:93198684:T | 6 | 11.5 |  |
| 15:93198687:C | 17 | 182 | 43 |
| 15:93198688:C | 18 | 209 | 38 |
| 15:93616975:G | 10 | 22 | 50 |
| 15:99544418:T | 1 | 13 | 49 |
| 15:99544429:A | 10 | 15 | 50 |
| 16:10524659:G | 14 | 19 | 50 |
| 16:12021337:A | 1 | 69 | 43 |
| 16:1279732:A | 1 | 209 | 31 |
| 16:1279908:C | 1 | 57 | 7 |
| 16:1279909:G | 1 | 55 | 7 |
| 16:1290947:G | 1 | 160 | 25 |
| 16:1290948:C | 1 | 161 | 25 |
| 16:1290965:C | 1 | 181 | 24 |
| 16:1291160:T | 2 | 174 | 21 |
| 16:1291175:A | 1 | 167 | 25 |
| 16:1291178:C | 1 | 166 | 25 |
| 16:1291182:T | 1 | 162 | 27 |
| 16:1291318:A | 1 | 394 | 33 |
| 16:1291318:C | 4 | 394 | 33 |
| 16:1291318:C,A | 2 | 328 | 31 |
| 16:1291454:A | 3 | 218 | 6 |
| 16:1306355:C | 1 | 16 | 22 |
| 16:1306671:A | 1 | 151 | 23 |
| 16:1306681:A | 1 | 155 | 24 |
| 16:1306681:A,C | 1 | 155 | 24 |
| 16:1509123:A | 1 | 23 | 50 |
| 16:15463612:A | 6 | 104 | 12 |
| 16:16278863:T | 3 | 33 | 49 |
| 16:16278869:C | 1 | 43 | 49 |
| 16:16306059:T | 1 | 6 |  |
| 16:1695395:T | 1 | 27 | 49 |
| 16:2059737:A | 1 | 4.5 |  |
| 16:21416509:A | 1 | 122 | 4 |
| 16:21742179:T | 1 | 42 | 8 |
| 16:22269867:G | 3 | 102 | 50 |
| 16:22545285:C | 1 | 47 |  |
| 16:24583715:G | 1 | 19 | 49 |
| 16:25239805:C | 5 | 30 | 49 |
| 16:27974487:C | 6 | 34 | 49 |
| 16:28354319:G | 1 | 55 | 5 |
| 16:28354353:G | 3 | 50 | 14 |
| 16:28468295:G | 1 | 118 |  |
| 16:28507424:T | 2 | 103 | 46 |
| 16:28507452:T | 1 | 102 | 46 |
| 16:28649787:A | 2 | 10 | 19 |
| 16:29063445:T | 6 | 24 |  |
| 16:29791561:G | 10 | 28 | 50 |
| 16:30671235:A | 1 | 28 | 50 |
| 16:30936081:A | 3 | 27 | 50 |
| 16:31196423:C | 1 | 21 | 48 |
| 16:3170252:C | 1 | 4 |  |
| 16:3170253:T | 1 | 2 |  |
| 16:32077496:T | 8 | 113 | 46 |
| 16:32077537:T | 9 | 111 | 46 |
| 16:32077580:G | 6 | 97 | 45 |
| 16:4386814:C | 6 | 70 | 50 |
| 16:4445327:T | 7 | 36 | 49 |
| 16:47697618:G | 1 | 27 | 49 |
| 16:4930100:T | 2 | 29 | 49 |
| 16:55866949:C | 1 | 3 |  |
| 16:57702274:T | 1 | 27 | 50 |
| 16:57950057:A | 4 | 24 | 50 |
| 16:67229811:A | 1 | 21 | 43 |
| 16:67229817:A | 1 | 18 | 41 |
| 16:68088485:G | 3 | 28 | 49 |
| 16:69493016:G | 2 | 17 | 50 |
| 16:69988472:T | 2 | 24 | 15 |
| 16:70178401:T | 2 | 17 | 34 |
| 16:71487952:T | 3 | 19 | 49 |
| 16:71956529:T | 2 | 150 | 49 |
| 16:72042682:C | 8 | 12 | 49 |
| 16:72822033:T | 1 | 13 | 47 |
| 16:72992611:C | 1 | 111 |  |
| 16:74640753:C | 1 | 172 |  |
| 16:77233394:G | 1 | 35 | 50 |
| 16:80584432:A | 1 | 25 | 50 |
| 16:82203742:C | 5 | 23 | 47 |
| 16:82203758:T | 10 | 26 | 48 |
| 16:82203768:T | 8 | 27 | 48 |
| 16:830767:A | 1 | 45 |  |
| 16:830767:T | 1 | 108 |  |
| 16:83981825:T | 1 | 21 | 50 |
| 16:84030848:T | 1 | 25 | 49 |
| 16:84063130:G | 1 | 29 | 49 |
| 16:84158248:C | 1 | 18 | 50 |
| 16:85689986:A | 1 | 24.5 |  |
| 16:85689988:G | 3 | 49 |  |
| 16:85690000:T | 1 | 159 | 49 |
| 16:85948098:A | 2 | 27 | 50 |
| 16:863358:C | 6 | 4 |  |
| 16:863362:G | 6 | 7 |  |
| 16:863369:G | 1 | 10 | 49 |
| 16:863372:A | 2 | 18 | 49 |
| 16:88594544:G | 2 | 128 |  |
| 16:88599697:C | 6 | 58 |  |
| 16:88599701:C | 6 | 3 |  |
| 16:88717386:C | 4 | 33 | 49 |
| 16:88787610:C | 2 | 17.5 |  |
| 16:89264532:G | 2 | 3 |  |
| 16:90095573:T | 6 | 189 | 45 |
| 17:13972955:T | 1 | 22 | 49 |
| 17:15449158:G | 7 | 114 |  |
| 17:15510988:C | 2 | 26 | 15 |
| 17:15517284:C | 3 | 25 | 7 |
| 17:15604485:C | 1 | 97 | 37 |
| 17:15878112:C | 7 | 14 | 50 |
| 17:1733399:G | 10 | 23 | 50 |
| 17:17697102:A | 1 | 135 | 50 |
| 17:17948475:A | 2 | 42 | 50 |
| 17:18291544:G | 5 | 9 | 39 |
| 17:18659367:G | 9 | 132 | 27 |
| 17:18682505:C | 1 | 13 | 11 |
| 17:19608773:A | 1 | 25 | 50 |
| 17:19713740:T | 6 | 25 | 49 |
| 17:19729495:C | 4 | 30 | 49 |
| 17:20370767:C | 7 | 20 | 23 |
| 17:21318698:T | 1 | 49 | 48 |
| 17:21318821:C | 1 | 92 | 48 |
| 17:21319007:A | 3 | 80 | 49 |
| 17:21319302:C | 1 | 95 | 49 |
| 17:21319785:T | 1 | 88 | 49 |
| 17:21319860:T | 1 | 95 | 49 |
| 17:21319943:G | 1 | 88 | 49 |
| 17:25931745:T | 1 | 25 | 50 |
| 17:25958304:A | 2 | 24 | 49 |
| 17:26684392:C | 22 | 138 | 49 |
| 17:29159404:C | 1 | 38 | 49 |
| 17:29161960:T | 3 | 8 |  |
| 17:2966828:T | 2 | 54 | 4 |
| 17:30183857:C | 1 | 28 | 50 |
| 17:30351758:A | 2 | 27 | 36 |
| 17:34261831:G | 1 | 26 | 49 |
| 17:34523265:C | 1 | 87 | 5 |
| 17:34624839:C | 1 | 111 | 4 |
| 17:35696804:A | 15 | 15 | 49 |
| 17:35771468:T | 11 | 18 | 50 |
| 17:36290036:C | 2 | 32 | 9 |
| 17:36339584:T | 1 | 82 | 6 |
| 17:36339597:T | 4 | 88 | 5 |
| 17:36352489:C | 6 | 38 | 40 |
| 17:36353761:T | 9 | 130 | 48 |
| 17:36365172:T | 2 | 50 | 30 |
| 17:36474601:G | 2 | 26 | 49 |
| 17:36493598:G | 10 | 29 | 49 |
| 17:37815326:T | 1 | 18 |  |
| 17:38715186:C | 1 | 28 | 49 |
| 17:38975158:G | 2 | 100 | 46 |
| 17:38975158:T | 1 | 115 | 50 |
| 17:39197499:A | 1 | 17 | 38 |
| 17:39197601:C | 3 | 20 | 28 |
| 17:39197605:T | 3 | 25 | 32 |
| 17:39197609:A | 1 | 26 | 36 |
| 17:39240661:G | 1 | 163 | 47 |
| 17:39240729:G | 5 | 245 |  |
| 17:39240737:G | 6 | 234 |  |
| 17:39240795:T | 6 | 79 |  |
| 17:39240796:C | 2 | 27 |  |
| 17:39253815:A | 5 | 153 | 47 |
| 17:39261851:G | 1 | 34 | 28 |
| 17:39305773:G | 1 | 110.5 |  |
| 17:39305774:G | 1 | 110.5 |  |
| 17:39305775:C | 1 | 108 |  |
| 17:39305785:T | 13 | 202 | 44 |
| 17:39340729:A | 1 | 91 |  |
| 17:39340848:C | 4 | 119 | 36 |
| 17:39382942:A | 3 | 71 | 41 |
| 17:39383012:T | 9 | 89 | 43 |
| 17:39383156:A | 1 | 108 | 48 |
| 17:39394674:G | 8 | 79 | 29 |
| 17:39394717:T | 1 | 17 | 18 |
| 17:39406104:G | 3 | 106 | 44 |
| 17:39421971:C | 9 | 4.5 |  |
| 17:39471781:C | 2 | 72.5 |  |
| 17:39535388:C | 9 | 183 | 49 |
| 17:41121194:G | 1 | 13 | 49 |
| 17:41121195:T | 1 | 13 | 49 |
| 17:42927721:A | 1 | 32 | 49 |
| 17:43545893:C | 7 | 281 | 49 |
| 17:43552717:C | 3 | 495 | 37 |
| 17:43552921:T | 4 | 741 | 48 |
| 17:43922942:C | 1 | 115 | 49 |
| 17:43923180:A | 1 | 94 | 49 |
| 17:43923266:A | 3 | 96 | 49 |
| 17:43923683:G | 2 | 106 | 49 |
| 17:43923703:T | 3 | 95 | 49 |
| 17:43924073:C | 4 | 116 | 49 |
| 17:43924130:A | 4 | 98 | 49 |
| 17:43924200:G | 2 | 77 | 49 |
| 17:43924219:C | 2 | 84 | 49 |
| 17:43924231:A | 3 | 84 | 49 |
| 17:44060775:T | 8 | 224 | 49 |
| 17:44061023:A | 7 | 236 | 49 |
| 17:44061025:T | 8 | 237 | 49 |
| 17:44061036:C | 7 | 233 | 49 |
| 17:44061278:T | 4 | 110 | 49 |
| 17:44067382:C | 4 | 69 | 49 |
| 17:44067400:C | 8 | 80 | 49 |
| 17:44068924:A | 7 | 98 | 49 |
| 17:44071294:C | 4 | 16 | 50 |
| 17:44073889:G | 7 | 203 | 49 |
| 17:44073973:C | 8 | 200 | 49 |
| 17:44108906:G | 6 | 701 | 49 |
| 17:44110532:A | 1 | 476 | 49 |
| 17:44159849:C | 5 | 57 | 49 |
| 17:44248769:C | 5 | 429 | 49 |
| 17:44248814:A | 7 | 392 | 49 |
| 17:44409255:G | 1 | 239 | 17 |
| 17:44409341:A | 1 | 251 | 10 |
| 17:44626347:C | 5 | 170 | 30 |
| 17:44626773:C | 5 | 36 | 6 |
| 17:44828931:A | 6 | 50 | 49 |
| 17:46115079:T | 2 | 42 | 50 |
| 17:46608203:G | 1 | 56.5 |  |
| 17:47921426:T | 1 | 3 |  |
| 17:4837171:A | 4 | 116 | 46 |
| 17:4837204:T | 1 | 119 | 44 |
| 17:5087041:G | 3 | 74 |  |
| 17:51063068:G | 1 | 22 | 50 |
| 17:56056607:T | 2 | 94 | 49 |
| 17:56386607:T | 1 | 72.5 |  |
| 17:56544280:G | 1 | 10 | 50 |
| 17:56618030:T | 3 | 5 | 50 |
| 17:60351457:A | 3 | 14 | 32 |
| 17:61950694:A | 1 | 224 | 24 |
| 17:61973865:C | 1 | 20 | 9 |
| 17:62892031:T | 1 | 149 | 6 |
| 17:62892271:T | 2 | 182 | 21 |
| 17:64023642:G | 2 | 19 | 49 |
| 17:6406883:T | 7 | 27 | 50 |
| 17:64208285:G | 1 | 18 | 49 |
| 17:65212042:T | 5 | 21 | 49 |
| 17:67190540:A | 2 | 5 |  |
| 17:72889676:C | 2 | 91 | 46 |
| 17:72889685:A | 13 | 89 | 44 |
| 17:73926121:A | 2 | 33 | 50 |
| 17:74288410:T | 1 | 110 | 45 |
| 17:74288421:G | 2 | 99 | 45 |
| 17:74288421:G,T | 5 | 99 | 45 |
| 17:74288567:C | 10 | 6 |  |
| 17:77769127:G | 1 | 21 | 45 |
| 17:77769130:T | 1 | 18 | 44 |
| 17:79395747:A | 1 | 25 | 49 |
| 17:79912164:A | 3 | 24 | 50 |
| 17:80992932:C | 9 | 19 | 50 |
| 17:81009636:G | 1 | 25 | 49 |
| 18:14124359:C | 2 | 24 | 38 |
| 18:14513675:C | 4 | 137 |  |
| 18:14524966:C | 3 | 26 | 29 |
| 18:14542948:A | 7 | 364 | 35 |
| 18:166819:C | 4 | 15 | 49 |
| 18:19032046:A | 1 | 3 |  |
| 18:19032056:G | 1 | 5 |  |
| 18:20716021:T | 4 | 21 | 47 |
| 18:20953720:A | 3 | 29 | 50 |
| 18:28898294:G | 9 | 16 | 49 |
| 18:28956904:T | 2 | 25 | 49 |
| 18:30352078:C | 4 | 148 | 48 |
| 18:3452223:G | 1 | 7.5 |  |
| 18:43420177:A | 5 | 21 | 49 |
| 18:48723154:C | 1 | 31 | 48 |
| 18:49549:C | 3 | 36 | 26 |
| 18:51795960:G | 14 | 17 |  |
| 18:5410574:G | 2 | 38 | 49 |
| 18:59810563:G | 6 | 21 | 49 |
| 18:60492683:T | 1 | 24 |  |
| 18:60646253:C | 1 | 85.5 |  |
| 18:67534642:T | 1 | 29 | 50 |
| 18:71928150:T | 4 | 19 | 49 |
| 18:72201918:A | 5 | 18 | 50 |
| 18:76753768:G | 1 | 322 | 49 |
| 18:76754466:T | 1 | 336 | 49 |
| 18:9117867:C | 1 | 26 | 49 |
| 18:9887416:C | 2 | 103 | 34 |
| 19:1009577:G | 11 | 5 |  |
| 19:11310189:T | 1 | 25 | 49 |
| 19:11437480:G | 1 | 30 | 49 |
| 19:11598120:T | 1 | 6.5 |  |
| 19:11998777:T | 1 | 18 | 50 |
| 19:14200109:C | 1 | 154 | 50 |
| 19:1440856:C | 2 | 6 |  |
| 19:14769339:G | 11 | 17 | 50 |
| 19:14877845:G | 4 | 17 | 24 |
| 19:14877848:A | 4 | 18 | 24 |
| 19:14877857:A | 4 | 25 | 31 |
| 19:14910446:C | 1 | 13 |  |
| 19:1535196:A | 13 | 16 | 47 |
| 19:17049224:A | 1 | 23 | 50 |
| 19:17049230:C | 1 | 23 | 50 |
| 19:1825928:T | 2 | 1 |  |
| 19:1825930:T | 3 | 6 |  |
| 19:20044898:T | 1 | 78 |  |
| 19:20044900:A | 1 | 5 |  |
| 19:20044901:C | 1 | 5 |  |
| 19:20044902:C | 1 | 5.5 |  |
| 19:20044902:G | 1 | 7 |  |
| 19:20044903:A | 1 | 5 |  |
| 19:20044903:C | 1 | 3.5 |  |
| 19:20044903:C,A | 1 | 3.5 |  |
| 19:2015568:C | 1 | 158 |  |
| 19:2050823:T | 2 | 26 | 49 |
| 19:20989992:G | 5 | 32 | 48 |
| 19:24116560:T | 3 | 20 |  |
| 19:2820105:A | 1 | 34 | 50 |
| 19:34012655:A | 1 | 22 | 50 |
| 19:34872382:A | 1 | 24 | 50 |
| 19:35649281:G | 1 | 29 | 50 |
| 19:35660508:A | 8 | 25 | 50 |
| 19:35850711:G | 1 | 209 |  |
| 19:36297966:A | 1 | 182 | 46 |
| 19:36355595:G | 1 | 36 | 49 |
| 19:38202516:C | 4 | 12 | 50 |
| 19:38377414:T | 2 | 121 | 48 |
| 19:38377417:T | 2 | 118 | 48 |
| 19:38795554:G | 1 | 198.5 |  |
| 19:38795587:G | 2 | 186.5 |  |
| 19:39423332:C | 1 | 6 |  |
| 19:40374034:G | 4 | 72 | 30 |
| 19:40376881:C | 1 | 69 | 5 |
| 19:40377034:A | 1 | 283.5 |  |
| 19:40384956:A | 1 | 130 | 4 |
| 19:40385078:C | 7 | 69 | 3 |
| 19:40385103:G | 12 | 86 | 3 |
| 19:40389657:G | 1 | 19 | 11 |
| 19:40389741:G | 1 | 24 | 25 |
| 19:40392359:C | 4 | 23 | 6 |
| 19:40392360:A | 4 | 25 | 6 |
| 19:40392482:G | 2 | 96 | 32 |
| 19:40392585:G | 2 | 329 | 37 |
| 19:40392719:A | 8 | 72 | 26 |
| 19:40399814:G | 3 | 5.5 |  |
| 19:40400766:C | 3 | 91 | 3 |
| 19:40400791:G | 3 | 82.5 |  |
| 19:41628014:C | 1 | 35 | 5 |
| 19:42342319:G | 1 | 34 | 50 |
| 19:42736267:G | 10 | 16 | 50 |
| 19:43269699:T | 4 | 26 | 49 |
| 19:43269704:C | 4 | 27 | 49 |
| 19:43269705:A | 4 | 27 | 49 |
| 19:43709647:A | 2 | 35 | 46 |
| 19:43709654:A | 9 | 34 | 42 |
| 19:43709656:G | 9 | 32 | 42 |
| 19:44351168:A | 11 | 3.5 |  |
| 19:44590001:A | 5 | 3.5 |  |
| 19:44590002:T | 7 | 7.5 |  |
| 19:44778181:G | 1 | 20 | 40 |
| 19:44778796:A | 6 | 6 |  |
| 19:44778798:A | 4 | 81 | 50 |
| 19:44778799:A | 6 | 70 | 50 |
| 19:4499642:A | 14 | 35 | 45 |
| 19:4499647:A | 25 | 44 | 44 |
| 19:45004288:T | 2 | 32 | 50 |
| 19:4512705:C | 1 | 19 | 26 |
| 19:4512891:A | 2 | 4 |  |
| 19:4512910:T | 2 | 42.5 |  |
| 19:4512933:A | 2 | 92.5 |  |
| 19:4512934:G | 2 | 95 |  |
| 19:4512939:C | 2 | 104.5 |  |
| 19:4512945:T | 1 | 67 |  |
| 19:4512958:G | 2 | 109 | 12 |
| 19:4513032:A | 1 | 236 |  |
| 19:4513033:G | 2 | 234.5 |  |
| 19:4513038:C | 1 | 232 |  |
| 19:45213778:G | 1 | 15 | 50 |
| 19:46519369:G | 2 | 5.5 |  |
| 19:47774668:T | 7 | 18 | 50 |
| 19:48305436:A | 2 | 22 | 34 |
| 19:48305564:A | 1 | 94 | 42 |
| 19:48305566:T | 1 | 95 | 42 |
| 19:48364367:T | 1 | 26 | 42 |
| 19:48544837:T | 4 | 18 | 49 |
| 19:48622427:G | 7 | 29 | 49 |
| 19:48950010:G | 1 | 139 |  |
| 19:49000886:A | 1 | 28 | 49 |
| 19:49000886:A,T | 1 | 28 | 49 |
| 19:49447750:T | 1 | 6 |  |
| 19:49526191:A | 2 | 55 | 18 |
| 19:49526203:C | 1 | 78 | 18 |
| 19:49558210:G | 3 | 13 | 20 |
| 19:49558211:G | 3 | 13 | 20 |
| 19:49558216:T | 3 | 20 | 26 |
| 19:49573365:T | 2 | 9 |  |
| 19:496532:A | 2 | 20 | 49 |
| 19:49657526:A | 1 | 168 | 44 |
| 19:49657527:C | 1 | 166 | 44 |
| 19:49657530:C | 1 | 163 | 44 |
| 19:49894152:T | 10 | 31 | 49 |
| 19:49894179:A | 4 | 14 | 49 |
| 19:50156736:A | 1 | 28 | 50 |
| 19:501719:C | 1 | 235 | 46 |
| 19:501786:A | 1 | 274 | 45 |
| 19:50383591:G | 9 | 20 | 50 |
| 19:50484234:A | 5 | 21 | 49 |
| 19:50510999:A | 1 | 110.5 |  |
| 19:50511000:C | 1 | 111 |  |
| 19:50881825:A | 3 | 160 | 48 |
| 19:52096039:C | 1 | 108.5 |  |
| 19:52133292:G | 1 | 202 |  |
| 19:52888071:T | 1 | 105 | 36 |
| 19:53269683:T | 1 | 12 |  |
| 19:54664752:C | 2 | 945 | 49 |
| 19:54676763:T | 7 | 390 | 49 |
| 19:54722275:A | 3 | 602 | 49 |
| 19:54724407:T | 7 | 183 | 38 |
| 19:54724411:T | 5 | 70 | 9 |
| 19:54724431:A | 2 | 53 | 3 |
| 19:54724443:A | 1 | 17 |  |
| 19:54724457:A | 2 | 150 | 13 |
| 19:54724457:A,C | 1 | 150 | 13 |
| 19:54724457:C | 9 | 226 | 26 |
| 19:54724458:G | 10 | 227 | 26 |
| 19:54725156:C | 13 | 36 |  |
| 19:54725745:G | 1 | 11 |  |
| 19:54725755:C | 2 | 12 |  |
| 19:54725756:A | 1 | 11 |  |
| 19:54726226:C | 1 | 217 | 34 |
| 19:54726241:T | 4 | 286 | 27 |
| 19:54726299:C | 1 | 219 | 30 |
| 19:54726628:T | 1 | 203 | 18 |
| 19:54726630:T | 1 | 159 | 12 |
| 19:54726816:T | 6 | 497 | 25 |
| 19:54726839:T | 1 | 122.5 |  |
| 19:54744195:A | 4 | 249 | 33 |
| 19:54744209:C | 1 | 230 | 30 |
| 19:54744210:G | 1 | 226 | 30 |
| 19:54744710:T | 1 | 82.5 |  |
| 19:54744711:G | 1 | 83 |  |
| 19:54744722:C | 1 | 117 | 16 |
| 19:54745496:T | 5 | 246 |  |
| 19:54745507:C | 3 | 689 | 27 |
| 19:54745508:A | 2 | 142 |  |
| 19:54745525:C | 1 | 128.5 |  |
| 19:54745550:T | 2 | 122.5 |  |
| 19:54746051:C | 1 | 161 | 28 |
| 19:54746591:T | 1 | 56.5 |  |
| 19:54778552:A | 2 | 31 | 15 |
| 19:54778581:A | 2 | 12 | 15 |
| 19:54778587:C | 2 | 13 | 15 |
| 19:54849463:T | 1 | 491 | 49 |
| 19:54867572:T | 9 | 645 | 49 |
| 19:54871664:C | 13 | 23 | 44 |
| 19:54937862:T | 1 | 355 | 49 |
| 19:55020248:C | 1 | 11 | 20 |
| 19:55021777:A | 7 | 126 | 46 |
| 19:55045042:A | 6 | 198 | 49 |
| 19:55144186:T | 2 | 213 | 48 |
| 19:55147510:C | 3 | 266 | 49 |
| 19:55178162:G | 1 | 749 | 49 |
| 19:55236747:A | 1 | 13 |  |
| 19:55237677:T | 3 | 99.5 |  |
| 19:55246731:A | 6 | 410 | 48 |
| 19:55258808:T | 4 | 12 | 40 |
| 19:55263898:A | 1 | 2251 | 48 |
| 19:55281315:T | 4 | 20 | 50 |
| 19:55317439:T | 1 | 85 | 3 |
| 19:55327960:T | 5 | 12 |  |
| 19:55329901:G | 10 | 930 | 43 |
| 19:55329922:T | 9 | 890 | 43 |
| 19:55485899:A | 5 | 29 | 49 |
| 19:55536595:A | 3 | 34 | 50 |
| 19:55644325:C | 7 | 30 | 49 |
| 19:55699454:C | 7 | 16 | 50 |
| 19:56241265:G | 10 | 16 | 50 |
| 19:56274193:C | 1 | 155 | 8 |
| 19:56274213:A | 1 | 19 | 9 |
| 19:56274214:T | 1 | 19 | 9 |
| 19:56274503:G | 3 | 20 | 15 |
| 19:56274506:A | 1 | 12 | 5 |
| 19:56274507:G | 1 | 12 | 5 |
| 19:56274525:A | 1 | 26 | 0 |
| 19:56274531:G | 2 | 32 | 1 |
| 19:56284396:T | 15 | 42 | 0 |
| 19:56284428:A | 1 | 29 | 20 |
| 19:56284464:G | 1 | 96 | 27 |
| 19:56599452:C | 6 | 107 | 50 |
| 19:58038964:A | 11 | 25 | 49 |
| 19:58385536:C | 1 | 7 |  |
| 19:6763718:G | 1 | 40 | 50 |
| 19:7011877:A | 2 | 7 |  |
| 19:7011878:A | 2 | 149 |  |
| 19:7051376:A | 4 | 104 |  |
| 19:7051437:A | 1 | 238.5 |  |
| 19:8389898:T | 3 | 132 | 50 |
| 19:8555875:T | 1 | 11 |  |
| 19:8567475:C | 2 | 12 | 49 |
| 19:868115:T | 1 | 645 | 49 |
| 19:868421:A | 1 | 451 | 49 |
| 19:9646898:A | 2 | 22 | 49 |
| 19:9801443:T | 5 | 9 |  |
| 19:9801445:T | 1 | 7 |  |
| 1:100618008:C | 1 | 5 |  |
| 1:10699146:C | 2 | 23 |  |
| 1:10699149:C | 1 | 161 |  |
| 1:108769307:A | 4 | 23 |  |
| 1:110203841:G | 1 | 5 |  |
| 1:110203845:T | 1 | 4 |  |
| 1:110235888:A | 3 | 25 |  |
| 1:111783996:G | 10 | 27 | 50 |
| 1:112018657:A | 2 | 24 | 50 |
| 1:115236057:A | 1 | 14 | 50 |
| 1:11561101:G | 1 | 5 |  |
| 1:117452869:G | 1 | 31 | 50 |
| 1:120572547:C | 1 | 49 | 32 |
| 1:120611964:C | 10 | 20 | 25 |
| 1:120612006:A | 8 | 32 | 34 |
| 1:120612013:A | 1 | 29 | 26 |
| 1:120612014:A | 1 | 30 | 26 |
| 1:1243191:A | 1 | 16 | 49 |
| 1:12779560:C | 2 | 26 | 49 |
| 1:12779618:C | 2 | 25 | 49 |
| 1:12853415:G | 1 | 91 | 28 |
| 1:12853509:C | 2 | 66 | 32 |
| 1:12853544:G | 1 | 73 | 31 |
| 1:12854068:C | 5 | 86 | 28 |
| 1:12854090:A | 1 | 112 | 27 |
| 1:12854097:C | 1 | 110 | 28 |
| 1:12854105:G | 1 | 98 | 27 |
| 1:12854162:T | 9 | 49 | 28 |
| 1:12854188:C | 9 | 55 | 31 |
| 1:12854370:G | 7 | 127 | 29 |
| 1:12854401:T | 2 | 151 | 29 |
| 1:12855752:A | 8 | 144 | 31 |
| 1:12855774:G | 3 | 120 | 27 |
| 1:12855843:T | 1 | 20 | 11 |
| 1:12855844:A | 1 | 20 | 11 |
| 1:12855845:C | 1 | 22 | 11 |
| 1:12856010:G | 8 | 233 | 36 |
| 1:12887220:G | 3 | 53.5 |  |
| 1:12887221:T | 1 | 118.5 |  |
| 1:12887224:T | 1 | 122 |  |
| 1:12887226:T | 1 | 121 |  |
| 1:12887227:A | 1 | 122 |  |
| 1:12952917:T | 1 | 35 |  |
| 1:12952951:C | 2 | 11 |  |
| 1:12952966:A | 9 | 4 |  |
| 1:12952968:G | 1 | 2.5 |  |
| 1:12953166:T | 10 | 32 | 8 |
| 1:12954852:C | 1 | 20.5 |  |
| 1:12954967:T | 1 | 41 | 17 |
| 1:12979729:A | 1 | 186 | 22 |
| 1:12979845:C | 2 | 373 | 23 |
| 1:12980074:A | 5 | 192 | 12 |
| 1:12980127:G | 1 | 88 | 0 |
| 1:12980127:T | 9 | 88 | 0 |
| 1:13036330:A | 1 | 25 | 17 |
| 1:13036447:G | 7 | 45 | 3 |
| 1:13038119:C | 2 | 22 | 4 |
| 1:13038201:C | 12 | 121 | 5 |
| 1:13038202:A | 11 | 120 | 5 |
| 1:13038221:T | 13 | 151 | 6 |
| 1:13038327:T | 2 | 137 | 4 |
| 1:13052789:A | 3 | 19 | 43 |
| 1:13052871:T | 10 | 118 | 32 |
| 1:13052907:C | 7 | 98 | 27 |
| 1:13329095:G | 7 | 13 | 0 |
| 1:13329150:G | 1 | 15 | 0 |
| 1:13329337:G | 9 | 22 | 0 |
| 1:13329350:T | 9 | 18 | 0 |
| 1:13329354:C | 8 | 18 | 0 |
| 1:13365913:C | 1 | 59 | 5 |
| 1:13365924:A | 1 | 55 | 6 |
| 1:13448547:C | 6 | 122 | 2 |
| 1:13448548:A | 7 | 122 | 2 |
| 1:13448551:C | 3 | 125 | 3 |
| 1:13450014:G | 7 | 109 | 0 |
| 1:13474788:A | 1 | 115 |  |
| 1:13523852:C | 10 | 21 | 22 |
| 1:13670869:G | 9 | 107 | 0 |
| 1:13671653:C | 1 | 70 | 0 |
| 1:13695579:A | 7 | 64 |  |
| 1:13695580:G | 8 | 66 |  |
| 1:13695600:C | 5 | 36.5 |  |
| 1:13695603:C | 4 | 39 |  |
| 1:13695974:C | 9 | 17 | 7 |
| 1:13696090:C | 3 | 24 | 2 |
| 1:138928:G | 3 | 12 | 6 |
| 1:139296:A | 2 | 14 | 14 |
| 1:1423267:G | 3 | 29 | 50 |
| 1:1431165:T | 1 | 240 |  |
| 1:143767513:T | 5 | 112 | 39 |
| 1:143767522:G | 8 | 91 | 37 |
| 1:143767547:A | 8 | 88 | 33 |
| 1:143767643:C | 1 | 54 | 26 |
| 1:143906122:A | 4 | 55 | 6 |
| 1:144811805:G | 3 | 89 |  |
| 1:144811810:G | 4 | 84 | 10 |
| 1:144811823:C | 2 | 42 | 8 |
| 1:144811827:A | 1 | 35 | 5 |
| 1:144823868:G | 12 | 35 | 19 |
| 1:144828548:A | 1 | 24 | 12 |
| 1:144828784:G | 1 | 246 | 34 |
| 1:144855812:C | 4 | 52 | 47 |
| 1:144873887:T | 3 | 70 | 49 |
| 1:144874815:C | 2 | 130 | 49 |
| 1:144880832:C | 9 | 48 | 49 |
| 1:144882581:G | 4 | 137 | 49 |
| 1:144912233:T | 9 | 119 | 49 |
| 1:144916748:G | 1 | 122 | 49 |
| 1:144917546:G | 7 | 106 | 49 |
| 1:144917841:C | 5 | 173 | 43 |
| 1:144918957:A | 9 | 81 | 49 |
| 1:144990002:T | 2 | 33 | 0 |
| 1:1452615:T | 1 | 55 |  |
| 1:145273335:A | 6 | 225 | 43 |
| 1:145281543:T | 2 | 285 | 43 |
| 1:145293425:A | 1 | 713 | 44 |
| 1:145293490:C | 7 | 682 | 47 |
| 1:145293498:C | 9 | 645 | 47 |
| 1:145293510:G | 8 | 614 | 46 |
| 1:145296372:G | 6 | 160 | 41 |
| 1:145296403:T | 1 | 20 | 4 |
| 1:145299787:G | 5 | 81 | 10 |
| 1:145299792:G | 1 | 136 | 23 |
| 1:145299805:C | 2 | 159 | 25 |
| 1:145299809:A | 2 | 163 | 25 |
| 1:145301739:T | 2 | 18 | 24 |
| 1:145301793:G | 5 | 19 | 14 |
| 1:145301802:G | 2 | 14 | 19 |
| 1:145302704:G | 4 | 23 | 8 |
| 1:145311110:C | 2 | 27 | 36 |
| 1:145330829:G | 9 | 68 | 4 |
| 1:145338661:A | 1 | 55 | 5 |
| 1:145343385:A | 3 | 11 | 6 |
| 1:145348109:A | 10 | 13 | 17 |
| 1:145349628:C | 1 | 158 | 6 |
| 1:145349719:C | 3 | 56 | 5 |
| 1:145359110:C | 2 | 14 | 16 |
| 1:145366857:T | 2 | 46 | 38 |
| 1:145367719:A | 1 | 17 | 12 |
| 1:145586679:A | 1 | 1.5 |  |
| 1:146400186:T | 2 | 49.5 |  |
| 1:146400239:T | 1 | 15 |  |
| 1:146400242:C | 1 | 15 |  |
| 1:146400257:G | 1 | 17 |  |
| 1:146404787:A | 8 | 82.5 |  |
| 1:146406560:C | 9 | 20 | 16 |
| 1:146409969:C | 6 | 40 | 4 |
| 1:146414187:A | 2 | 25 | 4 |
| 1:146418342:T | 8 | 30 | 6 |
| 1:147595042:G | 2 | 60.5 |  |
| 1:147955126:G | 1 | 169 |  |
| 1:147955246:T | 2 | 121 |  |
| 1:147955256:G | 6 | 58.5 |  |
| 1:148010911:A | 2 | 102 | 19 |
| 1:148012531:T | 8 | 26 | 8 |
| 1:148015634:C | 8 | 22 | 23 |
| 1:148021591:C | 1 | 4 |  |
| 1:148022984:C | 1 | 99 | 11 |
| 1:148023067:T | 1 | 78 | 5 |
| 1:148025763:A | 2 | 25 | 16 |
| 1:148341812:A | 2 | 97 | 18 |
| 1:148343676:T | 1 | 19 | 8 |
| 1:148343679:T | 1 | 20 | 8 |
| 1:148343714:G | 9 | 13 | 19 |
| 1:148343777:A | 7 | 21 | 17 |
| 1:148344741:C | 4 | 11 | 6 |
| 1:148579636:C | 1 | 21 | 15 |
| 1:148741720:C | 8 | 31 | 17 |
| 1:148754858:C | 1 | 74 | 8 |
| 1:148756499:G | 7 | 10 | 6 |
| 1:148756515:C | 9 | 15 | 5 |
| 1:148756607:T | 2 | 49 | 7 |
| 1:148756648:T | 20 | 129 | 2 |
| 1:148756664:T | 5 | 117 | 2 |
| 1:148756665:T | 5 | 117 | 2 |
| 1:149885190:T | 1 | 19 |  |
| 1:150199042:A | 2 | 26 | 49 |
| 1:150199045:C | 2 | 26 | 49 |
| 1:150199051:C | 11 | 107 | 49 |
| 1:150280546:T | 10 | 17 | 49 |
| 1:151700081:T | 1 | 27 | 47 |
| 1:152129115:C | 1 | 109 | 46 |
| 1:152185815:A | 1 | 11.5 |  |
| 1:152185816:G | 1 | 12 |  |
| 1:152185823:T | 1 | 7.5 |  |
| 1:152186222:C | 2 | 80 | 8 |
| 1:152186422:G | 1 | 108 | 6 |
| 1:152186490:T | 1 | 115 |  |
| 1:152188940:G | 1 | 157 | 4 |
| 1:152189055:C | 2 | 57 | 3 |
| 1:152327325:A | 1 | 70.5 |  |
| 1:152327329:C | 1 | 178 |  |
| 1:152327425:G | 1 | 200 |  |
| 1:152327542:G | 1 | 1.5 |  |
| 1:152327810:A | 1 | 177.5 |  |
| 1:152327810:T | 1 | 185 |  |
| 1:152749003:C | 2 | 5 |  |
| 1:152749004:A | 2 | 3 |  |
| 1:152749042:G | 4 | 8.5 |  |
| 1:152882982:G | 1 | 195 | 40 |
| 1:152975812:A | 4 | 122 |  |
| 1:152975816:T | 3 | 135 |  |
| 1:153320372:G | 8 | 20 | 49 |
| 1:153320401:A | 3 | 22 | 50 |
| 1:153907287:C | 1 | 37 | 48 |
| 1:153907315:T | 1 | 55.5 |  |
| 1:155204797:A | 1 | 459 | 27 |
| 1:157489535:A | 2 | 14 | 49 |
| 1:157566136:A | 1 | 14 | 49 |
| 1:1575784:T | 2 | 197 |  |
| 1:1577093:T | 3 | 42 | 6 |
| 1:1580524:T | 7 | 107 | 3 |
| 1:159796701:A | 1 | 21 | 49 |
| 1:160209885:G | 1 | 15 |  |
| 1:160209892:C | 1 | 183 |  |
| 1:160580549:T | 1 | 28 | 50 |
| 1:161645052:T | 1 | 23 | 27 |
| 1:162367071:T | 4 | 25 | 50 |
| 1:162367103:T | 2 | 21 | 49 |
| 1:1643740:T | 1 | 102 | 6 |
| 1:1670958:G | 1 | 92 | 7 |
| 1:167893759:A | 6 | 24 | 49 |
| 1:16895634:G | 8 | 65 | 41 |
| 1:16899669:T | 5 | 14 | 29 |
| 1:16902884:C | 3 | 29 | 17 |
| 1:16902894:G | 3 | 13 | 15 |
| 1:16903912:A | 2 | 19 | 4 |
| 1:16905719:A | 1 | 229 | 32 |
| 1:16905837:A | 1 | 116 | 22 |
| 1:16909100:C | 2 | 28 | 0 |
| 1:16909129:C | 2 | 47 | 0 |
| 1:16909208:G | 2 | 81 | 1 |
| 1:16913677:C | 3 | 41 | 5 |
| 1:16914252:T | 1 | 104 | 7 |
| 1:16915434:C | 1 | 40 | 1 |
| 1:16915513:G | 3 | 86 | 0 |
| 1:17248532:G | 10 | 16 | 50 |
| 1:173808512:G | 1 | 11 |  |
| 1:175129946:T | 5 | 194 | 45 |
| 1:17658130:G | 15 | 126 | 48 |
| 1:178745947:G | 1 | 30 | 49 |
| 1:179457770:T | 1 | 20 | 47 |
| 1:179457796:C | 1 | 22 | 43 |
| 1:179876988:G | 9 | 13 | 49 |
| 1:183596703:A | 1 | 25 | 49 |
| 1:1850654:C | 9 | 93 |  |
| 1:1850678:C | 1 | 2 |  |
| 1:1991014:G | 5 | 27 | 50 |
| 1:200867552:T | 1 | 30 | 50 |
| 1:201179097:C | 1 | 18 | 18 |
| 1:201179120:G | 1 | 22 | 16 |
| 1:201179121:G | 1 | 22 | 16 |
| 1:201180054:C | 1 | 78.5 |  |
| 1:201356006:A | 1 | 145 | 50 |
| 1:201386916:T | 2 | 14 | 49 |
| 1:201924660:A | 1 | 18 | 49 |
| 1:20411332:T | 4 | 21 | 49 |
| 1:204159612:G | 1 | 4.5 |  |
| 1:20416322:A | 1 | 52 |  |
| 1:205632220:T | 2 | 4 |  |
| 1:205632253:G | 2 | 4 |  |
| 1:205889329:T | 1 | 24 | 49 |
| 1:206566904:C | 1 | 24 | 6 |
| 1:206578650:C | 2 | 25 | 0 |
| 1:206603535:A | 1 | 19 | 50 |
| 1:206669465:T | 4 | 14 | 49 |
| 1:206773659:G | 6 | 27 | 49 |
| 1:207700165:C | 1 | 13 | 13 |
| 1:207726161:T | 1 | 124 | 2 |
| 1:207795320:G | 11 | 20 | 48 |
| 1:210003489:G | 2 | 16 | 49 |
| 1:21012575:A | 1 | 27 | 50 |
| 1:210267899:T | 1 | 75.5 |  |
| 1:214564340:C | 2 | 32 | 49 |
| 1:21806619:C | 1 | 13 | 7 |
| 1:21806624:G | 1 | 14 | 7 |
| 1:21807427:T | 2 | 17 | 10 |
| 1:21809014:C | 3 | 50 | 2 |
| 1:21945530:T | 1 | 27 | 50 |
| 1:21976289:T | 1 | 21 | 50 |
| 1:220603324:G | 3 | 34 | 49 |
| 1:22332008:C | 5 | 111.5 |  |
| 1:2255569:T | 1 | 34 | 49 |
| 1:226788371:A | 1 | 3 |  |
| 1:226924884:A | 12 | 30 | 48 |
| 1:235652513:C | 10 | 11 | 50 |
| 1:236557771:A | 4 | 29 | 50 |
| 1:240370932:A | 4 | 69 | 47 |
| 1:240370935:C | 2 | 65 | 46 |
| 1:240370952:T | 2 | 98 | 45 |
| 1:24393563:T | 1 | 29 | 49 |
| 1:244228389:A | 1 | 12 |  |
| 1:244999023:T | 1 | 20 | 49 |
| 1:24671406:A | 1 | 17 | 49 |
| 1:246720798:A | 10 | 21 | 49 |
| 1:247978544:A | 5 | 14 | 50 |
| 1:248084747:C | 2 | 11 |  |
| 1:248084749:A | 2 | 7 |  |
| 1:248084754:A | 2 | 8 |  |
| 1:248084756:C | 4 | 8 |  |
| 1:248084757:C | 4 | 10 |  |
| 1:248524992:G | 2 | 22 | 32 |
| 1:248525329:T | 12 | 134.5 |  |
| 1:248525330:G | 15 | 143 | 45 |
| 1:248604542:T | 1 | 26 | 7 |
| 1:248604943:A | 2 | 190 |  |
| 1:248605124:T | 1 | 314 | 36 |
| 1:248605374:A | 1 | 26 | 22 |
| 1:248637115:C | 1 | 253 | 17 |
| 1:248637256:T | 1 | 6.5 |  |
| 1:248637262:C | 1 | 5 |  |
| 1:248652058:A | 2 | 215 | 6 |
| 1:248652061:T | 1 | 218 | 6 |
| 1:248722611:G | 3 | 165 | 2 |
| 1:248722659:T | 1 | 94 | 0 |
| 1:248722671:A | 1 | 49.5 |  |
| 1:248722722:C | 1 | 41 | 0 |
| 1:248722723:A | 1 | 43 |  |
| 1:248737230:C | 1 | 30 | 5 |
| 1:248737343:T | 1 | 23 | 8 |
| 1:248737348:A | 1 | 33 | 11 |
| 1:248737454:G | 3 | 320.5 |  |
| 1:248801610:A | 1 | 33 |  |
| 1:248801778:T | 1 | 210 | 11 |
| 1:248801950:G | 1 | 14 | 37 |
| 1:248802469:T | 4 | 33 | 21 |
| 1:248813473:C,G | 1 | 163 |  |
| 1:248813653:C | 2 | 18 |  |
| 1:248814126:A | 8 | 170 |  |
| 1:25747230:C | 4 | 15 | 8 |
| 1:26608814:G | 4 | 21 | 46 |
| 1:26608828:A | 1 | 22 | 43 |
| 1:26608843:A | 1 | 74 | 41 |
| 1:26608867:A | 1 | 13 | 48 |
| 1:26608879:T | 4 | 24 | 47 |
| 1:26608883:G | 1 | 11 | 50 |
| 1:26608885:T | 4 | 34 | 40 |
| 1:26608889:T | 1 | 23 | 41 |
| 1:26628196:G | 1 | 18 | 50 |
| 1:28286610:C | 1 | 7 |  |
| 1:33777672:C | 1 | 2.5 |  |
| 1:36235455:T | 2 | 15 | 50 |
| 1:36643703:G | 3 | 7 |  |
| 1:39977575:T | 1 | 107 | 2 |
| 1:40236815:G | 1 | 29 | 21 |
| 1:46774783:G | 4 | 17 | 50 |
| 1:50884784:A | 1 | 162 | 48 |
| 1:52306066:A | 11 | 27 | 50 |
| 1:52499093:T | 2 | 4 |  |
| 1:53681699:G | 1 | 24 | 50 |
| 1:53712727:T | 5 | 24 | 49 |
| 1:55014013:T | 2 | 17 | 49 |
| 1:6529203:T | 1 | 148 | 47 |
| 1:67131957:G | 1 | 5 |  |
| 1:75037864:T | 4 | 167 |  |
| 1:7890026:G | 2 | 74 | 46 |
| 1:7890053:A | 1 | 103 | 45 |
| 1:7890055:A | 1 | 102 | 46 |
| 1:7909738:C | 1 | 27 | 49 |
| 1:85742012:C | 6 | 32 | 49 |
| 1:86375654:G | 11 | 21 | 49 |
| 1:86453307:C | 1 | 19 | 49 |
| 1:86512536:T | 2 | 26 | 50 |
| 1:86557967:A | 5 | 32 | 49 |
| 1:90309204:G | 1 | 12 | 49 |
| 1:92163682:G | 1 | 21 | 50 |
| 1:92457843:T | 11 | 21 | 49 |
| 1:98348885:A | 10 | 16 | 49 |
| 20:13976448:T | 2 | 30 | 49 |
| 20:170263:T | 1 | 25 | 49 |
| 20:18167977:C | 10 | 22 | 50 |
| 20:1896052:G | 1 | 3 |  |
| 20:1896052:T | 4 | 6 |  |
| 20:29623215:A | 3 | 24 | 34 |
| 20:29623223:C | 4 | 25 | 35 |
| 20:29628261:C | 1 | 29 | 19 |
| 20:29628271:T | 1 | 22 | 20 |
| 20:2996497:T | 7 | 29 | 49 |
| 20:30452782:T | 6 | 24 | 49 |
| 20:31768363:T | 1 | 20 | 49 |
| 20:31892670:A | 1 | 21 | 50 |
| 20:31897554:C | 1 | 29 | 49 |
| 20:31981849:C | 9 | 18 | 50 |
| 20:33320379:C | 1 | 13 | 50 |
| 20:33734991:T | 1 | 23 | 39 |
| 20:34084415:C | 2 | 25 | 49 |
| 20:3649640:A | 5 | 3.5 |  |
| 20:36946848:A | 2 | 24 | 50 |
| 20:36977970:A | 10 | 23 | 50 |
| 20:37001761:C | 1 | 36 | 49 |
| 20:37667182:T | 4 | 24 | 49 |
| 20:42939750:C | 8 | 24 | 49 |
| 20:43280227:T | 3 | 14 | 50 |
| 20:43976991:G | 1 | 26 | 49 |
| 20:45179196:A | 2 | 17 | 50 |
| 20:52611552:A | 1 | 32 | 50 |
| 20:60639800:A | 1 | 6 |  |
| 20:61463522:A | 5 | 21 | 49 |
| 20:61885767:T | 1 | 25 | 50 |
| 21:10942928:A | 1 | 162 | 50 |
| 21:10959771:T | 1 | 29 | 48 |
| 21:19751604:G | 1 | 14 | 49 |
| 21:30250555:C | 10 | 18 | 49 |
| 21:30250615:C | 10 | 22 | 49 |
| 21:30257568:C | 10 | 19 | 50 |
| 21:30458220:T | 1 | 32 | 49 |
| 21:31655212:C | 10 | 28 | 50 |
| 21:34614250:C | 4 | 22 | 49 |
| 21:34614255:G | 6 | 25 | 49 |
| 21:35467645:G | 4 | 27 | 49 |
| 21:35469193:A | 6 | 27 | 50 |
| 21:36042462:A | 4 | 102 | 40 |
| 21:36042475:A | 1 | 81 | 43 |
| 21:36042476:A | 1 | 84 | 43 |
| 21:36042478:A | 3 | 74 | 43 |
| 21:36042478:A,G | 2 | 74 | 43 |
| 21:37692589:T | 5 | 32 | 49 |
| 21:40584598:T | 11 | 29 | 49 |
| 21:42717662:C | 1 | 35 | 50 |
| 21:42879909:A | 5 | 20 | 49 |
| 21:43169357:G | 2 | 22 | 50 |
| 21:43411554:A | 3 | 74 |  |
| 21:43985955:A | 8 | 22 | 50 |
| 21:43985958:T | 2 | 26 | 50 |
| 21:44589215:T | 9 | 98 | 49 |
| 21:45538647:C | 9 | 31 | 49 |
| 21:45553596:C | 7 | 312 | 49 |
| 21:45588136:A | 1 | 121 | 35 |
| 21:45588146:A | 2 | 133 | 34 |
| 21:45970771:T | 5 | 11 |  |
| 21:45970772:G | 6 | 5.5 |  |
| 21:45970774:A | 6 | 8 |  |
| 21:46012240:G | 1 | 48 | 41 |
| 21:46020527:T | 10 | 22 | 49 |
| 21:46057619:C | 1 | 24 | 36 |
| 21:46057620:A | 1 | 23 | 36 |
| 21:46057621:A | 1 | 25 | 36 |
| 21:46057625:C | 1 | 23 | 36 |
| 21:46521023:G | 1 | 15 | 49 |
| 21:46534729:C | 1 | 5.5 |  |
| 21:46908355:C | 5 | 16 | 49 |
| 21:46924425:A | 1 | 20 | 36 |
| 21:47351605:C | 5 | 150 |  |
| 21:47351607:G | 5 | 151.5 |  |
| 21:47351612:G | 7 | 98 | 46 |
| 21:47351616:C | 1 | 120 | 45 |
| 22:17264565:T | 1 | 11 |  |
| 22:17265124:C | 6 | 38 | 4 |
| 22:17445698:A | 1 | 114 | 50 |
| 22:18727099:T | 1 | 10 | 25 |
| 22:18727100:C | 1 | 10 | 25 |
| 22:18727115:T | 1 | 18 | 9 |
| 22:18835221:G | 3 | 79 | 41 |
| 22:18835365:G | 1 | 12 | 13 |
| 22:18835523:C | 3 | 26 | 4 |
| 22:19420109:C | 6 | 27 | 49 |
| 22:20367781:C | 3 | 89 | 2 |
| 22:20456851:T | 3 | 139 | 4 |
| 22:20458029:C | 1 | 70 | 7 |
| 22:20708961:T | 1 | 66 | 40 |
| 22:20708972:T | 8 | 62 | 43 |
| 22:20708977:T | 6 | 110 | 46 |
| 22:20708980:A | 4 | 113 | 46 |
| 22:20709228:T | 1 | 15 | 37 |
| 22:20709231:G | 2 | 13 | 36 |
| 22:20709237:C | 2 | 11 | 34 |
| 22:20709238:C | 2 | 11 | 34 |
| 22:20709242:T | 2 | 11 | 34 |
| 22:20709282:A | 1 | 37 | 28 |
| 22:20709292:G | 2 | 28 | 33 |
| 22:20709296:C | 2 | 24 | 33 |
| 22:20709304:A | 2 | 18 | 34 |
| 22:20709307:T | 1 | 12 | 34 |
| 22:20709312:G | 2 | 15 | 36 |
| 22:20709318:T | 1 | 42 | 40 |
| 22:20710802:C | 1 | 108 | 37 |
| 22:20710820:T | 2 | 107 | 35 |
| 22:20710847:T | 2 | 85 | 34 |
| 22:20710850:T | 3 | 98 | 36 |
| 22:20780031:C | 12 | 139 | 49 |
| 22:21481188:C | 3 | 76 | 1 |
| 22:21481271:C | 1 | 46 | 11 |
| 22:21570790:G | 1 | 27 | 9 |
| 22:21570832:G | 1 | 15 | 11 |
| 22:21579717:C | 2 | 12 | 27 |
| 22:21741331:C | 3 | 137 |  |
| 22:21742684:T | 15 | 177 | 1 |
| 22:22550760:G | 2 | 24 | 49 |
| 22:22707299:C | 8 | 24 | 38 |
| 22:22707309:T | 7 | 23 | 42 |
| 22:22707638:G | 1 | 42 | 27 |
| 22:22707639:A | 1 | 43 | 27 |
| 22:22786753:T | 1 | 10 |  |
| 22:22899234:G | 8 | 22 | 49 |
| 22:23047144:T | 1 | 127 | 50 |
| 22:23089943:A | 1 | 3 |  |
| 22:23247082:T | 10 | 22 | 46 |
| 22:23248549:A | 7 | 27 | 11 |
| 22:23263602:T | 11 | 24 | 49 |
| 22:24199704:T | 2 | 16 | 50 |
| 22:24300059:A | 2 | 118.5 |  |
| 22:24300102:G | 3 | 199.5 |  |
| 22:24300634:T | 14 | 97 | 3 |
| 22:24325095:G | 10 | 165 |  |
| 22:24459438:C | 3 | 19 | 50 |
| 22:24917980:A | 1 | 24 | 49 |
| 22:25023506:A | 3 | 199 | 36 |
| 22:25023893:A | 4 | 243 | 40 |
| 22:25023915:G | 2 | 98.5 |  |
| 22:26157068:G | 1 | 26 | 49 |
| 22:26862212:C | 11 | 31 | 49 |
| 22:26879967:G | 6 | 39 | 44 |
| 22:29885594:T | 2 | 138 | 45 |
| 22:30793137:A | 6 | 26 | 50 |
| 22:32205632:C | 1 | 21 | 50 |
| 22:36598049:G | 7 | 30 | 49 |
| 22:36598058:C | 7 | 28 | 49 |
| 22:36598081:T | 1 | 16 | 49 |
| 22:37964413:A | 1 | 55 | 48 |
| 22:37964413:A,C | 8 | 54 | 46 |
| 22:37964413:C,A | 8 | 54 | 46 |
| 22:37964419:C | 6 | 64 | 46 |
| 22:38506509:G | 11 | 26 | 49 |
| 22:42525132:C | 1 | 760 | 45 |
| 22:42911257:G | 5 | 48 | 4 |
| 22:43193603:A | 2 | 11 | 50 |
| 22:44324727:G | 3 | 24 | 49 |
| 22:44324730:T | 3 | 24 | 49 |
| 22:44379838:G | 1 | 32 | 49 |
| 22:45789642:C | 8 | 32 | 49 |
| 22:50921164:C | 5 | 7.5 |  |
| 2:100343557:T | 10 | 23 | 49 |
| 2:107049714:G | 2 | 236 |  |
| 2:108455303:T | 6 | 6.5 |  |
| 2:108479165:C | 1 | 67 |  |
| 2:108479214:G | 2 | 24 |  |
| 2:109297180:A | 1 | 29 | 26 |
| 2:111598958:T | 6 | 26 | 50 |
| 2:112536264:T | 7 | 18 | 5 |
| 2:112551673:A | 2 | 76 | 10 |
| 2:113127773:G | 1 | 19 | 23 |
| 2:113138479:A | 2 | 77.5 |  |
| 2:113258916:T | 3 | 28 | 22 |
| 2:113671410:G | 1 | 25 | 49 |
| 2:113830347:G | 1 | 16 | 50 |
| 2:11853913:A | 1 | 16 | 49 |
| 2:118575161:C | 1 | 180 |  |
| 2:118771566:A | 6 | 20 | 49 |
| 2:120078778:C | 1 | 19 | 50 |
| 2:120199140:G | 11 | 14 | 50 |
| 2:121981950:G | 9 | 18 | 50 |
| 2:130832256:G | 1 | 145 | 17 |
| 2:130832292:A | 1 | 102 | 21 |
| 2:130832873:A | 1 | 244 | 11 |
| 2:130832927:G | 1 | 155 | 16 |
| 2:130869609:G | 1 | 25 | 20 |
| 2:131129933:C | 1 | 8.5 |  |
| 2:131129934:A | 1 | 9.5 |  |
| 2:131129948:G | 1 | 72 |  |
| 2:131221241:A | 1 | 271 |  |
| 2:131221499:A | 1 | 71 | 4 |
| 2:131221668:A | 2 | 18 |  |
| 2:131245713:G | 5 | 36 | 8 |
| 2:131377707:G | 16 | 22 | 5 |
| 2:131414737:G | 1 | 221 | 18 |
| 2:132019302:T | 1 | 35 | 8 |
| 2:132021629:T | 1 | 74 | 3 |
| 2:132021781:T | 5 | 283 | 22 |
| 2:132021815:A,C | 2 | 266 |  |
| 2:132021815:C | 7 | 279 |  |
| 2:132289349:G | 7 | 23 | 13 |
| 2:135749123:G | 1 | 16 | 50 |
| 2:153417441:T | 1 | 16 | 48 |
| 2:159663599:C | 1 | 26 | 49 |
| 2:159663616:A | 1 | 34 | 49 |
| 2:160136337:A | 10 | 20 | 50 |
| 2:161174693:T | 1 | 24 | 50 |
| 2:164467320:G | 1 | 70 | 50 |
| 2:165697678:C | 1 | 24 | 49 |
| 2:166032778:C | 3 | 10 | 50 |
| 2:171649402:T | 1 | 27 | 49 |
| 2:171678625:C | 1 | 24 | 49 |
| 2:175202208:A | 1 | 16 | 34 |
| 2:175292593:T | 1 | 9 |  |
| 2:176995288:A | 1 | 26 | 43 |
| 2:179621477:T | 6 | 17 | 50 |
| 2:17962998:A | 1 | 5 |  |
| 2:187559047:A | 5 | 158 | 48 |
| 2:187559050:G | 5 | 160 | 48 |
| 2:187559053:G | 3 | 166 | 48 |
| 2:188250301:A | 12 | 30 | 49 |
| 2:189940142:G | 3 | 28 | 50 |
| 2:191184475:G | 6 | 14 | 49 |
| 2:201526330:G | 5 | 29 | 49 |
| 2:207603221:C | 1 | 22 | 48 |
| 2:207603234:G | 3 | 24 | 49 |
| 2:209214770:G | 3 | 23 | 49 |
| 2:21235475:C | 10 | 12 | 49 |
| 2:21266782:A | 1 | 13 |  |
| 2:21266783:G | 1 | 35 |  |
| 2:217288388:C | 1 | 20 | 50 |
| 2:227662524:A | 1 | 7.5 |  |
| 2:227662524:A,C | 1 | 11 |  |
| 2:227662524:C | 1 | 9 |  |
| 2:227662524:C,A | 1 | 5 |  |
| 2:228102723:C | 2 | 32 | 49 |
| 2:228111435:C | 9 | 31 | 50 |
| 2:228194480:T | 9 | 21 | 49 |
| 2:228194481:T | 9 | 21 | 49 |
| 2:228217260:A | 1 | 29 | 49 |
| 2:231258150:T | 7 | 23 | 49 |
| 2:232325435:T | 1 | 182 | 50 |
| 2:233712227:G | 5 | 33 | 49 |
| 2:233712229:C | 1 | 10 |  |
| 2:234761225:T | 2 | 22 | 50 |
| 2:238258814:G | 2 | 32 | 49 |
| 2:238499866:T | 5 | 63 | 50 |
| 2:238999883:C | 3 | 20 | 49 |
| 2:240684619:A | 1 | 22 | 50 |
| 2:240946766:C | 4 | 27 | 50 |
| 2:240981513:C | 6 | 109 |  |
| 2:240981519:G | 1 | 45 |  |
| 2:240981520:G | 2 | 46 |  |
| 2:240981527:A | 4 | 113 |  |
| 2:240981555:A | 6 | 75 |  |
| 2:240981556:T | 4 | 85 |  |
| 2:240981571:G | 1 | 77 |  |
| 2:240981940:C | 2 | 48 |  |
| 2:240981968:C | 2 | 103 |  |
| 2:240982200:T | 1 | 138 |  |
| 2:240982219:G | 2 | 166 |  |
| 2:240982227:A | 2 | 168 |  |
| 2:240982285:G | 1 | 81.5 |  |
| 2:240982379:C | 1 | 92 |  |
| 2:241624543:T | 1 | 28 | 11 |
| 2:241624544:G | 1 | 27 | 11 |
| 2:242035491:G | 1 | 9.5 |  |
| 2:242163366:G | 3 | 12.5 |  |
| 2:242163367:T | 2 | 16 |  |
| 2:242716380:T | 5 | 25 | 50 |
| 2:24390517:A | 11 | 25 | 49 |
| 2:26804247:C | 7 | 14 | 50 |
| 2:27804371:G | 1 | 57 | 46 |
| 2:27804381:G | 1 | 54 | 46 |
| 2:40488155:T | 1 | 16 | 50 |
| 2:46707884:G | 1 | 103 | 49 |
| 2:46707885:G | 1 | 101 | 49 |
| 2:47045321:G | 1 | 34 | 49 |
| 2:54482716:A | 10 | 214 | 47 |
| 2:54558114:C | 1 | 18 | 49 |
| 2:55887325:T | 1 | 30 | 50 |
| 2:61413592:T | 2 | 16 |  |
| 2:68794483:A | 1 | 27 | 50 |
| 2:70129800:T | 4 | 61 | 44 |
| 2:70677994:A | 5 | 15 | 50 |
| 2:71170807:T | 1 | 27 | 49 |
| 2:85549868:G | 4 | 33 | 49 |
| 2:85549874:G | 2 | 37 | 49 |
| 2:87088964:G | 5 | 25 | 43 |
| 2:87243501:C | 1 | 29.5 |  |
| 2:88081797:T | 1 | 142 | 4 |
| 2:89161069:C | 10 | 14 | 49 |
| 2:89161072:C | 10 | 13 | 49 |
| 2:89417017:G | 3 | 7 |  |
| 2:89417024:A | 2 | 1.5 |  |
| 2:89417028:G | 1 | 1.5 |  |
| 2:89417043:C | 3 | 2 |  |
| 2:89417049:A | 5 | 6 |  |
| 2:89891207:A | 1 | 197 | 8 |
| 2:90121845:G | 1 | 359 |  |
| 2:90121985:G | 2 | 333 |  |
| 2:90193417:G | 9 | 19 | 14 |
| 2:90260246:A | 1 | 43 | 47 |
| 2:90260247:A | 1 | 43 | 47 |
| 2:95847047:A | 19 | 198 | 50 |
| 2:96780986:T | 10 | 192 | 45 |
| 2:97877470:A | 1 | 11 |  |
| 2:97915895:C | 8 | 47 |  |
| 2:97915896:A | 8 | 49 |  |
| 2:9983686:T | 6 | 11 | 49 |
| 3:108557797:T | 1 | 21 | 50 |
| 3:111356083:C | 1 | 29 | 49 |
| 3:111603970:C | 1 | 7 |  |
| 3:113222036:A | 3 | 28 | 50 |
| 3:113376119:T | 1 | 50 | 48 |
| 3:11596302:C | 8 | 25 | 49 |
| 3:118865132:C | 3 | 105 |  |
| 3:121351315:A | 5 | 163 | 45 |
| 3:121361787:A | 2 | 27 | 49 |
| 3:121396174:G | 1 | 10 |  |
| 3:122103120:A | 2 | 18 | 49 |
| 3:123458811:C | 1 | 20 | 50 |
| 3:123458823:A | 1 | 20 | 49 |
| 3:124482503:T | 3 | 4 |  |
| 3:125725272:T | 2 | 7.5 |  |
| 3:126291219:G | 1 | 12 |  |
| 3:12783984:A | 1 | 24 | 50 |
| 3:130122493:G | 1 | 18 | 50 |
| 3:130318621:A | 1 | 15 | 50 |
| 3:130360527:T | 1 | 34 | 50 |
| 3:137981393:T | 1 | 26 | 49 |
| 3:138347988:C | 1 | 27 | 49 |
| 3:140675438:A | 1 | 10 |  |
| 3:14105843:T | 1 | 135 | 46 |
| 3:14105893:C | 1 | 68 | 46 |
| 3:14105894:A | 3 | 53 | 46 |
| 3:14105896:A | 1 | 63 | 45 |
| 3:14105897:A,G | 3 | 51 | 49 |
| 3:14105897:G,A | 3 | 51 | 49 |
| 3:14106033:C | 3 | 22 | 49 |
| 3:14106037:C | 3 | 23 | 49 |
| 3:14106354:T | 3 | 161 | 46 |
| 3:150421543:T | 1 | 124 | 49 |
| 3:15637914:A | 1 | 21 | 49 |
| 3:158388780:C | 1 | 29 | 49 |
| 3:167742358:G | 1 | 125 | 50 |
| 3:170824990:A | 3 | 12 | 50 |
| 3:183465483:A | 1 | 22 | 49 |
| 3:185198305:G | 1 | 7 |  |
| 3:190106073:C | 3 | 196 |  |
| 3:195447919:G | 3 | 16 |  |
| 3:195452870:A | 1 | 12 | 15 |
| 3:195452872:C | 1 | 12 | 15 |
| 3:195452873:A | 1 | 13 | 15 |
| 3:195452951:C | 1 | 871 | 48 |
| 3:195453064:G | 9 | 1037 | 48 |
| 3:195501149:T | 8 | 263 | 49 |
| 3:195505247:T | 1 | 36 | 5 |
| 3:195506323:C | 3 | 69 |  |
| 3:195506548:C | 1 | 23 |  |
| 3:195506555:T | 1 | 23 |  |
| 3:195506558:C | 1 | 23 |  |
| 3:195506560:T | 1 | 26 |  |
| 3:195506740:C | 2 | 51 | 38 |
| 3:195506788:C | 1 | 25 | 34 |
| 3:195507756:G | 1 | 9 |  |
| 3:195507769:A | 1 | 1.5 |  |
| 3:195507798:C | 1 | 1.5 |  |
| 3:195507846:G | 1 | 2 |  |
| 3:195507858:T | 5 | 8 |  |
| 3:195511142:C | 1 | 404 | 37 |
| 3:195511451:C | 1 | 168 | 43 |
| 3:195511956:T | 2 | 114 |  |
| 3:195512948:A | 1 | 71 | 27 |
| 3:195512949:A | 1 | 68 | 27 |
| 3:195512965:A | 1 | 56 | 27 |
| 3:195512965:A,C | 1 | 56 | 27 |
| 3:195512965:C,A | 1 | 56 | 27 |
| 3:195513345:C | 2 | 403 | 40 |
| 3:195515386:A | 1 | 65 | 30 |
| 3:195515388:A | 1 | 56 | 30 |
| 3:195515401:G | 1 | 211 | 35 |
| 3:195515403:C | 1 | 215 | 35 |
| 3:195515411:C | 1 | 215 | 36 |
| 3:195515411:T | 3 | 177 | 37 |
| 3:195515413:T | 3 | 174 | 37 |
| 3:195515414:C | 2 | 175 | 37 |
| 3:195515449:T | 1 | 173 | 39 |
| 3:195515459:T | 1 | 142 | 39 |
| 3:195515460:G | 1 | 144 | 39 |
| 3:197579466:T | 1 | 36 | 49 |
| 3:28533658:A | 2 | 26 | 49 |
| 3:33134710:A | 2 | 10 |  |
| 3:42733387:A | 4 | 71 |  |
| 3:43732523:T | 1 | 27 | 49 |
| 3:4767262:C | 10 | 18 | 49 |
| 3:48282695:G | 4 | 12 | 49 |
| 3:57261947:C | 1 | 27 | 50 |
| 3:58625875:A | 5 | 18 | 49 |
| 3:59997112:A | 2 | 20 | 49 |
| 3:66396808:C | 1 | 20 | 46 |
| 3:66419942:G | 1 | 27 | 49 |
| 3:66419956:T | 1 | 28 | 49 |
| 3:87276699:T | 5 | 17 | 49 |
| 3:97983494:T | 2 | 8 |  |
| 3:97983497:A | 5 | 172 |  |
| 3:98520461:A | 1 | 18 | 50 |
| 3:9920138:C | 4 | 20 | 49 |
| 4:100045616:T | 11 | 17 | 49 |
| 4:100140306:T | 11 | 10 | 50 |
| 4:101108877:C | 2 | 132 |  |
| 4:108641300:C | 6 | 31 | 49 |
| 4:108641317:A | 2 | 22 | 49 |
| 4:1090625:A | 1 | 9 | 50 |
| 4:109745336:G | 1 | 30 | 50 |
| 4:109769942:C | 1 | 14 | 49 |
| 4:109841743:T | 3 | 18 | 49 |
| 4:110678925:C | 11 | 22 | 49 |
| 4:122078317:C | 1 | 85.5 |  |
| 4:1388378:C | 4 | 90.5 |  |
| 4:1388379:A | 4 | 87 |  |
| 4:1388563:G | 1 | 12 |  |
| 4:1388564:C | 1 | 11 |  |
| 4:1388724:G | 1 | 87.5 |  |
| 4:140811075:T | 1 | 102 | 47 |
| 4:140811108:T | 1 | 141 | 46 |
| 4:140811111:T | 3 | 157 | 48 |
| 4:144801641:A | 1 | 97 | 46 |
| 4:144920596:A | 7 | 28 | 49 |
| 4:145041686:G | 5 | 15 | 38 |
| 4:145041707:A | 2 | 17 | 26 |
| 4:145041708:C | 2 | 17 | 25 |
| 4:145041720:G | 4 | 21 | 35 |
| 4:151719263:G | 2 | 37 | 50 |
| 4:155295076:G | 7 | 18 | 48 |
| 4:156787340:A | 10 | 33 | 49 |
| 4:156787368:A | 1 | 22 | 50 |
| 4:15689018:G | 2 | 9 |  |
| 4:15689018:G,T | 1 | 7.5 |  |
| 4:15689018:T | 1 | 14.5 |  |
| 4:15689018:T,G | 1 | 13.5 |  |
| 4:157557726:G | 9 | 29 | 50 |
| 4:158238830:C | 8 | 33 | 49 |
| 4:170678993:C | 6 | 24 | 50 |
| 4:175688141:A | 1 | 26 | 50 |
| 4:175899088:T | 1 | 151 | 45 |
| 4:175899091:T | 1 | 149 | 45 |
| 4:175899102:C | 2 | 133 | 46 |
| 4:177106011:A | 3 | 3 |  |
| 4:177106013:T | 3 | 5 |  |
| 4:184367561:T | 9 | 7 |  |
| 4:185615700:A | 2 | 7 |  |
| 4:185655190:G | 2 | 21 | 49 |
| 4:185655192:C | 3 | 26 | 50 |
| 4:2176454:A | 4 | 28 | 49 |
| 4:25265395:T | 1 | 11.5 |  |
| 4:265333:T | 1 | 45 | 38 |
| 4:265333:T,C | 1 | 45 | 38 |
| 4:26673787:G | 11 | 28 | 49 |
| 4:3076665:C | 2 | 163 | 49 |
| 4:3076672:G | 1 | 188 | 45 |
| 4:3230413:A | 1 | 22 |  |
| 4:36340753:A | 2 | 21 | 49 |
| 4:36340755:A | 3 | 22 | 49 |
| 4:3769307:A | 1 | 8 | 50 |
| 4:41673604:C | 7 | 27 | 50 |
| 4:4198950:C | 3 | 7 |  |
| 4:4198954:G | 1 | 7 |  |
| 4:42020142:G | 1 | 28 | 49 |
| 4:4276132:C | 1 | 26 | 50 |
| 4:466365:C | 1 | 21 | 46 |
| 4:47839929:T | 2 | 32 | 49 |
| 4:48165757:A | 1 | 22 | 50 |
| 4:48988450:A | 4 | 30 | 49 |
| 4:56874517:C | 2 | 30 | 49 |
| 4:57361553:G | 2 | 18 | 50 |
| 4:6693798:A | 2 | 5.5 |  |
| 4:68442968:G | 1 | 43 | 49 |
| 4:68829109:T | 4 | 9 | 49 |
| 4:68995529:T | 6 | 15 | 50 |
| 4:70898903:A | 1 | 31 | 49 |
| 4:70898905:C | 1 | 12 | 50 |
| 4:71468348:T | 5 | 8 |  |
| 4:72669661:A | 1 | 15 | 50 |
| 4:74124077:G | 1 | 60 | 43 |
| 4:7765495:T | 2 | 17 | 50 |
| 4:78987157:G | 7 | 21 | 49 |
| 4:80905990:G | 8 | 18 | 50 |
| 4:80905991:T | 1 | 21 | 50 |
| 4:81529518:C | 11 | 20 | 49 |
| 4:88535818:C | 1 | 142 | 47 |
| 4:88535830:C | 4 | 124 | 47 |
| 4:88535831:A | 4 | 124 | 47 |
| 4:88535832:G | 4 | 124 | 47 |
| 4:88536321:A | 2 | 149 |  |
| 4:88536457:C | 6 | 167 |  |
| 4:88536460:T | 2 | 65.5 |  |
| 4:88536550:C | 3 | 10 |  |
| 4:88536551:A | 4 | 20 |  |
| 4:88536553:T | 1 | 140 |  |
| 4:88536886:T | 3 | 101 | 48 |
| 4:88536917:A | 1 | 92 | 41 |
| 4:88536919:C | 1 | 94 | 41 |
| 4:88537060:C | 1 | 125 |  |
| 4:88537072:C | 2 | 127 | 41 |
| 4:88537078:C | 1 | 29 | 32 |
| 4:88537088:G | 1 | 45 | 31 |
| 4:88537114:T | 2 | 11 |  |
| 4:88537117:T | 2 | 15 |  |
| 4:88537123:T | 1 | 10 |  |
| 4:88537132:T | 4 | 29 |  |
| 4:88537135:A | 1 | 7 |  |
| 4:88537135:C | 2 | 4.5 |  |
| 4:88537141:C | 1 | 11.5 |  |
| 4:88537150:T | 4 | 8.5 |  |
| 4:88537153:C | 3 | 29 |  |
| 4:88537162:A | 2 | 54 |  |
| 4:88537162:A,T | 1 | 20.5 |  |
| 4:88537168:C | 2 | 56 |  |
| 4:88537285:T | 4 | 235 |  |
| 4:88537297:A | 1 | 221 |  |
| 4:88537312:T | 1 | 217.5 |  |
| 4:88537315:T | 1 | 82.5 |  |
| 4:88537411:T | 1 | 128 | 44 |
| 4:9217234:G | 1 | 112 | 4 |
| 4:9246121:G | 1 | 141 | 5 |
| 4:9250430:A | 1 | 84 | 3 |
| 4:9250440:G | 1 | 94 | 3 |
| 4:9274248:C | 2 | 137 | 8 |
| 4:9388088:T | 1 | 45 | 10 |
| 4:9390708:T | 1 | 10 | 45 |
| 5:10235368:T | 1 | 41 | 49 |
| 5:109190938:T | 7 | 5.5 |  |
| 5:110079450:C | 1 | 31 | 49 |
| 5:111756111:C | 1 | 4 |  |
| 5:112312676:C | 2 | 37 | 49 |
| 5:113829121:G | 10 | 19 | 50 |
| 5:115141151:T | 1 | 13 | 49 |
| 5:115341638:C | 6 | 24 | 49 |
| 5:118485784:T | 1 | 137 | 50 |
| 5:118691795:A | 1 | 26 | 49 |
| 5:122165317:T | 1 | 30 | 49 |
| 5:134907552:A | 1 | 17 | 49 |
| 5:135178124:C | 11 | 23 | 50 |
| 5:135178142:G | 4 | 23 | 49 |
| 5:137216525:A | 1 | 24 | 49 |
| 5:13944512:C | 7 | 24 | 49 |
| 5:140186979:A | 3 | 6.5 |  |
| 5:140186980:A | 3 | 7.5 |  |
| 5:140186982:A | 2 | 5 |  |
| 5:140186984:A | 3 | 6.5 |  |
| 5:140186986:C | 3 | 6.5 |  |
| 5:140186988:G | 3 | 6.5 |  |
| 5:140186990:A | 3 | 9.5 |  |
| 5:140558314:C | 3 | 24 | 33 |
| 5:140558315:C | 3 | 23 | 33 |
| 5:140558317:G | 2 | 25 | 34 |
| 5:140563763:A | 10 | 21 | 28 |
| 5:140573991:C | 2 | 51 | 31 |
| 5:140574175:C | 1 | 29 | 38 |
| 5:140594388:T | 4 | 221 |  |
| 5:14488196:G | 1 | 4 |  |
| 5:145838641:T | 1 | 122 | 48 |
| 5:145838662:A | 1 | 212 | 49 |
| 5:146619206:C | 2 | 41 | 50 |
| 5:150282713:T | 2 | 5.5 |  |
| 5:162890953:A | 6 | 34 | 49 |
| 5:167913510:A | 6 | 19 | 49 |
| 5:175533585:T | 2 | 209.5 |  |
| 5:176005562:T | 1 | 2.5 |  |
| 5:176005563:T | 1 | 102 |  |
| 5:176013823:T | 1 | 15 | 50 |
| 5:176026129:A | 9 | 4 |  |
| 5:176026138:A | 4 | 22 | 37 |
| 5:176026144:T | 8 | 13 |  |
| 5:177159074:C | 1 | 89.5 |  |
| 5:177161891:G | 11 | 22 | 18 |
| 5:177163580:G | 1 | 28 | 3 |
| 5:179071947:G | 2 | 154 | 49 |
| 5:179072006:G | 1 | 25 | 20 |
| 5:180485353:A | 3 | 13 | 50 |
| 5:180485374:C | 1 | 14 | 49 |
| 5:180582260:T | 4 | 21 | 48 |
| 5:180687440:C | 12 | 202 | 50 |
| 5:23976115:A | 1 | 27 | 50 |
| 5:34807928:T | 4 | 37 | 49 |
| 5:39141240:A | 1 | 23 | 49 |
| 5:40852451:G | 3 | 37 | 50 |
| 5:472055:T | 1 | 29 | 49 |
| 5:53606295:C | 9 | 19 | 50 |
| 5:57854018:T | 3 | 21 | 49 |
| 5:60200665:G | 1 | 27 | 50 |
| 5:64097145:G | 5 | 14 | 50 |
| 5:65317181:T | 2 | 37 | 49 |
| 5:668499:G | 1 | 19 | 9 |
| 5:668500:C | 1 | 16 | 9 |
| 5:68695940:G | 10 | 21 | 50 |
| 5:70751818:G | 10 | 309 | 49 |
| 5:72894207:A | 8 | 16 | 49 |
| 5:73090261:C | 2 | 26 | 48 |
| 5:73148481:A | 8 | 21 | 49 |
| 5:74021853:T | 4 | 76.5 |  |
| 5:741736:T | 9 | 16 | 40 |
| 5:74506658:T | 6 | 24 | 50 |
| 5:74931677:T | 1 | 1 |  |
| 5:76704849:C | 10 | 26 | 49 |
| 5:77311343:A | 2 | 87 |  |
| 5:79950708:C | 7 | 15 | 47 |
| 5:79950715:C | 3 | 12 | 49 |
| 5:79950724:C | 3 | 72 | 49 |
| 5:79950750:G | 7 | 13 | 49 |
| 5:833910:T | 1 | 27 | 31 |
| 5:833915:T | 18 | 21 | 31 |
| 5:837556:G | 1 | 20 | 32 |
| 5:843815:T | 1 | 67 | 4 |
| 5:94772540:T | 9 | 36 | 49 |
| 6:10891781:T | 4 | 20 | 43 |
| 6:10906256:G | 2 | 21 | 49 |
| 6:10928643:T | 1 | 20 | 49 |
| 6:111310252:A | 1 | 37 | 49 |
| 6:112508769:G | 10 | 23 | 49 |
| 6:112508770:T | 10 | 23 | 49 |
| 6:121620608:T | 1 | 18 | 50 |
| 6:123658776:T | 1 | 31 | 50 |
| 6:123687288:C | 6 | 26 | 50 |
| 6:123696766:T | 2 | 22 | 49 |
| 6:132029857:T | 5 | 80 | 4 |
| 6:132029865:A | 1 | 80 | 3 |
| 6:132045195:G | 1 | 22 | 50 |
| 6:133119564:A | 3 | 17 | 49 |
| 6:137026266:G | 8 | 18 | 49 |
| 6:150268515:T | 1 | 60 | 29 |
| 6:151670332:A | 2 | 10 |  |
| 6:160211649:C | 9 | 130 |  |
| 6:160560883:C | 3 | 8 |  |
| 6:165895824:C | 1 | 16 | 50 |
| 6:166736362:T | 4 | 13 | 49 |
| 6:166739646:C | 3 | 20 | 49 |
| 6:167590574:C | 1 | 22 | 12 |
| 6:167595371:C | 3 | 33 |  |
| 6:167790053:T | 1 | 31 | 4 |
| 6:167790110:T | 1 | 123 | 16 |
| 6:167791530:C | 1 | 25 | 11 |
| 6:167791549:G | 2 | 131 | 36 |
| 6:168459845:C | 1 | 29 | 49 |
| 6:170871046:G | 1 | 149 | 45 |
| 6:170871058:A | 1 | 158 | 48 |
| 6:25420350:C | 1 | 30 | 49 |
| 6:26637796:A | 1 | 103 | 0 |
| 6:27115129:G | 1 | 152 | 49 |
| 6:28294209:G | 1 | 18 | 50 |
| 6:29342775:A | 10 | 211 | 49 |
| 6:29342825:A | 9 | 194 | 49 |
| 6:29364615:T | 9 | 202 | 49 |
| 6:29364643:C | 8 | 209 | 49 |
| 6:29364815:G | 6 | 224 | 49 |
| 6:29364835:G | 5 | 227 | 49 |
| 6:29364951:A | 9 | 241 | 49 |
| 6:29365241:C | 9 | 199 | 49 |
| 6:29910358:G | 1 | 1838 | 49 |
| 6:29910640:T | 2 | 2539 | 49 |
| 6:29910663:A | 1 | 2169 | 48 |
| 6:29910717:G | 2 | 1260 | 46 |
| 6:29910730:A | 1 | 1117 | 45 |
| 6:29910731:A | 1 | 1091 | 44 |
| 6:29910759:A | 2 | 30 | 17 |
| 6:29910761:A | 1 | 28 | 16 |
| 6:29910767:C | 3 | 26 | 14 |
| 6:29910771:T | 5 | 24 | 12 |
| 6:29910773:G | 4 | 23 | 11 |
| 6:29910774:C | 4 | 23 | 11 |
| 6:29910777:T | 3 | 24 | 11 |
| 6:29910779:C | 5 | 27 | 12 |
| 6:29911063:G | 6 | 1131 | 45 |
| 6:29911149:T | 1 | 887 | 46 |
| 6:29911225:G | 1 | 765 | 42 |
| 6:29911228:C | 8 | 750 | 42 |
| 6:29911228:T | 5 | 738 | 43 |
| 6:29911228:T,C | 2 | 738 | 43 |
| 6:29911240:G | 8 | 733 | 42 |
| 6:29911260:C | 3 | 654 | 43 |
| 6:29911261:G | 2 | 651 | 43 |
| 6:29912348:G | 8 | 1062 | 47 |
| 6:29913037:A | 2 | 1885 | 46 |
| 6:30071330:T | 2 | 1069 | 49 |
| 6:30075903:T | 1 | 323 | 49 |
| 6:30131515:A | 7 | 730 | 49 |
| 6:30695920:T | 3 | 19 | 49 |
| 6:30882634:T | 6 | 1140 | 49 |
| 6:30882781:T | 10 | 1289 | 49 |
| 6:30887988:T | 6 | 799 | 49 |
| 6:30888169:A | 8 | 1004 | 49 |
| 6:30890483:T | 6 | 1428 | 49 |
| 6:30892322:T | 7 | 653 | 49 |
| 6:30893428:A | 7 | 949 | 49 |
| 6:30919391:A | 2 | 144 | 49 |
| 6:30954738:A | 5 | 432 | 44 |
| 6:30954909:G | 1 | 438 | 40 |
| 6:30954919:G | 1 | 417 | 38 |
| 6:30954939:T | 1 | 488 | 37 |
| 6:30955189:A | 1 | 29 | 38 |
| 6:30955190:C | 1 | 28 | 38 |
| 6:30994024:T | 1 | 27 | 38 |
| 6:30994025:G | 1 | 26 | 38 |
| 6:30994026:A | 1 | 26 | 38 |
| 6:30994059:G | 1 | 17 | 44 |
| 6:31002527:G | 4 | 235 | 49 |
| 6:31079236:A | 7 | 216 | 49 |
| 6:31079643:C | 8 | 218 | 49 |
| 6:31079889:G | 9 | 223 | 49 |
| 6:31080016:G | 5 | 297 | 49 |
| 6:31084787:G | 4 | 566 | 49 |
| 6:31084945:G | 4 | 9 | 49 |
| 6:31110391:C | 7 | 414 | 49 |
| 6:31112737:A | 3 | 1634 | 49 |
| 6:31122482:A | 1 | 1087 | 49 |
| 6:31122500:A | 3 | 1023 | 49 |
| 6:31138371:A | 4 | 645 | 49 |
| 6:31170528:G | 8 | 661 | 49 |
| 6:31237776:T | 2 | 1154 | 37 |
| 6:31238930:C | 1 | 1092 | 43 |
| 6:31239050:A | 1 | 1307 | 45 |
| 6:31239108:A | 7 | 1630 | 47 |
| 6:31239613:T | 1 | 45 |  |
| 6:31322303:G | 2 | 954 | 49 |
| 6:31322980:T | 2 | 1269 | 49 |
| 6:31323321:A | 5 | 1410 | 47 |
| 6:31323958:G | 6 | 395 | 40 |
| 6:31324003:A | 10 | 454 | 35 |
| 6:31324003:A,G | 10 | 454 | 35 |
| 6:31324003:G | 10 | 454 | 35 |
| 6:31324004:G | 10 | 456 | 35 |
| 6:31324004:G,T | 10 | 456 | 35 |
| 6:31324004:T | 10 | 456 | 35 |
| 6:31324024:A | 9 | 487 | 34 |
| 6:31324024:T | 9 | 487 | 34 |
| 6:31324025:C | 1 | 535 | 35 |
| 6:31324145:C | 3 | 683 | 45 |
| 6:31324154:A | 3 | 710 | 45 |
| 6:31324200:C | 2 | 13 | 15 |
| 6:31324201:A | 1 | 13 | 15 |
| 6:31324201:G | 1 | 23 | 15 |
| 6:31324202:A | 1 | 132 | 31 |
| 6:31324202:C | 1 | 13 | 15 |
| 6:31324206:C | 2 | 13 | 15 |
| 6:31324207:C | 2 | 13 | 15 |
| 6:31324208:A | 3 | 130 | 30 |
| 6:31324208:A,T | 1 | 130 | 30 |
| 6:31324208:T | 2 | 858 | 48 |
| 6:31324209:A | 2 | 13 | 15 |
| 6:31324210:A | 6 | 14 | 15 |
| 6:31324489:G | 2 | 24 | 32 |
| 6:31324491:A | 3 | 21 | 31 |
| 6:31324494:G | 3 | 20 | 31 |
| 6:31324495:C | 3 | 20 | 31 |
| 6:31324496:T | 2 | 11 | 22 |
| 6:31324497:A | 3 | 20 | 31 |
| 6:31324499:G | 1 | 25 |  |
| 6:31324506:T | 3 | 14 | 30 |
| 6:31324516:A | 9 | 16 | 28 |
| 6:31324523:C | 3 | 4 |  |
| 6:31324525:A | 1 | 8 |  |
| 6:31324525:T | 1 | 28 | 15 |
| 6:31324526:G | 2 | 28 | 15 |
| 6:31324528:A | 1 | 1.5 |  |
| 6:31324528:T | 1 | 28 | 15 |
| 6:31324530:C | 2 | 28 | 18 |
| 6:31324531:G | 2 | 30 | 19 |
| 6:31324531:G,T | 1 | 30 | 19 |
| 6:31324531:T | 1 | 30 | 19 |
| 6:31324531:T,G | 1 | 30 | 19 |
| 6:31324536:A | 6 | 14 | 24 |
| 6:31324536:C | 1 | 12 | 26 |
| 6:31324536:G | 1 | 11 | 21 |
| 6:31324538:C | 1 | 3.5 |  |
| 6:31324539:T | 1 | 5 |  |
| 6:31324547:C | 3 | 26 | 32 |
| 6:31324549:C | 2 | 23 | 26 |
| 6:31324552:C | 3 | 5.5 |  |
| 6:31324586:T | 10 | 859 | 42 |
| 6:31324641:A | 1 | 1277 | 42 |
| 6:31324664:G | 1 | 1341 | 42 |
| 6:31324666:T | 8 | 1397 | 42 |
| 6:31324887:C | 3 | 687 | 42 |
| 6:31324888:T | 3 | 679 | 42 |
| 6:31540556:C | 3 | 573 | 49 |
| 6:31610686:G | 1 | 2187 | 49 |
| 6:31639979:T | 1 | 1535 | 49 |
| 6:31675501:A | 2 | 829 | 49 |
| 6:31704068:G | 2 | 4 |  |
| 6:31762843:C | 2 | 516 | 49 |
| 6:31762844:T | 4 | 507 | 49 |
| 6:31839331:T | 8 | 393 | 49 |
| 6:31846741:A | 2 | 31 | 49 |
| 6:31864547:T | 6 | 1484 | 49 |
| 6:31922452:T | 2 | 27 |  |
| 6:31922453:C | 1 | 150 |  |
| 6:31922456:C | 2 | 153.5 |  |
| 6:31964316:C | 1 | 20 | 27 |
| 6:31964321:C | 1 | 20 | 24 |
| 6:31964330:G | 1 | 29 | 26 |
| 6:31964331:G | 1 | 32 | 26 |
| 6:31973973:A | 3 | 22 | 49 |
| 6:31974849:G | 3 | 24 | 42 |
| 6:31974857:T | 3 | 9.5 |  |
| 6:31997401:A | 1 | 169 | 23 |
| 6:32006886:A | 1 | 1688 | 49 |
| 6:32010272:A | 9 | 1242 | 47 |
| 6:32012333:T | 1 | 87 | 2 |
| 6:32097290:T | 3 | 143 | 49 |
| 6:32151443:T | 3 | 2552 | 49 |
| 6:32298372:G | 17 | 16 | 49 |
| 6:32299822:A | 6 | 12 | 50 |
| 6:32307382:A | 7 | 29 | 50 |
| 6:32336586:G | 18 | 12 | 50 |
| 6:32363816:C | 6 | 640 | 49 |
| 6:32363844:T | 1 | 700 | 49 |
| 6:32370835:C | 6 | 337 | 49 |
| 6:32485524:G | 1 | 48 | 39 |
| 6:32486344:C | 2 | 15 | 37 |
| 6:32486358:C | 1 | 5 |  |
| 6:32486437:C | 4 | 16 | 19 |
| 6:32486438:A | 1 | 15 | 19 |
| 6:32487175:A | 5 | 180 | 34 |
| 6:32487205:T | 9 | 246 | 31 |
| 6:32489731:C | 1 | 43 | 36 |
| 6:32489732:A | 1 | 42 | 36 |
| 6:32489733:C | 1 | 42 | 36 |
| 6:32489734:A | 1 | 24 | 27 |
| 6:32489735:T | 1 | 23 | 27 |
| 6:32489744:T | 2 | 15 | 11 |
| 6:32489745:G | 2 | 12 | 11 |
| 6:32489747:C | 2 | 12 | 11 |
| 6:32489749:C | 2 | 12 | 11 |
| 6:32489753:T | 1 | 12 | 11 |
| 6:32489755:C | 1 | 12 | 11 |
| 6:32489757:G | 1 | 11 | 11 |
| 6:32489758:C | 2 | 13 | 11 |
| 6:32489766:T | 2 | 18 | 33 |
| 6:32489786:G | 1 | 43 | 34 |
| 6:32489791:G | 1 | 43 | 34 |
| 6:32489806:T | 1 | 19 | 11 |
| 6:32489835:G | 1 | 10 | 36 |
| 6:32489940:C | 1 | 49 | 33 |
| 6:32497904:T | 5 | 136 | 35 |
| 6:32497910:C | 2 | 16 | 26 |
| 6:32497913:A | 2 | 17 | 26 |
| 6:32497917:A | 1 | 15 | 22 |
| 6:32497960:A | 2 | 78 | 33 |
| 6:32497961:A | 2 | 68 |  |
| 6:32497962:C | 1 | 31 |  |
| 6:32497968:A | 1 | 25 | 24 |
| 6:32497970:C | 7 | 25 | 24 |
| 6:32497971:T | 1 | 25 | 24 |
| 6:32497975:G | 8 | 8 | 33 |
| 6:32497984:C | 1 | 18 | 43 |
| 6:32497988:C | 8 | 27 | 31 |
| 6:32548554:T | 2 | 221 |  |
| 6:32548556:T | 2 | 218.5 |  |
| 6:32548581:G | 4 | 101 | 44 |
| 6:32548632:A | 1 | 114 | 43 |
| 6:32549344:C | 1 | 32 | 19 |
| 6:32549351:A | 1 | 33 | 19 |
| 6:32549356:C | 8 | 33 | 19 |
| 6:32549357:A | 2 | 33 | 19 |
| 6:32549368:A | 2 | 14 | 13 |
| 6:32549376:G | 2 | 25 | 19 |
| 6:32549380:A | 1 | 18 | 26 |
| 6:32549548:A | 1 | 245.5 |  |
| 6:32549582:T | 1 | 30 | 31 |
| 6:32549583:C | 1 | 30 | 34 |
| 6:32549584:T | 1 | 30 | 34 |
| 6:32549588:A | 1 | 20 | 32 |
| 6:32549588:T | 1 | 49 | 21 |
| 6:32549589:C | 1 | 379 | 40 |
| 6:32549596:C | 1 | 18 | 30 |
| 6:32549611:C | 5 | 243 |  |
| 6:32549613:C | 5 | 243 |  |
| 6:32551953:G | 1 | 285 |  |
| 6:32551957:C | 9 | 233 | 35 |
| 6:32551957:C,T | 10 | 266 | 35 |
| 6:32551957:T | 10 | 266 | 35 |
| 6:32551957:T,C | 9 | 266 | 35 |
| 6:32551958:T | 1 | 100 | 33 |
| 6:32551960:C | 1 | 99 | 30 |
| 6:32552016:A | 1 | 21 | 16 |
| 6:32552026:T | 1 | 29 | 33 |
| 6:32552029:T | 1 | 23 | 32 |
| 6:32552059:A | 10 | 226 | 37 |
| 6:32552059:T | 11 | 15 | 20 |
| 6:32552059:T,A | 10 | 226 | 37 |
| 6:32552067:T | 1 | 12 | 18 |
| 6:32552072:G | 1 | 12 | 18 |
| 6:32552078:T | 1 | 23 | 18 |
| 6:32552080:C | 1 | 23 | 18 |
| 6:32552081:G | 7 | 166 | 38 |
| 6:32552085:C | 6 | 155 | 39 |
| 6:32552085:T | 7 | 23 | 18 |
| 6:32552091:C | 1 | 23 | 18 |
| 6:32552092:T | 4 | 23 | 18 |
| 6:32552117:G | 1 | 25 | 34 |
| 6:32552130:A | 2 | 48 | 37 |
| 6:32552132:A | 2 | 46 | 37 |
| 6:32552138:C | 1 | 40 | 38 |
| 6:32552147:A | 2 | 45 | 36 |
| 6:32557423:C | 1 | 22 | 12 |
| 6:32557435:C | 1 | 25 | 16 |
| 6:32557461:G | 1 | 17 | 32 |
| 6:32557465:A | 1 | 19 | 31 |
| 6:32557477:A | 2 | 15 | 18 |
| 6:32557478:A | 4 | 29 | 23 |
| 6:32557479:A | 3 | 29 | 23 |
| 6:32557483:C | 5 | 29 | 23 |
| 6:32557486:A | 1 | 15 | 18 |
| 6:32557487:A | 2 | 23 | 30 |
| 6:32557489:T | 1 | 15 | 18 |
| 6:32557490:A | 1 | 29 | 23 |
| 6:32557504:A | 1 | 18 | 24 |
| 6:32557506:C | 4 | 17 | 18 |
| 6:32609169:C | 1 | 32 | 25 |
| 6:32609173:G | 2 | 31 | 24 |
| 6:32609181:T | 9 | 25 | 23 |
| 6:32609192:G | 7 | 15 | 39 |
| 6:32609195:A | 1 | 15 | 40 |
| 6:32609212:A | 3 | 3 |  |
| 6:32609214:T | 2 | 2 |  |
| 6:32609216:T | 2 | 1 |  |
| 6:32609222:T | 1 | 1 |  |
| 6:32609223:T | 1 | 1 |  |
| 6:32609224:C | 1 | 1 |  |
| 6:32609229:A | 1 | 2 |  |
| 6:32609230:C | 1 | 2 |  |
| 6:32609241:A | 6 | 5 |  |
| 6:32609299:C | 2 | 13 | 11 |
| 6:32609299:G | 1 | 12 | 27 |
| 6:32609312:C | 13 | 23 | 25 |
| 6:32610436:C | 3 | 595 | 43 |
| 6:32610535:C | 1 | 17 | 16 |
| 6:32628022:G | 5 | 32 | 16 |
| 6:32629146:A | 7 | 197 | 42 |
| 6:32629227:T | 1 | 204 | 34 |
| 6:32629755:A | 9 | 304 | 47 |
| 6:32632578:G | 1 | 93 | 41 |
| 6:32632581:G | 2 | 87 | 41 |
| 6:32632589:G | 3 | 85 | 41 |
| 6:32632592:G | 3 | 85 | 41 |
| 6:32632593:T | 2 | 94 | 41 |
| 6:32632599:G | 1 | 95 | 41 |
| 6:32632601:T | 2 | 89 | 42 |
| 6:32632605:A | 4 | 90 | 42 |
| 6:32632608:G | 8 | 92 | 42 |
| 6:32632647:C | 1 | 26 | 14 |
| 6:32632703:A | 1 | 22 | 18 |
| 6:32632714:C | 1 | 15 | 21 |
| 6:32632718:A | 1 | 18 | 21 |
| 6:32632721:T | 1 | 21 | 22 |
| 6:32632724:T | 3 | 256 | 42 |
| 6:32632744:T | 1 | 19 | 22 |
| 6:32632745:A | 1 | 20 | 22 |
| 6:32632748:A | 1 | 20 | 22 |
| 6:32632749:T | 1 | 20 | 22 |
| 6:32632753:T | 3 | 13 | 9 |
| 6:32632820:G | 1 | 27 | 32 |
| 6:32632832:T | 1 | 35 | 33 |
| 6:32632833:G | 1 | 35 | 33 |
| 6:32634300:C | 5 | 23 | 20 |
| 6:32634301:C | 1 | 24 | 18 |
| 6:32634302:G | 5 | 23 | 20 |
| 6:32634303:A | 5 | 23 | 20 |
| 6:32634306:T | 5 | 23 | 20 |
| 6:32634313:G | 4 | 28 | 21 |
| 6:32634318:A | 4 | 28 | 21 |
| 6:32634331:A | 10 | 644 | 41 |
| 6:32634331:C | 10 | 644 | 41 |
| 6:32634331:C,A | 8 | 644 | 41 |
| 6:32725062:A | 9 | 14 | 50 |
| 6:32790089:A | 2 | 16 | 49 |
| 6:32797297:C | 8 | 318 | 49 |
| 6:32825090:A | 2 | 484 | 49 |
| 6:32942302:A | 1 | 337 | 49 |
| 6:32945686:A | 1 | 9.5 |  |
| 6:32948426:T | 7 | 1120 | 49 |
| 6:32975869:A | 1 | 805 | 49 |
| 6:33179689:T | 8 | 402 | 49 |
| 6:33235755:A | 8 | 1646 | 49 |
| 6:350829:A | 8 | 34 | 49 |
| 6:36733132:A | 8 | 20 | 50 |
| 6:38650628:G | 1 | 28 | 50 |
| 6:39048491:C | 7 | 19 | 49 |
| 6:41754576:T | 1 | 143 | 50 |
| 6:42123317:C | 6 | 24 | 49 |
| 6:42185564:C | 9 | 25 | 49 |
| 6:42233518:T | 1 | 26 | 50 |
| 6:43250734:G | 6 | 180 | 49 |
| 6:44140054:C | 1 | 53.5 |  |
| 6:47548626:A | 1 | 19 | 49 |
| 6:49712063:G | 1 | 22 | 49 |
| 6:49931783:G | 1 | 22 | 50 |
| 6:5086211:C | 2 | 24 | 50 |
| 6:52621101:T | 1 | 18 | 50 |
| 6:54054686:A | 11 | 24 | 49 |
| 6:55266625:C | 1 | 27 | 49 |
| 6:57393125:G | 10 | 17 | 50 |
| 6:57467100:C | 10 | 9 | 44 |
| 6:70733547:G | 1 | 32 | 50 |
| 6:70963124:C | 3 | 25 | 49 |
| 6:74466377:T | 7 | 25 | 50 |
| 6:74533192:G | 1 | 31 | 49 |
| 6:7563983:G | 9 | 20 | 49 |
| 6:84884494:C | 1 | 117 |  |
| 6:88108051:A | 11 | 8 |  |
| 6:89981413:C | 1 | 36 | 50 |
| 7:100550079:G | 6 | 26 | 50 |
| 7:100637605:C | 1 | 50 |  |
| 7:100641745:A | 1 | 194 | 7 |
| 7:100642950:C | 1 | 81 |  |
| 7:100642986:A | 1 | 128 | 18 |
| 7:100643060:A | 2 | 177 | 17 |
| 7:100643282:G | 1 | 123 | 11 |
| 7:100643575:T | 1 | 133 | 19 |
| 7:100644027:A | 1 | 151 | 14 |
| 7:100645825:A | 2 | 285 |  |
| 7:100646055:G | 5 | 10 |  |
| 7:100646127:G | 6 | 14.5 |  |
| 7:100646414:T | 3 | 25.5 |  |
| 7:100646418:C | 2 | 24 |  |
| 7:100646420:T | 2 | 24 |  |
| 7:100646966:C | 1 | 11 | 17 |
| 7:100680463:T | 1 | 194 |  |
| 7:100680472:A | 1 | 184 |  |
| 7:100680490:G | 1 | 190 |  |
| 7:100680525:C | 1 | 174 |  |
| 7:101194424:T | 8 | 22 | 49 |
| 7:101837149:A | 6 | 21 | 50 |
| 7:101928495:C | 1 | 24 | 49 |
| 7:102125519:C | 3 | 44 | 6 |
| 7:102182021:A | 1 | 146 | 3 |
| 7:102210332:G | 1 | 73 |  |
| 7:102212939:C | 1 | 25 | 27 |
| 7:102294074:C | 1 | 43 | 1 |
| 7:102309418:A | 3 | 28 | 14 |
| 7:104110492:T | 5 | 15 | 49 |
| 7:115614254:G | 1 | 28 | 50 |
| 7:11871480:G | 1 | 23 |  |
| 7:11871481:A | 1 | 24 |  |
| 7:120776097:G | 2 | 30 | 49 |
| 7:121653377:G | 1 | 17.5 |  |
| 7:123672479:T | 5 | 179 | 47 |
| 7:128140982:C | 1 | 33 | 49 |
| 7:128587381:C | 6 | 20 | 41 |
| 7:130418689:G | 1 | 34 | 47 |
| 7:130418720:C | 8 | 33 | 49 |
| 7:130418721:G | 8 | 34 | 49 |
| 7:130418744:A | 7 | 32 | 48 |
| 7:134225827:G | 11 | 20 | 50 |
| 7:134264286:T | 10 | 21 | 50 |
| 7:135082953:C | 1 | 24 | 50 |
| 7:137128830:C | 8 | 19 | 50 |
| 7:141957504:G | 3 | 24 | 49 |
| 7:142498735:C | 5 | 103 | 44 |
| 7:142498738:G | 5 | 102 | 44 |
| 7:142498751:A | 2 | 89 | 45 |
| 7:142499071:C | 10 | 107 | 50 |
| 7:143269773:T | 1 | 160 | 8 |
| 7:143270001:T | 4 | 126 |  |
| 7:143417322:T | 2 | 60 | 1 |
| 7:143453710:A | 1 | 75 | 3 |
| 7:143956181:C | 1 | 187 | 2 |
| 7:144070338:G | 1 | 10 |  |
| 7:148936878:T | 4 | 17 | 49 |
| 7:150069404:A | 6 | 4 |  |
| 7:152520490:T | 3 | 175 | 33 |
| 7:15430486:G | 1 | 155 |  |
| 7:154429560:T | 10 | 38 | 49 |
| 7:154989982:A | 4 | 12 | 50 |
| 7:154990003:T | 4 | 23 | 49 |
| 7:157929370:T | 1 | 23 | 49 |
| 7:158664076:G | 3 | 8.5 |  |
| 7:22197488:G | 1 | 12 | 49 |
| 7:25266573:C | 8 | 162 |  |
| 7:25267963:C | 8 | 26 | 49 |
| 7:32209425:G | 10 | 20 | 49 |
| 7:32529936:C | 7 | 26 | 49 |
| 7:34874038:G | 1 | 18 | 49 |
| 7:38279663:C | 2 | 61 | 8 |
| 7:38279725:A | 1 | 108 | 26 |
| 7:38299789:G | 1 | 65 | 18 |
| 7:38309102:C | 1 | 28 | 44 |
| 7:43810764:A | 8 | 13 | 49 |
| 7:48545976:G | 10 | 27 | 49 |
| 7:5352809:A | 1 | 164 | 45 |
| 7:55238874:A | 7 | 10 | 49 |
| 7:5949726:C | 4 | 19 | 18 |
| 7:5965288:A | 2 | 29 | 27 |
| 7:5997641:G | 3 | 96 | 3 |
| 7:6005323:T | 1 | 37 |  |
| 7:6013049:G | 3 | 50 | 2 |
| 7:6803602:T | 1 | 144 | 25 |
| 7:6836259:T | 1 | 17 | 37 |
| 7:72412705:T | 1 | 26 | 20 |
| 7:72413423:T | 7 | 45 | 28 |
| 7:72413443:A | 2 | 48 | 36 |
| 7:72413581:A | 2 | 63 | 42 |
| 7:72413593:C | 1 | 43 | 38 |
| 7:72436652:G | 10 | 18 | 20 |
| 7:74193642:A | 1 | 23 | 4 |
| 7:74193668:A | 1 | 20 | 15 |
| 7:74212036:G | 1 | 35 | 39 |
| 7:74212048:T | 1 | 38 | 41 |
| 7:74223500:T | 3 | 21 | 7 |
| 7:75052079:A | 1 | 27 |  |
| 7:75052102:A | 7 | 22 | 5 |
| 7:75677504:T | 1 | 28 | 49 |
| 7:76129758:A | 4 | 20 | 10 |
| 7:76144560:C | 9 | 154 | 49 |
| 7:7841338:G | 2 | 18 | 50 |
| 7:94030899:C | 2 | 27 | 49 |
| 7:94946084:T | 2 | 29 | 49 |
| 7:95800820:C | 7 | 14 | 49 |
| 8:101719121:A | 1 | 23 | 36 |
| 8:103573015:G | 1 | 15 | 48 |
| 8:103573019:A | 8 | 63 | 48 |
| 8:103573019:A,G | 1 | 16 |  |
| 8:103573019:G | 5 | 63 | 48 |
| 8:103573019:G,A | 10 | 63 | 48 |
| 8:10466019:T | 2 | 16 | 40 |
| 8:10467605:T | 1 | 152 | 45 |
| 8:10467636:C | 1 | 100 | 41 |
| 8:10467652:C | 2 | 116 | 43 |
| 8:10467653:T | 3 | 116 | 43 |
| 8:104940095:T | 1 | 19 | 50 |
| 8:10530218:T | 8 | 19 | 49 |
| 8:113253974:A | 1 | 39 | 49 |
| 8:117783995:G | 2 | 8 |  |
| 8:119964052:C | 10 | 16 | 50 |
| 8:12041211:T | 1 | 84 | 2 |
| 8:12043908:G | 1 | 134 |  |
| 8:12043910:C | 1 | 132 |  |
| 8:12044328:C | 1 | 169 |  |
| 8:12283494:T | 1 | 73 | 1 |
| 8:12286189:G | 1 | 132 | 4 |
| 8:12286191:C | 1 | 133 | 4 |
| 8:12286609:C | 1 | 82 | 0 |
| 8:12291593:A | 2 | 22 | 13 |
| 8:133047071:A | 2 | 30 | 50 |
| 8:133067265:T | 1 | 25 | 49 |
| 8:133854769:A | 2 | 14.5 |  |
| 8:133854771:C | 1 | 152 |  |
| 8:134256610:T | 1 | 23 | 50 |
| 8:139642974:C | 1 | 27 | 50 |
| 8:139647262:G | 2 | 30 | 49 |
| 8:139712355:A | 2 | 23 | 48 |
| 8:143833850:A | 1 | 30 | 49 |
| 8:143833856:T | 1 | 31 | 49 |
| 8:143867905:T | 3 | 20 | 49 |
| 8:144295183:T | 10 | 21 | 49 |
| 8:144623574:C | 2 | 22 | 49 |
| 8:144911463:C | 1 | 19 | 49 |
| 8:144940300:A | 6 | 158 | 46 |
| 8:145557497:C | 2 | 29 | 49 |
| 8:145773344:T | 1 | 10 |  |
| 8:145999619:C | 4 | 10 |  |
| 8:1514009:A | 6 | 175 | 49 |
| 8:17270787:G | 11 | 20 | 48 |
| 8:22526559:A | 4 | 12 | 50 |
| 8:22584718:C | 7 | 19 | 50 |
| 8:24326313:G | 1 | 19 | 49 |
| 8:25323777:C | 10 | 13 | 50 |
| 8:27094399:T | 1 | 23 | 50 |
| 8:27396208:A | 4 | 17 | 50 |
| 8:38176435:G | 1 | 23 | 49 |
| 8:39862881:T | 4 | 25 | 49 |
| 8:6500544:T | 5 | 33 | 49 |
| 8:69143589:G | 3 | 27 | 50 |
| 8:70588878:C | 1 | 30 | 49 |
| 8:70850464:T | 11 | 6 |  |
| 8:7272594:G | 7 | 51 | 10 |
| 8:7320252:T | 8 | 66 | 15 |
| 8:7353480:T | 2 | 31 | 12 |
| 8:7673126:A | 4 | 34 | 10 |
| 8:7681350:T | 2 | 117 | 3 |
| 8:7694034:G | 3 | 28 | 34 |
| 8:79578393:A | 1 | 17 | 50 |
| 8:87226642:T | 7 | 6 |  |
| 8:87666251:G | 9 | 23 | 49 |
| 9:100616706:A | 1 | 56 | 47 |
| 9:103348361:A | 1 | 91 | 41 |
| 9:107367666:A | 1 | 2.5 |  |
| 9:107367666:T | 12 | 7 |  |
| 9:111945049:G | 1 | 23 | 50 |
| 9:112542787:T | 1 | 28 | 50 |
| 9:116973273:T | 2 | 21 | 50 |
| 9:117033022:C | 1 | 30 | 49 |
| 9:117166338:C | 5 | 32 | 49 |
| 9:122275247:T | 1 | 31 | 49 |
| 9:123291036:G | 1 | 26 | 49 |
| 9:130698029:C | 5 | 32 | 49 |
| 9:132630668:C | 18 | 151 | 50 |
| 9:132872686:G | 3 | 21 | 49 |
| 9:134401335:A | 1 | 23 | 49 |
| 9:134401335:T | 9 | 32 | 50 |
| 9:135105964:G | 11 | 19 | 49 |
| 9:136268084:G | 2 | 30 | 49 |
| 9:136518097:G | 3 | 30 | 50 |
| 9:138150721:T | 1 | 42 | 28 |
| 9:138150725:G | 2 | 24 | 17 |
| 9:138150726:G | 2 | 24 | 17 |
| 9:138150727:C | 2 | 24 | 17 |
| 9:138150737:G | 2 | 23 | 15 |
| 9:138150740:C | 2 | 23 | 15 |
| 9:138150742:T | 2 | 23 | 15 |
| 9:138150752:T | 2 | 22 | 14 |
| 9:138150753:C | 1 | 55 | 35 |
| 9:138150753:C,G | 1 | 55 | 35 |
| 9:138150753:G | 2 | 22 | 14 |
| 9:138150754:G | 2 | 22 | 14 |
| 9:138150809:C | 1 | 17 | 16 |
| 9:138150811:A | 1 | 19 | 16 |
| 9:138150825:A | 1 | 30 | 14 |
| 9:138150833:C | 1 | 39 | 16 |
| 9:138150836:T | 1 | 24 | 18 |
| 9:138150849:A | 1 | 55 | 18 |
| 9:138150849:A,G | 1 | 55 | 18 |
| 9:138150852:C | 1 | 56 | 17 |
| 9:138151062:G | 1 | 55 | 26 |
| 9:138151076:C | 1 | 27 | 10 |
| 9:138151077:A | 1 | 23 | 10 |
| 9:138151078:T | 1 | 24 | 9 |
| 9:138151117:C | 1 | 17 | 11 |
| 9:138151118:A | 1 | 17 | 11 |
| 9:138151118:A,G | 1 | 17 | 11 |
| 9:138151118:G | 1 | 17 | 11 |
| 9:138151118:G,A | 1 | 17 | 11 |
| 9:138151119:A | 1 | 21 | 12 |
| 9:138151145:C | 1 | 81 | 24 |
| 9:138151147:T | 1 | 120 | 33 |
| 9:138151156:T | 1 | 86 | 26 |
| 9:138151161:G | 1 | 122 | 34 |
| 9:138151169:C | 1 | 131 | 35 |
| 9:138151184:C | 1 | 112 | 32 |
| 9:138151225:C | 1 | 166 | 38 |
| 9:138836946:A | 3 | 53 |  |
| 9:139222174:C | 1 | 18 | 49 |
| 9:139849847:C | 2 | 25 | 49 |
| 9:140123265:G | 1 | 11 |  |
| 9:140262426:T | 5 | 31 | 49 |
| 9:178937:T | 2 | 13 | 18 |
| 9:178947:A | 1 | 20 | 19 |
| 9:18675897:G | 1 | 26 | 49 |
| 9:19622268:C | 8 | 14 | 49 |
| 9:33240225:A | 1 | 32 | 49 |
| 9:33548843:A | 1 | 5 |  |
| 9:33572347:C | 2 | 27 | 49 |
| 9:33796694:A | 4 | 102 |  |
| 9:34459025:T | 2 | 23 | 50 |
| 9:35906601:A | 2 | 120 | 44 |
| 9:37492679:T | 1 | 21 | 50 |
| 9:37711382:A | 1 | 23 | 50 |
| 9:38411408:G | 1 | 2 |  |
| 9:40706117:T | 2 | 152 | 3 |
| 9:40706227:A | 1 | 137 | 3 |
| 9:42410368:C | 1 | 154 | 2 |
| 9:43129577:A | 1 | 211 | 5 |
| 9:43625044:C | 1 | 29 |  |
| 9:43876040:A | 10 | 45 | 3 |
| 9:43876136:G | 5 | 24 | 6 |
| 9:46386846:T | 1 | 111 | 0 |
| 9:46390597:G | 3 | 59 | 9 |
| 9:65505933:A | 1 | 147 | 2 |
| 9:67960920:G | 1 | 26 | 7 |
| 9:67968720:C | 1 | 185 | 5 |
| 9:67968798:C | 1 | 203 | 14 |
| 9:67987865:C | 4 | 20 | 19 |
| 9:69238268:A | 3 | 26 | 39 |
| 9:69247550:A | 7 | 15 | 18 |
| 9:69256816:A | 3 | 72 | 6 |
| 9:69653122:T | 2 | 23 | 11 |
| 9:70177212:G | 1 | 43 | 3 |
| 9:7046901:G | 2 | 28 | 49 |
| 9:70919114:G | 1 | 91 | 2 |
| 9:72755129:G | 2 | 7.5 |  |
| 9:74674278:T | 1 | 15 |  |
| 9:75303653:T | 9 | 20 | 49 |
| 9:79318378:A | 10 | 5 |  |
| 9:79318381:T | 16 | 2 |  |
| 9:82187750:G | 4 | 19 | 50 |
| 9:8497250:C | 1 | 18 | 49 |
| 9:88275882:T | 1 | 16 | 50 |
| 9:88937856:C | 13 | 184.5 |  |
| 9:93978356:C | 1 | 19 | 50 |
| 9:96278538:A | 1 | 164 |  |
| 9:96438998:C | 8 | 71 |  |
| 9:96439007:T | 11 | 159 |  |
| 9:97080947:G | 17 | 13 | 9 |
| 9:99060704:T | 1 | 23 | 50 |
| X:100105159:A | 4 | 7.5 |  |
| X:100524197:T | 3 | 31 | 49 |
| X:100531437:T | 2 | 123 | 49 |
| X:100531443:A | 3 | 27 |  |
| X:100531443:A,C | 2 | 30.5 |  |
| X:100531443:C | 12 | 56 |  |
| X:100531446:C | 10 | 132 |  |
| X:100748078:G | 1 | 23 | 50 |
| X:101576798:C | 13 | 244 |  |
| X:102192380:T | 2 | 113 |  |
| X:102192787:T | 4 | 153.5 |  |
| X:102612705:A | 13 | 19 |  |
| X:102612709:A | 3 | 87.5 |  |
| X:102841883:T | 11 | 15 |  |
| X:102841886:C | 13 | 41.5 |  |
| X:103495061:C | 1 | 257 |  |
| X:103495101:T | 5 | 128.5 |  |
| X:103495109:T | 13 | 277 |  |
| X:103495134:A | 1 | 239 |  |
| X:103495163:T | 13 | 212 |  |
| X:103495190:G | 13 | 177.5 |  |
| X:103495202:T | 10 | 167 |  |
| X:103495207:C | 11 | 145 |  |
| X:103495208:A | 12 | 144 |  |
| X:103495220:T | 8 | 131 |  |
| X:103495268:A | 13 | 112 |  |
| X:103495268:A,T | 13 | 108.5 |  |
| X:107462966:A | 1 | 28 | 49 |
| X:112022628:G | 13 | 8 |  |
| X:112022628:T | 2 | 1.5 |  |
| X:112022630:G | 7 | 81 |  |
| X:112022631:G | 13 | 65 |  |
| X:114141475:G | 3 | 99.5 |  |
| X:114364721:C | 1 | 5 |  |
| X:114425210:A | 2 | 101 | 44 |
| X:114468509:C | 13 | 8 |  |
| X:114468509:G | 2 | 2.5 |  |
| X:118920677:C | 1 | 21 | 47 |
| X:120009234:T | 10 | 1.5 |  |
| X:125955451:A | 13 | 118.5 |  |
| X:132161713:C | 13 | 18 |  |
| X:134872227:T | 4 | 35 | 0 |
| X:134872271:C | 7 | 35 | 0 |
| X:134947963:G | 7 | 9 |  |
| X:134947965:G | 12 | 7 |  |
| X:134947967:T | 11 | 7.5 |  |
| X:134947984:A | 13 | 6 |  |
| X:134947986:A | 13 | 6 |  |
| X:134947996:T | 13 | 4 |  |
| X:134948000:T | 13 | 3 |  |
| X:134948014:G | 13 | 3 |  |
| X:134948023:C | 13 | 3 |  |
| X:134948034:G | 13 | 4 |  |
| X:134986700:C | 2 | 32 | 48 |
| X:135313739:C | 2 | 82 |  |
| X:135313788:C | 6 | 149 |  |
| X:136648991:T | 13 | 137 |  |
| X:136649003:T | 13 | 147.5 |  |
| X:13680922:G | 3 | 16.5 |  |
| X:13680924:T | 13 | 19 |  |
| X:13681504:G | 6 | 10.5 |  |
| X:13681506:C | 13 | 16 |  |
| X:139586507:C | 3 | 8 |  |
| X:139586509:A | 11 | 11 |  |
| X:139586512:T | 2 | 83 |  |
| X:140270784:C | 13 | 24.5 |  |
| X:140335705:T | 1 | 86 | 5 |
| X:140335706:C | 1 | 84 | 5 |
| X:140335742:C | 2 | 55 | 6 |
| X:140336584:C | 3 | 1 |  |
| X:140785696:C | 1 | 25 | 4 |
| X:140785739:C | 3 | 49 | 47 |
| X:140785741:C | 3 | 49 | 47 |
| X:140967157:G | 13 | 3.5 |  |
| X:140993885:T | 12 | 147 |  |
| X:140993886:A | 10 | 142.5 |  |
| X:140993905:G,T | 13 | 106 |  |
| X:140993905:T | 1 | 52.5 |  |
| X:140993905:T,G | 2 | 114 |  |
| X:140993926:G | 6 | 33 | 36 |
| X:140993932:A | 3 | 26 | 30 |
| X:140993945:C | 2 | 17 | 19 |
| X:140993947:C | 1 | 16 | 22 |
| X:140993957:G | 2 | 23 | 30 |
| X:140994031:T | 1 | 224 | 42 |
| X:140994096:A | 7 | 111 |  |
| X:140994109:A | 12 | 90 |  |
| X:140994113:C | 4 | 89 |  |
| X:140994120:T | 13 | 81.5 |  |
| X:140994136:C | 4 | 100.5 |  |
| X:140994136:C,G | 5 | 99 |  |
| X:140994136:G,C | 13 | 100 |  |
| X:140994197:G | 4 | 58 |  |
| X:140994201:A | 2 | 57 |  |
| X:140994462:C | 1 | 81.5 |  |
| X:140994472:T | 6 | 80 |  |
| X:140994486:C | 13 | 161 |  |
| X:140994510:A | 13 | 146 |  |
| X:140994512:T | 13 | 141 |  |
| X:140994535:C | 11 | 120 |  |
| X:140995535:C | 6 | 9 |  |
| X:140995538:C | 13 | 163 |  |
| X:141291565:A | 1 | 15 |  |
| X:142596941:G | 3 | 23 | 49 |
| X:144329121:C | 13 | 57 |  |
| X:14861870:A | 13 | 4 |  |
| X:14861875:A | 13 | 4 |  |
| X:148627327:A | 11 | 188 |  |
| X:148627329:A | 13 | 189 |  |
| X:148627335:T | 13 | 191 |  |
| X:148627336:T | 13 | 186 |  |
| X:148628334:T,G | 2 | 85 |  |
| X:149100781:T | 13 | 225 |  |
| X:149101932:T | 6 | 50 |  |
| X:149101934:C | 7 | 59.5 |  |
| X:149101991:T | 13 | 100 |  |
| X:149681040:C | 13 | 150 |  |
| X:149681049:T | 1 | 141 |  |
| X:150832707:G | 13 | 5 |  |
| X:150832716:G | 1 | 15 | 47 |
| X:150832720:G | 1 | 22 | 48 |
| X:150840695:T | 1 | 6 |  |
| X:150840698:A | 13 | 2 |  |
| X:150840699:A | 13 | 2 |  |
| X:150840706:A | 2 | 1.5 |  |
| X:150840707:A | 2 | 1.5 |  |
| X:150840799:A,C | 1 | 8 |  |
| X:150840799:A,T | 2 | 11 |  |
| X:150840799:C | 1 | 7 |  |
| X:150840799:C,A | 1 | 6 |  |
| X:151821277:A | 2 | 32 | 49 |
| X:151869339:A | 13 | 109 |  |
| X:151869356:T | 2 | 50.5 |  |
| X:151869684:A | 12 | 240 |  |
| X:151869731:A | 13 | 254.5 |  |
| X:151869998:A | 9 | 193 |  |
| X:151870043:T | 1 | 107.5 |  |
| X:151870050:T | 2 | 131 |  |
| X:151870055:C | 3 | 262 |  |
| X:151870070:G | 13 | 268 |  |
| X:151935354:C | 13 | 37.5 |  |
| X:151935356:G | 13 | 42 |  |
| X:151935366:G | 3 | 24.5 |  |
| X:151935469:T | 13 | 203.5 |  |
| X:151935747:A | 13 | 147 |  |
| X:151935789:T | 13 | 159 |  |
| X:151935918:A | 13 | 242 |  |
| X:151936140:A | 13 | 108 |  |
| X:152244509:A | 2 | 172 | 4 |
| X:153171699:G | 9 | 6 |  |
| X:153416315:G | 8 | 8 |  |
| X:153416316:A | 8 | 6.5 |  |
| X:153416346:C | 2 | 6 |  |
| X:153416346:G | 5 | 8.5 |  |
| X:153416411:T | 13 | 19 |  |
| X:153418456:A | 13 | 5 |  |
| X:153418486:C | 9 | 1.5 |  |
| X:153418514:A | 9 | 2 |  |
| X:153418516:T | 8 | 2 |  |
| X:153418524:T | 7 | 2 |  |
| X:153418535:G | 3 | 2 |  |
| X:153418541:G | 1 | 29 | 37 |
| X:153421927:A | 13 | 146.5 |  |
| X:153455644:A | 2 | 76 |  |
| X:153455646:T | 2 | 77 |  |
| X:153455654:T | 12 | 80 |  |
| X:153455665:G | 13 | 176 |  |
| X:153455679:A | 13 | 210 |  |
| X:153455691:A | 13 | 222 |  |
| X:153455695:G | 13 | 225 |  |
| X:153459057:A | 8 | 183 |  |
| X:153490660:T | 13 | 268 |  |
| X:153492716:G | 3 | 101 | 2 |
| X:153657145:A,G | 1 | 66 |  |
| X:154456747:G | 10 | 19 | 49 |
| X:15841211:G | 13 | 166 |  |
| X:18348758:C | 12 | 7 |  |
| X:18725922:T | 1 | 19 | 50 |
| X:26212262:T | 10 | 36 |  |
| X:26212334:C | 6 | 82.5 |  |
| X:26212334:T,C | 3 | 76 |  |
| X:2632482:T | 7 | 16 | 50 |
| X:2656258:A | 1 | 25 | 49 |
| X:2656261:G | 1 | 23 | 49 |
| X:2724760:C | 11 | 21 | 50 |
| X:27765405:A | 1 | 76 | 49 |
| X:27765408:A | 7 | 81 | 48 |
| X:27765420:A | 13 | 158 |  |
| X:27765435:A | 13 | 154 |  |
| X:2778076:C | 11 | 17 |  |
| X:2836007:C | 13 | 11 |  |
| X:2847299:A | 1 | 35 | 49 |
| X:295132:C | 8 | 322 | 49 |
| X:295231:G | 6 | 236 | 49 |
| X:299591:G | 5 | 461 | 49 |
| X:306317:C | 6 | 263 | 49 |
| X:34148877:G | 2 | 100 | 40 |
| X:34148882:T | 1 | 106 | 43 |
| X:34149683:C | 7 | 16.5 |  |
| X:34149684:T | 9 | 34 |  |
| X:34149695:C | 8 | 22.5 |  |
| X:34149696:T | 6 | 41 |  |
| X:37028388:T | 1 | 28 | 31 |
| X:37028389:G | 1 | 27 | 31 |
| X:37028415:A | 1 | 16 | 15 |
| X:37028424:T | 1 | 15 | 16 |
| X:37028425:G | 1 | 15 | 16 |
| X:37028427:G | 1 | 18 | 19 |
| X:37029578:G | 3 | 74 |  |
| X:3761423:A | 1 | 6 |  |
| X:37850325:C | 13 | 8 |  |
| X:37850326:A | 6 | 18 |  |
| X:37981362:G | 8 | 91.5 |  |
| X:38145121:C | 7 | 108 |  |
| X:38145132:T | 6 | 9 |  |
| X:38145142:C | 10 | 40.5 |  |
| X:38145304:C | 1 | 11 |  |
| X:38145351:C | 11 | 7.5 |  |
| X:38145561:T | 1 | 76 |  |
| X:38145708:A | 4 | 60 |  |
| X:38145708:G | 1 | 51.5 |  |
| X:38145708:T | 12 | 143 |  |
| X:38146032:C | 13 | 5 |  |
| X:38146052:T | 8 | 81 |  |
| X:46359390:G | 13 | 9 |  |
| X:47428430:T | 13 | 5.5 |  |
| X:48269486:T | 1 | 23 |  |
| X:48269487:C | 1 | 23.5 |  |
| X:48269495:A | 2 | 28 |  |
| X:48433155:G | 5 | 6 |  |
| X:48759546:T | 13 | 30 |  |
| X:48759553:C | 10 | 19 |  |
| X:48970565:C | 4 | 8 |  |
| X:48970568:C | 13 | 132.5 |  |
| X:49076221:C | 1 | 85.5 |  |
| X:49076224:C | 1 | 86 |  |
| X:49081291:A | 1 | 30 | 49 |
| X:49103224:A | 2 | 25 | 50 |
| X:49114790:C | 4 | 16 |  |
| X:49114792:A | 13 | 15 |  |
| X:49126995:T | 13 | 5 |  |
| X:49179711:T | 11 | 61 |  |
| X:49189246:T | 14 | 188 |  |
| X:49189258:C | 13 | 190 |  |
| X:49355893:G | 1 | 30 | 17 |
| X:49368274:T | 1 | 2.5 |  |
| X:49846390:A | 8 | 28 | 49 |
| X:51075856:T | 1 | 108 |  |
| X:51075886:A | 5 | 118.5 |  |
| X:51076081:T | 3 | 9 |  |
| X:51076083:G | 4 | 239 |  |
| X:51076295:T | 7 | 102 |  |
| X:52679429:G | 1 | 19 | 50 |
| X:53115059:T | 13 | 23 |  |
| X:54566663:G | 13 | 2 |  |
| X:54783870:T | 13 | 142.5 |  |
| X:54783871:T | 11 | 72 |  |
| X:54841980:C | 1 | 78 |  |
| X:54841981:A | 1 | 69 |  |
| X:55103931:C | 13 | 75 |  |
| X:55103957:T | 1 | 35.5 |  |
| X:55117864:C | 8 | 86.5 |  |
| X:55117888:A | 9 | 128.5 |  |
| X:55117890:T | 13 | 138 |  |
| X:55515165:T | 7 | 17 |  |
| X:56591811:T | 13 | 166 |  |
| X:57146357:T | 7 | 207 |  |
| X:57162747:A | 1 | 73 |  |
| X:57162833:T | 8 | 185.5 |  |
| X:57618721:G | 1 | 106.5 |  |
| X:57619114:C | 4 | 248 |  |
| X:57619118:C | 8 | 109 |  |
| X:57619121:C | 8 | 246 |  |
| X:57619129:C | 13 | 241 |  |
| X:57619156:G | 13 | 245.5 |  |
| X:57619164:G | 13 | 241 |  |
| X:57619166:T | 13 | 238 |  |
| X:57619168:C | 13 | 240.5 |  |
| X:57619273:T | 11 | 243 |  |
| X:57619276:T | 11 | 243.5 |  |
| X:57619399:T | 1 | 93.5 |  |
| X:57620515:C | 13 | 222 |  |
| X:57620701:T | 6 | 74 |  |
| X:57620760:G | 13 | 141 |  |
| X:57620870:G | 2 | 171 |  |
| X:57934493:A | 8 | 18 |  |
| X:57934493:A,C | 9 | 22 |  |
| X:57934493:C | 13 | 18 |  |
| X:57934493:C,A | 4 | 14.5 |  |
| X:57934520:A | 13 | 50 |  |
| X:57934522:C | 13 | 52 |  |
| X:57934556:A | 13 | 99 |  |
| X:57934566:T | 13 | 102 |  |
| X:57934577:C | 11 | 117 |  |
| X:57934586:T | 13 | 117.5 |  |
| X:57934588:T | 13 | 116 |  |
| X:57934878:T | 11 | 125 |  |
| X:57934904:T | 13 | 128 |  |
| X:57936433:C | 13 | 14 | 20 |
| X:57936462:A | 6 | 8.5 |  |
| X:63411894:G | 13 | 6 |  |
| X:6451793:G | 13 | 134.5 |  |
| X:6451804:C | 13 | 138 |  |
| X:6451809:C | 13 | 136 |  |
| X:6451813:A | 13 | 148 |  |
| X:6451839:T | 13 | 169.5 |  |
| X:6451843:A | 7 | 84.5 |  |
| X:6451873:A | 9 | 155 |  |
| X:6451924:C | 13 | 222.5 |  |
| X:6451993:A | 2 | 220 |  |
| X:6452051:A | 13 | 199.5 |  |
| X:6452077:T | 13 | 204 |  |
| X:6452086:A | 13 | 202 |  |
| X:6452125:T | 13 | 223.5 |  |
| X:6452180:A | 13 | 243.5 |  |
| X:6452199:A | 13 | 260 |  |
| X:6452461:T | 12 | 126 |  |
| X:70321147:A | 13 | 13 |  |
| X:70321147:A,C | 12 | 5.5 |  |
| X:70321147:C | 13 | 13 |  |
| X:70321147:C,A | 12 | 16.5 |  |
| X:70683773:T | 8 | 6.5 |  |
| X:70683779:A | 1 | 59 |  |
| X:71350182:G | 12 | 10 |  |
| X:71424983:T | 13 | 5 |  |
| X:7171244:T | 10 | 2.5 |  |
| X:7171247:T | 13 | 75 |  |
| X:72783303:A | 4 | 13.5 |  |
| X:76711867:G | 5 | 12 |  |
| X:76849305:T | 9 | 55.5 |  |
| X:77298857:A | 7 | 14 | 50 |
| X:77392419:C | 1 | 2 |  |
| X:7811288:G | 1 | 171 | 23 |
| X:8138270:T | 1 | 53.5 |  |
| X:8138607:T | 10 | 87 |  |
| X:8138641:C | 1 | 17 | 14 |
| X:8138662:A | 13 | 76 |  |
| X:83116204:C | 2 | 17 | 50 |
| X:83361968:A | 8 | 7.5 |  |
| X:83361970:G | 10 | 73 |  |
| X:8433506:C | 13 | 99.5 |  |
| X:8433801:G | 1 | 238 | 18 |
| X:8433833:T | 13 | 156.5 |  |
| X:8433892:A | 13 | 174 |  |
| X:8433892:C | 4 | 72 |  |
| X:8433906:A | 13 | 175 |  |
| X:8434097:T | 7 | 36.5 |  |
| X:8434098:C | 11 | 71 |  |
| X:8434101:A | 12 | 35.5 |  |
| X:8434106:G | 13 | 67 |  |
| X:8434128:G | 9 | 48 |  |
| X:8434191:A | 13 | 57.5 |  |
| X:8434196:G | 13 | 59 |  |
| X:8434199:G | 4 | 62 |  |
| X:8434406:G | 9 | 42 |  |
| X:8434417:C | 13 | 23.5 |  |
| X:8434423:G | 13 | 19 |  |
| X:8763143:C | 7 | 70.5 |  |
| X:8763146:C | 13 | 39.5 |  |
| X:8763149:C | 4 | 16 |  |
| X:8763192:T | 8 | 16.5 |  |
| X:8763195:T | 13 | 37 |  |
| X:8763198:C | 13 | 33 |  |
| X:8763351:T | 13 | 110 |  |
| X:8763354:T | 7 | 131 |  |
| X:92964690:A | 1 | 6.5 |  |
| X:92964690:C | 2 | 8 |  |
| X:9656197:A | 13 | 130 |  |
| X:9905197:C | 12 | 130 |  |
