# Supplementary material for: Recurrent miscalling of missense variation from short-read genome sequence data
Source: BMC Genomics. 2019 Jul 16;20(Suppl 8):546. doi: 10.1186/s12864-019-5863-2 (PMC6631443; doi:10.1186/s12864-019-5863-2)
Supplement: Supplementary file 6 — Table S6. Recurrent false positive variants by population cohort. (DOCX 304 kb) [file 12864_2019_5863_MOESM6_ESM.docx]

**Additional file 6: Table S6** – Recurrent false positive variants by population cohort.

| **Coord** | **1000G** | **Omani** | **Tiwi** |
| --- | --- | --- | --- |
| 7:74212048 | 0 | 0 | 1 |
| X:37029578 | 3 | 0 | 0 |
| 14:22102592 | 0 | 0 | 10 |
| 11:112123095 | 0 | 4 | 0 |
| 1:12779560 | 0 | 0 | 3 |
| 3:14106033 | 0 | 3 | 0 |
| X:153418535 | 3 | 0 | 0 |
| 19:4499642 | 12 | 0 | 3 |
| 6:32632581 | 0 | 0 | 3 |
| 5:57854018 | 0 | 3 | 0 |
| 11:71249158 | 3 | 0 | 0 |
| 6:29910779 | 3 | 2 | 0 |
| 6:31324586 | 0 | 0 | 10 |
| 19:54676763 | 0 | 0 | 6 |
| 4:36340753 | 0 | 2 | 0 |
| 1:144918957 | 0 | 0 | 10 |
| 4:88537312 | 1 | 0 | 0 |
| 11:47788670 | 6 | 0 | 0 |
| 3:75787688 | 0 | 0 | 0 |
| 12:52402998 | 0 | 1 | 0 |
| 19:49000886 | 0 | 2 | 0 |
| 12:123186880 | 0 | 1 | 0 |
| X:153418456 | 13 | 0 | 0 |
| 2:97817666 | 0 | 0 | 0 |
| 16:28649787 | 0 | 2 | 0 |
| 3:75786741 | 0 | 0 | 0 |
| 20:30452782 | 0 | 6 | 0 |
| X:153455691 | 13 | 0 | 0 |
| 6:33179689 | 0 | 0 | 9 |
| 16:29791561 | 0 | 0 | 8 |
| 1:145293510 | 0 | 0 | 5 |
| 19:56274531 | 0 | 2 | 0 |
| 3:14105896 | 0 | 1 | 0 |
| 6:30995030 | 0 | 1 | 0 |
| 1:248524971 | 0 | 0 | 0 |
| 17:21204192 | 0 | 0 | 0 |
| X:103495202 | 11 | 0 | 0 |
| X:140993905 | 16 | 0 | 0 |
| 5:801267 | 0 | 1 | 0 |
| 6:29910774 | 3 | 1 | 0 |
| 19:2050823 | 0 | 3 | 0 |
| 21:30257568 | 0 | 0 | 10 |
| 3:75787869 | 0 | 0 | 0 |
| 17:44828931 | 0 | 0 | 5 |
| 6:32632601 | 0 | 0 | 3 |
| 22:20710850 | 0 | 3 | 0 |
| 4:101108877 | 2 | 0 | 0 |
| 21:30250555 | 0 | 0 | 10 |
| 14:95903310 | 0 | 0 | 0 |
| 1:1577093 | 0 | 2 | 0 |
| 12:133050694 | 0 | 0 | 0 |
| X:92964690 | 3 | 0 | 0 |
| 1:201356006 | 0 | 1 | 0 |
| 8:7272594 | 0 | 0 | 6 |
| 3:97983494 | 2 | 0 | 0 |
| 19:54937862 | 0 | 0 | 4 |
| 9:43876136 | 4 | 1 | 0 |
| X:51075886 | 6 | 0 | 0 |
| 3:170824990 | 0 | 3 | 0 |
| 8:24326313 | 0 | 1 | 0 |
| 19:38377417 | 0 | 2 | 0 |
| 6:32551957 | 0 | 0 | 33 |
| 7:100552739 | 0 | 0 | 0 |
| 6:31084787 | 0 | 0 | 4 |
| 1:248525329 | 12 | 0 | 0 |
| X:55117888 | 9 | 0 | 0 |
| 15:23406993 | 0 | 2 | 0 |
| 4:190884278 | 0 | 0 | 0 |
| 2:26997936 | 0 | 1 | 0 |
| 1:145301793 | 0 | 5 | 0 |
| 4:170678993 | 0 | 7 | 0 |
| 10:88992705 | 2 | 0 | 0 |
| 15:23407163 | 0 | 1 | 0 |
| 2:131129933 | 1 | 0 | 0 |
| 20:36999943 | 0 | 1 | 0 |
| 15:90320161 | 0 | 5 | 0 |
| X:6452461 | 12 | 0 | 0 |
| 15:21071485 | 0 | 1 | 0 |
| 15:74368293 | 0 | 1 | 0 |
| 12:40874785 | 0 | 0 | 0 |
| 14:19562053 | 1 | 0 | 0 |
| 19:54726226 | 0 | 1 | 0 |
| 15:90248819 | 0 | 2 | 0 |
| 19:40368499 | 0 | 0 | 0 |
| 10:47919971 | 0 | 1 | 0 |
| 11:5688940 | 0 | 2 | 0 |
| 4:4276132 | 0 | 0 | 2 |
| 12:112036791 | 0 | 0 | 0 |
| 1:145299792 | 0 | 1 | 0 |
| 19:40400791 | 3 | 0 | 0 |
| 3:75788023 | 0 | 0 | 0 |
| 19:40385078 | 6 | 0 | 0 |
| 1:248616711 | 0 | 0 | 0 |
| 2:131129948 | 1 | 0 | 0 |
| 19:50383591 | 0 | 0 | 9 |
| 19:22363701 | 0 | 0 | 0 |
| 1:248813653 | 2 | 0 | 0 |
| 6:29913037 | 0 | 0 | 3 |
| 16:27974487 | 0 | 7 | 0 |
| 10:17362967 | 0 | 2 | 0 |
| 6:31324201 | 0 | 2 | 0 |
| 10:49388901 | 0 | 1 | 0 |
| 17:61950694 | 0 | 1 | 0 |
| 12:49171288 | 1 | 0 | 0 |
| 15:20740293 | 7 | 0 | 0 |
| X:55117890 | 13 | 0 | 0 |
| 1:16915434 | 0 | 1 | 0 |
| 12:132628412 | 0 | 0 | 0 |
| X:151870050 | 2 | 0 | 0 |
| 17:38975212 | 0 | 0 | 0 |
| 18:19032046 | 1 | 0 | 0 |
| 2:161174693 | 0 | 1 | 0 |
| 4:88537141 | 1 | 0 | 0 |
| 19:47774668 | 4 | 2 | 0 |
| X:306317 | 0 | 0 | 9 |
| 19:39423332 | 1 | 0 | 0 |
| 1:162367103 | 0 | 2 | 0 |
| 5:733926 | 0 | 1 | 0 |
| 4:185655190 | 0 | 3 | 0 |
| 17:20768757 | 0 | 0 | 0 |
| 19:11598120 | 1 | 0 | 0 |
| 1:183596703 | 0 | 1 | 0 |
| 3:75786518 | 0 | 0 | 0 |
| 2:21266783 | 1 | 0 | 0 |
| 1:13695580 | 9 | 0 | 0 |
| 3:75714853 | 0 | 0 | 0 |
| 9:117166338 | 0 | 0 | 5 |
| 11:1651161 | 0 | 2 | 2 |
| 2:96614325 | 0 | 0 | 0 |
| 2:97915895 | 8 | 0 | 0 |
| 6:31239108 | 0 | 0 | 6 |
| 5:145838635 | 0 | 0 | 0 |
| 2:89417028 | 1 | 0 | 0 |
| 6:32122472 | 0 | 0 | 1 |
| X:38145132 | 6 | 0 | 0 |
| 6:89981413 | 0 | 1 | 0 |
| 4:109769942 | 0 | 1 | 0 |
| 6:32557465 | 0 | 1 | 0 |
| 6:123687288 | 0 | 7 | 0 |
| 22:23247082 | 0 | 0 | 10 |
| X:48970565 | 4 | 0 | 0 |
| 15:23690479 | 0 | 0 | 0 |
| 17:39240729 | 5 | 0 | 0 |
| 2:166771798 | 0 | 1 | 0 |
| 17:39305785 | 10 | 3 | 0 |
| 6:31324539 | 1 | 0 | 0 |
| 19:35850711 | 1 | 0 | 0 |
| 1:152084699 | 0 | 0 | 0 |
| 3:195508548 | 0 | 0 | 0 |
| 15:34820194 | 0 | 1 | 0 |
| 2:108455303 | 7 | 0 | 0 |
| 1:12887226 | 1 | 0 | 0 |
| 9:75303653 | 0 | 9 | 0 |
| 11:1264183 | 0 | 1 | 0 |
| 9:114146832 | 0 | 1 | 0 |
| 1:1643740 | 0 | 1 | 0 |
| 5:112824045 | 0 | 0 | 0 |
| X:134947984 | 13 | 0 | 0 |
| 1:204159612 | 1 | 0 | 0 |
| 1:205632220 | 2 | 0 | 0 |
| 22:21742684 | 13 | 1 | 0 |
| 2:113830347 | 0 | 1 | 0 |
| X:2656261 | 0 | 1 | 0 |
| 10:58120988 | 0 | 1 | 0 |
| 22:19420109 | 0 | 6 | 0 |
| 15:93198688 | 16 | 2 | 0 |
| 16:88052214 | 0 | 1 | 0 |
| 9:138151156 | 0 | 1 | 0 |
| 16:70896122 | 0 | 0 | 0 |
| 17:51063068 | 0 | 1 | 0 |
| 4:3590841 | 0 | 0 | 0 |
| 11:65601560 | 0 | 2 | 0 |
| 14:106805212 | 0 | 0 | 0 |
| 17:81009636 | 0 | 2 | 0 |
| 14:106791201 | 0 | 0 | 7 |
| 19:8555875 | 1 | 0 | 0 |
| X:54566663 | 13 | 0 | 0 |
| 6:32549380 | 0 | 1 | 0 |
| 19:49526191 | 0 | 2 | 0 |
| 5:79950750 | 0 | 7 | 0 |
| 4:88536321 | 2 | 0 | 0 |
| 1:152327325 | 1 | 0 | 0 |
| 1:152749004 | 2 | 0 | 0 |
| 7:25266573 | 8 | 0 | 0 |
| 1:248084754 | 2 | 0 | 0 |
| 22:20709312 | 0 | 2 | 0 |
| 20:42939750 | 0 | 0 | 7 |
| X:100531437 | 0 | 2 | 0 |
| 4:15689018 | 5 | 0 | 0 |
| 6:109323519 | 0 | 1 | 0 |
| X:102841886 | 13 | 0 | 0 |
| 6:105291163 | 0 | 1 | 0 |
| 1:26608828 | 0 | 1 | 0 |
| 12:133049751 | 0 | 1 | 0 |
| X:139586512 | 2 | 0 | 0 |
| 3:75714337 | 0 | 0 | 0 |
| X:37850325 | 13 | 0 | 0 |
| 12:52435691 | 0 | 1 | 0 |
| 12:80765800 | 0 | 4 | 0 |
| 16:32077496 | 0 | 0 | 8 |
| 4:88537088 | 0 | 1 | 0 |
| 11:68668037 | 0 | 1 | 0 |
| 1:39977575 | 0 | 1 | 0 |
| 5:118691795 | 0 | 1 | 0 |
| 5:34807928 | 0 | 5 | 0 |
| 7:100641745 | 0 | 1 | 0 |
| 11:7847208 | 0 | 0 | 3 |
| X:153418514 | 9 | 0 | 0 |
| 4:169325251 | 0 | 1 | 0 |
| 9:136518097 | 0 | 3 | 0 |
| 3:150421606 | 0 | 0 | 0 |
| 11:56468198 | 0 | 0 | 0 |
| 2:97827839 | 0 | 0 | 0 |
| 15:33091105 | 1 | 0 | 0 |
| 11:1651211 | 0 | 2 | 0 |
| 16:4930100 | 0 | 2 | 0 |
| 20:43976991 | 0 | 1 | 0 |
| 16:22545285 | 1 | 0 | 0 |
| 13:25440318 | 0 | 1 | 0 |
| 16:71012855 | 0 | 0 | 0 |
| 12:11150240 | 0 | 0 | 5 |
| 1:152185823 | 1 | 0 | 0 |
| 22:28194933 | 0 | 0 | 0 |
| X:153421927 | 13 | 0 | 0 |
| 12:50745863 | 0 | 2 | 0 |
| 6:31973973 | 0 | 3 | 0 |
| 1:1575784 | 2 | 0 | 0 |
| 16:12027476 | 0 | 0 | 0 |
| X:57934520 | 13 | 0 | 0 |
| 12:122685164 | 0 | 0 | 0 |
| 14:22997524 | 0 | 1 | 0 |
| 2:215835049 | 0 | 1 | 0 |
| 1:85742012 | 0 | 7 | 0 |
| 10:51225872 | 0 | 1 | 0 |
| 8:7681350 | 0 | 2 | 0 |
| 8:2040337 | 0 | 0 | 1 |
| 4:156787368 | 0 | 1 | 0 |
| 16:88599701 | 6 | 0 | 0 |
| 19:49526203 | 0 | 1 | 0 |
| 6:32632832 | 0 | 1 | 0 |
| 12:133050700 | 0 | 0 | 0 |
| 19:40376881 | 0 | 1 | 0 |
| 1:12854307 | 0 | 1 | 0 |
| 1:36235455 | 0 | 2 | 0 |
| 6:31322996 | 0 | 0 | 1 |
| 14:76452150 | 0 | 1 | 0 |
| 4:88537117 | 2 | 0 | 0 |
| 1:145293498 | 0 | 0 | 7 |
| 9:136268084 | 0 | 0 | 4 |
| X:27765408 | 3 | 4 | 0 |
| 17:21319860 | 0 | 0 | 1 |
| 6:29364615 | 0 | 0 | 7 |
| 1:153320401 | 0 | 3 | 0 |
| 15:99544418 | 0 | 1 | 0 |
| 14:106363835 | 0 | 1 | 0 |
| 17:41121194 | 0 | 1 | 0 |
| 19:55349200 | 0 | 0 | 0 |
| 17:18291544 | 0 | 5 | 0 |
| 1:10699146 | 2 | 0 | 0 |
| 4:100140306 | 0 | 11 | 0 |
| 1:10555372 | 0 | 1 | 0 |
| 4:88537132 | 4 | 0 | 0 |
| 16:84158248 | 0 | 1 | 0 |
| 1:206773659 | 0 | 0 | 4 |
| 6:32549588 | 0 | 2 | 0 |
| 5:668500 | 0 | 1 | 0 |
| 6:350829 | 0 | 9 | 0 |
| 19:58385536 | 1 | 0 | 0 |
| 4:88536551 | 5 | 0 | 0 |
| 1:13329150 | 0 | 0 | 1 |
| 6:32609195 | 0 | 1 | 0 |
| 4:156787340 | 0 | 11 | 0 |
| 6:32552059 | 0 | 1 | 30 |
| 9:138150721 | 0 | 1 | 0 |
| 1:248801778 | 0 | 1 | 0 |
| X:14861870 | 13 | 0 | 0 |
| 17:34523265 | 0 | 1 | 0 |
| 18:31319487 | 0 | 0 | 0 |
| 17:25931745 | 0 | 1 | 0 |
| 1:1850678 | 1 | 0 | 0 |
| 12:53045615 | 5 | 0 | 0 |
| 9:70177212 | 0 | 1 | 0 |
| 11:56143592 | 0 | 0 | 3 |
| 17:21319767 | 0 | 0 | 1 |
| 22:25425282 | 0 | 0 | 0 |
| 11:71276655 | 0 | 1 | 0 |
| X:49114790 | 4 | 0 | 0 |
| 1:13695600 | 6 | 0 | 0 |
| X:102192787 | 4 | 0 | 0 |
| 1:179504037 | 0 | 0 | 0 |
| 2:87088964 | 0 | 5 | 0 |
| 3:100170628 | 0 | 0 | 0 |
| 1:179457739 | 0 | 0 | 0 |
| 6:31324499 | 1 | 0 | 0 |
| 1:12855845 | 0 | 1 | 0 |
| 2:152350373 | 0 | 1 | 0 |
| 17:43552717 | 0 | 0 | 1 |
| 1:120611964 | 0 | 5 | 7 |
| 6:31324664 | 0 | 0 | 1 |
| 19:54867572 | 0 | 0 | 9 |
| 18:9887416 | 0 | 2 | 0 |
| 4:4198950 | 3 | 0 | 0 |
| 1:148017629 | 0 | 0 | 1 |
| 6:29911063 | 0 | 0 | 7 |
| 1:248722723 | 1 | 0 | 0 |
| 11:56143537 | 0 | 1 | 0 |
| 14:70039824 | 15 | 0 | 0 |
| 1:220603324 | 0 | 5 | 0 |
| 3:113376119 | 0 | 1 | 0 |
| 7:6862979 | 0 | 1 | 0 |
| 9:46386846 | 0 | 1 | 0 |
| 16:24583718 | 0 | 0 | 0 |
| 22:24302486 | 0 | 0 | 0 |
| X:49368274 | 1 | 0 | 0 |
| 16:1279732 | 0 | 1 | 0 |
| 10:93963 | 0 | 1 | 0 |
| 8:38176435 | 0 | 1 | 0 |
| 6:32632608 | 0 | 0 | 8 |
| X:71350182 | 12 | 0 | 0 |
| X:76711867 | 5 | 0 | 0 |
| 6:32489735 | 0 | 1 | 0 |
| 1:12952951 | 2 | 0 | 0 |
| 15:47425885 | 0 | 1 | 0 |
| 1:24671406 | 0 | 1 | 0 |
| 5:156479568 | 0 | 1 | 0 |
| 12:133049776 | 0 | 0 | 0 |
| 4:9274248 | 0 | 2 | 0 |
| 8:12283494 | 0 | 1 | 0 |
| 17:21216846 | 0 | 0 | 0 |
| 9:116973273 | 0 | 2 | 0 |
| 17:38715186 | 0 | 1 | 0 |
| 15:28632820 | 2 | 0 | 0 |
| 11:1017419 | 0 | 1 | 0 |
| 19:54724411 | 4 | 1 | 0 |
| 17:26694447 | 0 | 2 | 0 |
| 1:6529203 | 0 | 1 | 0 |
| 1:226788371 | 1 | 0 | 0 |
| 3:195506323 | 3 | 0 | 0 |
| 17:43922942 | 0 | 0 | 4 |
| 2:240982227 | 2 | 0 | 0 |
| 13:72440664 | 0 | 0 | 0 |
| 11:1642884 | 0 | 0 | 6 |
| X:19554576 | 0 | 1 | 0 |
| 21:47351616 | 0 | 1 | 0 |
| 3:195452951 | 0 | 0 | 2 |
| 11:1265854 | 0 | 1 | 0 |
| 17:77769127 | 0 | 1 | 0 |
| 15:20743796 | 0 | 1 | 1 |
| X:140994096 | 7 | 0 | 0 |
| 3:195508082 | 0 | 0 | 0 |
| 21:47351605 | 5 | 0 | 0 |
| 2:171649402 | 0 | 2 | 0 |
| 19:56274213 | 0 | 1 | 0 |
| 7:105671267 | 0 | 1 | 0 |
| 19:44590001 | 5 | 0 | 0 |
| 12:11183512 | 0 | 0 | 1 |
| 8:133067265 | 0 | 1 | 0 |
| X:140995535 | 6 | 0 | 0 |
| 10:81608302 | 1 | 0 | 0 |
| 13:46108853 | 0 | 11 | 0 |
| 1:13329095 | 0 | 0 | 5 |
| 1:53681699 | 0 | 2 | 0 |
| 3:195452873 | 0 | 1 | 0 |
| X:153455695 | 13 | 0 | 0 |
| 12:40874783 | 0 | 0 | 0 |
| 10:51464617 | 2 | 1 | 0 |
| 12:53343132 | 0 | 1 | 0 |
| X:6452125 | 13 | 0 | 0 |
| 10:46322030 | 0 | 1 | 0 |
| 17:10427944 | 0 | 1 | 0 |
| 17:77769130 | 0 | 1 | 0 |
| 10:27436463 | 4 | 0 | 0 |
| 10:5034045 | 0 | 1 | 0 |
| X:53115059 | 13 | 0 | 0 |
| 9:19622268 | 0 | 9 | 0 |
| 3:9920138 | 0 | 5 | 0 |
| X:49355893 | 0 | 1 | 0 |
| 19:4513038 | 1 | 0 | 0 |
| 3:130122493 | 0 | 1 | 0 |
| 1:12854090 | 0 | 0 | 1 |
| 7:100646418 | 2 | 0 | 0 |
| 1:147955126 | 1 | 0 | 0 |
| 11:4976077 | 0 | 2 | 0 |
| 6:57393125 | 0 | 0 | 10 |
| 12:40875413 | 8 | 0 | 0 |
| 13:38138689 | 0 | 5 | 0 |
| 6:31838441 | 0 | 0 | 1 |
| X:149100781 | 13 | 0 | 0 |
| 19:7011878 | 2 | 0 | 0 |
| 14:106471449 | 0 | 1 | 0 |
| 7:157929370 | 0 | 1 | 0 |
| 11:502144 | 2 | 0 | 0 |
| 12:106641502 | 16 | 0 | 0 |
| 9:65505933 | 0 | 1 | 0 |
| 15:28625673 | 0 | 1 | 0 |
| 15:23685597 | 0 | 0 | 0 |
| 3:75713977 | 0 | 0 | 0 |
| 17:72889676 | 0 | 3 | 0 |
| 11:1092800 | 24 | 0 | 0 |
| 21:10959771 | 0 | 1 | 0 |
| 6:170871058 | 0 | 1 | 0 |
| 3:75787679 | 0 | 0 | 0 |
| 6:27115129 | 0 | 1 | 0 |
| 6:30890483 | 0 | 0 | 7 |
| 6:32489758 | 0 | 1 | 1 |
| 16:70954717 | 0 | 0 | 0 |
| 19:52888079 | 0 | 0 | 0 |
| 12:53189428 | 0 | 1 | 0 |
| 1:207726161 | 0 | 1 | 0 |
| 1:7909738 | 0 | 1 | 0 |
| 19:9801443 | 5 | 0 | 0 |
| 1:203024598 | 0 | 1 | 0 |
| 19:14877845 | 0 | 5 | 0 |
| X:114364721 | 1 | 0 | 0 |
| 22:17445698 | 0 | 1 | 0 |
| 11:118178007 | 0 | 4 | 0 |
| 6:32632592 | 0 | 0 | 2 |
| 11:1092802 | 20 | 0 | 0 |
| 1:148756665 | 3 | 2 | 0 |
| 2:90193417 | 0 | 0 | 8 |
| 16:830767 | 2 | 0 | 0 |
| 19:501719 | 0 | 1 | 0 |
| 2:159663599 | 0 | 1 | 0 |
| 12:40875390 | 0 | 4 | 0 |
| 3:140675438 | 1 | 0 | 0 |
| X:38145351 | 11 | 0 | 0 |
| 14:106111107 | 2 | 0 | 0 |
| 11:117772955 | 0 | 2 | 0 |
| 5:78610466 | 0 | 0 | 0 |
| 9:123476548 | 0 | 0 | 0 |
| 16:1291175 | 0 | 1 | 0 |
| 9:138150809 | 0 | 1 | 0 |
| 3:75787956 | 0 | 0 | 0 |
| 11:71276909 | 0 | 1 | 0 |
| 9:8497250 | 0 | 1 | 0 |
| 1:248813473 | 1 | 0 | 0 |
| 15:99544429 | 0 | 11 | 0 |
| 16:71163693 | 0 | 0 | 0 |
| 2:1946776 | 0 | 0 | 0 |
| 20:1896052 | 5 | 0 | 0 |
| 19:54745508 | 2 | 0 | 0 |
| 14:107178965 | 0 | 0 | 1 |
| X:118920677 | 0 | 1 | 0 |
| 18:9117867 | 0 | 1 | 0 |
| X:100531419 | 0 | 0 | 0 |
| 6:30695920 | 0 | 3 | 0 |
| 8:143833850 | 0 | 1 | 0 |
| 6:32487205 | 0 | 0 | 9 |
| 6:31239613 | 1 | 0 | 0 |
| 19:7011877 | 2 | 0 | 0 |
| 3:75790513 | 0 | 0 | 0 |
| 1:145299787 | 4 | 1 | 0 |
| 10:51371565 | 0 | 0 | 2 |
| 12:111993712 | 0 | 1 | 0 |
| 4:140811075 | 0 | 1 | 0 |
| 11:95825383 | 13 | 7 | 0 |
| 6:29910761 | 0 | 1 | 0 |
| X:38145561 | 1 | 0 | 0 |
| X:151869684 | 11 | 0 | 0 |
| 19:36297966 | 0 | 1 | 0 |
| 16:1291454 | 0 | 3 | 0 |
| 15:32688214 | 5 | 0 | 0 |
| 6:30887988 | 0 | 0 | 7 |
| 19:49657527 | 0 | 1 | 0 |
| X:102612705 | 13 | 0 | 0 |
| 3:195447919 | 3 | 0 | 0 |
| 2:241624544 | 0 | 1 | 0 |
| 19:49558216 | 0 | 3 | 0 |
| 11:56344846 | 2 | 0 | 0 |
| X:149101932 | 7 | 0 | 0 |
| X:150832707 | 13 | 0 | 0 |
| 14:22102333 | 0 | 0 | 4 |
| 6:32609173 | 0 | 2 | 0 |
| 1:13477410 | 0 | 0 | 1 |
| 2:89417024 | 2 | 0 | 0 |
| 22:23263602 | 0 | 12 | 0 |
| 11:48387279 | 0 | 0 | 0 |
| 8:1514009 | 0 | 0 | 6 |
| 3:195506560 | 1 | 0 | 0 |
| 15:23605893 | 0 | 1 | 0 |
| 11:1651178 | 0 | 0 | 0 |
| 21:14982886 | 0 | 0 | 0 |
| 6:137026266 | 0 | 9 | 0 |
| 2:240981555 | 6 | 0 | 0 |
| 1:13052907 | 0 | 0 | 8 |
| 17:44061036 | 0 | 0 | 8 |
| 17:39197609 | 0 | 1 | 0 |
| 12:109017465 | 4 | 0 | 0 |
| 1:20979371 | 0 | 1 | 0 |
| 18:61379838 | 0 | 0 | 2 |
| 19:12541347 | 0 | 1 | 0 |
| 3:195452870 | 0 | 1 | 0 |
| 6:29342825 | 0 | 0 | 7 |
| 7:100549884 | 0 | 0 | 0 |
| 9:138151118 | 0 | 4 | 0 |
| 6:31323945 | 0 | 0 | 1 |
| 2:54482716 | 7 | 3 | 0 |
| 16:30936081 | 0 | 3 | 0 |
| 1:145302704 | 0 | 4 | 0 |
| 1:248737348 | 0 | 1 | 0 |
| 12:50745822 | 0 | 1 | 0 |
| 22:22707312 | 0 | 1 | 0 |
| 6:32552072 | 0 | 1 | 0 |
| 10:88416964 | 0 | 1 | 0 |
| 6:32549583 | 0 | 1 | 0 |
| 17:30183857 | 0 | 1 | 0 |
| 5:115141151 | 0 | 1 | 0 |
| X:135313739 | 2 | 0 | 0 |
| 3:75786080 | 0 | 0 | 0 |
| 1:152185815 | 1 | 0 | 0 |
| 17:21203907 | 0 | 0 | 0 |
| 7:100646127 | 6 | 0 | 0 |
| 4:3076672 | 0 | 1 | 0 |
| 14:60007406 | 0 | 0 | 0 |
| 1:205632253 | 2 | 0 | 0 |
| 2:240946766 | 0 | 5 | 0 |
| X:8763192 | 8 | 0 | 0 |
| 20:36946848 | 0 | 2 | 0 |
| 4:3076665 | 0 | 2 | 0 |
| 19:36348055 | 0 | 0 | 0 |
| 6:32797297 | 0 | 0 | 6 |
| 12:11420589 | 1 | 0 | 0 |
| 22:20708980 | 3 | 1 | 0 |
| 19:55286796 | 0 | 0 | 0 |
| 14:22102293 | 0 | 0 | 1 |
| 1:145301739 | 0 | 2 | 0 |
| 22:20708972 | 3 | 5 | 0 |
| 19:56274506 | 0 | 1 | 0 |
| 17:44073889 | 0 | 0 | 7 |
| 8:22584718 | 0 | 7 | 0 |
| 12:21487544 | 0 | 1 | 0 |
| 1:206566904 | 0 | 1 | 0 |
| 1:13052871 | 0 | 0 | 6 |
| 1:152188940 | 0 | 1 | 0 |
| 15:34673722 | 4 | 0 | 0 |
| 10:81606700 | 0 | 1 | 0 |
| 19:55258808 | 0 | 4 | 0 |
| 6:32552091 | 0 | 1 | 0 |
| 19:6763718 | 0 | 1 | 0 |
| 11:1018169 | 0 | 0 | 0 |
| 16:81685855 | 0 | 1 | 0 |
| 10:49382926 | 0 | 7 | 0 |
| 19:48950010 | 1 | 1 | 0 |
| 6:112508770 | 0 | 0 | 10 |
| 11:65325349 | 3 | 0 | 0 |
| 15:23685913 | 0 | 6 | 0 |
| 6:29910752 | 0 | 1 | 0 |
| 22:18904414 | 0 | 0 | 1 |
| 19:54778581 | 0 | 2 | 0 |
| X:299591 | 0 | 0 | 3 |
| 2:240981520 | 2 | 0 | 0 |
| 15:34674006 | 0 | 4 | 0 |
| 7:123672479 | 2 | 4 | 0 |
| 19:55286650 | 0 | 0 | 0 |
| X:151869339 | 13 | 0 | 0 |
| 11:71249125 | 2 | 0 | 0 |
| 19:54724457 | 1 | 2 | 7 |
| 6:32489766 | 0 | 1 | 0 |
| 16:71015329 | 0 | 0 | 0 |
| 3:43732523 | 0 | 1 | 0 |
| 16:1291178 | 0 | 1 | 0 |
| 10:46999601 | 0 | 5 | 0 |
| 17:25958304 | 0 | 3 | 0 |
| 19:4499647 | 15 | 4 | 8 |
| 12:10041364 | 0 | 12 | 0 |
| 1:13448547 | 0 | 2 | 7 |
| X:132161713 | 13 | 0 | 0 |
| 1:957674 | 0 | 1 | 0 |
| X:139586507 | 2 | 0 | 0 |
| 1:179457796 | 0 | 1 | 0 |
| 1:148344741 | 0 | 0 | 2 |
| 15:22368862 | 0 | 0 | 1 |
| 14:74060514 | 0 | 0 | 0 |
| 10:51464656 | 7 | 0 | 0 |
| 16:16278863 | 0 | 0 | 4 |
| 14:53019985 | 0 | 1 | 0 |
| 3:75786822 | 0 | 0 | 0 |
| 1:145299805 | 0 | 2 | 0 |
| 11:134202036 | 0 | 1 | 0 |
| 18:28898294 | 0 | 10 | 0 |
| 10:51748684 | 1 | 0 | 0 |
| 3:195512949 | 0 | 1 | 0 |
| 3:195515387 | 0 | 0 | 0 |
| 6:32549611 | 5 | 0 | 0 |
| 2:242716380 | 0 | 6 | 0 |
| 6:29910640 | 0 | 0 | 1 |
| 21:47703744 | 0 | 1 | 0 |
| 6:31079236 | 0 | 0 | 8 |
| 9:34725047 | 0 | 1 | 0 |
| 19:868115 | 0 | 0 | 1 |
| 12:123011395 | 1 | 0 | 0 |
| 6:170627767 | 0 | 0 | 0 |
| 12:133050605 | 0 | 0 | 0 |
| 2:111598958 | 0 | 6 | 0 |
| 6:31997401 | 0 | 1 | 0 |
| 21:47351612 | 5 | 2 | 0 |
| 17:4837195 | 0 | 0 | 0 |
| 11:27384466 | 1 | 0 | 0 |
| 3:65342269 | 0 | 0 | 0 |
| 5:70751818 | 0 | 0 | 9 |
| 19:4512939 | 2 | 0 | 0 |
| 15:34820227 | 1 | 0 | 0 |
| 9:131684610 | 0 | 1 | 0 |
| 11:124253170 | 0 | 1 | 0 |
| 19:43709647 | 0 | 2 | 0 |
| 3:75715099 | 0 | 0 | 0 |
| 2:240981519 | 1 | 0 | 0 |
| 16:25239805 | 0 | 6 | 0 |
| 6:42075128 | 0 | 0 | 0 |
| X:3761423 | 1 | 0 | 0 |
| 1:108769307 | 4 | 0 | 0 |
| 16:28507452 | 0 | 1 | 0 |
| 11:117781435 | 0 | 12 | 0 |
| 9:138150740 | 0 | 2 | 0 |
| 11:56143556 | 0 | 2 | 0 |
| 1:161645052 | 0 | 1 | 0 |
| 1:21976289 | 0 | 1 | 0 |
| 6:16326772 | 0 | 1 | 0 |
| X:38145304 | 1 | 0 | 0 |
| 5:74021853 | 4 | 0 | 0 |
| 22:25425340 | 0 | 0 | 0 |
| 1:145348109 | 0 | 0 | 10 |
| 22:26879962 | 0 | 0 | 0 |
| 1:248722671 | 1 | 0 | 0 |
| 19:49573365 | 2 | 0 | 0 |
| 11:4976444 | 0 | 2 | 0 |
| 16:32077580 | 0 | 0 | 6 |
| 6:32006886 | 0 | 0 | 2 |
| 17:21319654 | 0 | 0 | 0 |
| 1:175129946 | 0 | 5 | 0 |
| 22:22707299 | 0 | 0 | 7 |
| 6:32557423 | 0 | 1 | 0 |
| 19:55241107 | 0 | 0 | 0 |
| 6:32370835 | 0 | 0 | 6 |
| X:70683773 | 8 | 0 | 0 |
| 3:125725272 | 2 | 0 | 0 |
| 6:32634300 | 0 | 5 | 0 |
| 6:44224427 | 0 | 1 | 0 |
| X:57162833 | 8 | 0 | 0 |
| 17:39383073 | 0 | 0 | 2 |
| 11:640065 | 0 | 1 | 0 |
| 1:1850654 | 9 | 0 | 0 |
| 8:12043910 | 1 | 0 | 0 |
| 4:88535830 | 0 | 5 | 0 |
| X:57934566 | 13 | 0 | 0 |
| 6:31974857 | 3 | 0 | 0 |
| X:136649003 | 13 | 0 | 0 |
| 8:27094399 | 0 | 1 | 0 |
| 6:32307382 | 0 | 0 | 6 |
| 11:48346541 | 0 | 0 | 0 |
| X:151935747 | 13 | 0 | 0 |
| 1:152327329 | 1 | 0 | 0 |
| 6:32948426 | 0 | 0 | 9 |
| 5:140186990 | 3 | 0 | 0 |
| 12:40874797 | 0 | 0 | 0 |
| 4:2176454 | 0 | 4 | 0 |
| 10:88994452 | 4 | 3 | 0 |
| 14:106805439 | 0 | 0 | 0 |
| 6:32552081 | 0 | 0 | 9 |
| 19:53116975 | 0 | 0 | 0 |
| 4:88536553 | 1 | 0 | 0 |
| 5:140186979 | 3 | 0 | 0 |
| X:70683779 | 1 | 0 | 0 |
| 6:30954729 | 0 | 0 | 1 |
| 6:32634306 | 0 | 5 | 0 |
| 6:74466377 | 0 | 8 | 0 |
| 2:130832927 | 0 | 1 | 0 |
| 7:100552549 | 0 | 0 | 0 |
| 5:145838641 | 0 | 1 | 0 |
| 6:132029857 | 2 | 2 | 0 |
| 4:3230413 | 1 | 0 | 0 |
| 2:118771566 | 0 | 6 | 0 |
| 1:21012575 | 0 | 1 | 0 |
| 15:30696687 | 0 | 1 | 0 |
| 3:195508537 | 0 | 0 | 0 |
| 1:248801610 | 1 | 0 | 0 |
| 12:9085337 | 1 | 0 | 0 |
| 5:135178124 | 0 | 12 | 0 |
| 16:21747633 | 0 | 1 | 0 |
| 7:130418689 | 0 | 0 | 1 |
| X:141291565 | 1 | 0 | 0 |
| 1:201179050 | 0 | 0 | 0 |
| 4:41673604 | 0 | 7 | 0 |
| 14:106805242 | 0 | 1 | 0 |
| 3:195506982 | 0 | 0 | 0 |
| 1:248737454 | 3 | 0 | 0 |
| 1:12779618 | 0 | 0 | 3 |
| 7:100552050 | 0 | 0 | 0 |
| 1:206578650 | 0 | 2 | 0 |
| X:8433506 | 13 | 0 | 0 |
| 6:31324489 | 0 | 2 | 0 |
| 14:56105908 | 0 | 1 | 0 |
| X:37850326 | 6 | 0 | 0 |
| 8:27396208 | 0 | 4 | 0 |
| 6:49712063 | 0 | 1 | 0 |
| 6:32609230 | 1 | 0 | 0 |
| 10:71678058 | 0 | 5 | 0 |
| 6:32489747 | 0 | 1 | 0 |
| 17:36353761 | 0 | 0 | 8 |
| 19:44778796 | 6 | 0 | 0 |
| 1:178745947 | 0 | 1 | 0 |
| 2:132289349 | 4 | 2 | 0 |
| X:34148882 | 0 | 1 | 0 |
| 22:22707638 | 0 | 0 | 1 |
| 19:55317439 | 0 | 1 | 0 |
| 8:68070699 | 0 | 0 | 0 |
| 4:100045616 | 0 | 12 | 0 |
| 11:59271103 | 1 | 0 | 0 |
| X:2656258 | 0 | 1 | 0 |
| 17:43923934 | 0 | 0 | 2 |
| 7:102223333 | 0 | 1 | 0 |
| 11:56468044 | 0 | 0 | 0 |
| 13:32676114 | 0 | 6 | 0 |
| 1:86453307 | 0 | 1 | 0 |
| 13:78146301 | 0 | 1 | 0 |
| 19:40364289 | 0 | 1 | 0 |
| 6:32557478 | 0 | 5 | 0 |
| 4:9250430 | 0 | 1 | 0 |
| 1:248525330 | 12 | 3 | 0 |
| 20:34090361 | 0 | 1 | 0 |
| 6:31324496 | 0 | 2 | 0 |
| 6:31324202 | 0 | 2 | 0 |
| X:34149696 | 6 | 0 | 0 |
| 6:31324536 | 1 | 4 | 2 |
| 3:65433713 | 0 | 1 | 0 |
| 15:20739886 | 0 | 1 | 0 |
| 7:15430486 | 1 | 0 | 0 |
| 1:13038221 | 0 | 4 | 7 |
| 11:48347140 | 0 | 0 | 0 |
| X:153416316 | 8 | 0 | 0 |
| 14:106053321 | 5 | 0 | 0 |
| 1:240370952 | 0 | 2 | 0 |
| 1:248722611 | 2 | 1 | 0 |
| 9:138150725 | 0 | 2 | 0 |
| 7:128587381 | 0 | 7 | 0 |
| 4:157557726 | 0 | 10 | 0 |
| 2:233712229 | 1 | 0 | 0 |
| 22:18835523 | 0 | 3 | 0 |
| 19:54725745 | 1 | 0 | 0 |
| 4:9390708 | 0 | 1 | 0 |
| 16:90095573 | 0 | 7 | 0 |
| 19:35649281 | 0 | 1 | 0 |
| 6:57398270 | 0 | 0 | 0 |
| 1:21806619 | 0 | 1 | 0 |
| 14:106478287 | 5 | 0 | 0 |
| 17:35696804 | 0 | 5 | 9 |
| 19:54724443 | 1 | 0 | 0 |
| X:150832720 | 0 | 1 | 0 |
| 19:56274193 | 0 | 1 | 0 |
| 19:14200109 | 0 | 1 | 0 |
| X:54783870 | 13 | 0 | 0 |
| 8:139712355 | 0 | 2 | 0 |
| 7:100643060 | 0 | 2 | 0 |
| 11:122774946 | 0 | 1 | 0 |
| 1:100618008 | 1 | 0 | 0 |
| 11:4976654 | 1 | 0 | 0 |
| 19:2820105 | 0 | 1 | 0 |
| 1:13671653 | 0 | 0 | 1 |
| 19:22363719 | 0 | 0 | 0 |
| 7:102182021 | 0 | 1 | 0 |
| 1:26628196 | 0 | 1 | 0 |
| 7:95800820 | 0 | 7 | 0 |
| 19:4513032 | 1 | 0 | 0 |
| 1:150280546 | 0 | 0 | 10 |
| 1:26608883 | 0 | 1 | 0 |
| 12:40876352 | 3 | 0 | 0 |
| 20:29631562 | 0 | 1 | 0 |
| 14:22476146 | 3 | 1 | 0 |
| X:48759546 | 13 | 0 | 0 |
| 22:32205632 | 0 | 1 | 0 |
| 6:29364643 | 0 | 0 | 6 |
| 2:89417043 | 3 | 0 | 0 |
| X:37981362 | 8 | 0 | 0 |
| 6:32552029 | 0 | 1 | 0 |
| 16:3170253 | 1 | 0 | 0 |
| X:72783303 | 4 | 0 | 0 |
| X:140995538 | 13 | 0 | 0 |
| 20:31768363 | 0 | 1 | 0 |
| 17:74288410 | 0 | 1 | 0 |
| 5:176013823 | 0 | 1 | 0 |
| 6:168459845 | 0 | 1 | 0 |
| 5:162890953 | 0 | 0 | 6 |
| 6:123658776 | 0 | 1 | 0 |
| 1:110235888 | 3 | 0 | 0 |
| 6:167592576 | 0 | 0 | 0 |
| 7:134225827 | 0 | 12 | 0 |
| 1:52499093 | 2 | 0 | 0 |
| 5:140186980 | 3 | 0 | 0 |
| 15:32888763 | 0 | 1 | 0 |
| 15:65134221 | 0 | 1 | 0 |
| 19:40392482 | 0 | 0 | 2 |
| 9:139849847 | 0 | 2 | 0 |
| 4:47839929 | 0 | 2 | 0 |
| 6:166739646 | 0 | 4 | 0 |
| 12:101368625 | 0 | 1 | 0 |
| 3:195509498 | 0 | 0 | 0 |
| 15:22473042 | 1 | 0 | 1 |
| 8:39862881 | 0 | 4 | 0 |
| 19:43269705 | 0 | 4 | 0 |
| 6:31122482 | 0 | 0 | 2 |
| 16:70975667 | 0 | 0 | 0 |
| 7:101837149 | 0 | 6 | 0 |
| 20:36977970 | 0 | 0 | 10 |
| 1:40236815 | 0 | 1 | 0 |
| 22:21481271 | 0 | 0 | 3 |
| 15:74002006 | 0 | 1 | 0 |
| 6:112508769 | 0 | 0 | 10 |
| 19:40389657 | 0 | 2 | 0 |
| 6:32497961 | 2 | 0 | 0 |
| 3:195507858 | 5 | 0 | 0 |
| 11:77921062 | 1 | 0 | 0 |
| 9:72755129 | 2 | 0 | 0 |
| 2:131377707 | 13 | 3 | 0 |
| 17:39394717 | 0 | 2 | 0 |
| 19:56241265 | 0 | 0 | 10 |
| 22:20709304 | 0 | 2 | 0 |
| X:34148842 | 0 | 0 | 0 |
| 2:188250301 | 0 | 12 | 1 |
| 1:1423267 | 0 | 3 | 0 |
| 4:88537315 | 1 | 0 | 0 |
| 5:145838662 | 0 | 1 | 0 |
| X:8433801 | 0 | 1 | 0 |
| 17:36493598 | 0 | 0 | 10 |
| 4:145041708 | 1 | 1 | 0 |
| 8:62412054 | 0 | 0 | 0 |
| X:57619121 | 8 | 0 | 0 |
| 8:12286609 | 0 | 1 | 0 |
| 11:64591972 | 0 | 6 | 0 |
| 17:39305775 | 1 | 0 | 0 |
| 1:248605374 | 0 | 1 | 0 |
| 14:22476143 | 3 | 0 | 0 |
| 2:73496597 | 0 | 0 | 0 |
| 2:227662524 | 4 | 0 | 0 |
| 9:138151062 | 0 | 1 | 0 |
| 19:54746591 | 1 | 0 | 0 |
| 6:32609299 | 0 | 3 | 0 |
| 22:24917980 | 0 | 1 | 0 |
| 6:150239449 | 0 | 0 | 0 |
| 5:78610460 | 0 | 0 | 0 |
| 14:23549896 | 2 | 0 | 0 |
| 5:109190938 | 7 | 0 | 0 |
| 19:54746051 | 0 | 1 | 0 |
| 3:75787520 | 0 | 0 | 0 |
| 4:70898905 | 0 | 1 | 0 |
| 21:31655212 | 0 | 0 | 10 |
| 7:102230654 | 0 | 1 | 0 |
| 15:28947419 | 0 | 1 | 0 |
| 8:119964052 | 0 | 11 | 0 |
| 12:40875415 | 8 | 0 | 0 |
| 14:106926223 | 0 | 0 | 7 |
| 1:148343777 | 0 | 0 | 8 |
| 11:56468493 | 0 | 0 | 0 |
| 12:53167395 | 0 | 2 | 0 |
| 1:152191444 | 0 | 0 | 0 |
| 12:42624073 | 0 | 0 | 0 |
| 6:32549344 | 0 | 1 | 0 |
| 12:133633898 | 0 | 0 | 0 |
| 3:190106073 | 3 | 0 | 0 |
| 13:108518715 | 0 | 1 | 0 |
| 19:22156856 | 0 | 0 | 0 |
| 22:20710847 | 0 | 2 | 0 |
| 7:100552536 | 0 | 0 | 0 |
| 1:167893759 | 0 | 7 | 0 |
| 18:14124359 | 0 | 2 | 0 |
| X:27765420 | 13 | 0 | 0 |
| 7:11871480 | 1 | 0 | 0 |
| 17:36290036 | 0 | 0 | 1 |
| 18:49147 | 0 | 0 | 0 |
| 5:833910 | 0 | 1 | 0 |
| 17:21319302 | 0 | 0 | 1 |
| 11:56468449 | 0 | 0 | 0 |
| 3:195515470 | 0 | 0 | 0 |
| 16:31196423 | 0 | 1 | 0 |
| 1:68947838 | 0 | 1 | 0 |
| 9:74674278 | 1 | 0 | 0 |
| 17:26699206 | 0 | 0 | 0 |
| 17:15449158 | 7 | 0 | 0 |
| 1:144916748 | 0 | 0 | 2 |
| 7:130418744 | 0 | 0 | 6 |
| 17:29159404 | 0 | 1 | 0 |
| X:34148844 | 0 | 0 | 0 |
| 15:42386630 | 0 | 1 | 0 |
| 6:31324526 | 1 | 1 | 0 |
| 12:95774672 | 0 | 3 | 0 |
| 1:153907315 | 1 | 0 | 0 |
| 2:171678625 | 0 | 1 | 0 |
| 22:18727099 | 0 | 1 | 0 |
| 10:15719621 | 0 | 1 | 0 |
| X:57934586 | 13 | 0 | 0 |
| 8:6500544 | 0 | 5 | 0 |
| 3:75786521 | 0 | 0 | 0 |
| 1:12835984 | 0 | 1 | 0 |
| 1:92163682 | 0 | 1 | 0 |
| X:153459057 | 8 | 0 | 0 |
| 17:43923703 | 0 | 0 | 3 |
| 1:20416322 | 1 | 0 | 0 |
| 9:138150754 | 0 | 2 | 0 |
| X:8434101 | 12 | 0 | 0 |
| 19:14877857 | 0 | 5 | 0 |
| 1:248604542 | 0 | 1 | 0 |
| 4:337637 | 0 | 0 | 2 |
| 16:71956517 | 0 | 0 | 0 |
| 9:100616706 | 0 | 1 | 0 |
| 17:21318821 | 0 | 0 | 1 |
| 4:88537078 | 0 | 1 | 0 |
| 16:67229817 | 0 | 1 | 0 |
| 7:32209425 | 0 | 0 | 10 |
| 6:31974849 | 0 | 3 | 0 |
| 17:44061025 | 0 | 0 | 9 |
| 1:16915357 | 0 | 0 | 0 |
| 21:46534729 | 1 | 0 | 0 |
| 10:124768583 | 0 | 4 | 0 |
| 19:501695 | 0 | 0 | 0 |
| 1:248524992 | 0 | 2 | 0 |
| 2:131221499 | 0 | 1 | 0 |
| 6:32552112 | 0 | 0 | 1 |
| 16:89264532 | 2 | 0 | 0 |
| 1:146404787 | 8 | 0 | 0 |
| 3:195511884 | 0 | 0 | 0 |
| 6:32552132 | 0 | 0 | 1 |
| X:125955451 | 13 | 0 | 0 |
| 6:32609222 | 1 | 0 | 0 |
| 1:152186490 | 1 | 0 | 0 |
| 20:45179196 | 0 | 2 | 0 |
| 1:149885190 | 1 | 0 | 0 |
| 15:82555242 | 0 | 3 | 0 |
| 2:47045321 | 0 | 1 | 0 |
| 3:75787267 | 0 | 0 | 0 |
| 14:19553642 | 0 | 2 | 0 |
| 12:11214145 | 0 | 0 | 3 |
| 2:11853913 | 0 | 1 | 0 |
| 17:39535388 | 0 | 0 | 9 |
| X:8763198 | 13 | 0 | 0 |
| 14:88862529 | 0 | 4 | 0 |
| 15:32686365 | 0 | 0 | 0 |
| 1:147955246 | 2 | 0 | 0 |
| 19:40392360 | 2 | 2 | 0 |
| 11:60971069 | 0 | 4 | 0 |
| 16:1291318 | 0 | 7 | 0 |
| 4:175899091 | 0 | 1 | 0 |
| 17:6406883 | 0 | 7 | 0 |
| 7:75052079 | 2 | 0 | 0 |
| 1:12854188 | 0 | 0 | 6 |
| 16:1291160 | 0 | 3 | 0 |
| 16:22269867 | 0 | 0 | 1 |
| 6:31322980 | 0 | 0 | 3 |
| 22:20710820 | 0 | 2 | 0 |
| X:56591811 | 13 | 0 | 0 |
| 17:39197605 | 0 | 3 | 0 |
| 1:120612014 | 0 | 1 | 0 |
| 5:64097145 | 0 | 6 | 0 |
| X:151821277 | 0 | 0 | 3 |
| 6:32551953 | 1 | 0 | 0 |
| 5:1878160 | 0 | 1 | 0 |
| 3:75787486 | 0 | 0 | 0 |
| X:148627329 | 13 | 0 | 0 |
| 17:21217513 | 0 | 0 | 0 |
| X:8434098 | 11 | 0 | 0 |
| 1:92457843 | 0 | 12 | 0 |
| 11:1651643 | 0 | 0 | 4 |
| 17:38975259 | 0 | 0 | 0 |
| 1:235652513 | 0 | 0 | 10 |
| 2:21266782 | 1 | 0 | 0 |
| 2:240982219 | 2 | 0 | 0 |
| X:148628334 | 2 | 0 | 0 |
| 1:86557967 | 0 | 6 | 0 |
| X:134947986 | 13 | 0 | 0 |
| 8:144623574 | 0 | 2 | 0 |
| 1:152975816 | 3 | 0 | 0 |
| 6:32634301 | 0 | 1 | 0 |
| 7:5997641 | 0 | 3 | 0 |
| 22:25424579 | 0 | 0 | 0 |
| 3:75787416 | 0 | 0 | 0 |
| 12:122064785 | 16 | 3 | 0 |
| 6:32629146 | 0 | 0 | 6 |
| 1:148341812 | 0 | 2 | 0 |
| 6:32497917 | 0 | 1 | 0 |
| 6:31322911 | 0 | 0 | 5 |
| 18:14524966 | 0 | 3 | 0 |
| 11:65325348 | 2 | 0 | 0 |
| 22:24325095 | 10 | 1 | 0 |
| 15:34331127 | 0 | 0 | 0 |
| 2:90121845 | 1 | 0 | 0 |
| 14:55864130 | 0 | 2 | 0 |
| 12:25368462 | 0 | 0 | 9 |
| 6:31324538 | 1 | 0 | 0 |
| 10:51859758 | 0 | 1 | 0 |
| 8:101719121 | 0 | 1 | 0 |
| 7:100552738 | 0 | 0 | 0 |
| 12:52631313 | 0 | 1 | 0 |
| 17:74288567 | 10 | 0 | 0 |
| 19:1825930 | 3 | 0 | 0 |
| 9:136421044 | 0 | 0 | 0 |
| 7:134618477 | 0 | 0 | 0 |
| 12:51620072 | 0 | 0 | 0 |
| 21:46924430 | 0 | 0 | 0 |
| 2:233712227 | 0 | 6 | 0 |
| 2:96610413 | 0 | 0 | 0 |
| 3:123458823 | 0 | 1 | 0 |
| 14:106478242 | 1 | 0 | 0 |
| 16:70955079 | 0 | 0 | 0 |
| 10:17869577 | 5 | 0 | 0 |
| 9:138151119 | 0 | 1 | 0 |
| 9:138150727 | 0 | 2 | 0 |
| 12:133049463 | 0 | 0 | 0 |
| 2:70129800 | 1 | 4 | 0 |
| 6:31324525 | 1 | 1 | 0 |
| 1:7890053 | 0 | 1 | 0 |
| 15:22473061 | 3 | 0 | 0 |
| 12:52844400 | 1 | 0 | 0 |
| 21:44589215 | 0 | 0 | 9 |
| 6:29910771 | 4 | 1 | 0 |
| 11:1018374 | 0 | 0 | 7 |
| 6:32609169 | 0 | 1 | 0 |
| 19:56284428 | 0 | 1 | 0 |
| 20:3649640 | 5 | 0 | 0 |
| 6:32609214 | 2 | 0 | 0 |
| 16:74640753 | 1 | 0 | 0 |
| 10:4868534 | 0 | 1 | 0 |
| 6:30994026 | 0 | 1 | 0 |
| 11:56237605 | 0 | 0 | 0 |
| 3:75787728 | 0 | 0 | 0 |
| 1:13450798 | 0 | 0 | 2 |
| 18:5410574 | 0 | 2 | 0 |
| 11:123754844 | 0 | 0 | 8 |
| 19:54725755 | 2 | 0 | 0 |
| 22:24459438 | 0 | 0 | 4 |
| 11:1016991 | 0 | 0 | 0 |
| X:153418541 | 0 | 1 | 0 |
| 12:9994450 | 15 | 1 | 0 |
| 19:56284396 | 12 | 2 | 0 |
| 10:88416963 | 0 | 1 | 0 |
| 16:32077537 | 0 | 0 | 8 |
| 6:31964321 | 0 | 1 | 0 |
| 19:52096039 | 1 | 0 | 0 |
| 1:16909100 | 0 | 2 | 0 |
| 1:13448551 | 0 | 2 | 3 |
| 3:75787637 | 0 | 0 | 0 |
| 2:187559050 | 0 | 5 | 0 |
| 1:2488153 | 0 | 1 | 0 |
| 8:103573015 | 0 | 1 | 0 |
| X:140994109 | 12 | 0 | 0 |
| 17:21215552 | 0 | 0 | 0 |
| 3:75787497 | 0 | 0 | 0 |
| 1:1423281 | 0 | 1 | 0 |
| X:153455679 | 13 | 0 | 0 |
| 6:32557489 | 0 | 2 | 0 |
| 1:13038201 | 0 | 2 | 10 |
| 12:40874776 | 0 | 0 | 0 |
| 14:77493806 | 0 | 0 | 0 |
| 2:230456547 | 1 | 0 | 0 |
| X:2847299 | 0 | 1 | 0 |
| 1:179457772 | 0 | 0 | 0 |
| 19:55869903 | 0 | 0 | 0 |
| 5:176005563 | 1 | 0 | 0 |
| 5:79950717 | 0 | 0 | 0 |
| X:13680924 | 13 | 0 | 0 |
| 12:53189427 | 0 | 1 | 0 |
| 20:37667182 | 0 | 5 | 0 |
| 6:32489806 | 0 | 1 | 0 |
| 12:25656706 | 0 | 2 | 0 |
| 11:36440836 | 0 | 1 | 0 |
| 2:176995288 | 0 | 1 | 0 |
| 20:52611552 | 0 | 1 | 0 |
| 1:120572547 | 0 | 0 | 2 |
| X:37028415 | 0 | 1 | 0 |
| 3:75786490 | 0 | 0 | 0 |
| 3:14105897 | 0 | 6 | 0 |
| 2:113258916 | 0 | 3 | 0 |
| 16:863372 | 0 | 2 | 0 |
| 19:55349189 | 0 | 0 | 0 |
| 22:20907454 | 0 | 1 | 0 |
| 6:32497962 | 1 | 0 | 0 |
| 12:11183217 | 0 | 0 | 2 |
| 19:55045042 | 0 | 0 | 7 |
| 15:20739924 | 0 | 0 | 3 |
| 4:80905990 | 0 | 9 | 0 |
| 4:88536917 | 0 | 1 | 0 |
| 19:43709656 | 0 | 10 | 0 |
| 1:207700165 | 0 | 1 | 0 |
| 4:140811108 | 0 | 2 | 0 |
| 21:10942928 | 0 | 1 | 0 |
| 15:82387862 | 0 | 2 | 0 |
| X:140993932 | 0 | 3 | 0 |
| 1:53712727 | 0 | 5 | 0 |
| X:49179711 | 11 | 0 | 0 |
| 2:131129934 | 1 | 0 | 0 |
| 1:231413275 | 0 | 1 | 0 |
| 6:32632833 | 0 | 1 | 0 |
| 7:74223500 | 0 | 3 | 0 |
| 3:195515411 | 0 | 4 | 0 |
| 11:48387115 | 0 | 0 | 0 |
| 4:88535818 | 0 | 1 | 0 |
| 11:1080883 | 0 | 0 | 0 |
| 19:55329901 | 0 | 0 | 7 |
| 4:185655192 | 0 | 3 | 0 |
| 10:51748683 | 1 | 0 | 0 |
| 1:67131957 | 1 | 0 | 0 |
| 10:97106165 | 0 | 3 | 0 |
| 17:39197499 | 0 | 1 | 0 |
| 17:74288421 | 0 | 7 | 0 |
| 12:133049516 | 0 | 4 | 0 |
| 7:5949726 | 0 | 4 | 0 |
| 9:138150753 | 0 | 4 | 0 |
| 3:195509443 | 0 | 0 | 0 |
| 14:22476147 | 5 | 0 | 0 |
| 5:140563763 | 0 | 0 | 10 |
| 1:139296 | 0 | 0 | 3 |
| 17:1733399 | 0 | 11 | 0 |
| 21:46021494 | 0 | 0 | 1 |
| 16:82203768 | 0 | 8 | 0 |
| 19:20044900 | 1 | 0 | 0 |
| 1:75037864 | 4 | 0 | 0 |
| 11:1093710 | 0 | 0 | 2 |
| X:153171699 | 10 | 0 | 0 |
| 19:54744722 | 0 | 1 | 0 |
| 1:248084749 | 2 | 0 | 0 |
| 6:32551958 | 0 | 1 | 0 |
| 14:106805440 | 0 | 0 | 0 |
| 11:56468212 | 0 | 0 | 0 |
| 1:248802469 | 0 | 4 | 0 |
| 10:15719604 | 0 | 1 | 0 |
| 1:148756648 | 13 | 1 | 1 |
| 9:140262426 | 0 | 6 | 0 |
| X:151935469 | 13 | 0 | 0 |
| 19:38377414 | 0 | 2 | 0 |
| 17:46608203 | 1 | 0 | 0 |
| 19:34872382 | 0 | 1 | 0 |
| 17:43552921 | 0 | 0 | 4 |
| 6:31324003 | 0 | 0 | 30 |
| 19:55349273 | 0 | 0 | 0 |
| 11:48346999 | 0 | 0 | 0 |
| 19:58361433 | 0 | 1 | 0 |
| 12:131323639 | 0 | 1 | 0 |
| 3:111603970 | 1 | 0 | 0 |
| 15:43922923 | 0 | 1 | 0 |
| 5:668499 | 0 | 1 | 0 |
| 8:10466019 | 0 | 2 | 0 |
| 19:40368496 | 0 | 0 | 0 |
| 22:20780031 | 3 | 7 | 2 |
| 14:106926287 | 0 | 0 | 5 |
| X:140994031 | 0 | 1 | 0 |
| 8:139642974 | 0 | 1 | 0 |
| X:57620760 | 13 | 0 | 0 |
| 13:25670726 | 0 | 1 | 0 |
| 1:21807427 | 0 | 2 | 0 |
| 5:79950708 | 0 | 7 | 0 |
| 1:248637256 | 1 | 0 | 0 |
| 17:43545893 | 0 | 0 | 8 |
| 16:88787610 | 2 | 0 | 0 |
| 22:25435959 | 0 | 0 | 0 |
| 17:5087041 | 3 | 0 | 0 |
| 2:232325435 | 0 | 1 | 0 |
| 5:177163580 | 0 | 1 | 0 |
| 11:7656809 | 0 | 1 | 0 |
| 11:22881002 | 0 | 6 | 0 |
| 1:52306066 | 10 | 2 | 0 |
| X:134948034 | 13 | 0 | 0 |
| 11:122646967 | 0 | 3 | 0 |
| 15:23685598 | 0 | 0 | 0 |
| 11:134202031 | 0 | 1 | 0 |
| 22:38506509 | 0 | 12 | 0 |
| 8:139647262 | 0 | 3 | 0 |
| 14:21557021 | 0 | 4 | 0 |
| 15:23606545 | 0 | 2 | 0 |
| 8:133854771 | 1 | 0 | 0 |
| 6:32497970 | 6 | 1 | 0 |
| 6:31238930 | 0 | 0 | 1 |
| 12:113717990 | 0 | 1 | 0 |
| 11:1264742 | 5 | 0 | 0 |
| 6:32497910 | 0 | 2 | 0 |
| 22:43193603 | 0 | 2 | 0 |
| 6:42123317 | 0 | 8 | 0 |
| 19:40364088 | 0 | 1 | 0 |
| 1:148756607 | 0 | 0 | 1 |
| 19:56423927 | 0 | 0 | 0 |
| 4:4198954 | 1 | 0 | 0 |
| 3:195505247 | 0 | 1 | 0 |
| 19:55144186 | 0 | 0 | 1 |
| 11:7847472 | 0 | 0 | 7 |
| 14:72945012 | 0 | 1 | 0 |
| 4:3590683 | 0 | 0 | 0 |
| 1:146400257 | 1 | 0 | 0 |
| 7:100680463 | 1 | 0 | 0 |
| 9:112542787 | 0 | 1 | 0 |
| 20:37001761 | 0 | 1 | 0 |
| 8:145999619 | 4 | 0 | 0 |
| 19:43269699 | 0 | 4 | 0 |
| 10:75000739 | 3 | 0 | 0 |
| X:101576798 | 13 | 0 | 0 |
| 6:121620608 | 0 | 1 | 0 |
| 3:75787876 | 0 | 0 | 0 |
| 6:70963124 | 0 | 3 | 0 |
| 21:30458220 | 0 | 1 | 0 |
| 1:86375654 | 0 | 12 | 0 |
| 15:23690475 | 0 | 0 | 0 |
| 17:38975158 | 0 | 3 | 0 |
| X:140994486 | 13 | 0 | 0 |
| 1:206603535 | 0 | 1 | 0 |
| 12:53343133 | 0 | 1 | 0 |
| 10:71657272 | 0 | 3 | 0 |
| 6:30882634 | 0 | 0 | 3 |
| 7:134264286 | 0 | 11 | 0 |
| 4:88536886 | 0 | 4 | 0 |
| 19:58437740 | 0 | 0 | 0 |
| 8:10530218 | 0 | 8 | 0 |
| 1:12952917 | 1 | 0 | 0 |
| 14:106053293 | 1 | 0 | 0 |
| 13:25671163 | 0 | 5 | 0 |
| 1:179457776 | 0 | 0 | 0 |
| 7:100646055 | 5 | 0 | 0 |
| 4:185615700 | 2 | 0 | 0 |
| X:295231 | 0 | 0 | 5 |
| 2:68794483 | 0 | 1 | 0 |
| 20:3065274 | 0 | 0 | 0 |
| 2:89924018 | 0 | 0 | 0 |
| 19:14877820 | 0 | 1 | 0 |
| 16:84063130 | 0 | 1 | 0 |
| 22:44083353 | 0 | 0 | 0 |
| X:151935356 | 13 | 0 | 0 |
| 19:501846 | 0 | 1 | 0 |
| 10:44878713 | 0 | 1 | 0 |
| 16:1290965 | 0 | 1 | 0 |
| 15:74366897 | 0 | 1 | 0 |
| X:57934577 | 11 | 0 | 0 |
| X:57162747 | 2 | 0 | 0 |
| 14:22555182 | 0 | 0 | 1 |
| 8:144295183 | 0 | 0 | 10 |
| 2:85549868 | 0 | 5 | 0 |
| 8:25323777 | 0 | 0 | 10 |
| 19:40392585 | 1 | 0 | 0 |
| 6:32632820 | 0 | 1 | 0 |
| X:153418486 | 9 | 0 | 0 |
| 19:49894179 | 0 | 4 | 0 |
| 17:43923654 | 0 | 0 | 2 |
| 6:32552117 | 0 | 1 | 0 |
| X:107462966 | 0 | 1 | 0 |
| 9:69247550 | 0 | 7 | 0 |
| 3:75714840 | 0 | 0 | 0 |
| X:140967157 | 13 | 0 | 0 |
| 11:89774363 | 1 | 0 | 0 |
| 2:238499866 | 0 | 6 | 0 |
| 11:56344847 | 2 | 0 | 0 |
| 8:77765301 | 0 | 0 | 0 |
| 6:32632593 | 0 | 0 | 3 |
| 22:41716695 | 0 | 1 | 0 |
| 4:177106011 | 3 | 0 | 0 |
| 11:124750455 | 9 | 1 | 0 |
| 7:142499071 | 0 | 0 | 8 |
| 3:75787199 | 0 | 0 | 0 |
| X:140270784 | 13 | 0 | 0 |
| 7:100552657 | 0 | 0 | 0 |
| 3:195515403 | 0 | 1 | 0 |
| 12:40876719 | 0 | 3 | 0 |
| 2:242163366 | 3 | 0 | 0 |
| 12:118506348 | 8 | 0 | 0 |
| 10:17362979 | 0 | 1 | 0 |
| 11:89536930 | 0 | 1 | 0 |
| 20:238441 | 0 | 0 | 0 |
| 5:179070463 | 0 | 1 | 0 |
| 17:4837204 | 0 | 1 | 0 |
| 10:3214942 | 0 | 1 | 0 |
| 9:43625044 | 1 | 0 | 0 |
| 15:74001993 | 0 | 4 | 0 |
| 7:142498735 | 0 | 0 | 6 |
| 14:106791200 | 0 | 0 | 6 |
| 15:65042560 | 0 | 1 | 0 |
| 11:48346547 | 0 | 0 | 0 |
| 5:73148481 | 0 | 8 | 0 |
| X:54841980 | 1 | 0 | 0 |
| 14:105352890 | 5 | 0 | 0 |
| 2:233697779 | 0 | 0 | 0 |
| 4:88537285 | 4 | 0 | 0 |
| 7:144070338 | 1 | 0 | 0 |
| 1:21806624 | 0 | 1 | 0 |
| 5:140574175 | 0 | 1 | 0 |
| 1:144917841 | 0 | 0 | 5 |
| 6:29365241 | 0 | 0 | 9 |
| 6:32549356 | 0 | 1 | 8 |
| 10:13166076 | 0 | 0 | 7 |
| 10:81063786 | 0 | 1 | 0 |
| 8:144911463 | 0 | 1 | 0 |
| 14:23018607 | 0 | 1 | 0 |
| 7:100551840 | 0 | 0 | 0 |
| 11:132200049 | 0 | 1 | 0 |
| 21:43169357 | 0 | 3 | 0 |
| X:135313788 | 6 | 0 | 0 |
| 15:23264144 | 0 | 0 | 1 |
| 12:40876752 | 0 | 1 | 0 |
| X:140994010 | 0 | 0 | 0 |
| 22:18835221 | 0 | 0 | 1 |
| 9:43876040 | 6 | 4 | 0 |
| 6:31864547 | 0 | 0 | 3 |
| 11:89531619 | 0 | 2 | 0 |
| 1:248814126 | 8 | 0 | 0 |
| 1:13036330 | 0 | 0 | 1 |
| 1:12854105 | 0 | 0 | 1 |
| 15:23684962 | 0 | 1 | 0 |
| 1:13329354 | 0 | 0 | 6 |
| 17:43924231 | 0 | 0 | 4 |
| 4:265333 | 0 | 2 | 0 |
| 17:6515291 | 0 | 0 | 0 |
| 22:20457932 | 0 | 1 | 0 |
| 17:39383012 | 0 | 0 | 7 |
| 3:15637914 | 0 | 1 | 0 |
| 9:136433542 | 0 | 0 | 0 |
| X:57619114 | 5 | 0 | 0 |
| 13:114325815 | 1 | 0 | 0 |
| 8:104940095 | 0 | 1 | 0 |
| 19:56274503 | 0 | 3 | 0 |
| 19:4512910 | 2 | 0 | 0 |
| 11:71544269 | 0 | 6 | 0 |
| 14:106478292 | 5 | 0 | 0 |
| 19:1081936 | 0 | 1 | 0 |
| 12:51740409 | 5 | 0 | 0 |
| 1:26608885 | 0 | 4 | 0 |
| 6:25420350 | 0 | 1 | 0 |
| 9:69238268 | 0 | 3 | 0 |
| 19:52888080 | 0 | 0 | 0 |
| X:13681506 | 13 | 0 | 0 |
| 6:32557506 | 0 | 4 | 0 |
| 17:64023642 | 0 | 3 | 0 |
| 17:21202237 | 0 | 0 | 0 |
| 7:100552251 | 0 | 0 | 0 |
| 11:1651169 | 0 | 0 | 0 |
| 9:40706117 | 0 | 2 | 0 |
| X:134872271 | 0 | 0 | 7 |
| 3:195508547 | 0 | 0 | 0 |
| 19:2015568 | 1 | 0 | 0 |
| 11:95825206 | 1 | 0 | 0 |
| X:57619166 | 13 | 0 | 0 |
| 9:43129577 | 0 | 1 | 0 |
| 1:152749042 | 4 | 0 | 0 |
| 1:17248532 | 0 | 0 | 8 |
| X:140994120 | 13 | 0 | 0 |
| 20:31892670 | 0 | 1 | 0 |
| 1:112018657 | 0 | 2 | 0 |
| 9:82187750 | 0 | 4 | 0 |
| 5:24487856 | 0 | 1 | 0 |
| 20:18167977 | 0 | 0 | 10 |
| 5:40852451 | 0 | 0 | 3 |
| 2:166032778 | 0 | 3 | 0 |
| 17:39261851 | 0 | 1 | 0 |
| 19:40400766 | 1 | 2 | 0 |
| 7:32529936 | 0 | 8 | 0 |
| 1:13450014 | 0 | 0 | 9 |
| 14:88852166 | 0 | 6 | 0 |
| 2:187559053 | 0 | 3 | 0 |
| 14:106478280 | 2 | 0 | 0 |
| 15:22473054 | 2 | 0 | 0 |
| 5:65317181 | 0 | 2 | 0 |
| 8:7320252 | 0 | 0 | 8 |
| 2:26804247 | 0 | 7 | 0 |
| 6:32632795 | 0 | 0 | 1 |
| 6:32552078 | 0 | 1 | 0 |
| 4:48988450 | 0 | 4 | 0 |
| 1:145359110 | 1 | 0 | 0 |
| 5:118485784 | 0 | 1 | 0 |
| 4:140811111 | 0 | 3 | 0 |
| 22:28194927 | 0 | 0 | 0 |
| 10:81270319 | 0 | 8 | 0 |
| X:103495061 | 1 | 0 | 0 |
| 16:85948098 | 0 | 2 | 0 |
| 17:35771468 | 0 | 12 | 0 |
| 9:37711382 | 0 | 1 | 0 |
| 17:47921426 | 1 | 0 | 0 |
| 3:195506558 | 1 | 0 | 0 |
| 8:144940230 | 0 | 0 | 1 |
| 15:93198687 | 16 | 1 | 0 |
| 11:46724722 | 5 | 0 | 0 |
| 6:31762844 | 0 | 0 | 8 |
| 3:121351315 | 0 | 6 | 0 |
| 6:32557477 | 0 | 2 | 0 |
| 1:152186422 | 0 | 1 | 0 |
| 18:48723154 | 0 | 1 | 0 |
| 13:77736068 | 0 | 1 | 0 |
| 7:100645825 | 2 | 0 | 0 |
| 11:48347342 | 0 | 0 | 0 |
| 6:167791530 | 0 | 1 | 0 |
| 7:100552311 | 0 | 0 | 0 |
| 12:56184353 | 0 | 1 | 0 |
| 1:146406560 | 0 | 0 | 10 |
| 16:12027475 | 0 | 0 | 0 |
| 17:39406104 | 0 | 0 | 5 |
| 1:248637115 | 0 | 2 | 0 |
| 14:107179013 | 0 | 0 | 0 |
| 19:56599452 | 6 | 0 | 0 |
| 6:31324641 | 0 | 0 | 2 |
| 19:9646898 | 0 | 2 | 0 |
| 14:67671485 | 0 | 0 | 0 |
| 6:42233518 | 0 | 1 | 0 |
| 2:130832292 | 0 | 1 | 0 |
| 22:50921164 | 5 | 0 | 0 |
| 7:6013049 | 0 | 4 | 0 |
| 5:140558314 | 0 | 4 | 0 |
| 7:115614254 | 0 | 1 | 0 |
| 6:31839331 | 0 | 0 | 9 |
| 6:32634303 | 0 | 5 | 0 |
| X:49179715 | 1 | 0 | 0 |
| 2:209214770 | 0 | 3 | 0 |
| 7:100643575 | 0 | 1 | 0 |
| 11:56468200 | 0 | 0 | 0 |
| 15:23686207 | 0 | 1 | 0 |
| 21:46057620 | 0 | 1 | 0 |
| 3:75715087 | 0 | 0 | 0 |
| 11:1017041 | 0 | 0 | 0 |
| 9:7046901 | 0 | 2 | 0 |
| 1:144823868 | 0 | 2 | 9 |
| 16:55866957 | 0 | 0 | 0 |
| 17:20370767 | 4 | 3 | 0 |
| 7:94946084 | 0 | 2 | 0 |
| 19:55236747 | 1 | 0 | 0 |
| 3:75787540 | 0 | 0 | 0 |
| 8:10467653 | 0 | 3 | 0 |
| 1:21945530 | 0 | 1 | 0 |
| 19:36355595 | 0 | 1 | 0 |
| 2:97817661 | 0 | 0 | 0 |
| 3:195515388 | 0 | 1 | 0 |
| 16:1279908 | 0 | 1 | 0 |
| 10:124356566 | 1 | 2 | 0 |
| 11:48387107 | 0 | 0 | 0 |
| 20:13976448 | 0 | 2 | 0 |
| 19:40374034 | 0 | 0 | 5 |
| 3:137981393 | 0 | 1 | 0 |
| 7:100552436 | 0 | 0 | 0 |
| 11:94800751 | 0 | 0 | 0 |
| X:140994197 | 4 | 0 | 0 |
| 1:151700081 | 0 | 1 | 0 |
| X:19045359 | 0 | 1 | 0 |
| 13:20006620 | 0 | 1 | 0 |
| 19:4512958 | 1 | 1 | 0 |
| 11:59271101 | 1 | 0 | 0 |
| 1:120612006 | 0 | 1 | 9 |
| 7:100552535 | 0 | 0 | 0 |
| X:6451804 | 13 | 0 | 0 |
| 15:30437693 | 0 | 1 | 0 |
| 16:28507424 | 0 | 2 | 0 |
| 22:22707309 | 0 | 0 | 6 |
| 17:6515292 | 0 | 0 | 0 |
| X:49076221 | 1 | 0 | 0 |
| 3:195508523 | 0 | 0 | 0 |
| 9:138150852 | 0 | 1 | 0 |
| 17:44626773 | 4 | 1 | 0 |
| 2:111753546 | 1 | 0 | 0 |
| 1:148343679 | 0 | 1 | 0 |
| 4:88537060 | 1 | 0 | 0 |
| 18:60492683 | 1 | 0 | 0 |
| X:151869731 | 13 | 0 | 0 |
| 6:7563983 | 0 | 9 | 1 |
| 9:93978356 | 0 | 1 | 0 |
| 17:60351457 | 0 | 3 | 0 |
| 8:10467636 | 0 | 1 | 0 |
| 1:143767547 | 0 | 0 | 6 |
| 1:12918986 | 0 | 0 | 1 |
| 19:40368495 | 0 | 0 | 0 |
| 14:106791119 | 0 | 0 | 7 |
| 4:88537574 | 0 | 0 | 0 |
| 1:1452615 | 1 | 0 | 0 |
| X:102612709 | 3 | 0 | 0 |
| 6:10928643 | 0 | 1 | 0 |
| 5:843815 | 0 | 1 | 0 |
| 11:56468554 | 0 | 0 | 8 |
| 1:117150628 | 0 | 0 | 0 |
| 18:59810563 | 0 | 6 | 0 |
| 7:72413593 | 0 | 0 | 2 |
| 6:31324495 | 0 | 3 | 0 |
| 1:16903912 | 0 | 3 | 0 |
| 2:68765137 | 0 | 0 | 0 |
| 3:75714298 | 0 | 0 | 0 |
| 4:88536457 | 7 | 0 | 0 |
| 6:30856726 | 0 | 1 | 0 |
| 15:75581966 | 1 | 0 | 0 |
| 1:12954852 | 1 | 0 | 0 |
| 1:171688351 | 0 | 1 | 0 |
| 6:32552075 | 0 | 1 | 0 |
| 1:148004625 | 0 | 0 | 0 |
| X:7171247 | 13 | 0 | 0 |
| 10:98115019 | 0 | 1 | 0 |
| 19:11437480 | 0 | 1 | 0 |
| 11:56468448 | 0 | 0 | 0 |
| 11:1265627 | 2 | 0 | 0 |
| 17:44060775 | 0 | 0 | 8 |
| 17:19608773 | 0 | 1 | 0 |
| 9:40706227 | 0 | 1 | 0 |
| 5:180485353 | 0 | 3 | 0 |
| 2:234761225 | 0 | 2 | 0 |
| 1:13474788 | 1 | 0 | 0 |
| 21:46057621 | 0 | 1 | 0 |
| 19:54664752 | 0 | 0 | 4 |
| 3:69299233 | 0 | 1 | 0 |
| 3:111356083 | 0 | 1 | 0 |
| 10:88782100 | 0 | 1 | 0 |
| X:140994472 | 7 | 0 | 0 |
| 2:233712223 | 0 | 0 | 0 |
| 19:55284986 | 0 | 0 | 0 |
| 2:95847047 | 16 | 2 | 1 |
| X:83361970 | 10 | 0 | 0 |
| 12:52307113 | 0 | 1 | 0 |
| 18:67534642 | 0 | 1 | 0 |
| 3:195508550 | 0 | 0 | 0 |
| 11:56468047 | 0 | 0 | 0 |
| 16:12021361 | 0 | 0 | 0 |
| 4:145041707 | 1 | 1 | 0 |
| 16:70902568 | 0 | 0 | 0 |
| 9:33796694 | 4 | 0 | 0 |
| 6:31323321 | 0 | 0 | 8 |
| 8:12286191 | 0 | 1 | 0 |
| 11:65383896 | 0 | 0 | 10 |
| X:140993947 | 0 | 1 | 0 |
| 13:45148705 | 0 | 1 | 0 |
| 6:31324516 | 0 | 2 | 6 |
| 19:501701 | 0 | 0 | 0 |
| 22:26879967 | 0 | 6 | 0 |
| X:37028425 | 0 | 1 | 0 |
| 19:55147510 | 0 | 0 | 2 |
| 22:29885594 | 0 | 2 | 0 |
| 19:7051437 | 1 | 0 | 0 |
| 19:44778181 | 0 | 1 | 0 |
| 19:54745550 | 3 | 0 | 0 |
| 5:115341638 | 0 | 7 | 0 |
| 7:72413443 | 0 | 0 | 1 |
| 16:10524659 | 13 | 2 | 0 |
| 21:36042478 | 1 | 4 | 0 |
| 6:54054686 | 0 | 12 | 0 |
| 6:29910717 | 0 | 0 | 2 |
| 3:195511956 | 2 | 0 | 0 |
| 11:48387535 | 0 | 0 | 0 |
| 9:135105964 | 0 | 12 | 0 |
| 13:108518717 | 1 | 0 | 0 |
| X:140994113 | 4 | 0 | 0 |
| 17:44248769 | 0 | 0 | 5 |
| 19:50484234 | 0 | 6 | 0 |
| 17:15604485 | 0 | 1 | 0 |
| 22:20708961 | 0 | 1 | 0 |
| 11:60971694 | 0 | 6 | 0 |
| 6:32632745 | 0 | 1 | 0 |
| 6:32552092 | 0 | 1 | 4 |
| 19:54744210 | 0 | 0 | 1 |
| 6:32632749 | 0 | 1 | 0 |
| 17:26684392 | 16 | 4 | 3 |
| 19:46519369 | 2 | 0 | 0 |
| 14:77493794 | 2 | 0 | 0 |
| 12:11174276 | 0 | 0 | 9 |
| 15:74536404 | 7 | 2 | 0 |
| 4:88536880 | 0 | 0 | 0 |
| X:48269495 | 2 | 0 | 0 |
| 6:31324552 | 3 | 0 | 0 |
| 12:40876741 | 0 | 1 | 0 |
| 11:71276702 | 8 | 0 | 0 |
| 17:37815326 | 1 | 0 | 0 |
| 11:56468694 | 0 | 0 | 0 |
| 1:12942941 | 0 | 0 | 0 |
| 19:56274507 | 0 | 1 | 0 |
| 19:55349299 | 0 | 0 | 0 |
| 19:49558211 | 0 | 3 | 0 |
| 8:69143589 | 0 | 4 | 0 |
| 4:7941204 | 0 | 1 | 0 |
| 9:33386469 | 0 | 0 | 0 |
| 1:1569451 | 0 | 1 | 0 |
| 5:94772540 | 0 | 0 | 8 |
| 1:146418342 | 0 | 0 | 8 |
| 2:240981940 | 2 | 0 | 0 |
| 5:110079450 | 0 | 1 | 0 |
| 17:4837192 | 0 | 0 | 0 |
| 2:9983686 | 0 | 7 | 0 |
| 7:74193642 | 0 | 1 | 0 |
| 1:244228389 | 1 | 0 | 0 |
| 16:1290948 | 0 | 1 | 0 |
| 2:160136337 | 0 | 0 | 10 |
| 17:42927721 | 0 | 1 | 0 |
| 19:24116560 | 3 | 0 | 0 |
| 4:9388088 | 0 | 1 | 0 |
| 3:184039775 | 0 | 0 | 0 |
| 1:26608879 | 0 | 4 | 0 |
| 4:48165757 | 0 | 1 | 0 |
| 1:146400239 | 1 | 0 | 0 |
| 11:56143561 | 0 | 3 | 0 |
| 3:14105893 | 0 | 2 | 0 |
| 6:167590574 | 0 | 1 | 0 |
| X:153490660 | 13 | 0 | 0 |
| 11:71276948 | 0 | 1 | 0 |
| 10:18138656 | 1 | 0 | 0 |
| 2:89891207 | 0 | 1 | 0 |
| 6:31324208 | 0 | 4 | 3 |
| 7:102235769 | 0 | 0 | 0 |
| 2:175202208 | 0 | 1 | 0 |
| 3:195509476 | 0 | 0 | 0 |
| 19:54778587 | 0 | 2 | 0 |
| 2:217498287 | 0 | 0 | 0 |
| 4:88537114 | 2 | 0 | 0 |
| X:151869356 | 2 | 0 | 0 |
| 1:111957561 | 0 | 0 | 0 |
| 3:118865132 | 3 | 0 | 0 |
| 3:130318621 | 0 | 1 | 0 |
| 9:117033022 | 0 | 2 | 0 |
| 13:24436475 | 0 | 1 | 0 |
| 16:85689986 | 1 | 0 | 0 |
| 15:84651452 | 2 | 0 | 0 |
| 7:6005323 | 1 | 0 | 0 |
| 2:46707884 | 0 | 1 | 0 |
| 5:179072006 | 0 | 1 | 0 |
| 22:21064271 | 0 | 0 | 0 |
| 7:150069404 | 6 | 0 | 0 |
| 1:12853530 | 0 | 0 | 2 |
| 11:124750453 | 0 | 7 | 0 |
| X:149681040 | 13 | 0 | 0 |
| 5:10235368 | 0 | 1 | 0 |
| 6:32634318 | 0 | 4 | 1 |
| 9:138151078 | 0 | 1 | 0 |
| 12:40874775 | 0 | 0 | 0 |
| 20:33734991 | 0 | 1 | 0 |
| 11:58346943 | 0 | 0 | 0 |
| 3:75787927 | 0 | 0 | 0 |
| 10:93841227 | 0 | 0 | 4 |
| 12:132547093 | 2 | 0 | 0 |
| 3:158388780 | 0 | 1 | 0 |
| 2:207603234 | 0 | 4 | 0 |
| 8:62412053 | 0 | 0 | 0 |
| X:148627336 | 13 | 0 | 0 |
| 14:92537379 | 0 | 0 | 0 |
| 22:36598058 | 0 | 8 | 0 |
| 17:15510988 | 0 | 2 | 0 |
| 8:117783995 | 2 | 0 | 0 |
| 6:32487175 | 0 | 0 | 4 |
| 11:4968175 | 0 | 0 | 0 |
| 2:189940142 | 0 | 3 | 0 |
| 1:12854097 | 0 | 0 | 1 |
| 1:148015634 | 0 | 0 | 6 |
| 3:75787186 | 0 | 0 | 0 |
| 6:30075903 | 0 | 0 | 1 |
| 16:85690000 | 0 | 1 | 0 |
| 15:66850103 | 0 | 1 | 0 |
| 9:138151225 | 0 | 1 | 0 |
| 19:34012655 | 0 | 1 | 0 |
| 4:71468348 | 5 | 0 | 0 |
| 1:152327810 | 2 | 0 | 0 |
| 3:101443534 | 0 | 1 | 0 |
| 1:20411332 | 0 | 4 | 0 |
| 4:77087421 | 0 | 1 | 0 |
| 6:32486344 | 1 | 0 | 3 |
| X:151935918 | 13 | 0 | 0 |
| 1:148741720 | 0 | 4 | 6 |
| 3:75786737 | 0 | 0 | 0 |
| 3:58625875 | 0 | 5 | 0 |
| 11:1959707 | 0 | 2 | 0 |
| 5:137088945 | 0 | 0 | 0 |
| 19:52888075 | 0 | 0 | 0 |
| 18:49549 | 0 | 3 | 0 |
| 1:111957553 | 0 | 0 | 0 |
| 1:55014013 | 0 | 2 | 0 |
| 11:1266479 | 0 | 1 | 0 |
| 22:21064212 | 0 | 0 | 0 |
| X:18348758 | 13 | 0 | 0 |
| X:34148841 | 0 | 0 | 0 |
| 5:140186984 | 3 | 0 | 0 |
| 13:31309784 | 0 | 1 | 0 |
| 1:143767522 | 0 | 0 | 8 |
| 17:17697102 | 0 | 1 | 0 |
| 2:242716387 | 0 | 1 | 0 |
| 11:4976659 | 1 | 0 | 0 |
| 6:32557479 | 0 | 4 | 0 |
| 7:121653377 | 1 | 0 | 0 |
| 16:85689988 | 3 | 0 | 0 |
| 2:228111435 | 0 | 10 | 0 |
| 2:132019302 | 0 | 1 | 0 |
| 1:17658130 | 13 | 2 | 0 |
| 1:16915513 | 0 | 2 | 0 |
| 4:337638 | 0 | 0 | 3 |
| 15:23259531 | 0 | 1 | 0 |
| 17:56056607 | 1 | 1 | 0 |
| 4:68829109 | 0 | 4 | 0 |
| 1:2255569 | 0 | 1 | 0 |
| 14:77493809 | 0 | 1 | 0 |
| 7:43810764 | 0 | 8 | 0 |
| 7:131241049 | 0 | 0 | 0 |
| 6:32489755 | 0 | 1 | 0 |
| 22:22707639 | 0 | 0 | 1 |
| 19:56284464 | 0 | 1 | 0 |
| X:153418516 | 8 | 0 | 0 |
| 9:138150737 | 0 | 2 | 0 |
| X:103495208 | 12 | 0 | 0 |
| 16:57702274 | 0 | 1 | 0 |
| 1:145296372 | 0 | 0 | 6 |
| 7:100552044 | 0 | 0 | 0 |
| 17:44110532 | 0 | 0 | 1 |
| 14:106478243 | 1 | 0 | 0 |
| 3:4825554 | 0 | 1 | 0 |
| 6:32497960 | 1 | 0 | 0 |
| 7:100550079 | 0 | 0 | 4 |
| 9:37492679 | 0 | 2 | 0 |
| 12:52696930 | 0 | 1 | 0 |
| X:51076081 | 3 | 0 | 0 |
| 11:123306172 | 0 | 1 | 0 |
| 6:32549548 | 1 | 0 | 0 |
| 1:248722659 | 0 | 1 | 0 |
| 3:75713980 | 0 | 0 | 0 |
| X:34149684 | 9 | 0 | 0 |
| 1:148343676 | 0 | 1 | 0 |
| 1:98348885 | 0 | 0 | 10 |
| 1:152975812 | 4 | 0 | 0 |
| 8:87226642 | 7 | 0 | 0 |
| 16:69988472 | 0 | 2 | 0 |
| 12:40876939 | 0 | 0 | 0 |
| 6:170627695 | 0 | 0 | 0 |
| 5:134907552 | 0 | 1 | 0 |
| 1:26608843 | 0 | 2 | 0 |
| 17:39305774 | 1 | 0 | 0 |
| 6:42185564 | 0 | 9 | 0 |
| 4:88536460 | 2 | 0 | 0 |
| 14:19574244 | 0 | 1 | 0 |
| X:57619118 | 8 | 0 | 0 |
| 6:32628022 | 0 | 5 | 0 |
| 1:12952968 | 2 | 0 | 0 |
| 6:31704068 | 2 | 0 | 0 |
| 15:23685904 | 0 | 3 | 0 |
| X:150840799 | 5 | 0 | 0 |
| 1:145349628 | 0 | 1 | 0 |
| X:153455644 | 2 | 0 | 0 |
| 21:45970774 | 6 | 0 | 0 |
| 4:56874517 | 0 | 2 | 0 |
| 1:247978544 | 4 | 0 | 0 |
| 1:12887220 | 3 | 0 | 0 |
| 1:248605124 | 0 | 1 | 0 |
| X:134947967 | 12 | 0 | 0 |
| 4:190884280 | 0 | 0 | 0 |
| 2:240982285 | 1 | 0 | 0 |
| 4:88537072 | 1 | 1 | 0 |
| 2:17962998 | 1 | 0 | 0 |
| 14:94847285 | 0 | 0 | 5 |
| 17:21208413 | 0 | 0 | 0 |
| X:134947963 | 7 | 0 | 0 |
| 6:150343148 | 0 | 1 | 0 |
| 12:7045897 | 0 | 1 | 0 |
| 22:39537381 | 0 | 0 | 0 |
| 19:53116979 | 0 | 0 | 0 |
| X:150840698 | 13 | 0 | 0 |
| 13:61102581 | 3 | 0 | 0 |
| 11:48346579 | 0 | 0 | 0 |
| 21:45588136 | 0 | 1 | 0 |
| 17:61973865 | 0 | 1 | 0 |
| 17:44067382 | 0 | 0 | 6 |
| 7:100552978 | 0 | 0 | 0 |
| 19:11998777 | 0 | 1 | 0 |
| 1:13523852 | 0 | 0 | 10 |
| 6:32549589 | 0 | 0 | 3 |
| 11:124267178 | 0 | 1 | 0 |
| 22:17265124 | 2 | 5 | 0 |
| 19:20044903 | 3 | 0 | 0 |
| 6:32549376 | 0 | 2 | 0 |
| 1:156514215 | 0 | 1 | 0 |
| 15:69652451 | 0 | 2 | 0 |
| 14:23000062 | 0 | 3 | 0 |
| 8:90949268 | 0 | 1 | 0 |
| 7:100549942 | 0 | 0 | 0 |
| 22:26157068 | 0 | 1 | 0 |
| 1:205889329 | 0 | 1 | 0 |
| X:57936433 | 12 | 1 | 0 |
| X:140994462 | 1 | 0 | 0 |
| 7:104110492 | 0 | 5 | 0 |
| 3:123458811 | 0 | 1 | 0 |
| 7:11871481 | 1 | 0 | 0 |
| 12:97098549 | 0 | 9 | 0 |
| 17:36339584 | 0 | 1 | 0 |
| 15:43814989 | 1 | 0 | 0 |
| 17:44061278 | 0 | 0 | 6 |
| 1:46774783 | 0 | 4 | 0 |
| 1:148021591 | 1 | 0 | 0 |
| 6:32945686 | 1 | 0 | 0 |
| 1:214564340 | 0 | 2 | 0 |
| 1:144855812 | 0 | 0 | 2 |
| 14:92537364 | 0 | 1 | 0 |
| 1:244999023 | 0 | 1 | 0 |
| 19:14910446 | 1 | 0 | 0 |
| 3:14105843 | 0 | 1 | 0 |
| 9:96438998 | 8 | 0 | 0 |
| 1:206647742 | 0 | 0 | 2 |
| 4:145041686 | 0 | 5 | 0 |
| 7:72413423 | 0 | 0 | 8 |
| 6:32609298 | 0 | 0 | 0 |
| X:153418524 | 8 | 0 | 0 |
| 5:135178142 | 0 | 4 | 0 |
| 4:177605086 | 0 | 0 | 0 |
| 6:32609312 | 0 | 8 | 5 |
| 1:210003489 | 0 | 2 | 0 |
| X:6451793 | 13 | 0 | 0 |
| 16:863358 | 6 | 0 | 0 |
| 14:74060515 | 0 | 0 | 0 |
| X:46359390 | 13 | 0 | 0 |
| 7:100646414 | 3 | 0 | 0 |
| 6:30954909 | 0 | 0 | 2 |
| 16:71026076 | 0 | 0 | 0 |
| 4:88536550 | 3 | 0 | 0 |
| 15:72955443 | 0 | 1 | 0 |
| 17:4837171 | 0 | 4 | 0 |
| 8:12291593 | 0 | 2 | 0 |
| 15:82932833 | 0 | 2 | 0 |
| 5:472055 | 0 | 1 | 0 |
| 19:20044898 | 1 | 0 | 0 |
| 12:22063115 | 0 | 0 | 10 |
| 17:7077747 | 0 | 1 | 0 |
| 19:54664209 | 0 | 0 | 1 |
| 1:201180054 | 1 | 0 | 0 |
| 9:132630668 | 15 | 3 | 0 |
| 2:100343557 | 0 | 10 | 0 |
| 5:39141240 | 0 | 1 | 0 |
| 16:72822033 | 0 | 1 | 0 |
| X:112022630 | 7 | 0 | 0 |
| 4:3590911 | 0 | 0 | 0 |
| 4:110791090 | 0 | 0 | 0 |
| X:134948014 | 13 | 0 | 0 |
| 19:877030 | 0 | 1 | 0 |
| 6:29911261 | 0 | 0 | 3 |
| 6:30071330 | 0 | 0 | 3 |
| 3:195509497 | 0 | 0 | 0 |
| 8:103573019 | 22 | 3 | 0 |
| 9:46386994 | 0 | 0 | 0 |
| X:38146032 | 13 | 0 | 0 |
| 11:56143544 | 0 | 1 | 0 |
| 19:41628014 | 0 | 1 | 0 |
| 5:177159074 | 1 | 0 | 0 |
| 2:228217260 | 0 | 1 | 0 |
| 14:74992800 | 0 | 10 | 0 |
| 9:140123265 | 1 | 0 | 0 |
| 6:31540556 | 0 | 0 | 1 |
| 2:96614313 | 0 | 0 | 0 |
| 9:138150825 | 0 | 1 | 0 |
| 16:4445327 | 0 | 8 | 0 |
| 2:89161072 | 0 | 0 | 10 |
| 22:24300102 | 3 | 0 | 0 |
| 1:240370932 | 0 | 4 | 0 |
| 21:45538647 | 0 | 0 | 9 |
| 7:76129758 | 0 | 4 | 0 |
| 10:93099 | 0 | 0 | 0 |
| 20:61463522 | 0 | 6 | 0 |
| X:49114792 | 13 | 0 | 0 |
| 1:145366857 | 0 | 0 | 2 |
| X:49103224 | 0 | 0 | 3 |
| 17:44071294 | 0 | 0 | 7 |
| 11:48347142 | 0 | 0 | 0 |
| 6:31324210 | 0 | 2 | 0 |
| 1:160209892 | 1 | 0 | 0 |
| 6:38650628 | 0 | 1 | 0 |
| 9:88937856 | 13 | 0 | 0 |
| 11:47788669 | 2 | 0 | 0 |
| 9:138150742 | 0 | 2 | 0 |
| 14:106926311 | 0 | 0 | 2 |
| 19:5831816 | 0 | 1 | 0 |
| 6:32557486 | 0 | 1 | 0 |
| 12:11461584 | 0 | 2 | 0 |
| 3:195497205 | 0 | 1 | 0 |
| X:140994136 | 23 | 0 | 0 |
| 2:71170807 | 0 | 3 | 0 |
| 6:32609297 | 0 | 0 | 0 |
| 13:24322265 | 0 | 1 | 0 |
| 1:1580524 | 2 | 1 | 5 |
| 16:71487952 | 0 | 3 | 0 |
| 6:30856760 | 0 | 1 | 0 |
| 6:32549596 | 0 | 1 | 0 |
| 15:48807637 | 0 | 0 | 10 |
| X:26212262 | 10 | 0 | 0 |
| 16:21416509 | 0 | 1 | 0 |
| 12:9315209 | 0 | 7 | 0 |
| X:6451873 | 9 | 0 | 0 |
| 1:248604943 | 2 | 0 | 0 |
| 17:43924219 | 0 | 0 | 2 |
| 11:56143725 | 0 | 0 | 0 |
| X:140785696 | 0 | 1 | 0 |
| 3:75787918 | 0 | 0 | 0 |
| 7:148936878 | 0 | 4 | 0 |
| 22:25023506 | 2 | 1 | 0 |
| 21:40584598 | 0 | 12 | 0 |
| 2:88081797 | 0 | 1 | 0 |
| 17:21204187 | 0 | 0 | 0 |
| 6:31610686 | 0 | 0 | 2 |
| 10:5260682 | 0 | 1 | 0 |
| 1:144882581 | 0 | 0 | 5 |
| 12:47168898 | 0 | 0 | 3 |
| 22:18727100 | 0 | 1 | 0 |
| 6:32552144 | 0 | 0 | 1 |
| 10:88992622 | 0 | 1 | 0 |
| 6:32298372 | 0 | 10 | 7 |
| 12:7080212 | 16 | 7 | 8 |
| 1:145281543 | 0 | 0 | 2 |
| 6:31324666 | 0 | 0 | 5 |
| 1:12953166 | 0 | 0 | 7 |
| 7:94030899 | 0 | 3 | 0 |
| 21:47351607 | 5 | 0 | 0 |
| 19:15276781 | 0 | 1 | 0 |
| 12:109017673 | 0 | 1 | 0 |
| 5:60200665 | 0 | 1 | 0 |
| 11:1093342 | 0 | 2 | 0 |
| 6:31080016 | 0 | 0 | 3 |
| 15:55700981 | 0 | 4 | 0 |
| 17:34624839 | 0 | 1 | 0 |
| X:6451813 | 13 | 0 | 0 |
| 8:143833856 | 0 | 1 | 0 |
| 10:46965727 | 0 | 0 | 1 |
| 1:1991014 | 0 | 5 | 0 |
| X:140993886 | 10 | 0 | 0 |
| 1:16902884 | 0 | 3 | 0 |
| 12:133634450 | 0 | 0 | 0 |
| 6:32714168 | 0 | 0 | 1 |
| 11:60291413 | 0 | 2 | 0 |
| 5:180485374 | 0 | 1 | 0 |
| 6:167791549 | 0 | 2 | 0 |
| 16:68373862 | 0 | 1 | 0 |
| X:37028389 | 0 | 1 | 0 |
| 12:112036756 | 0 | 0 | 0 |
| 1:144917546 | 0 | 0 | 6 |
| 17:43924073 | 0 | 0 | 2 |
| X:8434128 | 9 | 0 | 0 |
| 1:179876988 | 0 | 9 | 0 |
| 11:121403229 | 0 | 0 | 10 |
| 6:32489749 | 0 | 1 | 0 |
| 6:32825090 | 0 | 0 | 4 |
| 14:106354418 | 0 | 3 | 0 |
| 3:75787962 | 0 | 0 | 0 |
| 18:31319489 | 0 | 0 | 0 |
| 14:106478296 | 5 | 0 | 0 |
| 11:1271223 | 2 | 0 | 0 |
| 9:79318381 | 16 | 0 | 0 |
| 16:70926334 | 0 | 0 | 0 |
| 6:43250734 | 5 | 1 | 0 |
| 11:56468020 | 0 | 0 | 0 |
| 1:25747230 | 0 | 4 | 0 |
| 1:248604614 | 0 | 0 | 0 |
| 22:20709237 | 0 | 2 | 0 |
| X:148627327 | 10 | 0 | 0 |
| 19:38202516 | 0 | 4 | 0 |
| 18:71928150 | 0 | 5 | 0 |
| 6:32725062 | 0 | 10 | 0 |
| 11:118430519 | 0 | 4 | 0 |
| 5:79950724 | 0 | 3 | 0 |
| 21:36042462 | 3 | 1 | 0 |
| 11:3661585 | 0 | 1 | 0 |
| 16:70954774 | 0 | 0 | 0 |
| 19:55349190 | 0 | 0 | 0 |
| 6:29797639 | 0 | 0 | 2 |
| 11:1018116 | 0 | 0 | 0 |
| 16:70989299 | 0 | 0 | 0 |
| 19:54726628 | 0 | 1 | 0 |
| 11:18195364 | 0 | 0 | 0 |
| 16:12021353 | 0 | 0 | 0 |
| X:153455665 | 13 | 0 | 0 |
| 11:44089249 | 0 | 2 | 0 |
| 6:44140054 | 1 | 0 | 0 |
| 4:88537162 | 3 | 0 | 0 |
| 16:83981825 | 0 | 1 | 0 |
| 6:111310252 | 0 | 1 | 0 |
| 9:107367666 | 14 | 0 | 0 |
| 13:25670730 | 0 | 1 | 0 |
| 14:32419331 | 0 | 5 | 0 |
| 22:42911257 | 0 | 6 | 0 |
| 2:96610410 | 0 | 0 | 0 |
| 6:32549582 | 0 | 1 | 0 |
| 7:74212036 | 0 | 0 | 4 |
| 12:97098555 | 0 | 1 | 0 |
| 19:56423928 | 0 | 0 | 0 |
| 3:75787996 | 0 | 0 | 0 |
| 6:42075131 | 0 | 0 | 0 |
| 6:41754576 | 0 | 1 | 0 |
| 16:1306681 | 0 | 2 | 0 |
| 3:195508534 | 0 | 0 | 0 |
| 11:3721864 | 0 | 1 | 0 |
| 12:6450945 | 0 | 7 | 0 |
| 1:144811823 | 0 | 3 | 0 |
| X:151870055 | 3 | 0 | 0 |
| 19:54724430 | 0 | 1 | 0 |
| X:14861875 | 13 | 0 | 0 |
| X:140993945 | 0 | 2 | 0 |
| 12:48439141 | 0 | 2 | 0 |
| 18:19032056 | 1 | 0 | 0 |
| 10:46321555 | 0 | 1 | 0 |
| 1:220603312 | 0 | 0 | 0 |
| 19:55485899 | 0 | 6 | 0 |
| 19:52133292 | 1 | 0 | 0 |
| 6:32632718 | 0 | 1 | 0 |
| 11:1643246 | 0 | 1 | 0 |
| 3:138347988 | 0 | 1 | 0 |
| 6:32632605 | 0 | 0 | 4 |
| X:57934522 | 13 | 0 | 0 |
| X:8763354 | 8 | 0 | 0 |
| 12:49724085 | 1 | 0 | 0 |
| 6:32632721 | 0 | 1 | 0 |
| 17:39383156 | 0 | 0 | 2 |
| X:63411894 | 13 | 0 | 0 |
| 1:248616311 | 0 | 1 | 0 |
| X:38145708 | 18 | 0 | 0 |
| 2:74784038 | 0 | 1 | 0 |
| X:6451924 | 13 | 0 | 0 |
| 19:53116988 | 0 | 0 | 0 |
| 7:72413581 | 0 | 0 | 2 |
| 16:15463612 | 0 | 0 | 4 |
| 9:138150726 | 0 | 2 | 0 |
| 12:11214634 | 1 | 0 | 0 |
| 1:13449788 | 0 | 0 | 2 |
| 6:32609241 | 6 | 0 | 0 |
| 21:14987871 | 0 | 0 | 0 |
| 1:12856010 | 0 | 0 | 8 |
| 21:30250615 | 0 | 0 | 10 |
| 3:12783984 | 0 | 1 | 0 |
| 17:44409341 | 0 | 1 | 0 |
| 19:9407539 | 0 | 1 | 0 |
| 11:1266892 | 0 | 1 | 0 |
| 17:38975272 | 0 | 1 | 0 |
| 17:2966828 | 0 | 2 | 0 |
| X:48759553 | 10 | 0 | 0 |
| 14:106363818 | 0 | 1 | 0 |
| 12:9847364 | 0 | 1 | 0 |
| 21:28212625 | 0 | 1 | 0 |
| 16:28354353 | 0 | 3 | 0 |
| 22:21741331 | 2 | 0 | 0 |
| X:8433833 | 13 | 0 | 0 |
| 1:210267899 | 1 | 0 | 0 |
| 15:39893091 | 0 | 1 | 0 |
| 6:31324888 | 0 | 0 | 2 |
| 15:22369426 | 0 | 0 | 5 |
| 6:32557461 | 0 | 1 | 0 |
| 4:175899088 | 0 | 1 | 0 |
| 11:320606 | 0 | 1 | 0 |
| 4:155511824 | 0 | 1 | 0 |
| 22:20708977 | 5 | 1 | 0 |
| 14:106478194 | 0 | 0 | 9 |
| 6:31922453 | 1 | 0 | 0 |
| 8:145773344 | 1 | 0 | 0 |
| 3:126291219 | 1 | 0 | 0 |
| 17:73926121 | 0 | 2 | 0 |
| 1:173808512 | 1 | 0 | 0 |
| 5:73090261 | 0 | 4 | 0 |
| 14:70039805 | 14 | 1 | 0 |
| 19:50511000 | 1 | 0 | 0 |
| 3:195515460 | 0 | 1 | 0 |
| 6:31324024 | 0 | 0 | 20 |
| 8:133854769 | 2 | 0 | 0 |
| 22:20709228 | 0 | 1 | 0 |
| 7:150783922 | 0 | 0 | 0 |
| 2:87243501 | 1 | 0 | 0 |
| 16:66628 | 0 | 1 | 0 |
| 1:146414187 | 0 | 2 | 0 |
| X:140785741 | 0 | 0 | 4 |
| 14:106053461 | 0 | 0 | 2 |
| 4:3591069 | 0 | 0 | 0 |
| X:27765405 | 0 | 1 | 0 |
| 2:217498290 | 0 | 0 | 0 |
| 5:111756111 | 1 | 0 | 0 |
| 11:108183167 | 0 | 0 | 10 |
| 3:130360527 | 0 | 1 | 0 |
| 7:143269773 | 0 | 1 | 0 |
| 10:90582712 | 0 | 1 | 0 |
| 6:32609229 | 1 | 0 | 0 |
| 12:132628391 | 0 | 0 | 0 |
| 12:11187133 | 0 | 1 | 0 |
| 15:23407160 | 0 | 1 | 0 |
| 6:31324528 | 1 | 1 | 0 |
| 4:74124077 | 0 | 1 | 0 |
| 16:16278869 | 0 | 0 | 1 |
| X:18725922 | 0 | 1 | 0 |
| 5:74931677 | 1 | 0 | 0 |
| 7:101928495 | 0 | 0 | 2 |
| 6:32632647 | 0 | 1 | 0 |
| X:140994510 | 13 | 0 | 0 |
| 3:113222036 | 0 | 3 | 0 |
| 17:18997106 | 0 | 1 | 0 |
| 6:170871040 | 0 | 0 | 0 |
| 4:42020142 | 0 | 1 | 0 |
| X:55117864 | 8 | 0 | 0 |
| X:8434097 | 8 | 0 | 0 |
| 1:16899669 | 0 | 5 | 0 |
| 6:32010272 | 0 | 0 | 9 |
| X:140993957 | 0 | 2 | 0 |
| X:26212334 | 10 | 0 | 0 |
| 12:122685163 | 0 | 0 | 0 |
| 1:206669465 | 0 | 4 | 0 |
| 10:119774598 | 0 | 1 | 0 |
| 17:36339597 | 0 | 5 | 0 |
| 1:248084757 | 4 | 0 | 0 |
| 1:248737230 | 0 | 1 | 0 |
| 15:23685751 | 0 | 0 | 0 |
| 1:13670869 | 0 | 0 | 6 |
| 4:466365 | 0 | 2 | 0 |
| 17:39240795 | 6 | 0 | 0 |
| 3:75713654 | 0 | 0 | 0 |
| X:150832716 | 0 | 1 | 0 |
| 8:133175736 | 0 | 1 | 0 |
| 6:166736362 | 0 | 5 | 0 |
| 6:30131515 | 0 | 0 | 9 |
| 1:150199042 | 0 | 2 | 0 |
| 5:837556 | 0 | 1 | 0 |
| 14:106478244 | 1 | 0 | 0 |
| 1:12887224 | 1 | 0 | 0 |
| 5:176005562 | 1 | 0 | 0 |
| 18:166819 | 0 | 4 | 0 |
| 3:197579466 | 0 | 1 | 0 |
| 17:20768803 | 0 | 0 | 0 |
| 2:85549874 | 0 | 2 | 0 |
| 4:57361553 | 0 | 3 | 0 |
| 19:55286795 | 0 | 0 | 0 |
| 17:21319473 | 0 | 0 | 1 |
| 10:97744383 | 0 | 7 | 0 |
| X:149101934 | 8 | 0 | 0 |
| 15:23685168 | 0 | 0 | 0 |
| 7:100634601 | 0 | 1 | 0 |
| 3:195507756 | 1 | 0 | 0 |
| 11:120107411 | 0 | 8 | 0 |
| 16:1291308 | 0 | 0 | 0 |
| X:48269486 | 1 | 0 | 0 |
| 17:43924200 | 0 | 0 | 3 |
| 6:32552026 | 0 | 1 | 0 |
| X:57619276 | 12 | 0 | 0 |
| X:37028424 | 0 | 1 | 0 |
| 19:4512705 | 0 | 1 | 0 |
| 8:143867905 | 0 | 4 | 0 |
| X:8763146 | 13 | 0 | 0 |
| 1:16902894 | 0 | 3 | 0 |
| 10:89124819 | 0 | 1 | 0 |
| X:83116204 | 0 | 2 | 0 |
| 1:111957554 | 0 | 0 | 0 |
| 4:185655171 | 0 | 1 | 0 |
| 20:29623215 | 0 | 3 | 0 |
| X:140785739 | 0 | 0 | 3 |
| 17:44067400 | 0 | 0 | 5 |
| 22:23047144 | 0 | 1 | 0 |
| 1:248616275 | 0 | 1 | 0 |
| 2:90121985 | 2 | 0 | 0 |
| 19:38795587 | 2 | 0 | 0 |
| 1:143767513 | 0 | 0 | 5 |
| 6:32790089 | 0 | 2 | 0 |
| X:150349560 | 0 | 0 | 0 |
| 4:68995529 | 0 | 6 | 0 |
| 4:108641317 | 0 | 2 | 0 |
| 11:134856252 | 1 | 0 | 0 |
| 4:88537150 | 4 | 0 | 0 |
| 13:25670721 | 0 | 1 | 0 |
| 1:24393563 | 0 | 1 | 0 |
| 1:12853509 | 0 | 0 | 1 |
| 6:31324145 | 0 | 0 | 2 |
| 1:144880832 | 0 | 0 | 8 |
| 6:29364951 | 0 | 0 | 9 |
| X:57619156 | 13 | 0 | 0 |
| 4:155295076 | 0 | 7 | 0 |
| 6:32552080 | 0 | 1 | 0 |
| 12:53073837 | 1 | 0 | 0 |
| 6:32634313 | 0 | 4 | 0 |
| 22:20709296 | 0 | 2 | 0 |
| 3:75790774 | 0 | 0 | 0 |
| X:2836007 | 13 | 0 | 0 |
| 22:37964413 | 0 | 19 | 0 |
| 7:100552094 | 0 | 0 | 0 |
| 6:32632714 | 0 | 1 | 0 |
| 2:97877470 | 1 | 0 | 0 |
| 3:195501149 | 0 | 0 | 5 |
| 15:32690804 | 1 | 1 | 0 |
| 2:131245713 | 0 | 4 | 0 |
| 1:10699149 | 1 | 0 | 0 |
| 10:93962 | 0 | 1 | 0 |
| 21:46020527 | 0 | 0 | 10 |
| 6:32549351 | 0 | 1 | 0 |
| 11:56468561 | 0 | 0 | 4 |
| 1:90309204 | 0 | 1 | 0 |
| 1:12919682 | 0 | 0 | 1 |
| 2:40488155 | 0 | 1 | 0 |
| 7:100643282 | 0 | 1 | 0 |
| 11:56468452 | 0 | 0 | 0 |
| 19:55358734 | 0 | 0 | 0 |
| 6:29911228 | 0 | 0 | 11 |
| 16:28507458 | 0 | 0 | 0 |
| 19:38795554 | 1 | 0 | 0 |
| 16:55866949 | 1 | 0 | 0 |
| 7:6836259 | 0 | 1 | 0 |
| 11:56237608 | 0 | 0 | 0 |
| 11:1268931 | 0 | 1 | 0 |
| 19:48622427 | 0 | 7 | 0 |
| 22:25023893 | 3 | 1 | 0 |
| 2:240684619 | 0 | 1 | 0 |
| 1:13365913 | 0 | 1 | 0 |
| 12:50745783 | 0 | 1 | 0 |
| 11:117789327 | 0 | 12 | 0 |
| 19:55699454 | 0 | 0 | 2 |
| X:103495101 | 6 | 0 | 0 |
| 6:29911240 | 0 | 0 | 7 |
| 2:55887325 | 0 | 1 | 0 |
| 10:13699153 | 2 | 2 | 0 |
| 6:30954939 | 0 | 0 | 2 |
| 7:130418721 | 0 | 0 | 10 |
| 22:17264904 | 0 | 0 | 0 |
| 6:32549368 | 0 | 2 | 0 |
| 16:88717386 | 0 | 5 | 0 |
| 12:133049481 | 0 | 1 | 0 |
| 9:138150811 | 0 | 1 | 0 |
| 17:44108906 | 0 | 0 | 2 |
| 6:10906256 | 0 | 3 | 0 |
| 3:150421543 | 0 | 1 | 0 |
| 9:69256816 | 0 | 4 | 0 |
| 17:21203934 | 0 | 0 | 0 |
| 7:100552550 | 0 | 0 | 0 |
| 14:106478278 | 2 | 0 | 0 |
| 6:32552085 | 0 | 1 | 7 |
| 11:48346822 | 0 | 0 | 0 |
| 1:145301802 | 0 | 3 | 0 |
| 6:32552138 | 0 | 0 | 1 |
| X:148627335 | 13 | 0 | 0 |
| 19:54726839 | 1 | 0 | 0 |
| 17:21319943 | 0 | 0 | 1 |
| 3:195506740 | 0 | 2 | 0 |
| 19:14877848 | 0 | 4 | 0 |
| 11:111795085 | 0 | 4 | 0 |
| 6:31324206 | 0 | 2 | 0 |
| 19:54871664 | 0 | 6 | 5 |
| 7:100552435 | 0 | 0 | 0 |
| 4:88535832 | 0 | 5 | 0 |
| 3:195452872 | 0 | 1 | 0 |
| 4:3769307 | 0 | 1 | 0 |
| 6:31762843 | 0 | 0 | 6 |
| 1:201179060 | 0 | 0 | 0 |
| 7:102210332 | 1 | 0 | 0 |
| 4:36340755 | 0 | 3 | 0 |
| 1:13036447 | 0 | 0 | 6 |
| 17:56618030 | 0 | 3 | 0 |
| 15:38776827 | 0 | 2 | 0 |
| 15:23686217 | 0 | 1 | 0 |
| 3:195509515 | 0 | 0 | 0 |
| 16:12021356 | 0 | 0 | 0 |
| X:120009234 | 10 | 0 | 0 |
| 2:240981527 | 4 | 0 | 0 |
| 13:26148966 | 0 | 10 | 0 |
| 14:20528451 | 14 | 0 | 0 |
| 15:40453430 | 0 | 2 | 0 |
| 12:40876933 | 0 | 0 | 0 |
| X:8434191 | 13 | 0 | 0 |
| 6:5086211 | 0 | 0 | 1 |
| 12:7045900 | 0 | 0 | 0 |
| 6:31322303 | 0 | 0 | 3 |
| 19:11310189 | 0 | 1 | 0 |
| 22:20709307 | 0 | 1 | 0 |
| 1:144811810 | 0 | 4 | 3 |
| 3:122103120 | 0 | 2 | 0 |
| 2:1946788 | 0 | 0 | 0 |
| 1:201386916 | 0 | 2 | 0 |
| X:153416315 | 8 | 0 | 0 |
| 6:31324494 | 0 | 3 | 0 |
| 18:60646253 | 1 | 0 | 0 |
| 15:23686201 | 0 | 0 | 0 |
| 6:32548581 | 3 | 0 | 0 |
| X:140336584 | 3 | 0 | 0 |
| 11:48346681 | 0 | 0 | 0 |
| 18:3452223 | 1 | 0 | 0 |
| 2:70677994 | 0 | 6 | 0 |
| 17:29161960 | 3 | 0 | 0 |
| 16:57950057 | 0 | 5 | 0 |
| 17:15878112 | 6 | 1 | 0 |
| 6:31922456 | 2 | 0 | 0 |
| 15:20739887 | 0 | 1 | 0 |
| 12:4716513 | 0 | 1 | 0 |
| 8:145557497 | 0 | 0 | 1 |
| 9:96278538 | 1 | 0 | 0 |
| 19:48305436 | 0 | 2 | 0 |
| X:76849305 | 9 | 0 | 0 |
| 1:162367071 | 0 | 4 | 0 |
| 19:40368733 | 0 | 1 | 0 |
| 6:32497971 | 0 | 1 | 0 |
| 15:60690089 | 0 | 11 | 0 |
| 2:27804413 | 0 | 0 | 0 |
| 6:31324530 | 1 | 1 | 0 |
| 16:12021329 | 0 | 0 | 0 |
| 6:32632599 | 0 | 0 | 3 |
| 19:53116976 | 0 | 0 | 0 |
| 6:32557490 | 0 | 1 | 0 |
| 6:31324887 | 0 | 0 | 3 |
| 11:5510426 | 5 | 3 | 0 |
| 22:45789642 | 0 | 0 | 7 |
| 8:17270787 | 0 | 12 | 0 |
| 4:80905991 | 0 | 1 | 0 |
| 6:32632744 | 0 | 1 | 0 |
| 1:16895634 | 0 | 0 | 7 |
| 10:120919246 | 0 | 12 | 0 |
| 3:183465483 | 0 | 2 | 0 |
| 1:111783996 | 0 | 0 | 10 |
| 15:32685060 | 0 | 2 | 0 |
| 12:50749739 | 1 | 0 | 0 |
| 1:7890026 | 0 | 1 | 0 |
| 19:54724407 | 4 | 0 | 4 |
| 19:54778552 | 0 | 2 | 0 |
| 4:110678925 | 0 | 12 | 0 |
| 6:160211649 | 9 | 0 | 0 |
| X:8434106 | 13 | 0 | 0 |
| X:149681049 | 1 | 0 | 0 |
| 17:36047376 | 1 | 0 | 0 |
| 22:18835365 | 0 | 1 | 0 |
| 6:167595371 | 2 | 0 | 0 |
| 3:14106354 | 0 | 3 | 0 |
| 8:22526559 | 0 | 4 | 0 |
| 9:33572347 | 0 | 3 | 0 |
| 1:145338661 | 0 | 1 | 0 |
| 17:19713740 | 0 | 6 | 0 |
| 3:167742358 | 0 | 0 | 0 |
| 19:17049224 | 0 | 1 | 0 |
| 11:71276681 | 0 | 1 | 0 |
| 1:207700110 | 0 | 1 | 0 |
| 3:75786773 | 0 | 0 | 0 |
| 15:100794363 | 0 | 8 | 0 |
| 20:29628261 | 0 | 1 | 0 |
| 5:167913510 | 0 | 7 | 0 |
| 1:201179082 | 0 | 0 | 0 |
| 2:112536264 | 0 | 9 | 0 |
| 16:70884524 | 0 | 0 | 0 |
| 19:42342319 | 0 | 0 | 2 |
| 2:96610399 | 0 | 0 | 0 |
| 17:15517284 | 0 | 3 | 0 |
| 13:28674628 | 0 | 5 | 0 |
| 3:75714345 | 0 | 0 | 0 |
| X:57934588 | 13 | 0 | 0 |
| 9:139222174 | 0 | 1 | 0 |
| 15:31453147 | 0 | 1 | 0 |
| 12:11214212 | 0 | 0 | 3 |
| X:48433155 | 5 | 0 | 0 |
| 22:20709292 | 0 | 2 | 0 |
| 2:120199140 | 0 | 12 | 0 |
| X:8763143 | 8 | 0 | 0 |
| 7:152520490 | 2 | 1 | 0 |
| 1:13038327 | 0 | 2 | 0 |
| 7:102294074 | 0 | 1 | 0 |
| 21:45970771 | 5 | 0 | 0 |
| 1:148022984 | 0 | 1 | 0 |
| 22:24199704 | 0 | 2 | 0 |
| 10:89120396 | 2 | 0 | 0 |
| 1:201179097 | 0 | 1 | 0 |
| 17:39421952 | 0 | 0 | 0 |
| X:71424983 | 13 | 0 | 0 |
| 15:50875343 | 2 | 0 | 0 |
| 1:145273335 | 0 | 0 | 4 |
| 19:54725156 | 13 | 0 | 0 |
| 6:32557435 | 0 | 1 | 0 |
| 9:178947 | 0 | 1 | 0 |
| 5:140186982 | 2 | 0 | 0 |
| 19:49558210 | 0 | 3 | 0 |
| 21:46924434 | 0 | 1 | 0 |
| 9:130698029 | 0 | 6 | 0 |
| 11:48347363 | 0 | 0 | 0 |
| 14:60063472 | 0 | 12 | 0 |
| 6:31868075 | 0 | 0 | 1 |
| X:134948023 | 13 | 0 | 0 |
| 6:31675501 | 0 | 0 | 1 |
| 15:23686218 | 0 | 1 | 0 |
| 15:30700159 | 0 | 0 | 4 |
| 17:36474601 | 0 | 1 | 1 |
| 11:1264188 | 0 | 1 | 0 |
| 4:9217234 | 0 | 1 | 0 |
| 8:133047071 | 0 | 3 | 0 |
| 8:12041211 | 0 | 1 | 0 |
| 17:20768762 | 0 | 0 | 0 |
| 11:193722 | 0 | 0 | 1 |
| 1:144828683 | 0 | 0 | 0 |
| 15:21004910 | 0 | 1 | 0 |
| 11:94800748 | 0 | 0 | 0 |
| 6:32497988 | 7 | 1 | 0 |
| 11:1016629 | 0 | 1 | 0 |
| 2:228194480 | 0 | 10 | 0 |
| X:57619164 | 13 | 0 | 0 |
| 17:73725458 | 0 | 1 | 0 |
| 19:52888071 | 0 | 2 | 0 |
| 1:152327542 | 1 | 0 | 0 |
| 4:158238830 | 0 | 9 | 0 |
| 12:124835175 | 0 | 1 | 0 |
| 14:22476144 | 0 | 4 | 0 |
| 17:79912164 | 0 | 3 | 0 |
| 4:88537168 | 2 | 0 | 0 |
| 14:107178938 | 0 | 0 | 7 |
| 6:31079643 | 0 | 0 | 5 |
| 13:110839550 | 0 | 0 | 8 |
| 3:195515414 | 0 | 2 | 0 |
| 20:34084415 | 0 | 0 | 4 |
| 8:7353480 | 1 | 1 | 0 |
| 19:54726299 | 0 | 0 | 1 |
| 7:6803602 | 0 | 1 | 0 |
| X:57620701 | 6 | 0 | 0 |
| 19:49657526 | 0 | 1 | 0 |
| 19:40389741 | 0 | 1 | 0 |
| 4:88537411 | 0 | 1 | 0 |
| 15:34331123 | 0 | 0 | 0 |
| 15:77176158 | 0 | 1 | 0 |
| 2:191184475 | 0 | 6 | 0 |
| 11:5629607 | 0 | 8 | 0 |
| 6:29910731 | 0 | 0 | 3 |
| 20:33320379 | 0 | 1 | 0 |
| 4:175688141 | 0 | 1 | 0 |
| 19:18123738 | 0 | 1 | 0 |
| X:112022628 | 15 | 0 | 0 |
| 22:20458029 | 0 | 1 | 0 |
| 2:113135662 | 0 | 1 | 0 |
| 5:176026144 | 8 | 0 | 0 |
| 13:107145463 | 0 | 0 | 1 |
| 2:54558114 | 0 | 1 | 0 |
| 19:8567475 | 0 | 3 | 0 |
| 15:20740294 | 8 | 0 | 0 |
| 18:43420177 | 0 | 6 | 0 |
| 1:12853544 | 0 | 0 | 3 |
| 16:24583715 | 0 | 1 | 0 |
| 6:32363816 | 0 | 0 | 9 |
| 1:144874815 | 0 | 0 | 2 |
| 15:22383249 | 0 | 0 | 7 |
| 1:16905837 | 0 | 1 | 0 |
| 1:12855843 | 0 | 1 | 0 |
| 1:146409969 | 3 | 3 | 0 |
| 16:82203742 | 0 | 6 | 0 |
| 3:195506555 | 1 | 0 | 0 |
| 15:32690806 | 1 | 1 | 0 |
| 4:1388564 | 1 | 0 | 0 |
| 6:32975869 | 0 | 0 | 1 |
| 9:138151145 | 0 | 1 | 0 |
| 2:159663616 | 0 | 1 | 0 |
| X:15841211 | 13 | 0 | 0 |
| 14:106478252 | 1 | 0 | 0 |
| 11:134202030 | 0 | 1 | 0 |
| 7:100644027 | 0 | 1 | 0 |
| 12:50745791 | 0 | 1 | 0 |
| 1:148023067 | 0 | 1 | 0 |
| 1:145293490 | 0 | 0 | 6 |
| 16:767294 | 0 | 4 | 0 |
| 8:7673126 | 0 | 4 | 0 |
| 15:93616975 | 0 | 11 | 0 |
| X:151870070 | 13 | 0 | 0 |
| 16:66645 | 0 | 1 | 0 |
| 6:32549361 | 0 | 0 | 1 |
| 6:150268515 | 0 | 1 | 0 |
| 19:4512934 | 2 | 0 | 0 |
| 3:75713976 | 0 | 0 | 0 |
| 2:187559047 | 0 | 5 | 0 |
| 8:144940300 | 0 | 0 | 4 |
| 16:88594544 | 2 | 0 | 0 |
| 18:59895716 | 0 | 1 | 0 |
| 2:175292593 | 1 | 0 | 0 |
| 2:97817670 | 0 | 0 | 0 |
| 11:48346924 | 0 | 0 | 0 |
| 13:100888122 | 0 | 2 | 0 |
| 6:57398226 | 0 | 0 | 0 |
| X:48970568 | 13 | 0 | 0 |
| 11:3721868 | 0 | 1 | 0 |
| 22:21481188 | 0 | 3 | 0 |
| 1:179457753 | 0 | 0 | 0 |
| 1:86515834 | 0 | 1 | 0 |
| 16:71956529 | 0 | 3 | 0 |
| 1:76282174 | 0 | 1 | 0 |
| 4:6693798 | 2 | 0 | 0 |
| 2:219249889 | 0 | 1 | 0 |
| 2:132021781 | 3 | 3 | 0 |
| 5:176026129 | 9 | 0 | 0 |
| 15:90320173 | 4 | 0 | 0 |
| 2:238258814 | 0 | 2 | 0 |
| 17:4837181 | 0 | 0 | 0 |
| X:149101991 | 13 | 0 | 0 |
| 19:7051376 | 4 | 0 | 0 |
| 11:82973004 | 0 | 2 | 0 |
| 11:48346996 | 0 | 0 | 0 |
| 6:30892322 | 0 | 0 | 2 |
| 20:59827596 | 0 | 1 | 0 |
| X:6452180 | 13 | 0 | 0 |
| 17:18659367 | 8 | 1 | 0 |
| 11:985547 | 0 | 0 | 6 |
| 6:32552016 | 0 | 1 | 0 |
| 4:190884281 | 0 | 0 | 0 |
| 22:24300634 | 9 | 5 | 0 |
| X:153492716 | 2 | 1 | 0 |
| 9:33240225 | 0 | 1 | 0 |
| 16:2059737 | 2 | 0 | 0 |
| X:77298857 | 0 | 0 | 2 |
| 21:45553596 | 0 | 0 | 5 |
| 11:56143539 | 0 | 1 | 0 |
| 7:100646420 | 2 | 0 | 0 |
| 9:103348361 | 0 | 1 | 0 |
| 6:32486437 | 3 | 1 | 0 |
| X:27765435 | 13 | 0 | 0 |
| 6:32609192 | 0 | 7 | 0 |
| 6:31324200 | 0 | 2 | 0 |
| 6:31964331 | 0 | 1 | 0 |
| 8:61767061 | 0 | 1 | 0 |
| 15:28502279 | 0 | 0 | 8 |
| 6:30919391 | 0 | 0 | 2 |
| X:8434417 | 13 | 0 | 0 |
| 6:70733547 | 0 | 2 | 0 |
| 11:10602082 | 1 | 0 | 0 |
| 1:145367800 | 0 | 0 | 1 |
| 6:32557504 | 0 | 1 | 0 |
| X:153455654 | 12 | 0 | 0 |
| 11:60165353 | 12 | 0 | 0 |
| 9:138150752 | 0 | 2 | 0 |
| 5:140558315 | 0 | 4 | 0 |
| 4:68442968 | 0 | 1 | 0 |
| 19:501786 | 0 | 1 | 0 |
| 5:801236 | 0 | 1 | 0 |
| 17:80992932 | 0 | 0 | 9 |
| 2:120078778 | 0 | 1 | 0 |
| X:144329121 | 13 | 0 | 0 |
| 19:4512945 | 1 | 0 | 0 |
| 6:32299822 | 0 | 2 | 5 |
| 19:56274214 | 0 | 1 | 0 |
| 10:81465798 | 3 | 0 | 0 |
| 17:43923266 | 0 | 0 | 4 |
| 3:66396808 | 0 | 1 | 0 |
| 17:56386607 | 1 | 0 | 0 |
| 3:75787490 | 0 | 0 | 0 |
| 15:65994812 | 0 | 1 | 0 |
| 11:1018174 | 0 | 0 | 0 |
| 17:39253835 | 0 | 0 | 2 |
| 19:54849463 | 0 | 0 | 1 |
| 1:145293425 | 0 | 0 | 1 |
| 14:106791131 | 0 | 0 | 9 |
| 4:3590817 | 0 | 0 | 0 |
| 19:56274424 | 0 | 0 | 0 |
| 6:30994059 | 0 | 1 | 0 |
| 17:44071319 | 0 | 1 | 0 |
| 3:121396174 | 1 | 0 | 0 |
| X:57620870 | 2 | 0 | 0 |
| 16:16306059 | 1 | 0 | 0 |
| 6:31084945 | 0 | 4 | 0 |
| X:151935789 | 13 | 0 | 0 |
| 1:148025763 | 0 | 2 | 0 |
| 2:113127773 | 0 | 1 | 0 |
| 19:1535196 | 12 | 2 | 0 |
| 6:31922452 | 2 | 0 | 0 |
| 12:53189431 | 0 | 1 | 0 |
| 16:70896033 | 0 | 0 | 0 |
| 11:48347124 | 0 | 0 | 0 |
| 10:51768676 | 2 | 1 | 0 |
| 11:56468416 | 0 | 0 | 0 |
| X:103495190 | 13 | 0 | 0 |
| 9:138150849 | 0 | 2 | 0 |
| 21:46057625 | 0 | 1 | 0 |
| 1:246720798 | 0 | 0 | 10 |
| 6:29911225 | 0 | 0 | 1 |
| 3:195506983 | 0 | 0 | 0 |
| 17:39340848 | 0 | 5 | 0 |
| 22:42525132 | 0 | 0 | 3 |
| 2:132021815 | 10 | 0 | 0 |
| 6:31002527 | 0 | 0 | 4 |
| 6:32151443 | 0 | 0 | 2 |
| 14:78234796 | 0 | 1 | 0 |
| 1:207795320 | 0 | 12 | 0 |
| 10:124329710 | 0 | 9 | 0 |
| 20:60891963 | 0 | 1 | 0 |
| 15:23406994 | 0 | 1 | 0 |
| 1:236557771 | 0 | 5 | 0 |
| 12:133503670 | 0 | 0 | 0 |
| 3:75787099 | 0 | 0 | 0 |
| 6:30954738 | 0 | 0 | 6 |
| 12:40876740 | 0 | 1 | 0 |
| 3:75715033 | 0 | 0 | 0 |
| 9:88275882 | 0 | 1 | 0 |
| 20:31897554 | 0 | 1 | 0 |
| 20:29628271 | 0 | 1 | 0 |
| 9:178937 | 0 | 2 | 0 |
| 9:138150836 | 0 | 1 | 0 |
| 21:42717662 | 0 | 1 | 0 |
| 21:36042475 | 0 | 1 | 0 |
| X:13681504 | 7 | 0 | 0 |
| X:6451809 | 13 | 0 | 0 |
| 6:30882781 | 0 | 0 | 5 |
| 10:81608550 | 1 | 0 | 0 |
| X:299362 | 0 | 0 | 1 |
| 14:20014613 | 0 | 2 | 0 |
| 2:118575161 | 1 | 0 | 0 |
| X:150840706 | 2 | 0 | 0 |
| 3:195511451 | 0 | 1 | 0 |
| 15:38776830 | 0 | 1 | 0 |
| 2:207603221 | 0 | 1 | 0 |
| 3:57261947 | 0 | 1 | 0 |
| 16:21742179 | 0 | 1 | 0 |
| X:140335705 | 0 | 1 | 0 |
| 2:131221668 | 2 | 0 | 0 |
| 11:1651157 | 0 | 1 | 0 |
| X:112022631 | 13 | 0 | 0 |
| 7:100553002 | 0 | 0 | 0 |
| 7:135082953 | 0 | 1 | 0 |
| 6:31324207 | 0 | 2 | 0 |
| 10:63976918 | 5 | 0 | 0 |
| 3:14105894 | 0 | 3 | 0 |
| 16:1695395 | 0 | 1 | 0 |
| 19:40385103 | 10 | 2 | 0 |
| 14:106478302 | 4 | 0 | 0 |
| 17:67190540 | 2 | 0 | 0 |
| 21:43985958 | 0 | 2 | 0 |
| 16:1306355 | 0 | 1 | 0 |
| 7:5965288 | 0 | 2 | 0 |
| 11:1643267 | 0 | 1 | 0 |
| 21:42879909 | 0 | 5 | 0 |
| 6:31239050 | 0 | 0 | 5 |
| 7:75677504 | 0 | 1 | 0 |
| 15:34677517 | 8 | 0 | 0 |
| 2:21235475 | 0 | 0 | 10 |
| 4:144801641 | 0 | 1 | 0 |
| 6:32610535 | 0 | 1 | 0 |
| X:49189246 | 14 | 0 | 0 |
| 4:1388563 | 1 | 0 | 0 |
| 3:52829633 | 0 | 1 | 0 |
| X:47428432 | 1 | 0 | 0 |
| 1:1243191 | 0 | 1 | 0 |
| 10:116008497 | 0 | 3 | 0 |
| 15:34674008 | 0 | 1 | 0 |
| 6:52621101 | 0 | 1 | 0 |
| 6:133119564 | 0 | 3 | 0 |
| X:8434196 | 13 | 0 | 0 |
| 1:13695974 | 0 | 0 | 7 |
| 19:49657530 | 0 | 1 | 0 |
| X:8763351 | 13 | 0 | 0 |
| 11:56143562 | 0 | 3 | 0 |
| 6:33235755 | 0 | 0 | 8 |
| 19:54745507 | 1 | 0 | 0 |
| 22:25435028 | 0 | 0 | 0 |
| 19:42736267 | 0 | 0 | 10 |
| 15:22369290 | 0 | 0 | 1 |
| 16:1290947 | 0 | 1 | 0 |
| X:140994201 | 2 | 0 | 0 |
| 16:70954718 | 0 | 0 | 0 |
| 20:238438 | 0 | 0 | 0 |
| 6:32634302 | 0 | 5 | 0 |
| 3:75714819 | 0 | 0 | 0 |
| X:34149683 | 7 | 0 | 0 |
| 1:12855752 | 0 | 0 | 6 |
| 8:61767033 | 0 | 1 | 0 |
| 19:20989992 | 0 | 0 | 3 |
| 22:22899234 | 0 | 9 | 0 |
| 16:71026098 | 0 | 0 | 0 |
| 22:23089943 | 1 | 0 | 0 |
| 1:11561101 | 1 | 0 | 0 |
| 9:42410368 | 0 | 1 | 0 |
| 2:27804381 | 0 | 1 | 0 |
| 17:21319785 | 0 | 0 | 1 |
| 12:11286790 | 0 | 0 | 2 |
| 22:18727115 | 0 | 1 | 0 |
| X:49081291 | 0 | 0 | 1 |
| 14:106363843 | 0 | 1 | 0 |
| 16:1291182 | 0 | 1 | 0 |
| 13:114150296 | 0 | 1 | 0 |
| 15:74365090 | 1 | 0 | 0 |
| 19:56274525 | 0 | 1 | 0 |
| 9:69653122 | 0 | 2 | 0 |
| 1:120612013 | 0 | 1 | 0 |
| 1:86512536 | 0 | 2 | 0 |
| 1:152189055 | 0 | 2 | 0 |
| 7:100552117 | 0 | 0 | 0 |
| X:103495134 | 1 | 0 | 0 |
| 9:132872686 | 0 | 4 | 0 |
| 3:5025179 | 0 | 1 | 0 |
| 1:110203841 | 1 | 0 | 0 |
| 6:30994025 | 0 | 1 | 0 |
| 4:144920596 | 0 | 7 | 0 |
| 11:56468048 | 0 | 0 | 0 |
| 7:100552915 | 0 | 0 | 0 |
| 3:66419956 | 0 | 1 | 0 |
| 10:124331867 | 0 | 7 | 0 |
| 10:71647223 | 0 | 2 | 0 |
| 12:133049506 | 0 | 0 | 0 |
| 19:49114018 | 0 | 1 | 0 |
| 6:49931783 | 0 | 1 | 0 |
| 11:76928356 | 0 | 1 | 0 |
| 6:32549613 | 5 | 0 | 0 |
| 9:46386995 | 0 | 0 | 0 |
| X:2724760 | 0 | 12 | 0 |
| 4:88537297 | 1 | 0 | 0 |
| 16:70923598 | 0 | 0 | 0 |
| 3:195506788 | 0 | 1 | 0 |
| 5:113829121 | 0 | 0 | 10 |
| 22:44324730 | 0 | 4 | 0 |
| 8:70850464 | 11 | 0 | 0 |
| 12:40874799 | 0 | 0 | 0 |
| 7:22197488 | 0 | 1 | 0 |
| 1:12854286 | 0 | 1 | 0 |
| 18:51795967 | 1 | 0 | 0 |
| 22:23248549 | 0 | 0 | 7 |
| 1:13038202 | 0 | 1 | 10 |
| X:57620515 | 13 | 0 | 0 |
| 13:24468329 | 0 | 4 | 0 |
| 11:35473473 | 0 | 1 | 0 |
| 2:241624543 | 0 | 1 | 0 |
| 12:40852535 | 0 | 2 | 0 |
| 6:32557483 | 0 | 6 | 0 |
| 2:96780986 | 0 | 6 | 4 |
| 11:56143729 | 0 | 0 | 0 |
| 4:72669661 | 0 | 1 | 0 |
| 15:28566562 | 0 | 1 | 0 |
| 2:112551673 | 0 | 3 | 0 |
| 4:1388724 | 1 | 0 | 0 |
| 19:54744711 | 1 | 0 | 0 |
| 1:153907287 | 0 | 1 | 0 |
| 6:32632578 | 0 | 0 | 3 |
| 1:16913677 | 0 | 3 | 0 |
| 14:22236756 | 0 | 0 | 9 |
| 20:744577 | 0 | 1 | 0 |
| 19:501762 | 0 | 0 | 0 |
| X:51076295 | 8 | 0 | 0 |
| 21:46924425 | 0 | 1 | 0 |
| 4:184367561 | 9 | 0 | 0 |
| 6:31630241 | 0 | 0 | 2 |
| 17:56544280 | 0 | 0 | 1 |
| 16:77233394 | 0 | 1 | 0 |
| 17:44626347 | 0 | 0 | 7 |
| 1:1670958 | 0 | 1 | 0 |
| 12:80839397 | 0 | 1 | 0 |
| 12:109017674 | 0 | 1 | 0 |
| X:140994512 | 13 | 0 | 0 |
| 3:195515449 | 0 | 1 | 0 |
| 19:58038964 | 0 | 12 | 0 |
| 5:179070469 | 0 | 1 | 0 |
| 22:19885609 | 0 | 1 | 0 |
| 2:61413592 | 2 | 0 | 0 |
| 6:32489835 | 0 | 1 | 0 |
| 16:70884534 | 0 | 0 | 0 |
| X:8138607 | 10 | 0 | 0 |
| X:49076224 | 1 | 0 | 0 |
| 22:25023915 | 2 | 0 | 0 |
| X:136648991 | 13 | 0 | 0 |
| 19:53269683 | 1 | 0 | 0 |
| 19:55333275 | 0 | 0 | 0 |
| 11:1264569 | 0 | 1 | 0 |
| 12:29521193 | 0 | 6 | 0 |
| 11:1642989 | 0 | 1 | 0 |
| 22:50659208 | 0 | 1 | 0 |
| 9:18675897 | 0 | 1 | 0 |
| 3:75787949 | 0 | 0 | 0 |
| 1:201179120 | 0 | 1 | 0 |
| X:134948000 | 13 | 0 | 0 |
| 2:89417017 | 3 | 0 | 0 |
| 16:1509123 | 0 | 1 | 0 |
| 10:7749183 | 0 | 1 | 0 |
| 11:49059278 | 0 | 1 | 0 |
| 9:138151077 | 0 | 1 | 0 |
| 9:111945049 | 0 | 1 | 0 |
| 11:6007272 | 0 | 0 | 1 |
| 6:32489757 | 0 | 1 | 0 |
| 18:28956904 | 0 | 3 | 0 |
| 1:13038119 | 0 | 0 | 6 |
| 17:43923683 | 0 | 0 | 2 |
| 6:32489745 | 0 | 1 | 0 |
| 9:34459025 | 0 | 2 | 0 |
| 8:10467652 | 0 | 2 | 0 |
| 4:1388378 | 4 | 0 | 0 |
| 11:129991670 | 3 | 1 | 0 |
| 19:54722275 | 0 | 0 | 4 |
| 6:29910759 | 0 | 3 | 0 |
| 4:88537153 | 3 | 0 | 0 |
| 1:146400186 | 2 | 0 | 0 |
| 22:22550760 | 0 | 0 | 1 |
| 19:55237677 | 3 | 0 | 0 |
| 7:100642950 | 1 | 0 | 0 |
| 5:122165317 | 0 | 1 | 0 |
| 9:33548843 | 1 | 0 | 0 |
| X:57618721 | 1 | 0 | 0 |
| 11:1270767 | 0 | 1 | 0 |
| X:140993926 | 5 | 2 | 0 |
| 14:106815723 | 0 | 1 | 0 |
| X:134986700 | 0 | 2 | 0 |
| 4:144801662 | 0 | 0 | 0 |
| 19:4512891 | 2 | 0 | 0 |
| 1:36643703 | 3 | 0 | 0 |
| 17:21204210 | 0 | 0 | 0 |
| 17:44409255 | 0 | 1 | 0 |
| 20:61885767 | 0 | 1 | 0 |
| X:57934878 | 11 | 0 | 0 |
| 19:54724431 | 0 | 2 | 0 |
| 15:20740347 | 1 | 0 | 0 |
| 14:74060517 | 0 | 0 | 0 |
| 1:21809014 | 0 | 3 | 0 |
| 6:32609216 | 2 | 0 | 0 |
| 2:108479214 | 2 | 0 | 0 |
| 6:32485524 | 0 | 0 | 1 |
| 22:17264565 | 1 | 0 | 0 |
| 1:226924884 | 9 | 4 | 0 |
| 2:97915896 | 8 | 0 | 0 |
| X:150840699 | 13 | 0 | 0 |
| 11:62847453 | 0 | 2 | 0 |
| 11:640061 | 0 | 1 | 0 |
| 4:9236806 | 0 | 1 | 0 |
| 22:36598049 | 0 | 8 | 0 |
| 1:12954967 | 0 | 0 | 1 |
| 17:7343011 | 0 | 1 | 0 |
| 17:79395747 | 0 | 1 | 0 |
| 21:19751604 | 0 | 1 | 0 |
| 6:31324209 | 0 | 2 | 0 |
| 2:130832873 | 0 | 1 | 0 |
| 19:55536595 | 0 | 3 | 0 |
| 3:42733387 | 4 | 0 | 0 |
| X:100531446 | 11 | 0 | 0 |
| 15:32891501 | 0 | 1 | 0 |
| 6:32548632 | 0 | 0 | 1 |
| 9:99060704 | 0 | 1 | 0 |
| 14:94847415 | 0 | 0 | 4 |
| 16:70942688 | 0 | 0 | 0 |
| 6:32489734 | 0 | 1 | 0 |
| 22:39537380 | 0 | 0 | 0 |
| 12:40874795 | 0 | 0 | 0 |
| 12:40875401 | 0 | 0 | 0 |
| 6:170627691 | 0 | 0 | 0 |
| 22:21064273 | 0 | 0 | 0 |
| 2:113671410 | 0 | 1 | 0 |
| 15:21063501 | 0 | 1 | 0 |
| 10:51568378 | 0 | 7 | 0 |
| 6:151670332 | 2 | 0 | 0 |
| 3:75787405 | 0 | 0 | 0 |
| 1:12854306 | 0 | 1 | 0 |
| 6:31323958 | 0 | 0 | 4 |
| 12:118511661 | 0 | 0 | 0 |
| 1:148756515 | 0 | 0 | 9 |
| 5:53606295 | 0 | 10 | 0 |
| 3:195508549 | 0 | 0 | 0 |
| 19:54726816 | 0 | 0 | 6 |
| 7:100680472 | 1 | 0 | 0 |
| 1:13329350 | 0 | 0 | 7 |
| 4:9246121 | 0 | 1 | 0 |
| 5:180582260 | 3 | 1 | 0 |
| 2:108479165 | 1 | 0 | 0 |
| 16:47697618 | 0 | 1 | 0 |
| 22:20709210 | 0 | 0 | 0 |
| X:142596941 | 0 | 0 | 3 |
| 19:496532 | 0 | 2 | 0 |
| 19:45213778 | 0 | 1 | 0 |
| 18:55315943 | 0 | 1 | 0 |
| X:8434423 | 13 | 0 | 0 |
| 17:4837190 | 0 | 0 | 0 |
| X:70321147 | 51 | 0 | 0 |
| X:151870043 | 1 | 0 | 0 |
| 1:145330829 | 0 | 1 | 7 |
| X:57146357 | 7 | 0 | 0 |
| 6:84884494 | 1 | 0 | 0 |
| 3:195508526 | 0 | 0 | 0 |
| 6:32497984 | 0 | 1 | 0 |
| 9:33386510 | 0 | 0 | 0 |
| X:6452051 | 13 | 0 | 0 |
| 6:47548626 | 0 | 1 | 0 |
| 1:159796701 | 0 | 1 | 0 |
| 21:43411554 | 3 | 0 | 0 |
| X:103495109 | 13 | 0 | 0 |
| 17:39240796 | 3 | 0 | 0 |
| 12:133049453 | 0 | 0 | 0 |
| 6:31324531 | 1 | 4 | 0 |
| 17:39432000 | 0 | 0 | 0 |
| 1:15909700 | 0 | 1 | 0 |
| 1:152749003 | 2 | 0 | 0 |
| 12:42628030 | 0 | 0 | 0 |
| 9:35906595 | 0 | 0 | 0 |
| 17:39240737 | 6 | 0 | 0 |
| 3:75788010 | 0 | 0 | 0 |
| 21:45588146 | 0 | 2 | 0 |
| X:140335742 | 0 | 2 | 0 |
| 6:31324506 | 0 | 3 | 0 |
| 11:56143559 | 0 | 3 | 0 |
| X:57934493 | 35 | 0 | 0 |
| 19:50156736 | 0 | 1 | 0 |
| 2:165697678 | 0 | 1 | 0 |
| 2:89161069 | 0 | 0 | 10 |
| 14:22999305 | 0 | 1 | 0 |
| 11:71249605 | 0 | 1 | 0 |
| 6:31112737 | 0 | 0 | 5 |
| 11:124253100 | 0 | 1 | 0 |
| 11:76701606 | 0 | 11 | 0 |
| 1:147595042 | 2 | 0 | 0 |
| 20:60639800 | 1 | 0 | 0 |
| 15:20740292 | 8 | 0 | 0 |
| 16:90095620 | 0 | 0 | 0 |
| 12:50745821 | 0 | 1 | 0 |
| 6:31110391 | 0 | 0 | 8 |
| 12:11183255 | 0 | 0 | 4 |
| 13:114503880 | 3 | 0 | 0 |
| 16:863369 | 0 | 1 | 0 |
| 6:123696766 | 0 | 2 | 0 |
| 9:67968720 | 0 | 1 | 0 |
| 1:16909208 | 0 | 2 | 0 |
| 6:29910767 | 2 | 1 | 0 |
| 3:195515401 | 0 | 1 | 0 |
| 16:82203758 | 0 | 10 | 0 |
| 6:32548554 | 2 | 0 | 0 |
| 1:201178957 | 0 | 0 | 0 |
| X:9905197 | 12 | 0 | 0 |
| 19:55349320 | 0 | 0 | 0 |
| 6:32632748 | 0 | 1 | 0 |
| 1:22332008 | 5 | 0 | 0 |
| 16:72042682 | 0 | 9 | 0 |
| 7:75052102 | 0 | 0 | 6 |
| 13:114312354 | 0 | 0 | 10 |
| 7:120776097 | 0 | 2 | 0 |
| 2:240981513 | 6 | 0 | 0 |
| X:134872227 | 0 | 0 | 2 |
| 11:637373 | 1 | 0 | 0 |
| 7:6836330 | 0 | 1 | 0 |
| 2:228102723 | 0 | 2 | 0 |
| 5:68695940 | 0 | 0 | 10 |
| 14:106478183 | 0 | 0 | 10 |
| X:57936462 | 6 | 0 | 0 |
| 7:34874038 | 0 | 1 | 0 |
| 10:81608501 | 0 | 1 | 0 |
| X:51076083 | 4 | 0 | 0 |
| 2:135749123 | 0 | 1 | 0 |
| 1:148579636 | 0 | 1 | 0 |
| 21:46924429 | 0 | 0 | 0 |
| X:7171244 | 10 | 0 | 0 |
| 9:96439007 | 11 | 0 | 0 |
| 11:48347392 | 0 | 0 | 0 |
| 2:130869609 | 0 | 1 | 0 |
| 21:34614255 | 0 | 7 | 0 |
| X:6452086 | 13 | 0 | 0 |
| X:34149695 | 8 | 0 | 0 |
| X:52679429 | 0 | 1 | 0 |
| 22:20709238 | 0 | 2 | 0 |
| 17:36352489 | 0 | 0 | 3 |
| X:47428430 | 13 | 0 | 0 |
| 17:39421971 | 9 | 0 | 0 |
| 7:143956181 | 0 | 1 | 0 |
| 19:1009577 | 11 | 0 | 0 |
| 1:157566136 | 0 | 1 | 0 |
| 7:100552440 | 0 | 0 | 0 |
| 4:81529518 | 0 | 12 | 0 |
| 19:44590002 | 7 | 0 | 0 |
| X:295132 | 0 | 0 | 6 |
| 6:32552067 | 0 | 1 | 0 |
| 10:51371581 | 0 | 0 | 2 |
| 1:12952966 | 10 | 0 | 0 |
| X:57934904 | 13 | 0 | 0 |
| X:153455646 | 2 | 0 | 0 |
| 12:11461583 | 0 | 2 | 0 |
| 1:152186222 | 1 | 1 | 0 |
| 11:124253161 | 0 | 1 | 0 |
| 1:145299809 | 0 | 2 | 0 |
| 13:112722441 | 1 | 0 | 0 |
| 9:35906601 | 0 | 2 | 0 |
| 17:36365172 | 0 | 0 | 1 |
| 6:30893428 | 0 | 0 | 9 |
| 19:55020248 | 0 | 1 | 0 |
| 6:29910663 | 0 | 0 | 2 |
| 7:38309102 | 0 | 2 | 0 |
| 3:4767262 | 0 | 11 | 0 |
| 2:240982200 | 1 | 0 | 0 |
| X:140335706 | 0 | 1 | 0 |
| 8:10467605 | 0 | 2 | 0 |
| 22:36598081 | 0 | 2 | 0 |
| 1:12855774 | 0 | 0 | 2 |
| 15:20739883 | 0 | 1 | 0 |
| 2:217288388 | 0 | 1 | 0 |
| 1:248801950 | 0 | 1 | 0 |
| 16:12021337 | 0 | 1 | 0 |
| 15:23685828 | 0 | 0 | 0 |
| 19:14769339 | 0 | 12 | 0 |
| 5:77311343 | 2 | 0 | 0 |
| X:6451993 | 2 | 0 | 0 |
| 17:44248814 | 0 | 0 | 6 |
| 22:20367781 | 1 | 2 | 0 |
| 16:863362 | 6 | 0 | 0 |
| 6:32552130 | 0 | 0 | 2 |
| 10:70741323 | 0 | 1 | 0 |
| 4:177106013 | 3 | 0 | 0 |
| 6:31324004 | 0 | 0 | 30 |
| 11:59945745 | 0 | 2 | 0 |
| 16:70894024 | 0 | 0 | 0 |
| 14:106471448 | 0 | 1 | 0 |
| 14:106791006 | 0 | 0 | 6 |
| 6:167790110 | 0 | 1 | 0 |
| 2:96614326 | 0 | 0 | 0 |
| 16:70178401 | 0 | 2 | 0 |
| 15:22383189 | 0 | 0 | 5 |
| 6:165895824 | 0 | 2 | 0 |
| 4:88536919 | 0 | 1 | 0 |
| 22:22786753 | 1 | 0 | 0 |
| 18:30352065 | 0 | 0 | 0 |
| 14:67671483 | 0 | 0 | 0 |
| 6:55266625 | 0 | 1 | 0 |
| 7:100552338 | 0 | 0 | 0 |
| 22:24300059 | 2 | 0 | 0 |
| 4:88537135 | 3 | 0 | 0 |
| 7:143417322 | 0 | 2 | 0 |
| 3:66419942 | 0 | 1 | 0 |
| 15:28949049 | 0 | 1 | 0 |
| 7:25267963 | 0 | 0 | 8 |
| 1:145586679 | 1 | 0 | 0 |
| 4:7765495 | 0 | 3 | 0 |
| X:140993885 | 12 | 0 | 0 |
| 7:102309418 | 1 | 2 | 0 |
| 11:56468560 | 0 | 0 | 5 |
| 1:144873887 | 0 | 0 | 5 |
| 7:101194424 | 0 | 8 | 0 |
| 6:32634331 | 0 | 0 | 25 |
| 1:144912233 | 0 | 0 | 8 |
| 11:56143543 | 0 | 1 | 0 |
| 3:75787519 | 0 | 0 | 0 |
| 15:31369123 | 0 | 0 | 1 |
| X:100531443 | 19 | 0 | 0 |
| 17:44061023 | 0 | 0 | 8 |
| 8:12286189 | 0 | 1 | 0 |
| 2:90260243 | 0 | 1 | 0 |
| 16:70954915 | 0 | 0 | 0 |
| 19:43269704 | 0 | 4 | 0 |
| 5:176026138 | 0 | 5 | 0 |
| 1:13696090 | 2 | 0 | 0 |
| 16:3170252 | 1 | 0 | 0 |
| 11:14991539 | 0 | 0 | 10 |
| 6:29912348 | 0 | 0 | 6 |
| 5:140186986 | 3 | 0 | 0 |
| X:151935366 | 3 | 0 | 0 |
| 16:70972595 | 0 | 0 | 0 |
| X:103495207 | 11 | 0 | 0 |
| 21:34614250 | 0 | 6 | 0 |
| X:38145142 | 10 | 0 | 0 |
| 15:57967219 | 0 | 0 | 10 |
| 22:21481466 | 0 | 1 | 0 |
| X:139586509 | 11 | 0 | 0 |
| 15:23606546 | 0 | 2 | 0 |
| 19:55359255 | 0 | 0 | 0 |
| 22:21481386 | 0 | 1 | 0 |
| X:49126995 | 13 | 0 | 0 |
| 7:38279725 | 0 | 1 | 0 |
| 12:124798840 | 0 | 1 | 0 |
| 16:70993566 | 0 | 0 | 0 |
| 19:55178162 | 0 | 0 | 1 |
| 6:168294583 | 0 | 1 | 0 |
| 6:13794147 | 0 | 1 | 0 |
| 2:238999883 | 0 | 3 | 0 |
| 17:46115079 | 0 | 2 | 0 |
| 14:106805508 | 0 | 0 | 0 |
| 22:20456851 | 0 | 3 | 0 |
| 1:13695603 | 4 | 0 | 0 |
| 15:23406990 | 0 | 1 | 0 |
| 11:132200056 | 0 | 2 | 0 |
| 19:40384956 | 0 | 1 | 0 |
| 6:16327894 | 0 | 0 | 0 |
| X:151869998 | 9 | 0 | 0 |
| 6:29910358 | 0 | 0 | 1 |
| 6:31138371 | 0 | 0 | 4 |
| 17:34261831 | 0 | 1 | 0 |
| 19:35660508 | 0 | 10 | 0 |
| 19:1825928 | 2 | 0 | 0 |
| 22:20709318 | 0 | 1 | 0 |
| X:51075856 | 1 | 0 | 0 |
| 17:30351758 | 0 | 2 | 0 |
| 11:56237609 | 0 | 0 | 0 |
| 3:75787236 | 0 | 0 | 0 |
| 8:134256610 | 0 | 1 | 0 |
| 17:44117119 | 0 | 0 | 1 |
| 15:23407173 | 0 | 1 | 0 |
| 16:67229811 | 0 | 1 | 0 |
| 12:27867727 | 0 | 1 | 0 |
| X:100105159 | 5 | 0 | 0 |
| 7:154989982 | 0 | 5 | 0 |
| 6:31324154 | 0 | 0 | 2 |
| 3:46487937 | 0 | 1 | 0 |
| 11:1264545 | 0 | 1 | 0 |
| 12:11214632 | 1 | 0 | 0 |
| 7:100634611 | 0 | 1 | 0 |
| 19:1440856 | 2 | 0 | 0 |
| 2:97817659 | 0 | 0 | 0 |
| X:8433892 | 17 | 0 | 0 |
| X:152244509 | 0 | 2 | 0 |
| 1:13695579 | 8 | 0 | 0 |
| 6:32632724 | 0 | 0 | 1 |
| 11:640064 | 0 | 1 | 0 |
| 1:200867552 | 0 | 1 | 0 |
| 14:63735862 | 0 | 1 | 0 |
| 9:13112116 | 0 | 0 | 3 |
| 19:55021777 | 0 | 0 | 5 |
| X:8434199 | 4 | 0 | 0 |
| 4:108641300 | 0 | 6 | 0 |
| 1:179457784 | 0 | 0 | 0 |
| 13:39263714 | 0 | 0 | 1 |
| 8:79578393 | 0 | 1 | 0 |
| 17:39340729 | 1 | 0 | 0 |
| 10:43650737 | 0 | 1 | 0 |
| 8:70588878 | 0 | 1 | 0 |
| 15:86287867 | 0 | 1 | 0 |
| 1:148012531 | 0 | 0 | 9 |
| 17:20768788 | 0 | 0 | 0 |
| 5:74506658 | 0 | 6 | 0 |
| 6:32557487 | 0 | 2 | 0 |
| 5:179070464 | 0 | 1 | 0 |
| 7:100552675 | 0 | 0 | 0 |
| 2:97817647 | 0 | 0 | 0 |
| 10:82122737 | 1 | 0 | 0 |
| X:9656197 | 13 | 0 | 0 |
| 10:49386184 | 0 | 1 | 0 |
| 6:132045195 | 0 | 1 | 0 |
| X:114468509 | 15 | 0 | 0 |
| X:54841981 | 1 | 0 | 0 |
| 4:88535831 | 0 | 5 | 0 |
| 12:112036797 | 0 | 0 | 0 |
| 14:74060512 | 0 | 0 | 0 |
| X:8138270 | 1 | 0 | 0 |
| 3:75787476 | 0 | 0 | 0 |
| 19:40377034 | 1 | 0 | 0 |
| 3:195453064 | 0 | 0 | 7 |
| 1:169811668 | 0 | 1 | 0 |
| 19:8389898 | 2 | 1 | 0 |
| 17:74288372 | 0 | 0 | 0 |
| 6:31324547 | 0 | 3 | 0 |
| 6:160560883 | 3 | 0 | 0 |
| 22:21570832 | 0 | 1 | 0 |
| 16:71122408 | 0 | 0 | 0 |
| 15:82637611 | 0 | 0 | 1 |
| 22:20709201 | 0 | 0 | 0 |
| 6:26637796 | 0 | 1 | 0 |
| 17:39471781 | 2 | 0 | 0 |
| 17:21318698 | 0 | 0 | 2 |
| 10:88782111 | 0 | 1 | 0 |
| 15:28235773 | 0 | 0 | 9 |
| 10:104622661 | 0 | 1 | 0 |
| 7:154990003 | 0 | 4 | 0 |
| 9:70919114 | 0 | 1 | 0 |
| 9:67987865 | 0 | 4 | 0 |
| 3:48282695 | 0 | 5 | 0 |
| 16:70934994 | 0 | 0 | 0 |
| 9:33386511 | 0 | 0 | 0 |
| 6:29910773 | 3 | 1 | 0 |
| 6:30994024 | 0 | 1 | 0 |
| 19:20044902 | 2 | 0 | 0 |
| 15:34331125 | 0 | 0 | 0 |
| 19:48305538 | 0 | 1 | 0 |
| 14:60007408 | 0 | 0 | 0 |
| 5:23976115 | 0 | 1 | 0 |
| 7:142625249 | 0 | 0 | 1 |
| 1:144828784 | 0 | 0 | 2 |
| 4:337627 | 0 | 0 | 1 |
| 2:46707885 | 0 | 1 | 0 |
| 1:144990002 | 0 | 3 | 0 |
| 3:75787726 | 0 | 0 | 0 |
| 11:48387284 | 0 | 0 | 0 |
| 15:43922917 | 0 | 1 | 0 |
| X:57619399 | 1 | 0 | 0 |
| 12:40874777 | 0 | 0 | 0 |
| 6:32609224 | 1 | 0 | 0 |
| 5:79950715 | 0 | 3 | 0 |
| 1:26608867 | 0 | 1 | 0 |
| X:57619273 | 12 | 0 | 0 |
| 17:18682505 | 0 | 1 | 0 |
| 1:248652061 | 0 | 1 | 0 |
| 16:70975565 | 0 | 0 | 0 |
| 1:152129115 | 0 | 1 | 0 |
| 5:140558317 | 0 | 3 | 0 |
| 1:248652058 | 1 | 1 | 0 |
| X:100748078 | 0 | 0 | 2 |
| X:13680922 | 4 | 0 | 0 |
| X:153416346 | 8 | 0 | 0 |
| 6:32551960 | 0 | 1 | 0 |
| 22:30793137 | 0 | 6 | 0 |
| 2:240981556 | 4 | 0 | 0 |
| 2:240981968 | 2 | 0 | 0 |
| 18:14542948 | 0 | 0 | 9 |
| 1:201924660 | 0 | 1 | 0 |
| 22:37964419 | 0 | 7 | 0 |
| 14:107095156 | 1 | 1 | 0 |
| 11:76751587 | 0 | 0 | 5 |
| 9:67960920 | 0 | 1 | 0 |
| 9:138151161 | 0 | 1 | 0 |
| 17:64208285 | 0 | 1 | 0 |
| 22:44379838 | 0 | 1 | 0 |
| 7:74193668 | 0 | 1 | 0 |
| 11:117078702 | 0 | 1 | 0 |
| 1:147955256 | 6 | 0 | 0 |
| 14:105352889 | 5 | 0 | 0 |
| 11:5221442 | 3 | 0 | 0 |
| 2:164467320 | 0 | 1 | 0 |
| 19:36348058 | 0 | 0 | 0 |
| 1:248652071 | 0 | 0 | 0 |
| 3:195515459 | 0 | 1 | 0 |
| 3:195515413 | 0 | 3 | 0 |
| 6:32610436 | 0 | 0 | 3 |
| X:55103957 | 1 | 0 | 0 |
| X:8434406 | 10 | 0 | 0 |
| 6:32632753 | 0 | 3 | 0 |
| 19:49894152 | 0 | 10 | 0 |
| 7:100680525 | 1 | 0 | 0 |
| 1:201285759 | 0 | 0 | 1 |
| 19:43709654 | 0 | 10 | 0 |
| 19:54746081 | 0 | 0 | 0 |
| 15:23407162 | 0 | 1 | 0 |
| 4:1090625 | 0 | 1 | 0 |
| 11:48346916 | 0 | 0 | 0 |
| 4:25265395 | 1 | 0 | 0 |
| 16:68088485 | 0 | 4 | 0 |
| 1:150199051 | 8 | 3 | 0 |
| 7:72412705 | 0 | 1 | 0 |
| 15:22074657 | 0 | 2 | 0 |
| 18:72201918 | 0 | 5 | 0 |
| 16:1279909 | 0 | 1 | 0 |
| 15:43922918 | 0 | 1 | 0 |
| 19:1482415 | 0 | 1 | 0 |
| 13:24471039 | 1 | 0 | 0 |
| 1:12980074 | 0 | 0 | 4 |
| 11:1017042 | 0 | 0 | 0 |
| 3:33134710 | 2 | 0 | 0 |
| 5:833915 | 13 | 5 | 0 |
| 6:170871046 | 0 | 1 | 0 |
| 1:12887221 | 1 | 0 | 0 |
| 17:39394674 | 0 | 0 | 7 |
| 1:248084747 | 2 | 0 | 0 |
| 17:21319007 | 0 | 0 | 1 |
| 3:124482503 | 3 | 0 | 0 |
| 6:29364835 | 0 | 0 | 7 |
| 16:71098649 | 0 | 0 | 0 |
| 16:69493016 | 0 | 2 | 0 |
| 4:78987157 | 0 | 8 | 0 |
| X:37028388 | 0 | 1 | 0 |
| 21:35469193 | 0 | 0 | 5 |
| 1:160209885 | 1 | 0 | 0 |
| 6:39048491 | 0 | 8 | 0 |
| 1:248737343 | 0 | 1 | 0 |
| 11:55029787 | 0 | 1 | 0 |
| 9:79318378 | 9 | 0 | 0 |
| 12:72070509 | 1 | 0 | 0 |
| 22:20710802 | 0 | 1 | 0 |
| 2:31567562 | 0 | 1 | 0 |
| 19:40399814 | 3 | 0 | 0 |
| 14:70039809 | 15 | 9 | 0 |
| X:114141475 | 3 | 0 | 0 |
| 16:29063445 | 6 | 0 | 0 |
| 3:14106037 | 0 | 3 | 0 |
| 15:34673973 | 8 | 1 | 0 |
| 17:39240661 | 0 | 0 | 1 |
| 14:77697967 | 0 | 0 | 10 |
| 15:93198684 | 6 | 0 | 0 |
| 4:175899102 | 0 | 2 | 0 |
| 13:46108854 | 0 | 10 | 0 |
| 1:115236057 | 0 | 1 | 0 |
| 6:32097290 | 0 | 0 | 2 |
| 1:12853415 | 0 | 0 | 2 |
| 3:195515386 | 0 | 1 | 0 |
| 22:21570790 | 0 | 1 | 0 |
| 1:16892277 | 0 | 0 | 0 |
| 17:19729495 | 0 | 4 | 0 |
| 12:10763236 | 0 | 8 | 0 |
| X:48269487 | 1 | 0 | 0 |
| 12:40876757 | 0 | 0 | 0 |
| 1:1431165 | 1 | 0 | 0 |
| 10:97990583 | 0 | 6 | 0 |
| 7:76144560 | 0 | 0 | 8 |
| 16:80584432 | 0 | 1 | 0 |
| 3:87276699 | 0 | 5 | 0 |
| 14:74010331 | 0 | 6 | 0 |
| 17:39253815 | 0 | 0 | 8 |
| 15:22473106 | 3 | 0 | 1 |
| 7:142498751 | 0 | 0 | 2 |
| 22:20710975 | 0 | 0 | 0 |
| 7:130418720 | 0 | 0 | 9 |
| 6:32632589 | 0 | 0 | 3 |
| 6:32629755 | 0 | 0 | 7 |
| 14:22133416 | 0 | 0 | 1 |
| 21:46057619 | 0 | 1 | 0 |
| 18:20953720 | 0 | 3 | 0 |
| 1:160580549 | 0 | 1 | 0 |
| 11:48347419 | 0 | 0 | 0 |
| 6:32489753 | 0 | 1 | 0 |
| X:153657145 | 1 | 0 | 0 |
| 5:72894207 | 0 | 8 | 0 |
| 11:3661586 | 0 | 1 | 0 |
| 5:7891506 | 0 | 1 | 0 |
| 7:100680490 | 1 | 0 | 0 |
| 10:122216862 | 0 | 1 | 0 |
| 15:23603641 | 0 | 5 | 0 |
| 7:100549914 | 0 | 0 | 0 |
| 1:248722722 | 0 | 1 | 0 |
| 11:68777323 | 0 | 1 | 0 |
| 19:50881825 | 1 | 2 | 0 |
| 7:100642986 | 0 | 2 | 0 |
| 3:75787516 | 0 | 0 | 0 |
| 15:43817807 | 1 | 0 | 0 |
| 1:110203845 | 1 | 0 | 0 |
| 1:26608889 | 0 | 1 | 0 |
| 18:30352078 | 0 | 5 | 0 |
| 1:157489535 | 0 | 3 | 0 |
| 9:138150833 | 0 | 1 | 0 |
| 5:140186988 | 3 | 0 | 0 |
| 7:5352809 | 0 | 1 | 0 |
| 3:121361787 | 0 | 3 | 0 |
| 6:32497968 | 0 | 1 | 0 |
| 9:138151147 | 0 | 1 | 0 |
| 1:13365924 | 0 | 1 | 0 |
| 20:60904111 | 0 | 1 | 0 |
| 1:12887227 | 1 | 0 | 0 |
| 21:35467645 | 0 | 0 | 4 |
| X:102841883 | 11 | 0 | 0 |
| 15:22473063 | 3 | 0 | 0 |
| X:151935354 | 13 | 0 | 0 |
| 15:23685920 | 0 | 1 | 0 |
| 8:104415415 | 0 | 1 | 0 |
| 11:772490 | 0 | 3 | 0 |
| 10:88419738 | 0 | 1 | 0 |
| 6:10891781 | 0 | 5 | 0 |
| 14:22102374 | 0 | 0 | 9 |
| 19:55246731 | 0 | 0 | 5 |
| 3:185198305 | 1 | 0 | 0 |
| 9:46390597 | 0 | 2 | 0 |
| 2:231258150 | 0 | 7 | 0 |
| 5:14488196 | 1 | 0 | 0 |
| 1:148756664 | 3 | 2 | 0 |
| 19:54745525 | 1 | 0 | 0 |
| 6:32609212 | 3 | 0 | 0 |
| X:103495268 | 26 | 0 | 0 |
| 2:109297180 | 0 | 1 | 0 |
| 17:39305773 | 1 | 0 | 0 |
| 6:32486438 | 0 | 1 | 0 |
| 8:87666251 | 0 | 10 | 0 |
| 3:195512948 | 0 | 1 | 0 |
| 11:7847466 | 0 | 0 | 6 |
| 19:54726630 | 0 | 1 | 0 |
| 19:54849481 | 0 | 0 | 1 |
| 15:54841874 | 0 | 2 | 0 |
| 19:9801445 | 1 | 0 | 0 |
| 1:152280665 | 0 | 0 | 0 |
| 6:88108051 | 11 | 0 | 0 |
| 12:49719940 | 0 | 1 | 0 |
| 14:74060513 | 0 | 0 | 0 |
| 1:12855844 | 0 | 1 | 0 |
| X:140993989 | 0 | 0 | 0 |
| 18:14513675 | 4 | 0 | 0 |
| 18:31319480 | 0 | 0 | 0 |
| 1:153320372 | 0 | 9 | 0 |
| 6:32336586 | 0 | 9 | 10 |
| 1:13329337 | 0 | 0 | 8 |
| 11:63472357 | 0 | 1 | 0 |
| 1:111957563 | 0 | 0 | 0 |
| X:102192380 | 2 | 0 | 0 |
| 4:337634 | 0 | 0 | 1 |
| 7:100551843 | 0 | 0 | 0 |
| 7:128140982 | 0 | 1 | 0 |
| X:100524197 | 0 | 3 | 0 |
| X:134947965 | 12 | 0 | 0 |
| 6:29342775 | 0 | 0 | 7 |
| 1:12854401 | 0 | 0 | 2 |
| 22:21579717 | 0 | 1 | 0 |
| X:57619168 | 13 | 0 | 0 |
| 7:100637605 | 1 | 0 | 0 |
| 3:75787926 | 0 | 0 | 0 |
| 6:32609223 | 1 | 0 | 0 |
| 22:20709242 | 0 | 2 | 0 |
| 12:40874772 | 0 | 0 | 0 |
| 6:32497904 | 0 | 0 | 2 |
| 4:3590730 | 0 | 0 | 0 |
| 13:25670370 | 0 | 1 | 0 |
| 6:57467100 | 0 | 11 | 0 |
| X:2778076 | 11 | 0 | 0 |
| 12:8325226 | 0 | 1 | 0 |
| 6:31170528 | 0 | 0 | 8 |
| 15:70975111 | 0 | 1 | 0 |
| 1:33777672 | 1 | 0 | 0 |
| 19:4512933 | 2 | 0 | 0 |
| 6:31964330 | 0 | 1 | 0 |
| 7:100551842 | 0 | 0 | 0 |
| X:83361968 | 9 | 0 | 0 |
| 4:26673787 | 0 | 12 | 0 |
| 22:44324727 | 0 | 4 | 0 |
| 19:17049230 | 0 | 1 | 0 |
| 1:150199045 | 0 | 2 | 0 |
| 15:34820404 | 0 | 3 | 0 |
| 7:48545976 | 0 | 4 | 3 |
| 16:4386814 | 0 | 0 | 5 |
| 5:146619206 | 0 | 2 | 0 |
| 10:37488689 | 0 | 2 | 0 |
| 6:57244780 | 0 | 0 | 0 |
| 1:28286610 | 1 | 0 | 0 |
| 4:79792142 | 0 | 0 | 0 |
| 11:56237606 | 0 | 0 | 0 |
| 7:21904233 | 0 | 1 | 0 |
| 3:75787870 | 0 | 0 | 0 |
| 13:73366609 | 0 | 0 | 0 |
| 2:27804371 | 0 | 1 | 0 |
| X:150840695 | 1 | 0 | 0 |
| 8:12043908 | 1 | 0 | 0 |
| 4:109745336 | 0 | 1 | 0 |
| 17:3323930 | 0 | 0 | 0 |
| 5:140594388 | 4 | 0 | 0 |
| 19:44778799 | 4 | 2 | 0 |
| 17:20768761 | 0 | 0 | 0 |
| 9:97080947 | 14 | 2 | 0 |
| 7:6798735 | 0 | 1 | 0 |
| 7:154429560 | 0 | 11 | 0 |
| 1:212799642 | 0 | 0 | 0 |
| 16:70896118 | 0 | 0 | 0 |
| 2:96610420 | 0 | 0 | 0 |
| 2:89417049 | 5 | 0 | 0 |
| 9:138836946 | 3 | 1 | 0 |
| 1:201179056 | 0 | 0 | 0 |
| 6:28294209 | 0 | 1 | 0 |
| 6:31237776 | 0 | 0 | 1 |
| 10:46967672 | 0 | 0 | 8 |
| 19:55327960 | 5 | 0 | 0 |
| 14:60932752 | 0 | 3 | 0 |
| 10:51620361 | 0 | 0 | 1 |
| 2:202245442 | 0 | 1 | 0 |
| 22:26862212 | 0 | 12 | 0 |
| 2:131221241 | 1 | 0 | 0 |
| 6:32609181 | 0 | 2 | 7 |
| 2:107049714 | 2 | 0 | 0 |
| 1:117150617 | 0 | 0 | 0 |
| X:55515165 | 7 | 0 | 0 |
| 3:195511142 | 0 | 0 | 1 |
| 17:41121195 | 0 | 1 | 0 |
| 11:71249152 | 11 | 2 | 0 |
| 1:148343714 | 0 | 0 | 8 |
| 1:13448548 | 0 | 2 | 6 |
| 2:96614322 | 0 | 0 | 0 |
| X:8763195 | 13 | 0 | 0 |
| 19:48544837 | 0 | 4 | 0 |
| 2:228194481 | 0 | 10 | 0 |
| 1:152185816 | 1 | 0 | 0 |
| 14:106053322 | 5 | 0 | 0 |
| 17:44068924 | 0 | 0 | 7 |
| 7:51098573 | 0 | 0 | 0 |
| 17:44159849 | 0 | 0 | 5 |
| 21:45970772 | 6 | 0 | 0 |
| 19:55329922 | 0 | 0 | 6 |
| 17:43924130 | 0 | 0 | 4 |
| 20:62656027 | 0 | 1 | 0 |
| 14:106053289 | 4 | 0 | 0 |
| 8:12044328 | 1 | 0 | 0 |
| 6:30997589 | 0 | 0 | 0 |
| 6:36733132 | 0 | 9 | 0 |
| 14:21560770 | 2 | 2 | 0 |
| 1:148023662 | 0 | 0 | 0 |
| 9:138151117 | 0 | 1 | 0 |
| 11:48347358 | 0 | 0 | 0 |
| 1:144811827 | 0 | 1 | 0 |
| 16:28354319 | 0 | 1 | 0 |
| 10:124330427 | 0 | 10 | 0 |
| 17:1638999 | 0 | 1 | 0 |
| X:6452199 | 13 | 0 | 0 |
| 1:240370956 | 0 | 1 | 0 |
| 19:54726241 | 0 | 0 | 5 |
| 12:40876885 | 0 | 0 | 0 |
| 20:43280227 | 0 | 3 | 0 |
| 16:21747639 | 0 | 1 | 0 |
| 17:21215557 | 0 | 0 | 0 |
| 1:26608814 | 0 | 4 | 0 |
| X:6452077 | 13 | 0 | 0 |
| X:54783871 | 11 | 0 | 0 |
| 10:118368606 | 0 | 2 | 0 |
| 18:20716021 | 0 | 4 | 0 |
| 19:55317524 | 0 | 0 | 0 |
| 4:151719263 | 0 | 2 | 0 |
| 10:55581885 | 1 | 0 | 0 |
| 2:95539831 | 0 | 0 | 0 |
| 14:22102612 | 0 | 0 | 10 |
| 2:242163367 | 2 | 0 | 0 |
| 17:21203893 | 0 | 0 | 0 |
| 16:84030848 | 0 | 1 | 0 |
| 3:195506548 | 1 | 0 | 0 |
| 5:177161891 | 0 | 12 | 0 |
| 7:38279663 | 0 | 2 | 0 |
| 10:46248649 | 3 | 0 | 0 |
| 4:9250440 | 0 | 1 | 0 |
| 16:88599697 | 6 | 0 | 0 |
| 1:16905719 | 0 | 1 | 0 |
| 2:242035491 | 1 | 0 | 0 |
| 2:240981571 | 1 | 0 | 0 |
| X:8433906 | 13 | 0 | 0 |
| 17:39197601 | 0 | 3 | 0 |
| 11:56468202 | 0 | 0 | 0 |
| 15:31362352 | 0 | 0 | 4 |
| 3:75787880 | 0 | 0 | 0 |
| 6:132029865 | 0 | 1 | 0 |
| 6:31079889 | 0 | 0 | 8 |
| 2:113138479 | 2 | 0 | 0 |
| 19:54725756 | 1 | 0 | 0 |
| 17:39382942 | 0 | 0 | 3 |
| 7:102212939 | 0 | 1 | 0 |
| 1:145367719 | 0 | 1 | 0 |
| 7:142498738 | 0 | 0 | 7 |
| 11:103152919 | 0 | 2 | 0 |
| 12:51740410 | 5 | 0 | 0 |
| 19:41754687 | 0 | 1 | 0 |
| 16:72992611 | 1 | 0 | 0 |
| 7:7841338 | 0 | 2 | 0 |
| 5:13944512 | 0 | 9 | 0 |
| 6:32548556 | 2 | 0 | 0 |
| 4:122078317 | 1 | 0 | 0 |
| 19:20044901 | 1 | 0 | 0 |
| 12:11174753 | 0 | 0 | 1 |
| 20:170263 | 0 | 1 | 0 |
| 19:45004288 | 0 | 2 | 0 |
| 17:65212042 | 0 | 5 | 0 |
| 12:112375990 | 0 | 5 | 0 |
| X:38145121 | 7 | 0 | 0 |
| 6:30888169 | 0 | 0 | 8 |
| 22:20709282 | 0 | 1 | 0 |
| 19:50510999 | 1 | 0 | 0 |
| X:34148877 | 0 | 2 | 0 |
| 1:248637262 | 1 | 0 | 0 |
| 10:51226118 | 0 | 1 | 0 |
| 10:124340406 | 0 | 1 | 0 |
| X:49189258 | 13 | 0 | 0 |
| 5:137216525 | 0 | 1 | 0 |
| 6:32549357 | 0 | 1 | 5 |
| 1:16909129 | 0 | 2 | 0 |
| 7:72436652 | 7 | 5 | 0 |
| 3:28533658 | 0 | 2 | 0 |
| 19:40392359 | 2 | 2 | 0 |
| 19:55644325 | 0 | 8 | 0 |
| 2:131220699 | 0 | 1 | 0 |
| 17:72889685 | 0 | 5 | 9 |
| 8:7694034 | 0 | 4 | 0 |
| 19:54744195 | 2 | 1 | 0 |
| 19:868421 | 0 | 0 | 1 |
| 1:145349719 | 0 | 3 | 0 |
| 1:13052789 | 0 | 3 | 0 |
| 5:76704849 | 0 | 0 | 10 |
| 1:111957558 | 0 | 0 | 0 |
| 11:46724728 | 5 | 0 | 0 |
| 15:78441769 | 0 | 7 | 0 |
| 4:109841743 | 0 | 3 | 0 |
| 2:130832256 | 0 | 2 | 0 |
| 21:46908355 | 0 | 6 | 0 |
| 19:53116972 | 0 | 0 | 0 |
| 7:100634244 | 0 | 1 | 0 |
| 16:28468295 | 1 | 0 | 0 |
| 1:117452869 | 0 | 1 | 0 |
| 19:48364367 | 0 | 1 | 0 |
| 3:195513345 | 0 | 0 | 2 |
| 3:195512965 | 0 | 3 | 0 |
| 6:31846741 | 0 | 3 | 0 |
| 9:67968798 | 0 | 1 | 0 |
| 19:30018208 | 0 | 2 | 0 |
| 11:95825407 | 11 | 9 | 0 |
| 4:70898903 | 0 | 1 | 0 |
| 19:40368498 | 0 | 0 | 0 |
| 22:16277852 | 0 | 0 | 0 |
| 19:54745496 | 5 | 0 | 0 |
| 9:138151184 | 0 | 1 | 0 |
| X:8763149 | 4 | 0 | 0 |
| 17:40926695 | 0 | 1 | 0 |
| 7:143270001 | 4 | 0 | 0 |
| 19:4513033 | 1 | 0 | 0 |
| 11:320649 | 1 | 0 | 0 |
| 3:37367997 | 0 | 1 | 0 |
| 3:75787464 | 0 | 0 | 0 |
| 6:42075114 | 0 | 0 | 0 |
| X:8138641 | 0 | 1 | 0 |
| X:6451839 | 13 | 0 | 0 |
| 5:100222234 | 0 | 1 | 0 |
| 5:60628168 | 0 | 0 | 0 |
| 5:1073891 | 0 | 1 | 0 |
| 6:31324523 | 3 | 0 | 0 |
| 6:32012333 | 0 | 1 | 0 |
| 6:29910777 | 2 | 1 | 0 |
| 19:49447750 | 1 | 0 | 0 |
| 14:22476139 | 5 | 0 | 0 |
| X:77392419 | 2 | 0 | 0 |
| 1:50884784 | 0 | 1 | 0 |
| 7:55238874 | 0 | 7 | 0 |
| X:8138662 | 13 | 0 | 0 |
| 14:106815970 | 1 | 0 | 0 |
| 5:140573991 | 0 | 0 | 2 |
| 9:138151169 | 0 | 1 | 0 |
| 6:32497913 | 0 | 2 | 0 |
| 15:35271874 | 1 | 0 | 0 |
| X:55103931 | 13 | 0 | 0 |
| 7:100552135 | 0 | 0 | 0 |
| 14:107095153 | 0 | 1 | 0 |
| 1:144340089 | 0 | 1 | 0 |
| 3:59997112 | 0 | 2 | 0 |
| 7:100552984 | 0 | 0 | 0 |
| 1:7890055 | 0 | 1 | 0 |
| 1:201179121 | 0 | 1 | 0 |
| 5:741736 | 0 | 10 | 0 |
| 7:158664076 | 3 | 0 | 0 |
| 18:51795960 | 14 | 0 | 0 |
| 3:195507798 | 1 | 0 | 0 |
| 3:195507846 | 1 | 0 | 0 |
| 17:21318897 | 0 | 0 | 1 |
| 17:6515294 | 0 | 0 | 0 |
| 3:195507769 | 1 | 0 | 0 |
| 7:137128830 | 0 | 9 | 0 |
| 1:144828548 | 0 | 1 | 0 |
| 2:121981950 | 0 | 9 | 0 |
| 1:179457780 | 0 | 0 | 0 |
| 9:138151076 | 0 | 1 | 0 |
| 11:27384469 | 1 | 1 | 0 |
| 22:21742124 | 0 | 1 | 0 |
| X:103495163 | 13 | 0 | 0 |
| 4:1388379 | 4 | 0 | 0 |
| 17:17948475 | 0 | 2 | 0 |
| 11:33763565 | 0 | 1 | 0 |
| 20:2996497 | 0 | 8 | 0 |
| 6:31324615 | 0 | 0 | 1 |
| 7:141957504 | 0 | 4 | 0 |
| 11:48346551 | 0 | 0 | 0 |
| 10:51827896 | 0 | 1 | 0 |
| 6:32609286 | 0 | 0 | 0 |
| X:153416411 | 13 | 0 | 0 |
| 6:32489744 | 0 | 1 | 0 |
| 13:43639845 | 0 | 5 | 0 |
| X:2632482 | 0 | 7 | 0 |
| 5:112312676 | 0 | 2 | 0 |
| 11:48346669 | 0 | 0 | 0 |
| 7:38299789 | 0 | 1 | 0 |
| 2:201526330 | 0 | 5 | 0 |
| 12:118511677 | 0 | 1 | 0 |
| 12:11183451 | 0 | 0 | 3 |
| 9:33386465 | 0 | 0 | 0 |
| 12:101705993 | 0 | 1 | 0 |
| X:37028427 | 0 | 1 | 0 |
| 12:11175087 | 0 | 0 | 2 |
| 21:36042476 | 0 | 1 | 0 |
| 19:44778798 | 3 | 1 | 0 |
| 15:30699723 | 0 | 1 | 0 |
| 20:29623223 | 0 | 4 | 0 |
| 20:60891964 | 0 | 1 | 0 |
| 3:97983497 | 5 | 0 | 0 |
| 1:240370935 | 0 | 3 | 0 |
| 1:145343385 | 0 | 0 | 4 |
| 2:179621477 | 0 | 0 | 6 |
| 21:46521023 | 0 | 1 | 0 |
| 6:33048640 | 0 | 0 | 1 |
| 17:21215537 | 0 | 0 | 0 |
| 4:88537123 | 1 | 0 | 0 |
| X:57934556 | 13 | 0 | 0 |
| 1:148756499 | 0 | 0 | 8 |
| 6:31324549 | 0 | 2 | 0 |
| 1:145293566 | 0 | 0 | 3 |
| X:154456747 | 0 | 0 | 10 |
| 17:62892271 | 0 | 0 | 1 |
| 6:32497975 | 6 | 2 | 0 |
| 6:31324491 | 1 | 2 | 0 |
| 3:108557797 | 0 | 1 | 0 |
| 7:100549935 | 0 | 0 | 0 |
| 5:179071947 | 0 | 0 | 2 |
| 1:179457770 | 0 | 1 | 0 |
| 19:40392719 | 0 | 0 | 8 |
| 20:29625935 | 0 | 1 | 0 |
| X:150840707 | 3 | 0 | 0 |
| X:114425210 | 0 | 3 | 0 |
| 3:11596302 | 0 | 8 | 0 |
| 6:32363844 | 0 | 0 | 2 |
| 1:12854162 | 0 | 0 | 4 |
| 1:248084756 | 4 | 0 | 0 |
| 6:29364815 | 0 | 0 | 7 |
| 9:122275247 | 0 | 1 | 0 |
| 15:90260145 | 0 | 7 | 0 |
| 19:55290108 | 0 | 0 | 0 |
| X:103495220 | 9 | 0 | 0 |
| X:151936140 | 13 | 0 | 0 |
| 6:31964316 | 0 | 1 | 0 |
| 19:44351168 | 11 | 0 | 0 |
| 6:31324497 | 0 | 3 | 0 |
| 19:55281315 | 0 | 4 | 0 |
| 1:146400242 | 1 | 0 | 0 |
| 5:180687440 | 11 | 1 | 0 |
| 14:106382687 | 0 | 9 | 0 |
| 1:145311110 | 0 | 2 | 1 |
| 19:22363702 | 0 | 0 | 0 |
| 21:37692589 | 0 | 6 | 0 |
| 3:98520461 | 0 | 1 | 0 |
| 19:15272017 | 0 | 1 | 0 |
| 10:95389041 | 0 | 6 | 0 |
| 16:1306671 | 0 | 1 | 0 |
| 19:55329021 | 0 | 0 | 0 |
| 2:24390517 | 0 | 12 | 0 |
| 11:55606812 | 1 | 0 | 0 |
| 6:32632703 | 0 | 1 | 0 |
| 5:150282713 | 2 | 0 | 0 |
| 1:12854370 | 0 | 0 | 5 |
| 10:81608553 | 1 | 0 | 0 |
| 19:54744710 | 1 | 0 | 0 |
| X:7811288 | 0 | 1 | 0 |
| 1:144811805 | 3 | 0 | 0 |
| X:134947996 | 13 | 0 | 0 |
| 2:240982379 | 1 | 0 | 0 |
| X:140994535 | 11 | 0 | 0 |
| 14:106478303 | 3 | 0 | 0 |
| 4:1388324 | 0 | 0 | 0 |
| 11:48347067 | 0 | 0 | 0 |
| 15:48443699 | 0 | 0 | 10 |
| 6:32549584 | 0 | 1 | 0 |
| 20:31981849 | 0 | 10 | 0 |
| 18:31319486 | 0 | 0 | 0 |
| 11:4976013 | 0 | 2 | 0 |
| 9:134401335 | 0 | 11 | 0 |
| 9:38411408 | 1 | 0 | 0 |
| 9:123291036 | 0 | 1 | 0 |
| X:49846390 | 0 | 0 | 8 |
| 6:32486358 | 1 | 0 | 0 |
| 11:122709291 | 0 | 1 | 0 |
| 1:145296403 | 0 | 1 | 0 |
| 3:57484251 | 0 | 1 | 0 |
| 5:175533585 | 2 | 0 | 0 |
| X:38146052 | 8 | 0 | 0 |
| 1:143906122 | 1 | 3 | 0 |
| 1:12980127 | 0 | 0 | 8 |
| 14:58598408 | 1 | 0 | 0 |
| X:6451843 | 7 | 0 | 0 |
| 3:75787702 | 0 | 0 | 0 |
| 22:20709231 | 0 | 2 | 0 |
| X:57619129 | 13 | 0 | 0 |
| 7:102125519 | 0 | 3 | 0 |
| 15:20740295 | 6 | 0 | 0 |
| 11:124266758 | 0 | 1 | 0 |
| 15:21071460 | 0 | 1 | 1 |
| 4:145041720 | 0 | 4 | 0 |
| 19:54724458 | 1 | 0 | 7 |
| 21:43985955 | 0 | 9 | 0 |
| 12:40876891 | 0 | 1 | 0 |
| 17:44073973 | 0 | 0 | 10 |
| 17:13972955 | 0 | 1 | 0 |
| 16:30671235 | 0 | 1 | 0 |
| 8:113253974 | 0 | 1 | 0 |
| 1:152327425 | 1 | 0 | 0 |
| 1:25573409 | 0 | 0 | 0 |
| 6:29911260 | 0 | 0 | 3 |
| 10:91487649 | 0 | 5 | 0 |
